# Zweifel olefination for C-glycosylation

**DOI:** 10.1038/s42004-024-01339-4

**Published:** 2024-12-21

**Authors:** Florian Trauner, Bilel Boutet, Fabian Pilz, Verena Weber, Dorian Didier

**Affiliations:** 1 Technische Universität Darmstadt, Clemens-Schöpf-Institut für Organische Chemie und Biochemie, Peter-Grünberg-Straße 4, 64287 Darmstadt, Germany; 2 Ludwig-Maximillians Universität, Department Chemie, Butenandtstraße 5, 81377 Munich, Germany; 3 Institute for Neuroscience and Medicine and Institute for Advanced Simulations (INM-9/IAS-5), Computational Biomedicine, Forschungszentrum Jülich, 52425 Jülich, Germany

**Keywords:** Synthetic chemistry methodology, Carbohydrate chemistry

## Abstract

C-glycosides are significant in medicinal chemistry due to their resistance to enzymatic hydrolysis, making them more stable and bioavailable compared to O-glycosides. Their unique structure also offers potential for developing drugs with improved therapeutic properties, particularly in treating diseases like diabetes and cancer. The main challenge in synthesizing C-glycosides lies in forming the carbon-carbon bond between the sugar and aglycone efficiently, while controlling the stereochemistry and minimizing side reactions. Starting from glycal derivatives, the Zweifel olefination presents an elegant opportunity to access *C*-glycosides in a selective manner. α-Lithiation of D-glucal, L-rhamnal, D-xylal and L-arabinal scaffolds was employed as a starting point in the synthesis of corresponding unsaturated aryl-, heteroaryl- and alkenyl-*C*-glycosides. This provides a straightforward strategy towards pharmacorelevant gliflozins and other unreported rhamnal- and xylal-analogs.

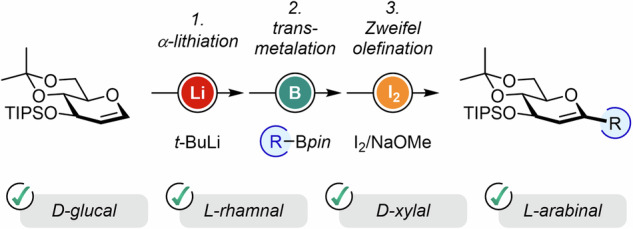

## Abstract (web summary)

Carbohydrates play a pivotal role in drug discovery and the pharmaceutical industry, however, synthetic methods for C-glycosylation remain challenging. Here, the authors report Zweifel olefination to access C-glycosides selectively by starting from unsaturated glycal derivatives.

## Introduction

Carbohydrates play a pivotal role in drug discovery and in the pharmaceutical industry, being omnipresent in bioactive compounds and natural derivatives^1–3^. They constitute the base of genetic material and are essential building blocks in epigenetic studies^4–6^.

**Fig. 1:**
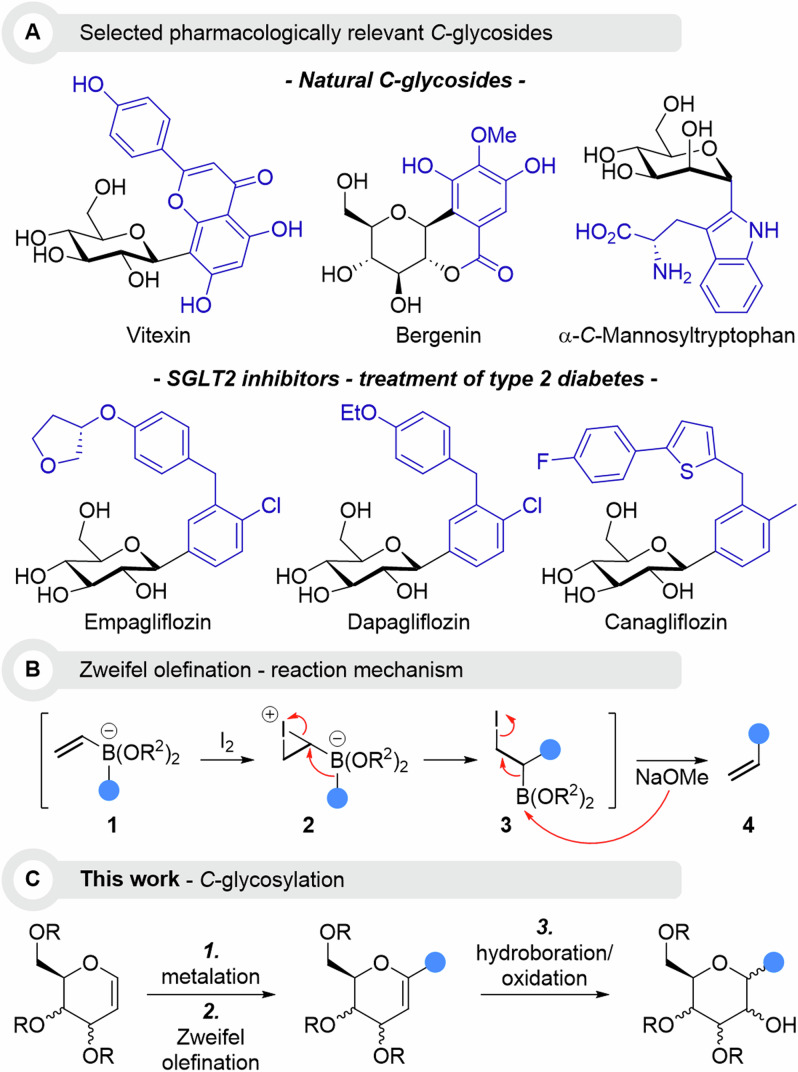
Our conceptual approach to C-glycosides in the context of current research interests. **A** Selected examples of pharmacologically relevant *C*-glycosides; **B** General mechanism of the Zweifel olefination; **C** Our strategic design to stereoselectively access *C*-glycosides via a sequence of Zweifel olefination/Brown oxidation.

Naturally occurring *C*-glycosides include Vitexin^7^, an apigenin (flavone) derivative found in different flowers and leaves, Bergenin^8^, isolated from *Bergenia* flowering plants and α-*C*-Mannosyltryptophan^9^, a product of post-translational modification by *C*-mannosyltransferases (Fig. 1A). Among synthetic pharmaceuticals of interest, gliflozins – compounds that we later access through our method - are a class of SGLT2 inhibitors used in the treatment of type 2 diabetes, as they exert effects on the sodium glucose cotransporter^10^. These *C*-glycosides are based on the glucose scaffolds, arylated at position 2, and include Empagliflozin, Dapagliflozin and Canagliflozin. More importantly, the FDA has recently approved Empagliflozin in the treatment of cardiovascular dysfunctions and chronic kidney diseases^11^.

When compared with *O*- and *N*-glycosylations, methods for *C*-glycosylation^12–14^ remain limited and mostly rely on either nucleophilic attack at the anomeric position or radical reactions^15^.

We envisioned that *C*-glycosides could easily be accessed from their parent unsaturated structures, strategically placing a double bond between C1 and C2. Such C=C bond can be regarded as polarized alkoxy-alkenes, prone to deprotonation at the α-position (C1) in the presence of appropriate bases, which provides a regioselective anchor for further functionalization. The carbohydrate integrity can then be restored through classical double bond manipulation such as the hydroboration/oxidation sequence (Fig. 1C)^16,17^.

Among the various elegant methods available for double bond manipulation, Zweifel olefination is most appealing because of its high efficiency, good versatility and stereocontrol. Pioneered by Zweifel in 1967^18,19^, it has received increasing attention over the last decade as an indispensable tool in total synthesis, especially by the group of Aggarwal^20–22^. In this reaction, a 1,2-metallate rearrangement is triggered upon addition of iodide onto a bis-organoborinate species **1** (Fig. 1B). The creation of an electrophilic site at the position α to the boron atoms via the intermediate iodonium species **2** promotes the key 1,2-rearrangement, transferring the organyl moiety in an intramolecular substitution reaction that leads to an α,β-iodoboronic ester intermediate **3**. An elimination then proceeds in the presence of a base, usually sodium methanolate, providing the formal coupling product between the organyl group and the former double bond.

Our recent interest on Zweifel olefination stems from the ability to perform C(sp^2^)-C(sp^2^) bond formation without the use of precious, often toxic transition metals. Aiming at improving its efficiency and applicability, we have established different methods that rely on the in situ formation of bisorganoborinate intermediates^23^ or the use of organocerium species, generated through halogen-cerium exchange^24,25^. Moreover, thorough investigations of reaction mechanism and de novo design of organoborate species led us to develop one-electron electro- and photocoupling processes based on an oxidative pseudo-1,2-metallate rearrangement^26–30^.

## Results and discussion

### Optimizations of reaction conditions

**Fig. 2:**
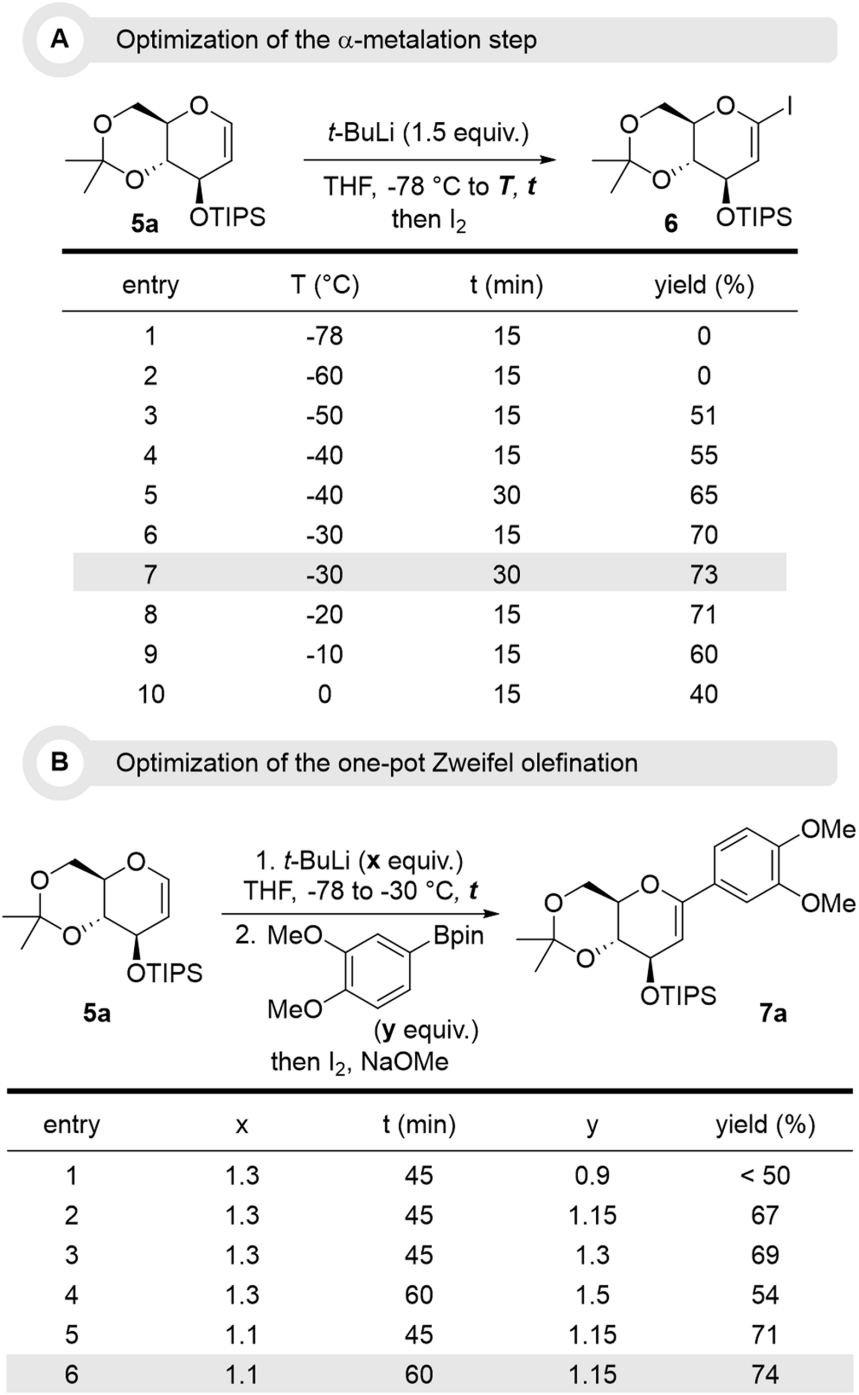
Stepwise optimization of reaction conditions. **A** Condition optimizations for the α-*O*-metalation step, controlled by iodolysis; **B** Optimization of the Zweifel olefination step using a *m*,*p*-dimethoxyarylboronic ester as benchmark reagent.

Metalation conditions were optimized first on a benchmark substrate, a protected D-glucal derivative (**5a**, Fig. 2A). In contrast with classical vinyl-ethers that can be easily metalated at the position α to the heteroatom, the metalation of protected glucal **5a** proved more difficult, with no conversion observed between −78 °C and −60 °C, using either *n*-BuLi, *s*-BuLi or even *t*-BuLi (entries 1 and 2, Fig. 2A). The first iodolysis product **6** was isolated in 51% yield when conducting the reaction for 15 min at −50 °C (entry 3). Warming the temperature up to −30 °C allowed the metalation to reach its maximum conversion, allowing for the isolation of **6** in 73% after 30 min (entry 7), while both lower and higher temperatures show any improvements (entries 5,6 and 8–10).

These first optimized conditions were then applied to the Zweifel olefination of compound **5a**, adding a boron pinacol ester as the organoboron partner (Fig. 2B). The reaction conducted in the presence of substoichiometric amount of boron species (0.9 equiv., entry 1, Fig. 2B) only yielded low amounts of desired products. However, employing a slight excess of ArBpin (entry 2) furnished **7a** in 67% yield. No significant improvement was noticed when further increasing the amount of organoboron species (entries 3 and 4). However, lowering the amount of based used in the initial α-metalation step to 1.15 equiv. provided **7a** in 71% (entry 5), value that was further increased to 74% under prolonged metalation time (60 min, entry 6).

### Scope of the reaction on D-Glucal derivatives

**Fig. 3:**
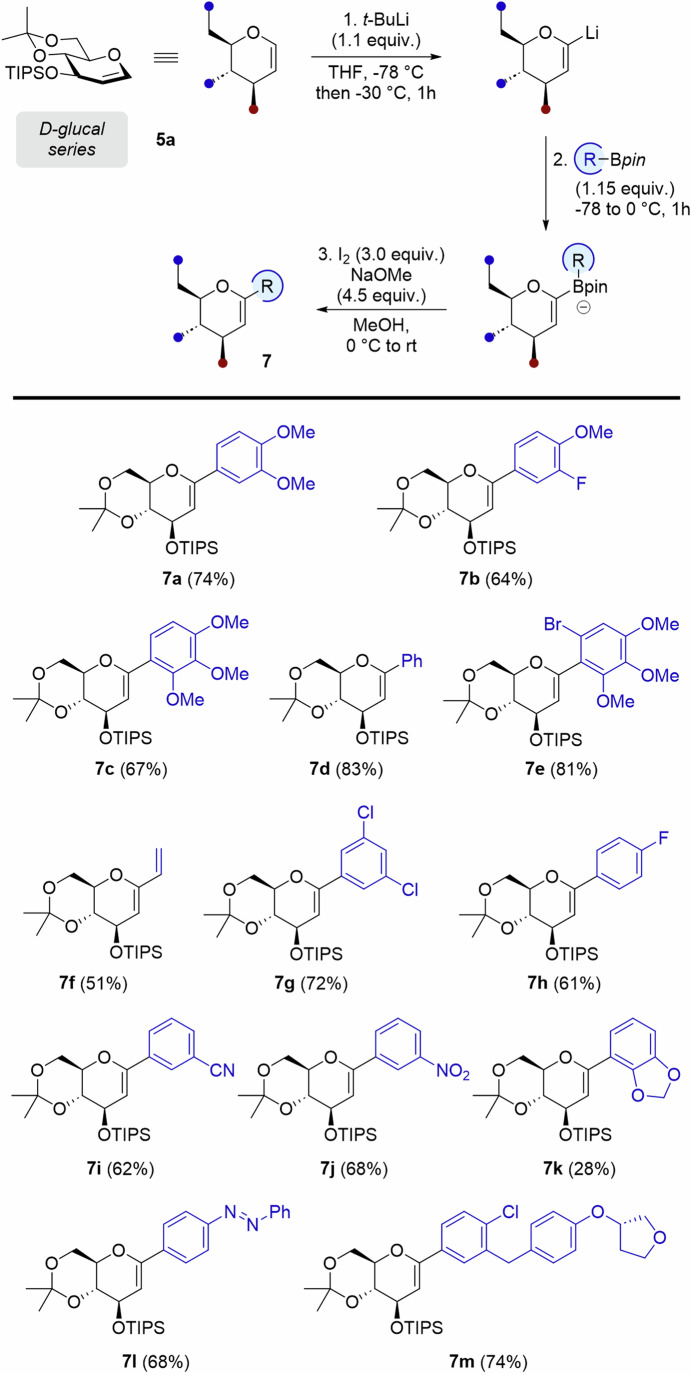
Zweifel olefination’s scope on D-Glucal substrates. The scope of the reaction with D-glucal was evaluated through the introduction of aryl and heteroaryl moieties, displaying both electron-donating and -withdrawing functional groups.

With optimized conditions in hands, we further evaluated the scope of the transformation on protected D-glucal **5a** (Fig. 3). Neutral phenylboronic pinacol ester gave **7** **d** in 83%, and electron-poor aryl boronic esters gave similarly high yields than electron-rich ones (**7b-c,** **7e** and **7g**-**j**, 61-81%). Drops in yields were however observed with acetal-protected reagent (**7k**, 28%), which could be attributed to sensitivity issues during the purification process, as we ruled out the question of sterical hindrance with examples **7c** and **7e** (67 and 81%, respectively). Interestingly, the addition of vinylboronic ester enabled the formation of diene **7** **f** in 51%. The procedure proved quite robust for the introduction of a diazobenzene moiety at glycosidic position (**7** **l**, 68%). The aryl substituent typically found in Empagliflozin was also introduced via its corresponding boronic ester to provide **7** **m** - a dehydrated Empagliflozin precursor - in 74%.

**Fig. 4:**
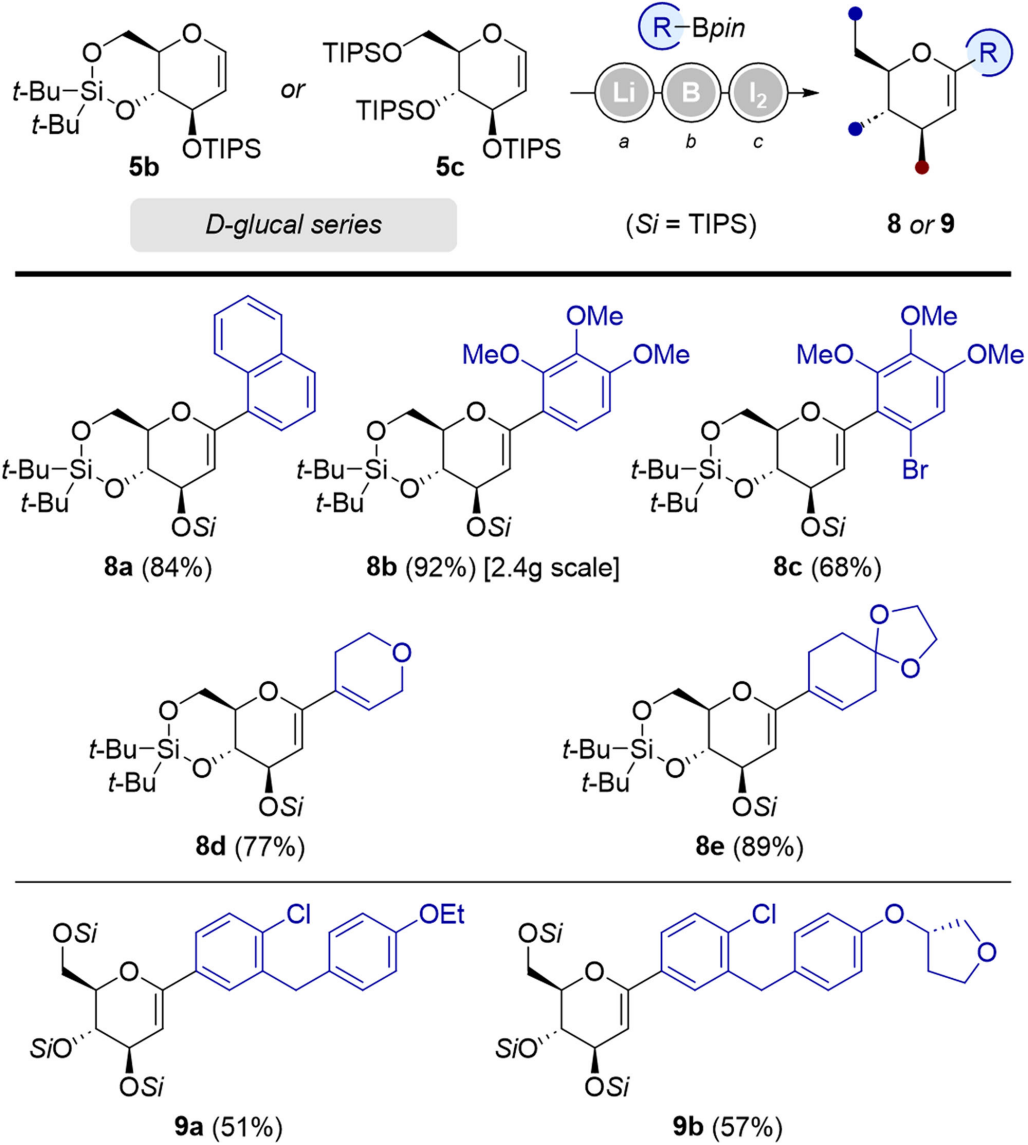
Variation of protecting groups on D-Glucal substrates. ^*a*^*t*-BuLi (1.3 equiv.), THF, −78 to −30 °C, 30 min. ^*b*^RBpin (1.15 equiv.), −78 to 0 °C, 1 h. ^*c*^I_2_ (3.0 equiv.), NaOMe (4.5 equiv.), MeOH, 0 °C to rt.

Other protecting groups on D-glucal substrates were successfully applied to the Zweifel olefination (Fig. 4). Arylated glucals **8a** and **8b** were obtained in good yields from silylacetal **5b**. The use of alkenylboronic esters as partners in the Zweifel olefination provided embedded diene systems **8** **d** and **8e** in 77 and 89% yield, respectively. The reaction proved less efficient when employing tris-TIPS protected D-glucal **5c**, furnishing dehydrated precursors of Dapagliflozin and Empagliflozin **9a** and **9b** in up to 57%.

### Scope of the reaction on other Glycal derivatives

**Fig. 5:**
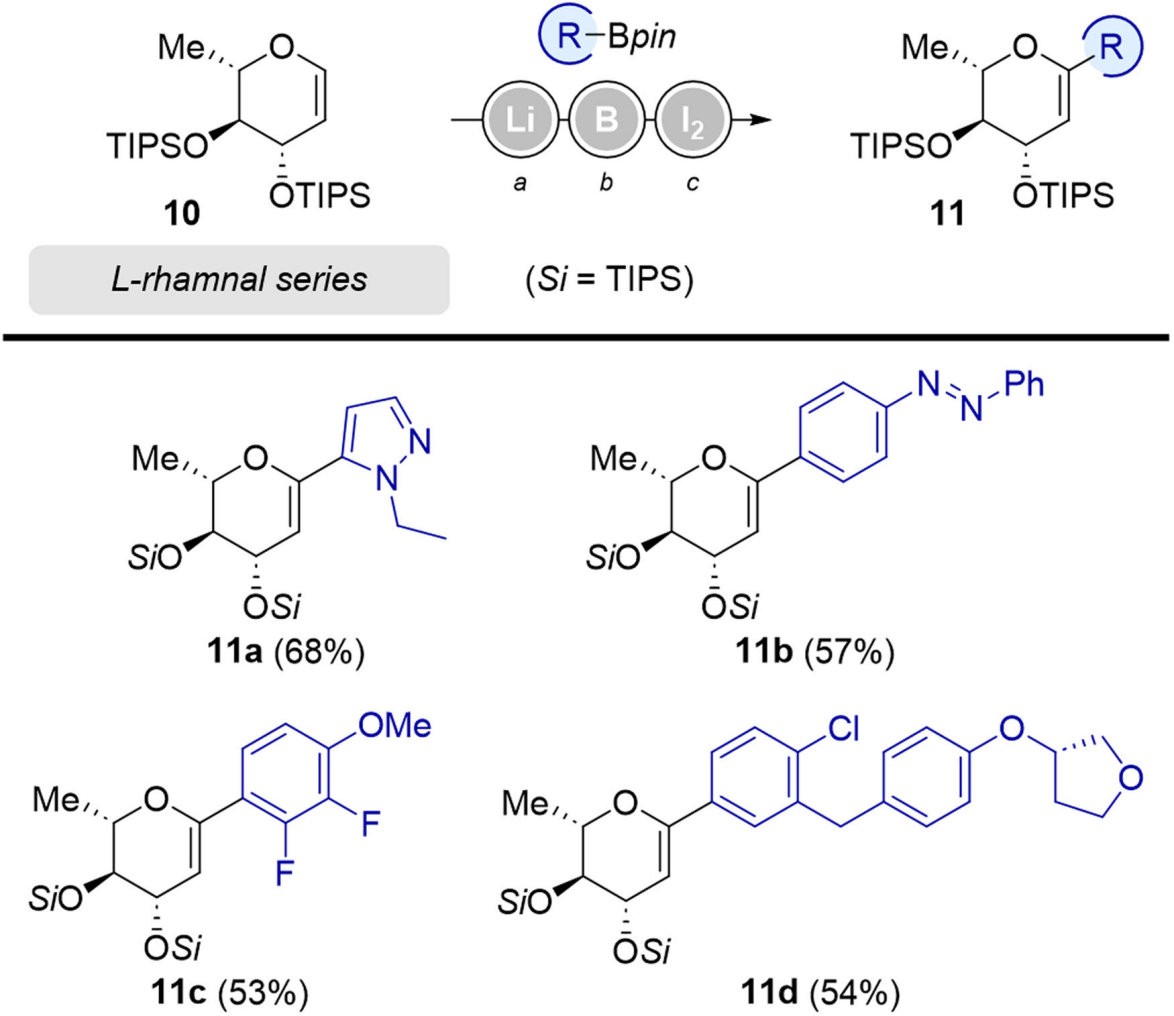
Scope of Zweifel’s olefination on L-Rhamnal substrates. ^*a*^*t*-BuLi (1.3 equiv.), THF, −78 to −30 °C, 30 min. ^*b*^RBpin (1.15 equiv.), −78 to 0 °C, 1 h. ^*c*^I_2_ (3.0 equiv.), NaOMe (4.5 equiv.), MeOH, 0 °C to rt.

The L-rhamnal series (6-desoxy-L-glucal) was examined next (Fig. 5), starting from bis-TIPS protected structure **10**. The same reaction sequence than for the D-glucal series was employed, providing a range of dehydrated *C*-rhamnosides **11**. Electron-rich pyrazol-containing boronic ester gave the heteroaryl derivative **11a** in 68%.

Lower yields were obtained for the introduction of diazobenzene (**11b**) and bisfluorinated anisole (**11c**). The rhamnose-analog precursor of Empagliflozin (**11** **d**) was isolated in 54% yield.

**Fig. 6:**
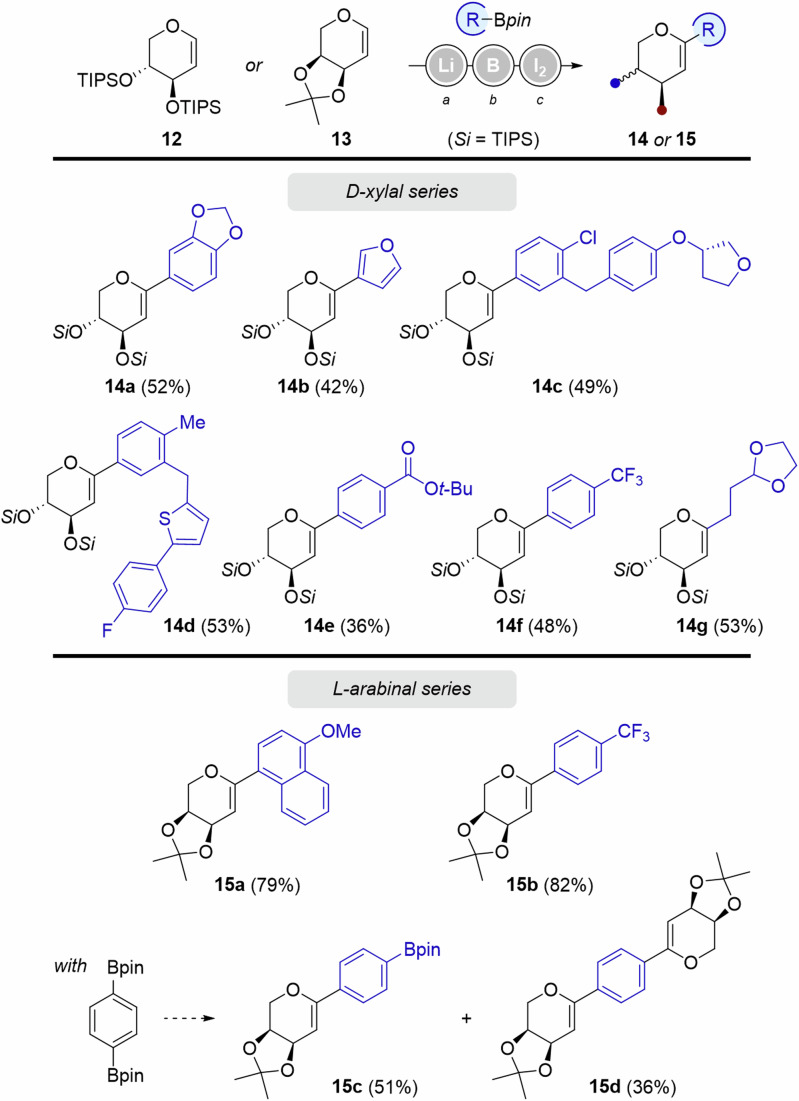
Scope of Zweifel’s olefination on D-Xylal and L-Arabinal substrates. ^*a*^*t*-BuLi (1.1 equiv.), THF, −78 to −30 °C, 30 min. ^*b*^RBpin (1.15 equiv.), −78 to 0 °C, 1 h for the D-Xylal series, 30 min for the L-arabinal series. ^*c*^I_2_ (3.0 equiv.), NaOMe (4.5 equiv.), MeOH, 0 °C to rt.

The variation of the carbohydrate core drove us to challenge unsaturated pentopyranoses **12** and **13** (D-xylal and L-arabinal series, respectively). Applying the optimized conditions described above provided dehydrated *C*-xylosides **14** and *C*-arabinosides **15** (Fig. 6). Yields in the D-xylal series (from *trans*-bis-TIPS protected D-xylal **12**) were generally lower than the ones obtained in the D-glucal and L-rhamnal series, independently from the nature of the boronic ester used in the transformation. Both electron-donating and electron-withdrawing partners provided olefination products **14a**-**f** with moderate yields (36–53%). The nature of the boronic ester was also evaluated via the introduction of alkyl substituents. Product **14** **g** was isolated in 53%.

Despite lower yields in the D-xylal series, it is worth noting that two new D-xylal precursors of Empagliflozin (**14c**, 49%) and Canagliflozin (**14** **d**, 53%) were obtained.

*Cis*-acetal-protected L-arabinal substrates led to the corresponding arylated L-arabinal series in similarly high yields up to 82% (**15a**-**b**), whatever the electronic nature of the boronic ester partner. Interestingly, when a bis-boronic ester was engaged in Zweifel olefination, both products of mono- and bis-olefination (**15c** and **15** **d**) were isolated, in 51 and 36% (overall yield of 87%).

### Further applications

**Fig. 7:**
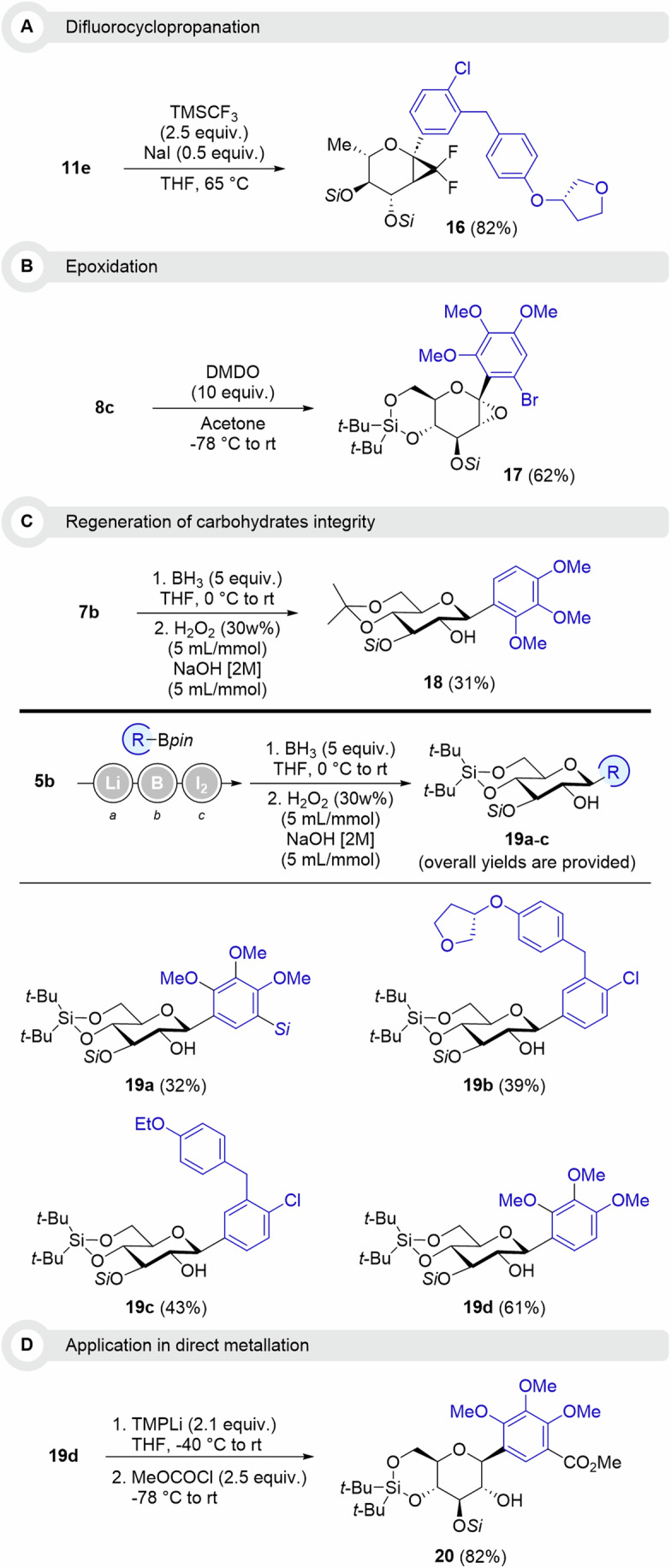
Further applications of glycal-based C-glycosides. Glycal derivatives obtained via Zweifel olefinations were engaged in further selective transformations: **A** Difluorocyclopropanation; **B** Epoxidation; **C** Regioselective Brown oxidation towards the regeneration of the carbohydrate integrity; **D** Directed metalation.

To demonstrate the applicability of this procedure, selected examples were engaged in further useful transformations. Given the paramount significance of fluorinated substituents as well as cyclopropyl-rings in drug discovery, gem-difluorocyclopropanes are anticipated to offer sophisticated frameworks for enhancing therapeutic attributes^31^. Therefore, L-rhamnal derivative **11e** was subjected to difluorocyclopropanation conditions recently described, providing stereoselectively the bicyclic compound **16** in 82% (Fig. 7A). The diastereoselectivity of electrophilic additions onto the double bond of glycal derivatives was demonstrated in previous reports and seemed to be dependent on the orientation of the silyl-ether substituent at position 3. Epoxidation of D-glucal **8c** was performed in the presence of DMDO, furnishing stereoselectively D-oxyglucal compound **17** in 62% (Fig. 7B). The regeneration of the carbohydrate entity was demonstrated on D-glucal **7a** through a first step of hydroboration followed by Brown’s oxidation (Fig. 7C). Protected C-glucoside **18** was obtained stereoselectively (anti-Markovnikov product) in moderate yield (31% over two steps). With a clean and straightforward Zweifel olefination procedure, we showed that the application of Brown’s oxidation did not necessitate purification of intermediates to yield C-glucosides **19a**-**c** in 32 to 43% (over 3 steps). This method offers a straightforward and highly stereoselective route towards protected Empagliflozin **19b** and Dapagliflozin **19c**. **19** **d** was also accessed in 61% from **5b** (Fig. 7C) and used in further metalation chemistry using TMPLi (Fig. 7D)^32^. This strategy proceeds regioselectively ortho to one of the methoxy groups on the aryl scaffold, allowing the introduction of an ester upon addition of methylchloroformate as electrophile. **20** was isolated in 82% as a single regioisomer.

## Conclusions

The simplicity of metal olefination was applied to the synthesis of *C*-glycosides, providing an efficient and selective alternative to oftentimes fastidious carbohydrate chemistry. A single-pot sequence based on a transition-metal free 1,2-boronate rearrangement allowed us to access functionalized D-glucals, L-rhamnals, D-xylals and L-arabinals in moderate to high yields, compounds that were further engaged in the regeneration and sophistication of sugar-based drug-like molecules and natural scaffolds. Given the importance of *C*-glycosides such as gliflozins in drug discovery, the efficiency and simplicity of this strategy could easily be applied in high-throughput screening methodology.

## Methods

Optimization of reaction conditions

See Supplementary Methods, section 2 p.4-8.

Synthetic procedures

See Supplementary Methods, section 4 p.10-14.

Analytical data

See Supplementary Methods, section 4 p.15-84.

NMR spectra

See Supplementary Data 1.

## Supplementary information

Supplementary Material

Description of Additional Supplementary Files

Supplementary Data 1

## Data Availability

Data for this manuscript has been deposited in figshare: 10.6084/m9.figshare.27233724.v1. “Supplementary Methods” contains detailed protocols for the preparation of substrates, reaction optimizations, scope evaluation and description of analytical data (^1^H and ^13^C NMR, HRMS). “Supplementary Data 1” contains all ^1^H and ^13^C NMR spectra.

## References

[1] Zhang, Y. & Wang, F. Carbohydrate drugs: current status and development prospect. *Drug Discoveries Therapeutics* **9**, 79–87 (2015). 25994058 10.5582/ddt.2015.01028

[2] Kilcoyne, M. & Joshi, L. Carbohydrates in Therapeutics. *Cardiovasc. Hematol. Agents Med. Chem.* **5**, 186–197 (2007). 17630944 10.2174/187152507781058663

[3] Osborn, H. M. I., Evans, P. G., Gemmell, N. & Osborne, S. D. Carbohydrate-based therapeutics. *J. Pharm. Pharmacol.* **56**, 691–702 (2004). 15231033 10.1211/0022357023619

[4] Dube, D. H. & Bertozzi, C. R. Glycans in cancer and inflammation-potential for therapeutics and diagnostics. *Nat. Rev.* **4**, 477–488 (2005). 10.1038/nrd1751 15931257

[5] Mahajan, R., Dixit, S., Khare, N. K. & Khare, A. Synthesis of Neoglycoproteins as Artificial Antigens. *J. Carb. Chem.* **13**, 63–73 (1994).

[6] Ohtsubo, K. & Marth, J. D. Glycosylation in cellular mechanisms of health and disease. *Cell* **126**, 855–867 (2006). 16959566 10.1016/j.cell.2006.08.019

[7] Zhang, Y., Jiao, J., Liu, C., Wu, X. & Zhang, Y. Isolation and purification of four flavone C-glycosides from antioxidant of bamboo leaves by macroporous resin column chromatography and preparative high-performance liquid chromatography. *Food Chem.* **107**, 1326 (2008).

[8] Dhalwal, K., Shinde, V. M., Biradar, Y. S. & Mahadik, K. R. Simultaneous quantification of bergenin, catechin, and gallic acid from Bergenia ciliata and Bergenia ligulata by using thin-layer chromatography. *J. Food Comp. Anal.* **21**, 496 (2008).

[9] Minakata, S. et al. Protein C-Mannosylation and C-Mannosyl Tryptophan in Chemical Biology and Medicine. *Molecules* **26**, 5258 (2021). 34500691 10.3390/molecules26175258 PMC8433626

[10] Usman, M. S. et al. Sodium-glucose co-transporter 2 inhibitors and cardiovascular outcomes: A systematic review and meta-analysis. *Eur. J. Prev. Cardiol.* **25**, 495–502 (2018). 29372664 10.1177/2047487318755531

[11] Braunwald, E. Gliflozins in the Management of Cardiovascular Disease. *N. Engl. J. Med.,* **386**, 2024–2034 (2022). 35613023 10.1056/NEJMra2115011

[12] Yang, Y. & Yu, B. Recent Advances in the Chemical Synthesis of C-Glycosides. *Chem. Rev.* **117**, 12281–12356 (2017). 28915018 10.1021/acs.chemrev.7b00234

[13] Kitamura, K., Ando, Y., Matsumoto, T. & Suzuki, K. Total Synthesis of Aryl C-Glycoside Natural Products: Strategies and Tactics. *Chem. Rev.* **118**, 1495–1598 (2018). 29281269 10.1021/acs.chemrev.7b00380

[14] Chen, A. et al. Palladium-catalyzed Suzuki-Miyaura cross-couplings of stable glycal boronates for robust synthesis of C-1 glycals. *Nat. Commun.* **15**, 5228 (2024). 38898022 10.1038/s41467-024-49547-9 PMC11187158

[15] Ghosh, T. & Nokami, T. Recent development of stereoselective C-glycosylation via generation of glycosyl radical. *Carb. Res.* **522**, 108677 (2022). 10.1016/j.carres.2022.108677 36193593

[16] Brown, H. C. & Zweifel, G. A Stereospecific Cis-Hydration of the Double Bond in Cyclic Derivatives. *J. Am. Chem. Soc.* **81**, 247 (1959).

[17] Brown, H. C. & Rao, B. C. Communications - Hydroboration of Olefins. A Remakably Fast Room-Temperature Addition of Diborane to Olefins. *J. Org. Chem.* **22**, 1137 (1957).

[18] Zweifel, G., Arzoumanian, H. & Whitney, C. C. A convenient stereoselective synthesis of substituted alkenes via hydroboration-iodination of alkynes. *J. Am. Chem. Soc.* **89**, 3652–3653 (1967).

[19] Zweifel, G., Polston, N. L. & Whitney, C. C. A stereoselective synthesis of conjugated dienes from alkynes via the hydroboration-iodination reaction. *J. Am. Chem. Soc.* **90**, 6243–6245 (1968).

[20] Armstrong, R. J. & Aggarwal, V. K. 50 Years of Zweifel Olefination: A Transition-Metal-Free Coupling. *Synthesis* **49**, 3323–3336 (2017).

[21] Armstrong, R. J., Niwetmarin, W. & Aggarwal, V. K. Synthesis of Functionalized Alkenes by a Transition-Metal-Free Zweifel Coupling. *Org. Lett.* **19**, 2762–2765 (2017). 28453280 10.1021/acs.orglett.7b01124

[22] Blair, D. J., Fletcher, C. J., Wheelhouse, K. M. P. & Aggarwal, V. K. Stereocontrolled Synthesis of Adjacent Acyclic Quaternary-Tertiary Motifs: Application to a Concise Total Synthesis of (−)-Filiformin. *Angew. Chem. Int. Ed.* **53**, 5552–5555 (2014). 10.1002/anie.201400944 24757079

[23] Music, A. et al. Single-Pot Access to Bisorganoborinates: Applications in Zweifel Olefination. *Org. Lett.* **21**, 2189–2193 (2019). 30864807 10.1021/acs.orglett.9b00493

[24] Music, A. & Didier, D. Organocerium: A New Contender for Halogen–Metal Exchanges. *Synlett*. **30**, 1843–1849 (2019).

[25] Music, A., Hoarau, C., Hilgert, N., Zischka, F. & Didier, D. Catalyst-Free Enantiospecific Olefination with In Situ Generated Organocerium Species. *Angew. Chem. Int. Ed.* **58**, 1188–1192 (2019). 10.1002/anie.201810327 30468288

[26] Music, A., Nuber, C. M., Lemke, Y., Spieß, P. & Didier, D. Electro-alkynylation: Intramolecular Rearrangement of Trialkynylorganoborates for Chemoselective C(sp2)–C(sp) Bond Formation. *Org. Lett.* **23**, 4179–4184 (2021). 34004116 10.1021/acs.orglett.1c01126

[27] Matz, F., Music, A., Didier, D. & Jagau, T.-C. Computational insights into electrochemical cross-coupling of quaternary borate salt. *Electrochem. Sci. Adv.* **2**, e2100032 (2021).

[28] Music, A. et al. Photocatalyzed Transition-Metal-Free Oxidative Cross-Coupling Reactions of Tetraorganoborates. *Chem. Eur. J.* **27**, 4322–4326 (2021). 33306228 10.1002/chem.202005282 PMC7986674

[29] Baumann, A. N. et al. Electro-Olefination—A Catalyst Free Stereoconvergent Strategy for the Functionalization of Alkenes. *Chem. Eur. J.* **26**, 8382–8387 (2020). 32203624 10.1002/chem.202001394 PMC7383514

[30] Music, A. et al. Electrochemical Synthesis of Biaryls via Oxidative Intramolecular Coupling of Tetra(hetero)arylborates. *J. Am. Chem. Soc.* **142**, 4341–4348 (2020). 32040918 10.1021/jacs.9b12300

[31] Bychek, R. M., Levterov, V. V., Sadkova, I. V., Tolmachev, A. A. & Mykhailiuk, P. K. Synthesis of Functionalized Difluorocyclopropanes: Unique Building Blocks for Drug Discovery. *Chem. Eur. J.* **24**, 12291–12297 (2018). 29419903 10.1002/chem.201705708

[32] Mack, K. A. & Collum, D. B. Case for Lithium Tetramethylpiperidide-Mediated Ortholithiations: Reactivity and Mechanisms. *J. Am. Chem. Soc.* **140**, 4877–4883 (2018). 29589920 10.1021/jacs.8b00590 PMC6141241

