# Supplementary material for: Zweifel olefination for C-glycosylation
Source: Commun Chem. 2024 Dec 21;7:306. doi: 10.1038/s42004-024-01339-4 (PMC11663222; doi:10.1038/s42004-024-01339-4)
Supplement: Supplementary file 1 — Supplementary Material [file 42004_2024_1339_MOESM1_ESM.pdf]

Zweifel Olefination for *C*-Glycosylation

Florain Trauner, [a] Bilel Boutet,[a] Fabian Pilz,[b] Verena Weber [c] and Dorian Didier*[a]

a. Technische Universität Darmstadt, Clemens-Schöpf-Institut für Organische Chemie und Biochemie, Peter-Grünberg-Straße 4, 64287 Darmstadt.
b. Ludwig-Maximillians Universität, Department Chemie, Butenandtstraße 5, 81377 Munich.
c. Institute for Neuroscience and Medicine and Institute for Advanced Simulations (INM-9/IAS-5), Computational Biomedicine, Forschungszentrum Jülich, 52425 Jülich.

Contents

1. GENERAL CONSIDERATIONS 2
   1.1 Investigated Glycals 3
2. OPTIMIZATION 4
   2.1 Initial Optimization: 4
   2.2 Optimization of Lithiation 5
   2.3 Deuterolysis Experiments 6
3. OBSERVATIONS 9
4. GENERAL PROCEDURES 10
5. REFERENCES 85

Supplementary Methods

1. General Considerations

All reactions were carried out under dry N_2 / Ar atmosphere in flame-dried glassware unless otherwise stated. Syringes, which were used to transfer anhydrous solvents or reagents, were purged with nitrogen or argon three times prior to use. THF (stabilized) was purchased in 99.5 % purity from Acros Organics. Organolithiums (*n*BuLi, *s*BuLi, *t*BuLi,) were purchased from Rockwood Lithium and the concentration was determined by titration against *i*PrOH using 1,10-phenantroline as indicator. Gringard reagents were prepared in THF, the used magnesium was activated by addition of 1,2-dibromoethane and subsequent heating to reflux. Titration of Gringard reagents was performed with benzoic acid and 4-phenylazodiphenylamin as indicator. Chromatographic purifications were performed using silica gel (SiO_2, 0.040-0.063 mm, 230- 400 mesh ASTM) from Merck or Alumina (Al_2O_3, 32-63 µm) from MP EcoChrom™. The spots were visualized under UV (254 nm) or by staining the TLC plate with either $KMnO_4$ solution (K_2CO_3, 10 g – $KMnO_4$, 1.5 g – H_2O, 150 mL – NaOH 10% in H_2O, 1.25 mL) or Curcumin solution (Curcumin, 0.4 g – EtOH, 400 mL – 2 M HCl, 20 mL). Yields refer to isolated yields of compounds estimated to be >95% pure as determined by ^{1}H-NMR and GC-analysis. The ^{13}C and ^{1}H-NMR spectra were recorded on VARIAN Mercury 200, BRUKER ARX 300, VARIAN VXR 400 S and BRUKER AMX 600 instruments. Chemical shifts are reported as δ values in ppm relative to the residual solvent peak (^{1}H-NMR, ^{13}C-NMR) in deuterated chloroform ($CDCl_3$: δ 7.26 ppm for ^{1}H-NMR and δ 77.16 ppm for ^{13}C-NMR). Abbreviations for signal coupling are as follows: s (singlet), d (doublet), t (triplet), q (quartet), quint (quintet), m (multiplet) and br (broad). Reaction endpoints were determined by GC monitoring of the reactions with *n*dodecane as an internal standard. Gas chromatography was performed with machines of Agilent Technologies 7890, using a column of type HP 5 (Agilent 5% phenylmethylpolysiloxane; length: 15 m; diameter: 0.25 mm; film thickness: 0.25 µm) or Hewlett-Packard 6890 or 5890 series II, using a column of type HP 5 (Hewlett-Packard, 5% phenylmethylpolysiloxane; length: 15 m; diameter: 0.25 mm; film thickness: 0.25 µm). High resolution mass spectra (HRMS)

and low-resolution mass spectra (LRMS) were recorded on Finnigan MAT 95Q, Finnigan MAT 90 instrument or JEOL JMS-700. Infrared spectra were recorded on a Perkin 281 IR spectrometer and samples were measured neat (ATR, Smiths Detection DuraSample IR II Diamond ATR). The absorption bands were reported in wave numbers (cm^{-1}) and abbreviations for intensity are as follows: vs (very strong; maximum intensity), s (strong; above 75% of max. intensity), m (medium; from 50% to 75% of max. intensity), w (weak; below 50% of max. intensity) and br (broad). Melting points were determined on a Büchi B-540 apparatus and are uncorrected

1.1 Investigated Glycals

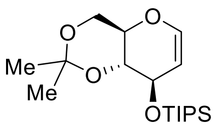

5a

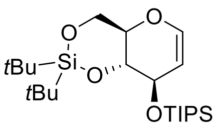

5b

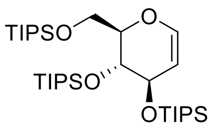

5c

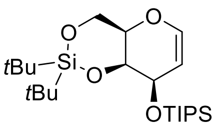

5d

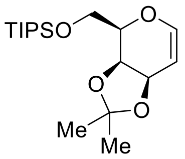

5e

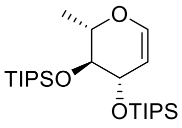

10

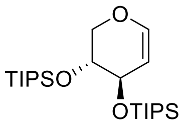

11

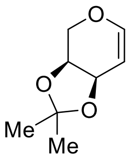

12

2. Optimization

2.1 Initial Optimization:

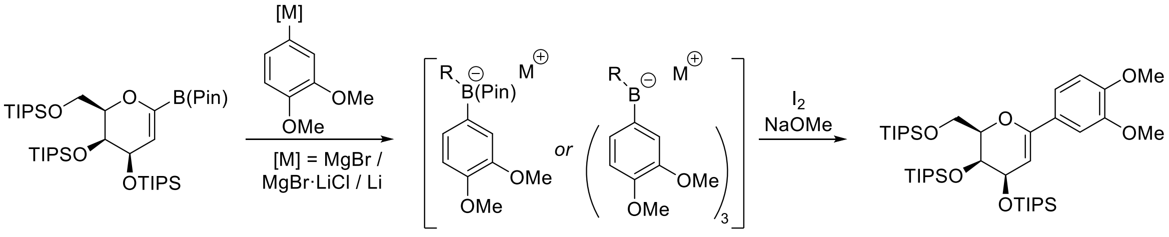

Entry[a]	Organometallic species [M]	Equiv.	Yield [%]
1[b]	MgBr	1.0	-
2[b]	MgBr	2.0	-
3[c]	MgBr · LiCl	2.0	-
4[d]	Li	1.0	Trace
5[d]	Li	1.5	38

[a] Experiments were performed according to GP-B. [b] According to GP-B, but as the first step Aryl-MgBr (0.84M in THF) was added to a solution of the respective glucal at -78 °C. [c] Aryl-MgBr · LiCl was generated from 4-bromo-1,2-dimethoxybenze (2 equiv.), Mg (3.2 equiv.) and LiCl (2.2 equiv.). [d] Aryllithium was generated from 4-bromo-1,2-dimethoxybenze (1 equiv. or 1.5 equiv., respectively) and *n*BuLi (1.1 equiv. or 1.6 equiv., respectively).

- No desired product could be detected via ^{11}B-NMR for entries 1 – 3 using Grignard and turbo Grignard reagents respectively.
- Using 1 equiv. of aryllithium species, traces of the desired zweifel coupling could be detected, 1.5 equiv. furnished the desired compound in 38 %.
- Due to the moderate reaction outcome (38%, entry 5) the "inverse" pathway (metalation of the respective glycal, followed by treatment with boronic ester) was chosen for further investigations.

2.2 Optimization of Lithiation – Temperature dependence

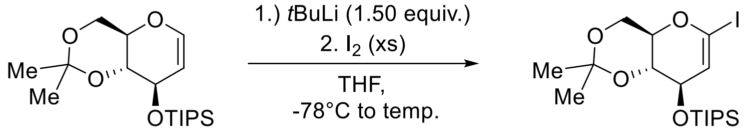

5a **6**

Entry [a]	Temperature [°C] [b]	Rct. time [min]	Yield [%] [c]
1	-78	15	0
2	-60	15	0
3	-50	15	51
4	-40	15	55
5	-40	30	65
6	-30	15	70
7	-30	30	73
8	-20	15	71
9	-10	15	60
10	0	15	40
11	0	45	0

[a] The reaction was carried out on a 0.30 mmol scale in THF (2.0 mL) with *t*BuLi (1.50 equiv.) and undecane (1.00 equiv.). Addition of *t*BuLi at -78°C followed by stirring for 10 min at this temperature and warming to the indicated temperature for the indicated time. Subsequent quench of an aliquot of the mixture with iodine (xs) afforded the substituted glycal. [b] Temperatures were adjusted using a dry ice/acetone bath. [c] The yields of these reactions were determined by GC using undecane as internal standard.

Entry	Temperature [°C] [b]	Rct. Time [min]	Yield [%] [c]
1	-30	30	69
2	-30	60	80
3	-30	90	63

[a] The reaction was carried out on a 0.20 mmol scale in THF (1.0 mL) with undecane (1.00 equiv.) as internal standard. Subsequent quench of an aliquot with iodine (xs). [b] Temperatures were adjusted using a dry ice/acetone bath. [c] The yields of the reactions were determined by GC using undecane as internal standard.

2.3 Deuterolysis Experiments

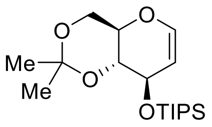

5a

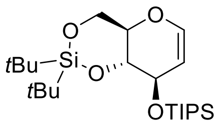

5b

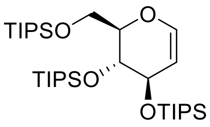

5c

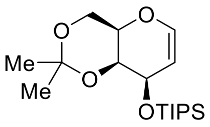

5d

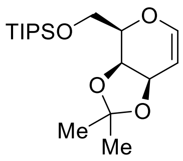

5e

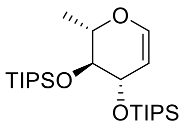

10

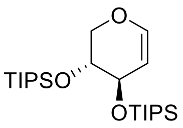

11

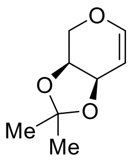

12

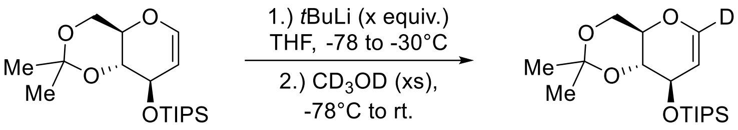

5a **6**

Entry	Glycal	Equiv. of *t*BuLi	Time [min]	D incorporation at C1 [%][a]
1	**5a**	1.1	15	89
2	**5a**	1.1	30	92
3	**5a**	1.1	45	92
4	**5a**	1.1	60	98
5	**5a**	1.3	15	97
6	**5a**	1.3	30	99
7	**5a**	1.3	85	99
8	**5b**	1.1	60	100
9	**5b**	1.3	30	100
10	**5c**	1.1	60	85
11	**5c**	1.3	30	79
12	**5d**	1.1	60	-
13[b]	**5d**	1.3	30	-
14	**5e**	1.1	60	-
15	**5e**	1.3	30	-
16	**10**	1.1	60	83
17	**10**	1.3	30	88
18	**11**	1.3	30	77
19	**11**	1.3	60	84
20	**12**	1.1	30	92
21	**12**	1.3	30	96

[a] Experiments were performed according to general procedure **A**. Equivalents of *t*BuLi and reaction time at -30°C were adjusted as listed in the table. [b] Reaction was performed according to general procedure **A**, but on a 0.10 mmol scale.

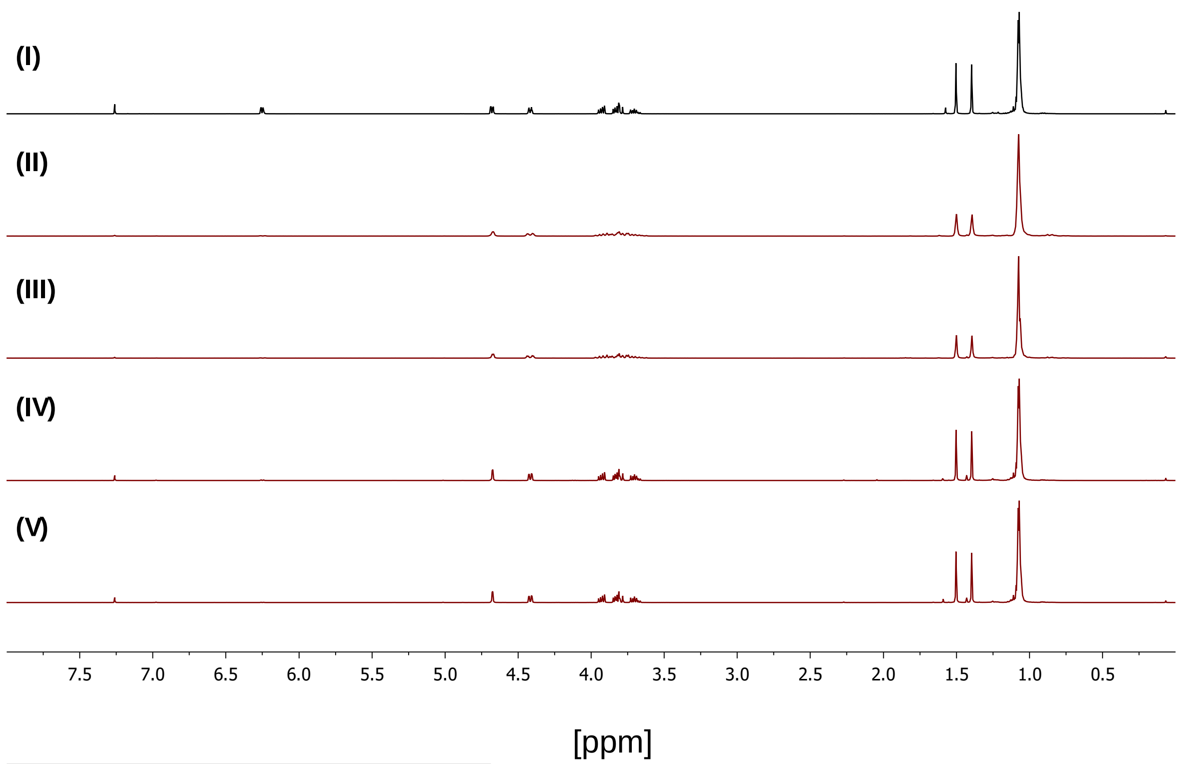

^{1}H-NMR spectra of (**I**) 1 and entries (**II**) 1, (**III**) 2, (**IV**) 3 and (**V**) 4.

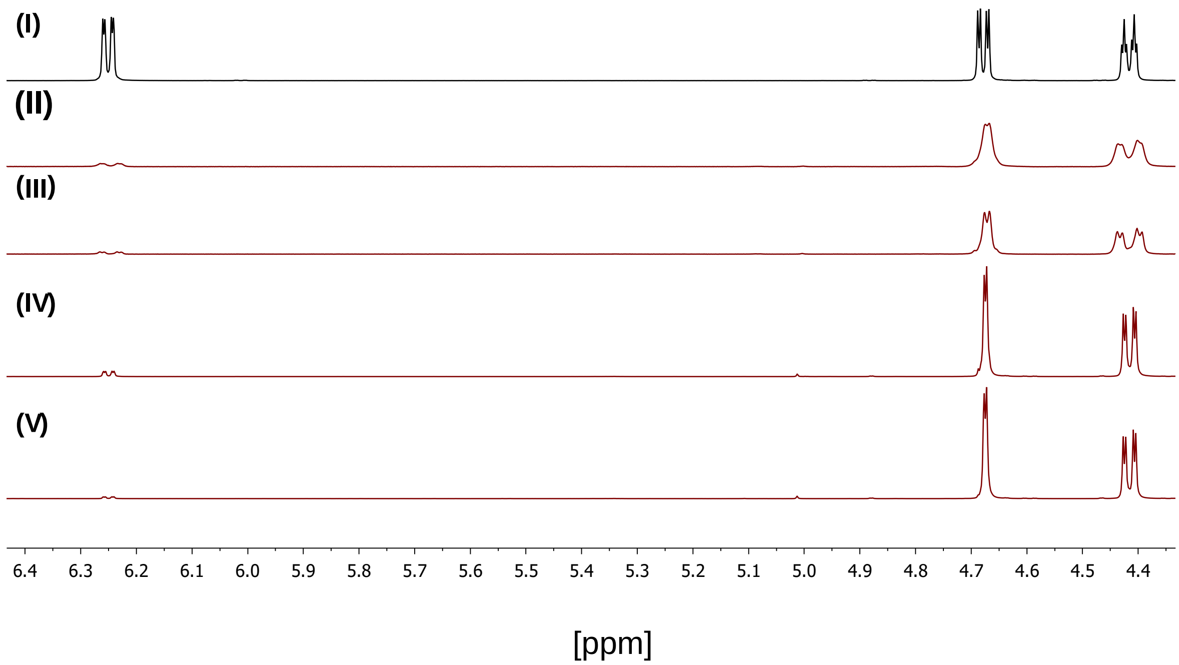

Zoom in on the ^{1}H-NMR spectra of (**I**) 1 and entries (**II**) 1, (**III**) 2, (**IV**) 3 and (**V**) 4.

3. Observations

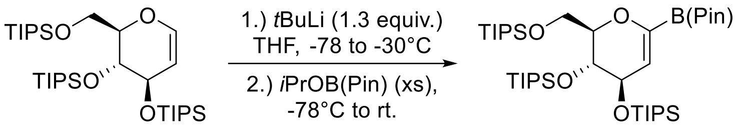

- Can be obtained with reasonable purity (90 %)
- Chromatographic separation from byproducts was not possible (Silica-Gel, Alumina and Florisil)
- B(Epin) derivative could also not be further purified.

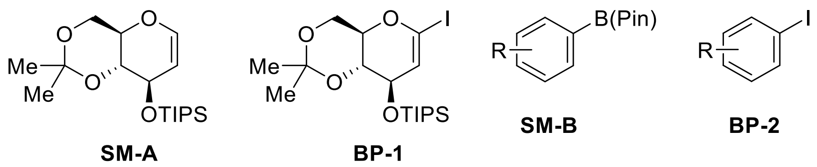

- Possible byproducts of the Zweifel-protocol include iodinated glycal (**BP-1**) and the iodo-(hetero)aryl derived by the respective boronic ester (**BP-2**).
- If after the Zweifel-protocol residual boronic acid pinacol ester is detected (TLC), a 4.2 M aq. solution of KHF_2 is added to the reaction mixture in MeOH. After 30 min of stirring at ambient temperature, the solvent is removed *in vacuo* and the residue is washed and decanted with Et_2O (3×20 mL). This facilitates chromatographic separation and avoids streaking of the residual boronic ester.
- Some electron-rich Zweifel-coupling products show rapid degradation at ambient temperature and should be stored at -30°C.
- TIPS protected glycals generally lead to less clean Li-incorporation.

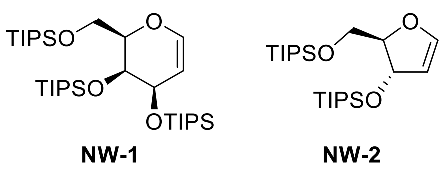

- Metallation and deuterolysis of **NW-1** (TIPS-galactal) and **NW-2** (TIPS-ribal) resulted in unsatisfactory D-incorporation, and required up to 3 equiv. of *t*BuLi, which resulted in major side product formation during the Zweifel-protocol.

4. General Procedures

General procedure A: Lithiation/Deuterization of Glycals

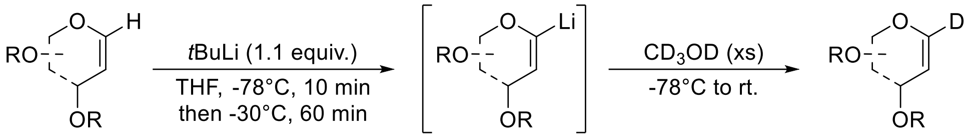

(5a-5e, 10-12)

A solution of the appropriate glycal (0.2 mmol, 1.0 equiv.) in THF (1.0 mL) at -78°C was treated dropwise with *t*BuLi (1.1 equiv.) and the solution stirred for 10 min, then stirred at -30°C for 60 min. The reaction mixture was cooled to -78°C, quenched with excess CD_3OD and allowed to warm to ambient temperature. After stirring at this temperature for approximately 30 min, the solution was filtered (Et_2O with a drop of methanol) through a glass pipette with $MgSO_4$ and concentrated. The crude deuterated reaction products were filtered (*i*Hexane/Et_2O) through a plug of silica gel and the solvents were removed *in vacuo*. ^{1}H-NMR integration allowed to determine the D-incorporation ratio.

General procedure B: Coupling of Glycals with Boronic acid pinacol esters

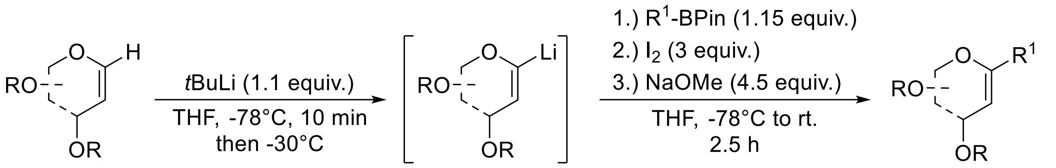

(5a-5e, 10-12)

(7a-7m, 8a-8e, 9a,9b, 11a-11d, 14a-14f, 15a-15d, 19a-19d)

A stirred solution of the respective protected glycal (0.2 mmol, 1.0 equiv.) in THF (1.0 mL) was cooled to -78°C and *t*BuLi (0.22 mmol, 1.10 equiv. or 0.26 mmol, 1.3 equiv.) was added dropwise. The resulting solution stirred at -78°C for 10 min and then warmed to -30°C and stirred for further 60 min (or as indicated) at this temperature. After this time, the solution was cooled back to -78°C and a solution of the respective boronic acid pinacol ester (0.23 mmol, 1.15 equiv.) in THF (0.5 mL) was added dropwise. The reaction mixture was allowed to stir at -78°C for 15 min and then warmed to 0°C and stirred for a further 45 min. After this time, the solution was cooled to -78°C and a solution of iodine (152 mg, 0.60 mmol, 3.00 equiv.) in THF (0.5 mL) was added dropwise over 5 min. The resulting dark red reaction mixture was stirred for 15 min at the aforementioned temperature, then warmed to 0°C and stirred for 30 min. After this time, a solution of sodium methoxide (0.5 M in MeOH, 1.80 mL, 0.90 mmol, 4.50 equiv.) was added dropwise. The resulting mixture stirred at 0°C for 30 min and was then allowed to reach ambient temperature. After reaching rt., the reaction is completed. The reaction was then quenched by addition of sat. aq. $Na_2S_2O_3$ (2 mL), followed by water (20 mL) and Et_2O (20 mL). The organic layer was separated and the aqueous layer was extracted twice with Et_2O (2×20 mL). The combined organic extracts were dried over $MgSO_4$, filtered and concentrated *in vacuo*. The crude product was purified *via* column chromatography (SiO_2).

General Procedure C: Preparation of Boronic Acid Pinacol Esters *via* Lithium-Halogen exchange (SI-5 – SI-8)

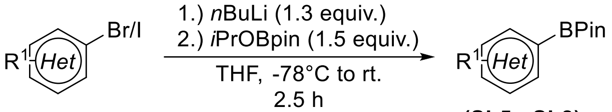

(Si-5 - Si-8)

The desired (hetero)aryl bromide/ iodide (5 mmol, 1.0 equiv.) was charged to a flame-dried flask and dissolved in dry THF (20 mL). *n*BuLi (6.5 mmol, 1.3 equiv.) was added dropwise to the mixture at -78°C and the mixture was stirred at this temperature for 30 min. *i*PrOBPin (7.5 mmol, 1.5 equiv.) was added dropwise to the mixture at -78°C and the solution was allowed to stir for 1 h at this temperature. After this period the reaction mixture was allowed to warm to rt. and stirred at this temperature for a further 60 min. The reaction was quenched by addition of sat. aq. NH_4Cl (5 mL), and water (50 mL) was added. The aq. phase was extracted with EtOAc (3 × 50 mL), the combined org. fractions were washed with Brine (50 mL), and all volatiles were removed *in vacuo*. Purification of the crude mixture *via* Flash column chromatography (SiO_2) furnished the desired hetero(aryl) boronic acid pinacol esters.

Note: Curcumin stain helps visualizing the respective boronic esters on TLC.

General Procedure D: Preparation of Glycals (10, 11)

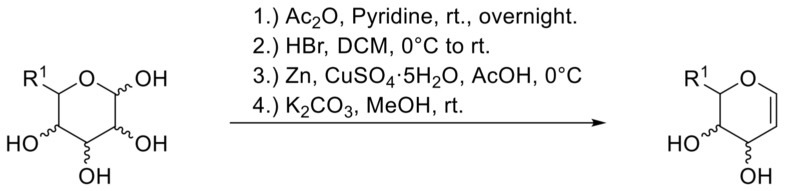

with R^1 = H/ Me

(**10**,**11**)

Peracetylation:

The respective sugar (20 mmol, 1.0 equiv.) was charged to a dry flask under argon atmosphere and dissolved in dry pyridine (50 mL). Acetic anhydride (23 mL, 0.24 mmol, 12 equiv.) was added dropwise to the mixture and the solution was allowed to stir overnight at rt. The next day the reaction mixture was transferred to a separatory funnel and EtOAc (100 mL) and H_2O (100 mL) was added. The organic fraction was separated and washed with sat. $NaHCO_3$ (100 mL) and Brine (50 mL). After drying over anhydr. $MgSO_4$ and concentration *in vacuo* the obtained crude Ac-sugar was directly employed in the next step.

Bromination

The peracetylated sugar was charged to a 250 mL flask and dissolved in DCM (100 mL). After cooling the mixture to 0°C, a solution of HBr in AcOH (33 wt%, 5.0 equiv.) was added dropwise *via* dropping funnel and the mixture was allowed to warm to rt. After TLC control showed full consumption of the starting material, DCM (100 mL) was added and the mixture was transferred to a separatory funnel. The organic phase was separated and washed three times with sat. aq. $NaHCO_3$ (150 mL) and Brine (100 mL). After drying over anhydr. $MgSO_4$ the solvent was removed *in vacuo.*

Elemination

The crude product from the previous step was dissolved in AcOH (100 mL) and $CuSO_4$ pentahydrate (5 mmol, 0.25 equiv.) was added. The reaction mixture was cooled to 0°C and Zn Powder (0.4 mol, 20 equiv.) was added in portions. After full consumption of starting material judged by TLC control, DCM (150 mL) was added and the reaction

mixture was transferred to a separatory funnel. H_2O (100 mL) was added and the organic phase was separated and washed three times with sat. aq. $NaHCO_3$ (100 mL) and Brine (100 mL). Drying over anhydr. $MgSO_4$ and removal of the solvent *in vacuo* afforded the crude Ac-Glycal, which was purified by Flash Colum Chromatography (SiO_2, pentane/ EtOAc).

Deprotection

The respective Ac-Glycal was dissolved in MeOH (50 mL) and K_2CO_3 (0.1 equiv) was added in one portion. After stirring overnight at rt., the reaction mixture was filtered over Celite and the solvent was removed *in vacuo*. The free anhydrosugar was directly used in further synthesis without additional purification.

4,6-*O*-isopropylidene-D-glucal (<u>SI-1</u>)

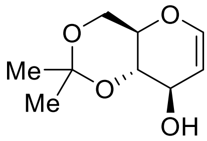

According to a modified literature procedure[1], 2,2-dimethoxypropane (21.2 mL, 171 mmol, 5.00 equiv.) was dissolved in DMF (52 mL) and the solution was acidified to p*H* 3 with *p*-TsOH monohydrate. D-Glucal (5.0 g, 34 mmol, 1.0 equiv.) was added at once and the reaction mixture was stirred at rt. for 45 min, then quenched with sat. $NaHCO_3$ solution and extracted with chloroform (3×150 mL). The combined organic layers were washed with Brine (150 mL) and dried over $MgSO_4$. All volatiles were removed *in vacuo*. Purification by flash column chromatography (SiO_2, *i*H/EtOAc 9:1 to 1:1) afforded the title compound **<u>SI-1</u>** as a colorless oil (2.16 g, 11.6 mmol, 34%).

^{1}H-NMR ($CDCl_3$, 400MHz, 300K) δ = = 6.24 (dd, *J* = 6.2, 1.8 Hz, 1H), 4.68 (dd, *J* = 6.1, 1.9 Hz, 1H), 4.35 – 4.23 (m, 1H), 3.89 (dd, *J* = 10.9, 5.5 Hz, 1H), 3.80 – 3.62 (m, 3H), 1.48 (s, 3H), 1.38 (s, 3H).

^{13}C-NMR ($CDCl_3$, 101MHz, 300K) δ = 144.1, 103.8, 99.9, 73.5, 69.3, 67.2, 61.6, 29.0, 19.1.

LR-MS (70eV): m/z [%] = 186.0 (9). 171.0 (14), 110.0 (34), 97.0 (25), 81.0 (38), 71.0 (68), 59.0 (100).

IR (FT-ATR) $\tilde{\nu}$ [cm^{-1}]: 3437 (w), 2994 (w), 2944 (w), 2893 (w), 1641 (m), 1479 (w), 1462 (w), 1436 (w), 1375 (m), 1301 (w), 1268 (m), 1228 (s), 1198 (s), 1166 (s), 1114 (s), 1088 (vs), 1062 (s), 1032 (s), 1002 (s), 965 (m), 941 (s), 918 (w), 866 (vs), 816 (w), 753 (s), 706 (w), 681 (w), 658 (w).

Spectral characteristics were in agreement with previously reported data.[1]

1,5-Anhydro-4,6-*O*-(isopropylidene)-3-(*O*-triisopropylsilyl)-2-*deoxy*-D*arabino*-hex-1-enitol (5a)

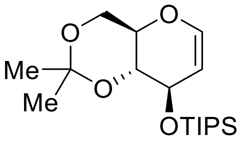

According to a modified literature procedure[2], the alcohol **SI-1** (2.16 g, 11.6 mmol, 1.00 equiv.) was dissolved in DMF (11 mL) and imidazole (1.58 g, 23.2 mmol, 2.00 equiv.) and TIPSCl (3.72 mL, 17.4 mmol, 1.50 equiv.) were added. The reaction mixture was stirred at 80°C overnight. After cooling to ambient temperature, H_2O (100 mL) was added and the aqueous layer was extracted with EtOAc (3×50 mL). The combined organic layers were washed with Brine (50 mL), dried over $MgSO_4$, filtered and concentrated *in vacuo*. The crude product was purified by flash column chromatography (SiO_2, *i*H/EtOAc, 9:1 with 5% NEt_3) to afford **5a** as a colorless oil (3.31 g, 9.64 mmol, 83%).

^{1}H-NMR ($CDCl_3$, 400MHz, 300K) δ = 6.25 (dd, *J* = 6.2, 1.6 Hz, 1H), 4.68 (dd, *J* = 6.2, 1.9 Hz, 1H), 4.42 (ddd, *J* = 7.2, 1.8, 1.8 Hz, 1H), 3.93 (dd, *J* = 11.0, 5.6 Hz, 1H), 3.83 (dd, *J* = 10.3, 7.2 Hz, 1H), 3.81 (dd, *J* = 10.8 Hz, 1H), 3.70 (ddd, *J* = 10.3, 5.5 Hz, 1H), 1.50 (s, 3H), 1.40 (s, 3H), 1.17 – 1.01 (m, 21H).

^{13}C-NMR ($CDCl_3$, 101MHz, 300K) δ = 143.3, 105.9, 99.7, 73.5, 69.8, 67.9, 61.9, 29.1, 19.0, 18.1, 18.0, 12.4.

LR-MS (70eV): m/z [%] = 299.1 (41), 241.0 (39), 213.0 (19), 197.0 (11), 185.0 (45), 169.0 (15), 155.0 (7), 143.0 (9), 131.0 (22), 115.0 (45), 103.0 (41), 87.0 (13), 75.0 (100), 61.0 (60).

IR (FT-ATR) $\tilde{\nu}$ [cm^{-1}]: 2995 (w), 2943 (m), 2894 (w), 2867 (m), 1638 (m), 1464 (w), 1382 (m), 1372 (m), 1268 (m), 1261 (w), 1232 (s), 1218 (m), 1200 (m), 1168 (m), 1119 (s), 1100 (vs), 1076 (s), 1066 (s), 1056 (s), 1010 (s), 998 (m), 974 (w), 943 (m), 922 (m), 874 (vs), 846 (m), 794 (s), 754 (m), 729 (m), 678 (s), 658 (m).

Spectral characteristics were in agreement with previously reported data.[2]

4,6-*O*-Di(*tert*-butyl)silanediyl-D-glucal (SI-2)

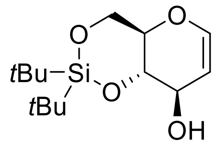

According to a literature procedure[2], D-glucal (754 mg, 5.16 mmol, 1.00 equiv.) was dissolved in DMF (7 mL). Then 2,6-lutidine (1.80 mL, 15.5 mmol, 3.0 equiv.) was added and the solution was cooled to -15 °C using an acetone/ice bath. Di(*tert*-butyl)silyl ditriflate (1.84 mL, 5.68 mmol, 1.1 equiv.) was added dropwise and the mixture was warmed to rt. and stirred for 1.5 h. The reaction mixture was diluted with H_2O (30 mL) and extracted with Et_2O (3×20 mL). The combined organic extracts were washed with Brine (20 mL), dried over $MgSO_4$ and filtered. The solvent was removed *in vacuo* to afford a light-yellow oil. The crude reside was purified *via* flash column chromatography (SiO_2, *i*H/EtOAc, 15:1), which afforded **SI-2** as a colorless solid (956 mg, 3.35 mmol, 65%).

^{1}H-NMR ($CDCl_3$, 400MHz, 300K) δ = 6.26 (dd, *J* = 6.1, 1.9 Hz, 1H), 4.75 (dd, *J* = 6.1, 1.9 Hz, 1H), 4.34 – 4.26 (m, 1H), 4.17 (dd, *J* = 10.2, 4.9 Hz, 1H), 4.01 – 3.88 (m, 2H), 3.83 (td, *J* = 10.2, 4.9 Hz, 1H), 2.50 (d, *J* = 2.9 Hz, 1H), 1.06 (s, 9H).

^{13}C-NMR ($CDCl_3$, 101MHz, 300K) δ = 143.8, 103.1, 77.5, 77.2, 76.8, 72.4, 70.3, 65.8, 27.6, 27.0, 22.9, 20.0.

LR-MS (70eV): m/z [%] = 286.0 (6), 268.1 (1), 229.0 (100), 211.0 (6), 199.0 (15), 187.0 (68), 168.9 (7), 157.0 (65), 143.0 (9), 131.0 (20), 115.0 (48), 103.0 (29), 91.0 (23), 77.0 (85), 57.0 (50), 41.0 (47).

IR (FT-ATR) $\tilde{\nu}$ [cm^{-1}]: 3606 (vw), 3459 (w), 3363 (vw), 2964 (w), 2934 (m), 2890 (m), 2859 (m), 1646 (m), 1470 (m), 1393 (w), 1364 (w), 1313 (vw), 1274 (w), 1233 (m), 1214 (w), 1186 (w), 1157 (m), 1140 (w), 1119 (s), 1093 (s), 1078 (s), 1057 (m), 1029 (m), 1012 (m), 989 (s), 953 (m), 938 (w), 905 (w), 867 (vs), 824 (vs), 780 (m), 763 (vs), 720 (w), 690 (w), 686 (w), 652 (s).

Spectral characteristics were in agreement with previously reported data.[2]

1,5-Anhydro-2-*deoxy*-4,6-*O*-bis(*tert*-butylsilylidene)-3-*O*-triisopropylsilylD-*arabino*-hex-1-enitol (5b)

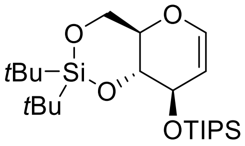

According to a literature procedure[3], to a solution of **SI-2** (936 mg, 3.27 mmol, 1.00 equiv.) in DMF (20 mL) was added imidazole (556 mg, 8.17 mmol, 2.50 equiv.) and TIPSCl (0.90 mL, 4.25 mmol, 1.30 equiv.). The reaction mixture was stirred at 60°C overnight, then cooled to rt. and quenched with H_2O (25 mL) and extracted with Et_2O (3×50 mL). The combined organic fractions were washed with H_2O (5×50 mL) and Brine (50 mL) and then dried over $MgSO_4$. After removal of the solvents *in vacuo*, the crude reside was purified by flash column chromatography (SiO_2, *i*H/EtOAc 19:1) to give **5b** as colorless crystals (1.38 g, 3.11 mmol, 95 %).

^{1}**H-NMR** ($CDCl_3$, 400MHz, 300K) δ = 6.23 (dd, *J* = 6.1, 1.6 Hz, 1H), 4.67 (dd, *J* = 6.1, 1.9 Hz, 1H), 4.42 (dd, *J* = 6.9, 1.9 Hz, 1H), 4.15 (dd, *J* = 10.3, 5.0, 1H), 4.05 – 3.96 (m, 1H), 4.02 – 3.91 (m, 1H), 3.80 (td, *J* = 10.3, 5.0 Hz, 1H), 1.23 – 1.06 (m, 21H), 1.06 (s, 9H), 0.99 (s, 9H).
^{13}**C-NMR** ($CDCl_3$, 101MHz, 300K) δ = 142.9, 105.5, 77.7, 72.9, 70.9, 66.2, 27.6, 27.1, 22.9, 20.0, 18.3, 12.6.
LR-MS (70eV): m/z [%] = 399.2 (2), 369.2 (1), 343.1 (1), 317.1 (7), 261.1 (1), 244.9 (1), 229.1 (1), 206.9 (3), 185.0 (6), 157.0 (3), 134.9 (4), 115.0 (8), 81.0 (100), 57.1 (11).
IR (FT-ATR) $\tilde{\nu}$ [cm^{-1}]: 2959 (m), 2943 (m), 2889 (m), 2861 (m), 1647 (m), 1470 (m), 1391 (w), 1381 (w), 1364 (w), 1282 (w), 1258 (w), 1238 (m), 1215 (w), 1185 (w), 1160 (s), 1142 (m), 1124 (s), 1105 (vs), 1080 (s), 1058 (s), 1012 (m), 998 (s), 968 (m), 917 (w), 875 (vs), 827 (vs), 773 (s), 762 (vs), 734 (s), 678 (s), 652 (vs).

Spectral characteristics were in agreement with previously reported data.[3]

1,5-Anhydro-2-deoxy-3,4,6-tri-*O*-triisopropylsilyl-D-arabino-hex-1-enitol (5c)

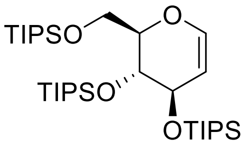

According to a modified literature procedure[4], D-glucal (4.00 g, 27.4 mmol, 1.00 equiv.) was dissolved in DMF (30 mL), and was cooled to 0°C. To the solution was added imidazole (14.9 g, 219 mmol, 8.00 equiv.) and TIPSCl (23.4 mL, 109 mmol, 4.00 equiv.). After being stirred at 80°C for 72 h, the reaction mixture was poured into cold sat. aq. $NaHCO_3$ (100 mL). The resultant mixture was filtered thought a paper filter. The aqueous layer was separated and extracted with Et_2O (2×50 mL). The combined organic extracts were washed with water (2×100 mL), Brine (50 mL), and then dried over $MgSO_4$. After removal of the solvents *in vacuo*, flash column chromatography (SiO_2, *i*Hexane/DCM 19:1 to 4:1) furnished the title compound **5c** as a colorless oil (11.2 g, 18.2 mmol, 66%).

^{1}H-NMR ($CDCl_3$, 400 MHz, 300 K) δ = 6.36 (d, *J* = 6.3 Hz, 1H), 4.80 (ddd, *J* = 6.7, 5.2, 1.8 Hz, 1H), 4.23 (ddt, *J* = 7.7, 3.7, 1.8 Hz, 1H), 4.11 – 4.02 (m, 2H), 3.94 (dt, *J* = 5.2, 2.1 Hz, 1H), 3.82 (dd, *J* = 11.3, 3.7 Hz, 1H), 1.06 (d, *J* = 3.9 Hz, 63H).
^{13}C-NMR ($CDCl_3$, 101MHz, 300K) δ = 143.0, 100.5, 80.9, 70.4, 65.1, 62.2, 18.3, 18.2, 18.2, 18.1, 18.1, 12.7, 12.6, 12.4, 12.1.
LR-MS (70eV): m/z [%] = 571.5 (15), 385.3 (80), 359.3 (19), 335.0 (7), 308.9 (17), 253.2 (43), 213.2 (29), 185.1 (29), 157.2 (80), 115.1 (100), 87.1 (66), 59.0 (85).

Spectral characteristics were in agreement with previously reported data.[4]

1,5-Anhydro-4,6-*O*-(di-*tert*-butyl)silanediyl-2-*deoxy*-D-lyxo-hex-1-enitol (<u>SI-3</u>)

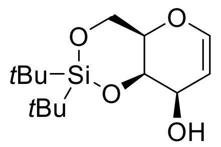

According to a literature procedure[5], D-galactal (121 mg, 830 µmol, 1.00 equiv.) was dissolved in DMF (3 mL) and was cooled to -45°C and $(tBu)_2Si(OTf)_2$ (300 µL, 913 µmol, 1.10 equiv.) was added dropwise over 5 min. The reaction mixture was left stirring at -40°C for 30 min after which pyridine (80 µL, 996 µmol, 1.20 equiv.) was added and the solution was allowed to warm to 0°C. After 30 min at 0°C the reaction was diluted with Et_2O (20 mL) and quenched with sat. aq. $NaHCO_3$ solution (5 mL). The organic layer was washed with Brine (4×20 mL), dried over $MgSO_4$, filtered and concentrated *in vacuo*. The crude product was purified by flash column chromatography (SiO_2, *i*H/EtOAc = 4:1) to afford **<u>SI-3</u>** as a colorless solid (153 mg, 535 µmol, 64%).

^{1}H-NMR ($CDCl_3$, 400MHz, 300K) δ = 6.31 (dd, *J* = 6.4, 1.8 Hz, 1H), 4.71 (dt, *J* = 6.4, 1.8 Hz, 1H), 4.40 (dt, *J* = 5.1, 1.4 Hz, 1H), 4.37 – 4.31 (m, 1H), 4.28 (dd, *J* = 12.5, 1.8 Hz, 1H), 4.23 (dd, *J* = 12.5, 1.8 Hz, 1H), 3.88 (td, *J* = 1.8, 1.0 Hz, 1H), 2.74 (d, J = 11.4 Hz, 1H), 1.08 (s, 9H), 1.02 (s, 9H).
^{13}C-NMR ($CDCl_3$, 101MHz, 300K) δ = 144.1, 103.4, 73.4, 68.8, 67.5, 63.9, 27.8, 27.1, 23.5, 21.1.
LR-MS (70eV): m/z [%] = 253.0 (1), 229.0 (47), 211.0 (9), 199.0 (11), 185.0 (3), 173.0 (80), 161.0 (46), 143.0 (15), 131.0 (12), 115.0 (55), 103.0 (31), 87.0 (8), 77.0 (100), 66.9 (2), 57.1 (45).

Spectral characteristics were in agreement with previously reported data.[5]

(4a*R*,8*R*,8a*S*)-2,2-Di-*tert*-butyl-8-((triisopropylsilyl)oxy)-4,4a,8,8a-tetrahydropyrano[3,2-*d*][1,3,2]dioxasiline (5d)

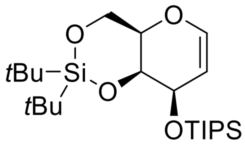

According to a literature procedure[6], to a stirred mixture of **SI-3** (139 mg, 486 µmol, 1.00 equiv.) and imidazole (66.2 mg, 972 µmol, 2.00 equiv.) in DMF (5 mL) was added TIPSCl (217 µL, 1.02 mmol, 2.10 equiv.) dropwise. The solution was heated to 60°C and stirred overnight. The next day, the reaction was quenched with H_2O (10 mL) and the aq. phase was extracted with Et_2O (3×20 mL). The combined organic fractions were dried over $MgSO_4$ and the solvents were removed *in vacuo.* Flash column chromatography (SiO_2, *i*H/EtOAc 19:1 to 9:1) furnished the title compound **5d** as colorless crystals. (194 mg, 438 µmol, 90 %).

^{1}H-NMR ($CDCl_3$, 400MHz, 300K) δ = 6.24 (dd, *J* = 6.4, 1.9 Hz, 1H), 4.63 (dt, *J* = 6.4, 1.9 Hz, 1H), 4.61 – 4.56 (m, 1H), 4.39 – 4.35 (m, 1H), 4.26 (dd, *J* = 12.4, 1.9 Hz, 1H), 4.22 (dd, *J* = 12.4, 1.9 Hz, 1H), 3.84 (t, *J* = 1.6 Hz, 1H), 1.11 – 1.08 (m, 18H), 1.08 – 1.07 (m, 3H), 1.06 (s, 9H), 1.02 (s, 9H).

^{13}C-NMR ($CDCl_3$, 101MHz, 300K) δ = 143.0, 104.0, 73.7, 69.6, 67.7, 66.0, 27.8, 27.2, 23.6, 21.0, 18.2, 18.2, 12.6.

LR-MS (70eV): m/z [%] = 427.2 (1), 399.8 (27), 385.3 (100), 367.2 (1), 343.2 (5), 317.2 (8), 289.1 (1), 269.1 (2), 247.2 (5), 229.2 (28), 211.1 (37), 185.1 (12), 157.1 (23), 135.0 (17), 115.1 (55), 81.1 (62), 57.1 (16).

IR (FT-ATR) $\tilde{\nu}$ [cm^{-1}]: 2940 (m), 2890 (m), 2864 (m), 1740 (vw), 1652 (w), 1474 (m), 1465 (m), 1396 (w), 1388 (w), 1365 (w), 1342 (w), 1274 (w), 1234 (m), 1214 (w), 1178 (s), 1137 (m), 1109 (s), 1081 (vs), 1028 (m), 1013 (m), 996 (m), 988 (m), 933 (s), 924 (s), 908 (s), 881 (s), 860 (s), 825 (s), 788 (m), 758 (s), 739 (m), 717 (m), 680 (s), 664 (s).

Spectral characteristics were in agreement with previously reported data.[6]

(1,5-Anhydro-2-*deoxy*-3,4,6-tris-*O*-triisopropylsilyl-D-*arabino*-hex-1enitolyl)boronic acid pinacol ester (<u>SI-4</u>)

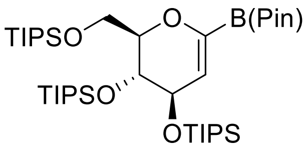

According to a modified literature procedure[7], to a solution of **<u>5c</u>** (2.50 g, 4.06 mmol, 1.00 equiv.) in THF (27 mL) was added *t*BuLi (16.7 mmol, 4.10 equiv.) at -78°C dropwise over 15 min. The dark yellow solution was stirred at -78°C for 15 min, then was allowed to warm to 0°C and stirred at that temperature for 45 min. Following this, 2-isopropoxy-4,4,5,5-tetramethyl-1,3,2-dioxaborolane (3.5 mL, 17.1 mmol, 4.20 equiv.) was added dropwise at -78°C over 15 min. The reaction mixture was stirred at that temperature for 15 min, then allowed to reach rt. and stirred overnight. The mixture was poured into a separatory funnel containing Et_2O (100 mL) and H_2O (75 mL). The organic layer was washed with H_2O (3×75 mL), Brine (75 mL), dried over $MgSO_4$ and the solvents were removed *in vacuo*. The title compound **<u>SI-4</u>** was obtained as colorless oil, which was used in further reactions without additional purification. The title compound was determined to be of 90% purity by ^1H spectroscopy (2.70 g, 3.60 mmol, 90 %).

Note: Any attempts to further purify the title compound by chromatographic separation (SiO_2, Alumina, Florisil) were unsuccessful.

^{1}**H-NMR** ($CDCl_3$, 400MHz, 300K) δ = 5.58 (dd, *J* = 5.3, 1.8 Hz, 1H), 4.31 (ddt, *J* = 7.4, 5.6, 1.8 Hz, 1H), 4.14 (q, *J* = 1.9 Hz, 1H), 3.99 – 3.91 (m, 2H), 3.87 (dd, *J* = 10.7, 5.6 Hz, 1H), 1.28, 1.26 (2 × s, 12H), 1.11 - 0.98 (m, 63H).
^{13}**C-NMR** ($CDCl_3$, 101MHz, 300K) δ = 114.0, 84.1, 79.7, 69.8, 65.1, 61.7, 25.0, 24.4, 18.4, 18.3, 18.3, 18.2, 12.7, 12.5, 12.1.
LR-MS (70eV): m/z [%] = 697.5 (21), 641.4 (1), 597.3 (1), 523.3 (51), 498.0 (15), 385.3 (100), 355.2 (8), 311.1 (11), 245.1 (5), 213.1 (77), 157.1 (55), 115.0 (71), 73.0 (47).
IR (FT-ATR) $\tilde{\nu}$ [cm^{-1}]: 2943 (m), 2893 (m), 2866 (m), 1644 (vw), 1464 (m), 1429 (w), 1407 (w), 1379 (m), 1371 (m), 1334 (m), 1283 (w), 1271 (w), 1250 (w), 1215 (w), 1146

(m), 1102 (s), 1061 (s), 1013 (m), 996 (m), 965 (w), 952 (w), 919 (w), 882 (vs), 864 (m), 848 (m), 830 (w), 811 (w), 761 (m), 741 (w), 716 (w), 679 (vs), 656 (s).

Spectral characteristics were in agreement with previously reported data.[7]

(((2*S*,3*S*,4*S*)-2-Methyl-3,4-dihydro-2*H*-pyran-3,4- diyl)bis(oxy))bis (triisopropylsilane) (<u>10</u>)

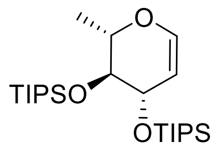

According to GP-D, starting from α-L-Rhamnose (3.3 g, 20 mmol, 1.0 equiv.) to obtain the intermediate (2*S*,3*S*,4*S*)-2-methyl-3,4-dihydro-2*H*-pyran-3,4-diyl diacetate as a colorless oil (1.7 g, 8 mmol, 39 % overall) after column chromatography (SiO_2; pentane – EtOAc | 8:2 to 1:1). This was directly employed in a deprotection-TIPS protection sequence.

The peracetylated glycal (1.7 g, 8 mmol, 1.0 equiv.) was dissolved in MeOH (50 mL) and treated with NaOMe (30w%, 10 mol%) and the reaction mixture was allowed to stir at ambient temperature overnight. After TLC control, the solvents were removed and the crude glycal was dissolved in dry DMF (40 mL) and imidazole (2.1 g, 31 mmol, 4.0 equiv.) was added. TIPSCl (3.7 mL, 17 mmol, 2.2 equiv.) was added dropwise at 0°C, and the mixture was allowed to stir overnight at rt. The next day, H_2O (100 mL) was added and the aq. phase was extracted with EtOAc (3 × 50 mL). The combined organic fractions were washed with water (100 mL) Brine (50 mL), and dried over anhydr. $MgSO_4$. After concentration *in vacuo*, flash column chromatography (SiO_2; pentane – EtOAc | 97:3) afforded the title compound as a colorless oil (5.8 g, 13 mmol, 66 %).

^{1}H-NMR (300 MHz, $CDCl_3$) δ = 6.31 – 6.20 (m, 1H), 4.71 (ddd, *J* = 6.2, 2.5, 0.7 Hz, 1H), 4.30 (dt, *J* = 6.6, 2.0 Hz, 1H), 3.95 (dq, *J* = 8.6, 6.6 Hz, 1H), 3.52 (ddd, *J* = 8.5, 6.2, 4.1 Hz, 1H), 2.19 (d, *J* = 4.1 Hz, 1H), 1.39 (dd, *J* = 6.6, 0.7 Hz, 3H), 1.18 – 0.98 (m, 42H).

^{13}C-NMR (75 MHz, $CDCl_3$) δ = 143.60, 103.46, 75.24, 74.41, 70.27, 18.21, 17.85, 12.59, 12.42.

Spectral characteristics were in agreement with previously reported data.[8]

(((3*R*,4*R*)-3,4-Dihydro-2*H*-pyran-3,4-diyl)bis(oxy))bis(triisopropylsilane) (11)

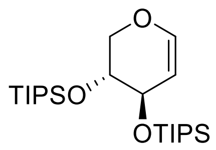

According to GP-D, starting from α-D-Xylose (4.5 g, 30 mmol, 1.0 equiv.) to obtain the intermediate (3*R*,4*R*)-3,4-dihydro-2*H*-pyran-3,4-diyl diacetate as a colorless oil (2.5 g, 13 mmol, 42% overall) after column chromatography (SiO_2; pentane – Acetone | 7:3 to 1:1). This was directly employed in a deprotection-TIPS protection sequence.
The peracetylated glycal (2 g, 10 mmol, 1.0 equiv.) was dissolved in MeOH (50 mL) and treated with NaOMe (30w%, 10 mol%) and the reaction mixture was allowed to stir at ambient temperature for 3 h. After TLC control, the solvents were removed and the crude glycal was dissolved in dry DMF (60 mL) and imidazole (2.7 g, 40 mmol, 4.0 equiv.) was added. TIPSCl (4.7 mL, 22 mmol, 2.2 equiv.) was added dropwise at 0°C, and the mixture was allowed to stir overnight at rt. The next day, H_2O (100 mL) was added and the aq. phase was extracted with EtOAc (3 × 50 mL). The combined organic fractions were washed with water (50 mL), Brine (50 mL), and dried over anhydr. $MgSO_4$. After concentration *in vacuo*, flash column chromatography (SiO_2; pentane – EtOAc | 99:1 to 96:4) afforded the title compound as a colorless oil (2.7 g, 6.3 mmol, 48 %).

^{1}H-NMR ($CDCl_3$, 500MHz, 300K) δ = 6.43 (d, *J* = 6.2 Hz, 1H), 4.83 (ddd, *J* = 6.2, 5.3, 1.7 Hz, 1H), 3.99 (t, *J* = 1.7 Hz, 2H), 3.95 – 3.89 (m, 1H), 3.85 (p, *J* = 2.1 Hz, 1H), 1.08 – 1.04 (m, 42H).
^{13}C-NMR ($CDCl_3$, 126MHz, 300K) δ = 145.44, 101.28, 69.64, 65.96, 64.28, 18.27, 18.21, 18.17, 18.13, 12.61, 12.57.
HR-MS (ESI): m/z calcd. for ($[C_{23}H_{49}O_3Si_2]^+$, $[M^+]$): 429.3222, found: 429.3216.

(3a*S*,7a*R*)-2,2-dimethyl-3a,7a-dihydro-4*H*-[1,3]dioxolo[4,5-c]pyran (12)

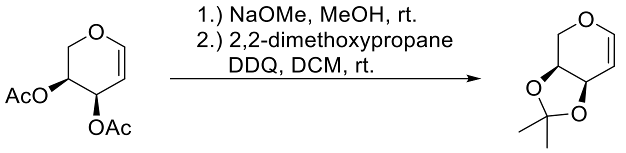

(3*S*,4*R*)-3,4-dihydro-2H-pyran-3,4-diyl diacetate (4 g, 20 mmol, 1.0 equiv.) was suspended in MeOH (50 mL) and treated with NaOMe (30w%, 10 mol%). After stirring for 2 h at ambient temperature the solvent was removed *in vacuo*. According to a modified literature procedure[9], the now deprotected (3*S*,4*R*)-3,4-dihydro-2H-pyran-3,4-diol (L-Arabinal) (10 mmol, 1.0 equiv.) was charged to a flame-dried flask and dissolved in dry DCM (120 mL). 2,2-Dimethoxypropane (4.9 mL, 40 mmol, 2.0 equiv.) and DDQ (0.45 g, 2 mmol, 10 mol%) was added, and the reaction mixture was allowed to stir at ambient temperature for 12 h. The next day, the solvents were removed *in vacuo* and the dark residue was purified by flash column chromatography (SiO_2; pentane – Et_2O |99:1 to 6:4) to obtain the title compound as a colorless oil (0.56 g, 3.6 mmol, 36 %).

Caution: the title compound is quite volatile

^{1}**H-NMR** ($CDCl_3$, 400MHz, 300K) δ = 6.54 (d, J = 6.1 Hz, 1H), 5.04 – 4.98 (m, 1H), 4.48 (t, J = 5.0 Hz, 1H), 4.19 (ddd, J = 8.0, 5.7, 4.1 Hz, 1H), 4.02 (dd, J = 11.1, 4.1 Hz, 1H), 3.63 (dd, J = 11.1, 8.0 Hz, 1H), 1.48 (s, 3H), 1.39 (s, 3H).
^{13}**C-NMR** ($CDCl_3$, 101MHz, 300K) δ = 148.23, 109.51, 100.59, 71.08, 67.73, 65.63, 28.98, 26.70.
Spectral characteristics were in agreement with previously reported data.[9]

Boronic esters

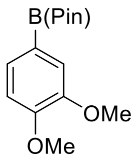

SI-5

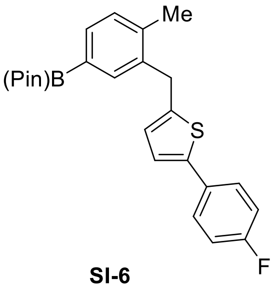

SI-6

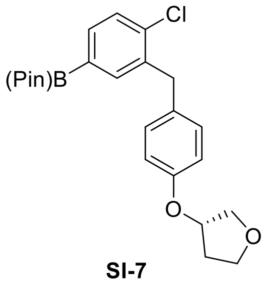

SI-7

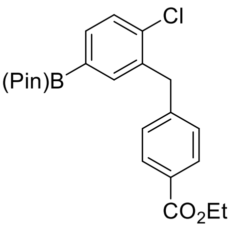

SI-8

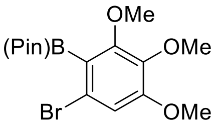

SI-9

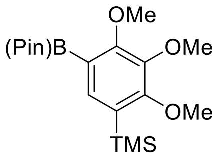

SI-10

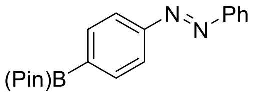

SI-11

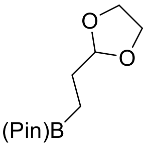

SI-12

- All other boronic acid pinacol esters were obtained from commercial sources.

3,4-Dimethoxyphenylboronic acid pinacol ester (SI-5)

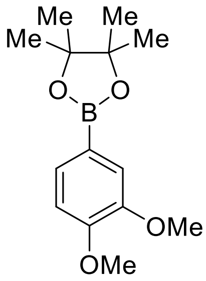

According to GP-C on a 10 mmol scale, with 4-bromoveratrole (1.3 mL, 10 mmol, 1.0 equiv.) in THF (12 mL) and *n*BuLi (11 mmol, 1.1 equiv.). Flash column chromatography (SiO_2, *i*H/EtOAc 9:1 to 3:1) furnished **SI-5** as colorless crystals (1.6 g, 6 mmol, 60 %).

^{1}**H-NMR** ($CDCl_3$, 400MHz, 300K) δ = 7.42 (dd, *J* = 8.0, 1.5 Hz, 1H), 7.28 (d, *J* = 1.5 Hz), 6.88 (d, *J* = 8.0 Hz), 3.92 (s, 3H), 3.90 (s, 3H), 1.34 (s, 12H).
^{13}**C-NMR** ($CDCl_3$, 101MHz, 300K) δ = 151.7, 148.4, 128.7, 116.6, 110.6, 83.8, 56.0, 55.9, 25.0.

Spectral characteristics were in agreement with previously reported data.[10]

2-(3-((5-(4-Fluorophenyl)thiophen-2-yl)methyl)-4-methylphenyl)-4,4,5,5-tetramethyl-1,3,2-dioxaborolane (SI-6)

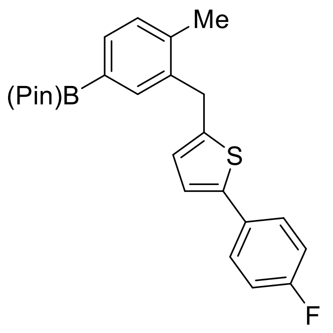

2-(4-Fluorophenyl)-5-[(5-iodo-2-methylphenyl)methyl]thiophene (6.1 g, 15 mmol, 1.0 equiv.) was dissolved in dry THF (70 mL) and cooled to -78°C. A solution of *n*BuLi in hexanes (19.5 mmol, 1.3 equiv.) was added dropwise to the mixture over 30 min *via* syringe pump. After stirring at the aforementioned temperature for a further 30 min, 2-methoxy-4,4,5,5-tetramethyl-1,3,2-dioxaborolane (3.7 mL, 22.5 mmol, 1.5 equiv.) was added dropwise. After 1 h at -78°C, the reaction mixture was allowed to warm to rt. stirring at this temperature for 1 h. The reaction was quenched by addition of sat. aq. NH_4Cl (10 mL) and H_2O (30 mL) was added. The aq. phase was extracted with EtOAc (3 × 100 mL), the combined org. fractions were washed with Brine (50 mL) and dried over anhydr. $MgSO_4$. After concentration *in vacuo,* flash-column chromatography (SiO_2, pentane – EtOAc: 97:3 -> 85:5) afforded the title compound as a colorless viscous oil (4.9 g, 12 mmol, 82 %).

^{1}H-NMR: ($CDCl_3$, 400 MHz, 300 K) δ = 7.75 (d, *J* = 1.3 Hz, 1H), 7.70 (dd, *J* = 7.4, 1.3 Hz, 1H), 7.51 (ddd, *J* = 8.7, 3.7, 3.2 Hz, 1H), 7.49 (ddd, *J* = 8.7, 3.7, 3.2 Hz, 1H), 7.24 (d, *J* = 7.4 Hz, 1H), 7.11 – 6.98 (m, 3H), 6.66 (dd, *J* = 3.7, 0.8 Hz, 1H), 4.18 (s, 2H), 2.37 (s, 3H), 1.39 (s, 12H)
^{13}C-NMR: ($CDCl_3$, 101 MHz, 300 K) δ = 162.05, 143.8, 141.3, 140.1, 137.5, 136.2, 133.7, 131.0, 130.9, 130.2, 127.0, 125.7, 122.6, 115.8, 83.7, 34.3, 24.9, 19.8.
Spectral characteristics were in agreement with previously reported data.[11]

(*S*)-2-(4-Chloro-3-(4-((tetrahydrofuran-3-yl)oxy)benzyl)phenyl)-4,4,5,5-tetramethyl-1,3,2-dioxaborolane (SI-7)

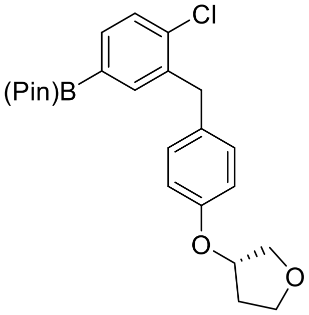

(*S*)-3-(4-(5-Bromo-2-chlorobenzyl)phenoxy)tetrahydrofuran (3.7 g, 10 mmol, 1.0 equiv.) was dissolved in dry THF (50 mL) and cooled to -78°C. A solution of *n*BuLi in hexanes (13 mmol, 1.3 equiv.) was added dropwise to the mixture over 30 min *via* syringe pump. After stirring at the aforementioned temperature for a further 30 min, 2-methoxy-4,4,5,5-tetramethyl-1,3,2-dioxaborolane (2.5 mL, 15 mmol, 1.5 equiv.) was added dropwise. After 1 h at -78°C, the reaction mixture was allowed to warm to rt. stirring at this temperature for 1 h. The reaction was quenched by addition of sat. aq. NH_4Cl (8 mL) and H_2O (20 mL) was added. The aq. phase was extracted with EtOAc (3 × 75 mL), the combined org. fractions were washed with Brine (40 mL) and dried over anhydr. $MgSO_4$. After concentration *in vacuo,* flash-column chromatography (SiO_2, pentane – EtOAc: 97:3 -> 9:1) afforded the title compound as a colorless oil, that solidified in the fridge (3.2 g, 7.6 mmol, 76 %).

^{1}H-NMR (300 MHz, $CDCl_3$) δ = 7.69 (d, *J* = 1.6 Hz, 1H), 7.60 (dd, *J* = 7.9, 1.6 Hz, 1H), 7.37 (d, *J* = 7.9 Hz, 1H), 7.13 – 7.07 (m, 2H), 6.79 – 6.74 (m, 2H), 4.88 (tt, *J* = 5.2, 2.7 Hz, 1H), 4.05 (s, 2H), 4.02 – 3.85 (m, 4H), 2.20 – 2.11 (m, 2H), 1.33 (s, 12H).
^{13}C-NMR (75 MHz, $CDCl_3$) δ = 155.86, 138.21, 137.79, 134.26, 132.32, 129.86, 129.29, 115.38, 84.13, 77.39, 73.28, 67.33, 38.46, 33.14, 25.00.

Spectral characteristics were in agreement with previously reported data.[12]

Ethyl 4-(2-chloro-5-(4,4,5,5-tetramethyl-1,3,2-dioxaborolan-2-yl)benzyl)benzoate (SI-8)

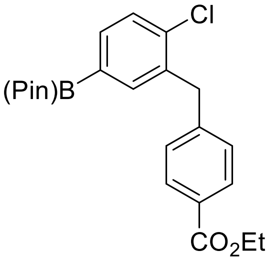

Ethyl 4-(5-bromo-2-chlorobenzyl)benzoate (1.8 g, 5 mmol, 1.0 equiv.) was dissolved in dry THF (30 mL) and cooled to -78°C. A solution of *n*BuLi in hexanes (6.5 mmol, 1.3 equiv.) was added dropwise to the mixture over 30 min *via* syringe pump. After stirring at the aforementioned temperature for a further 30 min, 2-methoxy-4,4,5,5-tetramethyl-1,3,2-dioxaborolane (1.2 mL, 7.5 mmol, 1.5 equiv.) was added dropwise. After 1 h at -78°C, the reaction mixture was allowed to warm to rt. stirring at this temperature for 1 h. The reaction was quenched by addition of sat. aq. NH_4Cl (5 mL) and H_2O (15 mL) was added. The aq. phase was extracted with EtOAc (3 × 50 mL), the combined org. fractions were washed with Brine (50 mL) and dried over anhydr. $MgSO_4$. After concentration *in vacuo,* flash-column chromatography (SiO_2, pentane – EtOAc: 92:8) afforded the title compound as a colorless solid (1.7 g, 4.3 mmol, 86 %).

^{1}H-NMR (300 MHz, $CDCl_3$) δ = 7.70 (d, J = 1.6 Hz, 1H), 7.60 (dd, J = 7.9, 1.6 Hz, 1H), 7.37 (d, J = 7.9 Hz, 1H), 7.13 – 7.06 (m, 2H), 6.84 – 6.77 (m, 2H), 4.06 (s, 2H), 3.99 (q, J = 7.0 Hz, 2H), 1.39 (t, J = 7.0 Hz, 3H), 1.34 (s, 12H).
^{13}C-NMR (75 MHz, $CDCl_3$) δ = 157.41, 138.36, 137.79, 134.19, 131.82, 129.75, 129.27, 114.47, 84.11, 63.48, 38.48, 25.00.^

HR-MS (ESI): m/z calcd. for ($[C_{22}H_{27}BClO_4]^+$, $[M]^+$): 401.7140, found: 401.7144.

2-(6-Bromo-2,3,4-trimethoxyphenyl)-4,4,5,5-tetramethyl-1,3,2-dioxaborolane (SI-9)

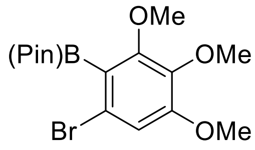

5-Bromo-1,2,3-trimethoxybenzene (3.3 g, 13 mmol, 1.0 equiv.) was dissolved in anhydr. Et_2O (10 mL) and was added in one portion to a freshly prepared solution of TMPLi (42 mmol, 3.2 equiv.) in Et_2O (90 mL) and THF (30 mL) at -100 °C (MeOH/ liquid N_2). After stirring for 1 min at the aforementioned temperature, 2-methoxy-4,4,5,5-tetramethyl-1,3,2-dioxaborolane (6.5 mL, 40 mmol, 3.0 equiv.) dissolved in Et_2O (20 mL) was added rapidly to the solution. The cooling bath was removed, and the mixture was allowed to reach ambient temperature. After that the reaction was quenched by addition of sat. aq. NH_4Cl (10 mL) and was transferred to a seperatory funnel. H_2O (50 mL) was added, and the aq. fraction was extracted with Et_2O (3 × 100 mL). The combined organic fractions were washed with Brine (50 mL) and dried over anhydr. $MgSO_4$. After removal of the solvents *in vacuo* and flash column chromatography (SiO_2; pentane/ EtOAc 97:3 -> 88:12), the title compound was obtained as a colorless oil, which solidified in the fridge (2.4 g, 6.4 mmol, 48 %).

^{1}H-NMR (300 MHz, $CDCl_3$) δ = 6.79 (s, 1H), 3.86 (s, 3H), 3.81 (d, J = 3.7 Hz, 6H), 1.39 (s, 12H).
^{13}C-NMR: ($CDCl_3$, 101 MHz, 300 K) δ = 157.01, 155.24, 141.06, 119.32, 112.16, 84.52, 24.86.

HR-MS (ESI): m/z calcd. for ($[C_{15}H_{23}BBrO_5]^+$, $[M]^+$): 374.0580, found: 374.0574.

Trimethyl(2,3,4-trimethoxy-5-(4,4,5,5-tetramethyl-1,3,2-dioxaborolan-2-yl)phenyl)silane (SI-10)

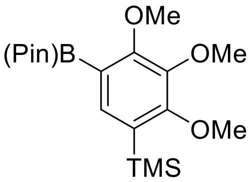

1,2,3-Trimethoxybenzene (0.84 g, 5 mmol, 1.0 equiv.) was added suspended in dry Et_2O (20 mL) and cooled to -20°C. Dry TMEDA (0.9 mL, 6 mmol, 1.2 equiv.) was added in one portion, followed by dropwise addition of *s*BuLi (6 mmol, 1.2 equiv.). After stirring at this temperature for 30 min, TMSCl (0.69 mL, 5.5 mmol, 1.1 equiv.) was added dropwise, and the mixture was allowed to warm to rt., stirring at this temperature for 2 h. The reaction was quenched by addition of sat. aq. NH_4Cl (5 mL), H_2O (20 mL) was added and the aq. fraction was extracted with EtOAc (3 × 50 mL). The combined organic phases were washed with Brine, dried over anhydr. $MgSO_4$ and the solvents were removed *in vacuo*. The crude trimethyl(2,3,4-trimethoxyphenyl)silane was dried under high-vacuum, before being dissolved in Et_2O (20 mL) and cooling to -20°C. Dry TMEDA (0.9 mL, 6 mmol, 1.2 equiv.) was added in one portion, followed by dropwise addition of *s*BuLi (6 mmol, 1.2 equiv.). After stirring at this temperature for 30 min, 2-methoxy-4,4,5,5-tetramethyl-1,3,2-dioxaborolane (0.98 mL, 6 mmol, 1.2 equiv.) was added dropwise and the mixture was allowed to warm to rt. stirring at this temperature overnight. The reaction was quenched by addition of sat. aq. NH_4Cl (5 mL), H_2O (20 mL) was added and the aq. fraction was extracted with EtOAc (3 × 50 mL). The combined organic phases were washed with Brine, dried over anhydr. $MgSO_4$ and the solvents were removed *in vacuo*. The crude product was purified *via* flash column chromatography (SiO_2; pentane/ EtOAc: 99:1 to 92:8) to obtain the title compound as a colorless solid (0.73 g, 3.1 mmol, 61 %)

^{1}H-NMR (300 MHz, $CDCl_3$) δ = 7.41 (s, 1H), 3.93 (s, 3H), 3.87 (d, J = 1.5 Hz, 6H), 1.34 (s, 12H), 0.26 (s, 9H).

^{13}C-NMR: ($CDCl_3$, 101 MHz, 300 K) δ = 145.13, 136.70, 129.37, 128.26, 83.51, 61.96, 60.84, 24.95, -0.41.

(*E*)-1-phenyl-2-(4-(4,4,5,5-tetramethyl-1,3,2-dioxaborolan-2-yl)phenyl)diazene (SI-11)

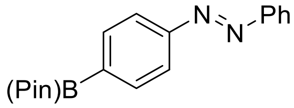

According to a modified literature procedure [13], (*E*)-1-(4-iodophenyl)-2-phenyldiazene (0.71 g, 2.3 mmol, 1.0 equiv.) was charged to an Ace pressure tube, containing dry KOAc (0.5 g, 5.1 mmol, 2.2 equiv.), bis(pinacolato)diboron (0.8 g, 3.2 mmol, 1.4 equiv.) and $PdCl_2(dppf)_2$ (220 mg, 0.3 mmol, 13 mol%). The pressure tube was evacuated and backfilled with Ar for three times, and dry dioxane (20 mL) was added. The vial was closed and the reaction mixture was heated to 90°C overnight. The next day, the reaction mixture was diluted with Et_2O and passed through a plug of silica, eluting with Et_2O (150 mL). After removing the solvents *in vacuo*, the crude product was purified by flash column chromatography (SiO_2; pentane/ EtOAc: 99:1 to 95:5) to obtain the title compound as an amorphous red solid (0.41 g, 1.3 mmol, 58 %).

^{1}H-NMR (500 MHz, $CDCl_3$) δ = 7.99 – 7.89 (m, 6H), 7.55 – 7.46 (m, 3H), 1.38 (s, 12H).
^{13}C-NMR (126 MHz, $CDCl_3$) δ = 154.50, 152.85, 135.79, 131.31, 129.24, 123.09, 122.11, 84.21, 25.05.

Spectral characteristics were in agreement with previously reported data.[13]

2-(2-(1,3-Dioxolan-2-yl)ethyl)-4,4,5,5-tetramethyl-1,3,2-dioxaborolane (SI-12)

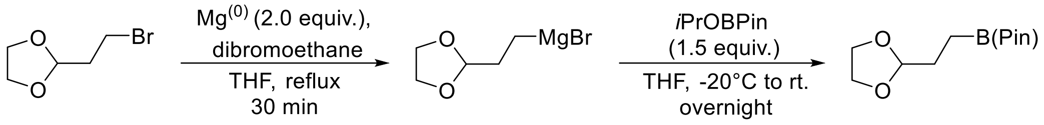

In a flame-dried Schlenk flask, magnesium turnings (1.49 g, 60 mmol, 2.0 equiv.) were suspended in dry THF (10 mL) and dibromoethane (5 drops) was added. The magnesium was activated by heating to reflux with a heat-gun twice. In a separate flask, 2-(2-bromoethyl)-1,3-dioxolane (3.5 mL, 30 mmol, 1.0 equiv.) was dissolved in THF (20 mL) and then added dropwise to the activated magnesium. Iodometric titration gave a concentration of 0.93 M.

A fraction of this grignard solution (10 mmol, 1.0 equiv.) was then added dropwise to a solution of *i*PrOBPin (3.1 mL, 15 mmol, 1.5 equiv.) in THF (20 mL) at -20°C. After 1 h at this temperature, the cooling bath was removed and the reaction mixture was allowed to stir at rt. overnight. The next day, sat. aq. NH_4Cl (5 mL), followed by water (30 mL) was added. The mixture was transferred to a separatory funnel and the aq. fraction was extracted with EtOAc (3 × 50 mL). The combined org. phases were washed with Brine (50 mL), dried over anhydr. $MgSO_4$ and concentrated *in vacuo*. Flash column chromatography (SiO_2; pentane/ EtOac = 95:5 to 9:1) gave the title compound as a viscous oil (1.9 g, 8.4 mmol, 84%).

^{1}H-NMR (500 MHz, $CDCl_3$) δ = 4.85 (t, J = 4.4 Hz, 1H), 3.93 – 3.87 (m, 2H), 3.82 – 3.76 (m, 2H), 1.75 (td, J = 7.8, 4.4 Hz, 2H), 1.20 (s, 12H), 0.80 (t, J = 7.8 Hz, 2H).
^{13}C-NMR (126 MHz, $CDCl_3$) δ = 105.30, 83.04, 65.01, 28.35, 24.87.

D-Glucal Series

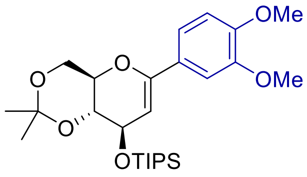

7a (74%)

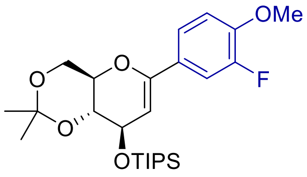

7b (64%)

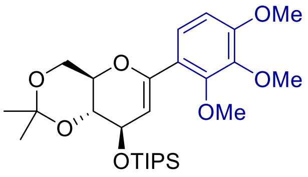

7c (67%)

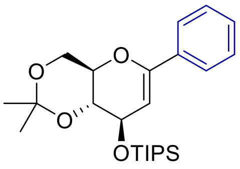

7d (83%)

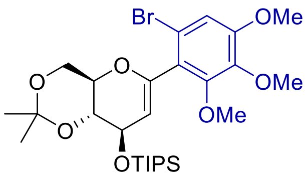

7e (81%)

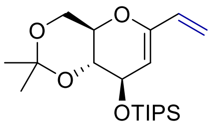

7f (51%)

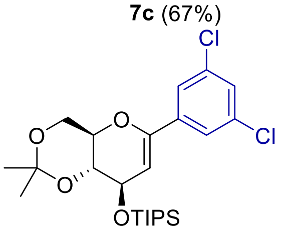

7g (72%)

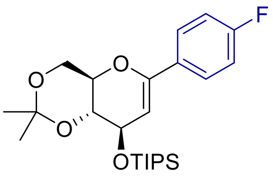

7h (61%)

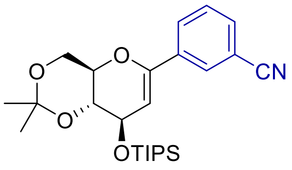

7i (62%)

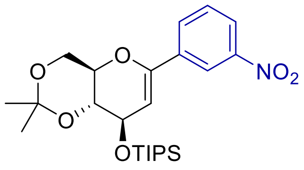

7j (68%)

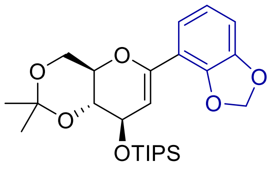

7k (28%)

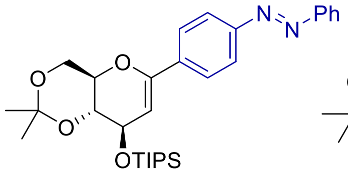

7l (68%)

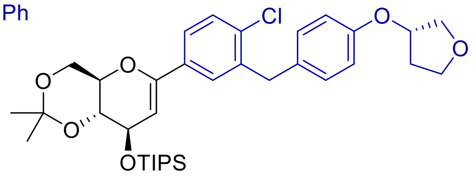

7m (74%)

(((4a*R*,8*R*,8a*R*)-6-(3,4-Dimethoxyphenyl)-2,2-dimethyl-4,4a,8,8a-tetrahydropyrano[3,2-*d*][1,3]dioxin-8-yl)oxy)triisopropylsilane (7a)

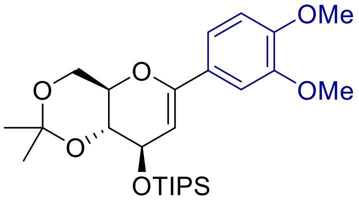

According to GP-B, using *t*BuLi (0.22 mmol, 1.1 equiv.) and stirring for 60 min at -30°C for lithiation and employing 2-(3,4-dimethoxyphenyl)-4,4,5,5-tetramethyl-1,3,2-dioxaborolane (61 mg, 0.23 mmol, 1.3 equiv.). Flash column chromatography (SiO_2 – pentane/ EtOAc 199:1 to 99:1) furnished the title compound as a colorless oil (71 mg, 0.15 mmol, 74 %).

^{1}H-NMR (400 MHz, $CDCl_3$) δ = 7.11 (dd, *J* = 8.4, 2.0 Hz, 1H), 7.03 (d, *J* = 2.0 Hz, 1H), 6.82 (d, *J* = 8.4 Hz, 1H), 5.11 (d, *J* = 2.3 Hz, 1H), 4.57 (dd, *J* = 7.0, 2.3 Hz, 1H), 4.08 (dd, *J* = 10.6, 5.1 Hz, 1H), 3.99 – 3.92 (m, 1H), 3.91 – 3.86 (m, 10H), 3.48 (q, *J* = 7.0 Hz, 1H), 1.53 (s, 3H), 1.43 (s, 3H), 1.23 – 1.19 (m, 3H), 1.14 – 1.08 (m, 18H).
^{13}C-NMR (101 MHz, $CDCl_3$) δ = 151.51, 149.62, 148.67, 127.38, 118.20, 110.79, 108.52, 100.54, 99.65, 73.48, 69.93, 68.78, 66.01, 62.12, 56.03, 29.13, 19.08, 18.18, 18.10, 12.47.
HR-MS (EI pos): m/z calcd. for ($[C_{26}H_{42}O_6Si]^+$, $[M]^+$): 478.2751, found: 478.2740.
IR (FT-ATR) $\tilde{\nu}$ [cm^{-1}]: 2993 (w), 2940 (m), 2866 (m), 1645 (w), 1606 (w), 1584 (w), 1515 (s), 1463 (m), 1417 (w), 1382 (m), 1370 (m), 1346 (w), 1325 (w), 1288 (w), 1260 (s), 1244 (m), 1211 (m), 1201 (m), 1172 (s), 1141 (m), 1114 (s), 1100 (vs), 1056 (s), 1030 (s), 1013 (m), 998 (m), 944 (m), 920 (m), 909 (m), 882 (m), 867 (s), 843 (m), 825 (w), 802 (s), 765 (s), 724 (w), 677 (s), 659 (m).

(((4a*R*,8*R*,8a*R*)-6-(3-Fluoro-4-methoxyphenyl)-2,2-dimethyl-4,4a,8,8a-tetrahydropyrano[3,2-*d*][1,3]dioxin-8-yl)oxy)triisopropylsilane (7b)

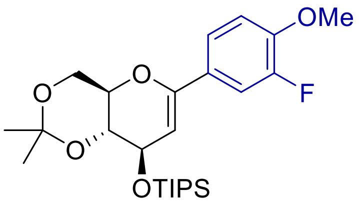

According to GP-B, using *t*BuLi (0.22 mmol, 1.1 equiv.) and stirring for 60 min at -30°C for lithiation and employing 2-(3-fluoro-4-methoxyphenyl)-4,4,5,5-tetramethyl-1,3,2-dioxaborolane (50 mg, 0.20 mmol, 1.0 equiv.). Flash column chromatography (SiO_2 – pentane/ EtOAc 199:1 to 32:1) furnished the title compound as a colorless oil (60 mg, 0.13 mmol, 64 %).

^{1}H-NMR: ($CDCl_3$, 400 MHz, 300 K) δ = 7.26 – 7.22 (m, 2H), 6.89 (t, *J* = 8.5 Hz, 1H), 5.11 (d, *J* = 2.3 Hz, 1H), 4.55 (dd, *J* = 6.9, 2.3 Hz, 1H), 4.06 (dd, *J* = 10.6, 5.1 Hz, 1H), 3.96 – 3.90 (m, 1H), 3.88 (s, 3H), 3.87 – 3.80 (m, 2H), 1.52 (s, 3H), 1.42 (s, 3H), 1.09 (td, *J* = 3.6, 1.7 Hz, 21H).
^{13}C-NMR: ($CDCl_3$, 101 MHz, 300 K) δ = 153.31, 150.88, 150.31 (d, *J* = 2.3 Hz), 148.03 (d, *J* = 11.0 Hz), 127.61 (d, *J* = 6.7 Hz), 121.13 (d, *J* = 3.5 Hz), 113.14 (d, *J* = 20.0 Hz), 112.83 (d, *J* = 2.1 Hz), 100.97, 99.68, 73.36, 69.92, 68.68, 62.03, 56.33, 29.85, 29.11, 19.05, 18.18, 18.10, 12.44.
^{19}F-NMR: ($CDCl_3$, 377 MHz, 300 K) δ = -135.39, -135.40, -135.42, -135.42, -135.43, -135.45, -135.46.
HR-MS (EI pos): m/z calcd. for ($[C_{25}H_{39}FO_5Si]^+$, $[M]^+$): 466.2551, found: 466.2550.
IR (FT-ATR) $\tilde{\nu}$ [cm^{-1}]: 2994 (w), 2942 (m), 2866 (m), 1646 (w), 1620 (w), 1581 (w), 1517 (s), 1464 (m), 1436 (m), 1424 (w), 1383 (m), 1370 (m), 1343 (m), 1313 (w), 1280 (m), 1259 (m), 1234 (m), 1218 (m), 1202 (m), 1192 (m), 1172 (m), 1134 (s), 1112 (s), 1099 (vs), 1056 (s), 1032 (s), 1010 (m), 998 (m), 967 (w), 945 (w), 927 (m), 914 (m), 882 (s), 869 (s), 847 (m), 803 (s), 762 (s), 718 (w), 679 (s), 658 (m).

(((4a*R*,8*R*,8a*R*)-2,2-Dimethyl-6-(2,3,4-trimethoxyphenyl)-4,4a,8,8a-tetrahydropyrano[3,2-*d*][1,3]dioxin-8-yl)oxy)triisopropylsilane (7c)

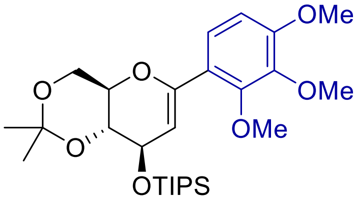

According to GP-B, using *t*BuLi (0.22 mmol, 1.1 equiv.) and stirring for 60 min at -30°C for lithiation and employing 4,4,5,5-tetramethyl-2-(2,3,4-trimethoxyphenyl)-1,3,2-dioxaborolane (68 mg, 0.23 mmol, 1.15 equiv.). Flash column chromatography (SiO_2 – pentane/ EtOAc 99:1 to 9:1) furnished the title compound as a colorless oil (68 mg, 0.13 mmol, 67 %). The title compound was employed in the Hydroboration-oxidation procedure to furnish compound **18**.

^{1}H-NMR (500 MHz, $CDCl_3$) δ = 7.15 (d, *J* = 8.8 Hz, 1H), 6.63 (d, *J* = 8.8 Hz, 1H), 5.33 (d, *J* = 2.2 Hz, 1H), 4.57 (dd, *J* = 7.1, 2.2 Hz, 1H), 4.02 (dd, *J* = 10.3, 5.0 Hz, 1H), 3.95 – 3.90 (m, 2H), 3.90 – 3.88 (m, 1H), 3.86 – 3.85 (m, 9H), 1.54 (s, 3H), 1.42 (s, 3H), 1.15 – 1.05 (m, 21H).
Note: ^{1}H-NMR shows trace of aromatic impurity.
^{13}C-NMR (126 MHz, $CDCl_3$) δ = 154.18, 152.18, 148.93, 142.88, 142.72, 132.73, 123.47, 121.91, 109.93, 107.02, 105.10, 99.62, 73.52, 69.90, 68.93, 63.00, 62.13, 61.04, 60.95, 56.12, 29.18, 19.14, 18.17, 18.08, 12.46.

HR-MS (ESI): m/z calcd. for ([$C_{27}H_{44}O_7Si$]$^+$, [H^+]): 509.2936, found: 509.293.

(((4a*R*,8*R*,8a*R*)-2,2-Dimethyl-6-phenyl-4,4a,8,8a-tetrahydropyrano[3,2-*d*][1,3]dioxin-8-yl)oxy)triisopropylsilane (7d)

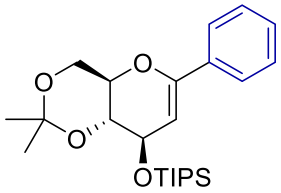

According to GP-B, using *t*BuLi (0.22 mmol, 1.1 equiv.) and stirring for 60 min at -30°C for lithiation and employing 4,4,5,5-tetramethyl-2-phenyl-1,3,2-dioxaborolane (47 mg, 0.23 mmol, 1.15 equiv.). Flash column chromatography (SiO_2 – pentane/ EtOAc 299:1 to 99:1) furnished the title compound as a colorless oil (70 mg, 0.17 mmol, 83 %).

^{1}H-NMR: ($CDCl_3$, 400 MHz, 300 K) δ = 7.56 – 7.52 (m, 2H), 7.36 – 7.31 (m, 3H), 5.25 (d, *J* = 2.3 Hz, 1H), 4.60 (dd, *J* = 6.9, 2.3 Hz, 1H), 4.09 (dd, *J* = 10.4, 5.0 Hz, 1H), 4.00 – 3.88 (m, 3H), 1.55 (s, 3H), 1.44 (s, 3H), 1.13 (dq, *J* = 5.9, 3.2 Hz, 21H).
^{13}C-NMR: ($CDCl_3$, 101 MHz, 300 K) δ = 151.57, 134.38, 128.79, 128.33, 125.19, 101.71, 99.66, 73.43, 69.94, 68.78, 62.11, 29.14, 19.08, 18.20, 18.12, 12.47.

LR-MS (70 eV): m/z [%] = 375.1 (46), 343.1 (5), 317.1 (25), 289.0 (9), 261.1 (15), 235.0 (8), 211.0 (4), 175.0 (8), 157.0 (17), 131.0 (16), 105.0 (100), 75.0 (27), 59.0 (15).
HR-MS (EI pos): m/z calcd. for ($[C_{24}H_{38}O_4Si]^+$, $[M^+]$): 418.2539, found: 418.2543.
IR (FT-ATR) $\tilde{\nu}$ [cm^{-1}]: 2994 (w), 2942 (m), 2866 (m), 1647 (w), 1496 (w), 1463 (w), 1448 (w), 1382 (m), 1370 (m), 1338 (w), 1282 (w), 1270 (w), 1258 (m), 1218 (w), 1200 (m), 1170 (m), 1116 (s), 1100 (vs), 1056 (s), 1031 (s), 1014 (m), 998 (m), 943 (m), 921 (w), 885 (s), 860 (m), 808 (s), 780 (m), 754 (s), 712 (w), 687 (s), 679 (s), 656 (m).

(((4a*R*,8*R*,8a*R*)-6-(6-Bromo-2,3,4-trimethoxyphenyl)-2,2-dimethyl-4,4a,8,8a-tetrahydropyrano[3,2-*d*][1,3]dioxin-8-yl)oxy)triisopropylsilane (7e)

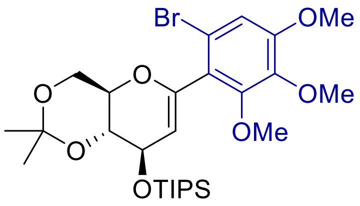

According to GP-B on a 0.5 mmol scale, using *t*BuLi (0.55 mmol, 1.1 equiv.) and stirring for 60 min at -30°C for lithiation and employing 2-(6-bromo-2,3,4-trimethoxyphenyl)-4,4,5,5-tetramethyl-1,3,2-dioxaborolane (215 mg, 0.58 mmol, 1.15 equiv.). Flash column chromatography (SiO_2 – pentane/ EtOAc 97:3 to 7:3) furnished the title compound as a colorless oil (0.24 g, 0.41 mmol, 81 %).

^{1}H-NMR: (500 MHz, $CDCl_3$) δ = 6.86 (s, 1H), 4.93 (d, *J* = 10.3 Hz, 1H), 4.36 (dd, *J* = 10.3, 8.5 Hz, 1H), 4.15 (s, 3H), 3.93 (dd, *J* = 10.3, 5.2 Hz, 1H), 3.82 (s, 3H), 3.81 – 3.73 (m, 2H), 3.71 (s, 3H), 3.57 (td, *J* = 10.3, 5.2 Hz, 1H), 1.49 (s, 3H), 1.38 (s, 3H), 1.27 (m, 3H), 1.15 (t, *J* = 7.3 Hz, 18H).
^{13}C-NMR: (126 MHz, $CDCl_3$) δ = 153.31, 151.99, 139.32, 126.17, 115.74, 114.60, 99.55, 85.35, 82.65, 77.41, 77.36, 77.16, 76.90, 75.13, 74.14, 70.42, 62.63, 62.14, 60.58, 56.31, 36.99, 29.29, 28.09, 25.85, 25.64, 18.89, 18.79, 18.61, 13.66.

HR-MS (ESI): m/z calcd. for ($[C_{27}H_{43}BrO_7Si]^+$, $[M^+]$): 587.2041, found: 587.204.

(((4a*R*,8*R*,8a*R*)-2,2-Dimethyl-6-vinyl-4,4a,8,8a-tetrahydropyrano[3,2-*d*][1,3]dioxin-8-yl)oxy)triisopropylsilane (7f)

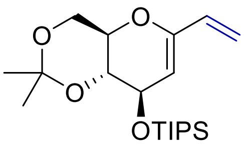

According to GP-B on a 0.2 mmol scale, using *t*BuLi (0.22 mmol, 1.1 equiv.) and stirring for 60 min at -30°C for lithiation and employing 4,4,5,5-tetramethyl-2-vinyl-1,3,2-dioxaborolane (58 mg, 0.23 mmol, 1.15 equiv.). Flash column chromatography (SiO_2 – pentane/ EtOAc 99.6:0.4 to 99:1) furnished the title compound as a colorless oil (38 mg, 0.1 mmol, 51 %). The title compound was obtained as a 3:1 mixture with the respective 6-iodoglucal. The yield was determined by GC-analysis.

^{1}H-NMR (400 MHz, $CDCl_3$) δ = 6.04 (dd, *J* = 17.2, 10.9 Hz, 1H), 5.53 – 5.42 (m, 1H), 5.11 (dd, *J* = 10.9, 1.6 Hz, 1H), 4.72 (d, *J* = 2.3 Hz, 1H), 4.48 (dd, *J* = 7.2, 2.3 Hz, 1H), 3.95 – 3.79 (m, 4H), 1.50 (d, *J* = 2.6 Hz, 3H), 1.40 (s, 3H), 1.08 (qd, *J* = 2.9, 1.7 Hz, 21H).
^{13}C-NMR (101 MHz, $CDCl_3$) δ = 150.46, 131.23, 114.95, 106.68, 99.62, 73.41, 69.55, 68.70, 62.07, 29.11, 19.04, 18.15, 18.06, 12.42.

HR-MS (EI pos): m/z calcd. for ($[C_{17}H_{29}O_4Si]^+$, [M-*i*Pr]$^+$): 325.1835, found: 325.1830.
LR-MS (70 eV): m/z [%] = 325.1 (98), 293.1 (9), 267.1 (50), 239.0 (13), 225.0 (10), 211.1 (100), 185.0 (25), 169.0 (10), 154.9 (9), 141.0 (10), 124.9 (21), 107.0 (38), 91.0 (10), 75.0 (62), 55.0 (60).
IR (FT-ATR) $\tilde{\nu}$ [cm^{-1}]: 2994 (w), 2943 (m), 2893 (w), 2866 (m), 1651 (vw), 1618 (w), 1599 (w), 1464 (w), 1409 (vw), 1382 (m), 1370 (m), 1349 (w), 1328 (vw), 1292 (w), 1266 (m), 1258 (m), 1233 (w), 1218 (w), 1202 (m), 1188 (m), 1169 (m), 1143 (w), 1093 (vs), 1056 (s), 1030 (m), 1014 (m), 997 (m), 980 (m), 944 (w), 917 (m), 903 (m), 879 (s), 863 (m), 813 (s), 781 (m), 762 (m), 731 (w), 718 (w), 678 (s), 656 (m).

(((4a*R*,8*R*,8a*R*)-6-(3,5-Dichlorophenyl)-2,2-dimethyl-4,4a,8,8a-tetrahydropyrano[3,2-*d*][1,3]dioxin-8-yl)oxy)triisopropylsilane (7g)

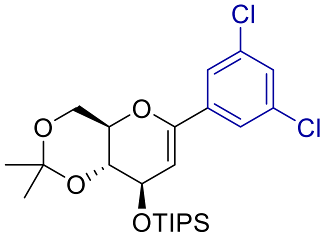

According to GP-B, using *t*BuLi (0.22 mmol, 1.1 equiv.) and stirring for 60 min at -30°C for lithiation and employing 2-(3,5-dichlorophenyl)-4,4,5,5-tetramethyl-1,3,2-dioxaborolane (63 mg, 0.23 mmol, 1.15 equiv.). Flash column chromatography (SiO_2 – pentane/ EtOAc 39:1 to 7:3) furnished the title compound as a colorless oil (70 mg, 0.14 mmol, 72 %).

^{1}H-NMR: ($CDCl_3$, 400 MHz, 300 K) δ = 7.39 (d, *J* = 1.9 Hz, 2H), 7.29 (t, *J* = 1.9 Hz, 1H), 5.24 (d, *J* = 2.3 Hz, 1H), 4.59 – 4.55 (m, 1H), 4.11 – 4.05 (m, 1H), 3.97 – 3.83 (m, 3H), 1.53 (s, 3H), 1.43 (s, 3H), 1.11 (dq, *J* = 5.8, 3.4 Hz, 21H).
^{13}C-NMR: ($CDCl_3$, 101 MHz, 300 K) δ = 149.15, 137.28, 135.02, 128.56, 123.64, 103.79, 99.79, 73.10, 70.19, 68.57, 61.91, 29.09, 19.03, 18.21, 18.13, 12.46.

LR-MS (70 eV): m/z [%] = 443.1 (78), 411.1 (7), 385.0 (55), 329.0 (58), 303.0 (8), 284.9 (7), 260.9 (11), 242.9 (18), 224.9 (26), 198.9 (5), 172.9 (100), 145.0 (28), 127.0 (13), 103.0 (50), 75.0 (85), 55.0 (11).
HR-MS (EI pos): m/z: calcd. for ($[C_{21}H_{29}Cl_2O_4Si]^+$, $[M-Me]^+$): 471.1525, found: 471.1549.
IR (FT-ATR) $\tilde{\nu}$ [cm^{-1}]: 2994 (w), 2942 (m), 2925 (m), 2866 (m), 1987 (w), 1644 (w), 1588 (w), 1562 (m), 1463 (m), 1440 (w), 1416 (m), 1383 (m), 1370 (m), 1333 (m), 1288 (w), 1270 (m), 1260 (m), 1243 (w), 1218 (m), 1200 (m), 1169 (m), 1125 (s), 1117 (s), 1100 (vs), 1057 (s), 1032 (m), 1015 (m), 996 (m), 944 (m), 922 (w), 902 (m), 882 (s), 859 (s), 815 (s), 801 (s), 787 (m), 762 (m), 744 (w), 680 (s), 652 (s).

(((4a*R*,8*R*,8a*R*)-6-(4-Fluorophenyl)-2,2-dimethyl-4,4a,8,8a-tetrahydropyrano[3,2-*d*][1,3]dioxin-8-yl)oxy)triisopropylsilane (7h)

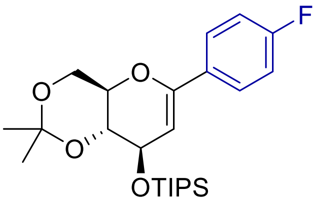

According to GP-B, using *t*BuLi (0.22 mmol, 1.1 equiv.) and stirring for 60 min at -30°C for lithiation and employing 4-fluorophenylboronic acid pinacol ester (51 mg, 0.23 mmol, 1.15 equiv.). Flash column chromatography (SiO_2 – pentane/ EtOAc 39:1) furnished the title compound as a colorless oil (54 mg, 0.12 mmol, 61 %).

^{1}H-NMR: ($CDCl_3$, 400 MHz, 300 K) δ = 7.54 – 7.48 (m, 2H), 7.06 – 6.95 (m, 2H), 5.16 (d, *J* = 2.3 Hz, 1H), 4.57 (dd, *J* = 6.9, 2.3 Hz, 1H), 4.07 (dd, *J* = 10.5, 5.0 Hz, 1H), 3.94 (t, *J* = 9.8 Hz, 1H), 3.92 – 3.81 (m, 2H), 1.54 (s, 3H), 1.43 (s, 3H), 1.18 – 1.05 (m, 21H).
^{13}C-NMR: δ = ($CDCl_3$, 101 MHz, 300 K) δ = 163.2 (d, *J* = 248.1 Hz), 150.8, 130.6 (d, *J* = 3.2 Hz), 127.1 (d, *J* = 8.2 Hz), 115.3 (d, *J* = 21.7 Hz), 101.5 (d, J = 1.6 Hz), 99.7, 73.4, 70.0, 68.7, 62.1, 29.1, 19.1, 18.2, 18.1, 12.5. 101.51.
^{19}F-NMR: ($CDCl_3$, 376 MHz, 300 K) δ = -113.0.

LR-MS (70 eV): m/z [%] = 393.1 (55), 361.0 (6), 335.1 (29), 307.0 (9), 279.1 (18), 253.0 (7), 211.0 (4), 192.9 (9), 174.9 (15), 146.0 (8), 123.0 (100), 102.9 (17), 75.0 (29), 59.0 (11).
HR-MS (EI pos): m/z calcd. for ($[C_{24}H_{37}FO_4Si]^+$, $[M^+]$): 436.2445, found: 436.2460.
IR (FT-ATR) $\tilde{\nu}$ [cm^{-1}]: 2994 (w), 2942 (m), 2894 (w), 2866 (m), 1647 (w), 1606 (w), 1510 (s), 1464 (m), 1411 (w), 1383 (m), 1370 (m), 1337 (m), 1297 (w), 1280 (m), 1270 (m), 1258 (m), 1234 (m), 1218 (m), 1200 (m), 1170 (m), 1159 (m), 1099 (vs), 1057 (s), 1032 (m), 1014 (m), 997 (m), 970 (w), 943 (m), 921 (w), 888 (s), 861 (m), 840 (s), 818 (m), 800 (s), 767 (s), 731 (w), 718 (w), 679 (s), 658 (m).

3-((4a*R*,8*R*,8a*R*)-2,2-Dimethyl-8-((triisopropylsilyl)oxy)-4,4a,8,8a-tetrahydropyrano[3,2-*d*][1,3]dioxin-6-yl)benzonitrile (7i)

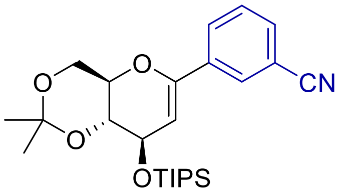

According to GP-B, using *t*BuLi (0.22 mmol, 1.1 equiv.) and stirring for 60 min at -30°C for lithiation and employing 3-cyanophenylboronic acid pinacol ester (53 mg, 0.23 mmol, 1.15 equiv.). Flash column chromatography (SiO_2 – pentane/ EtOAc 99:1 to 97:3) furnished the title compound as a colorless oil (55 mg, 0.12 mmol, 62 %).

^{1}H-NMR: ($CDCl_3$, 400 MHz, 300 K) δ = 7.82 (t, *J* = 1.7 Hz, 1H), 7.75 (dt, *J* = 8.0, 1.5 Hz, 1H), 7.58 (dt, *J* = 7.7, 1.5 Hz, 1H), 7.44 (t, *J* = 7.9 Hz, 1H), 5.30 (d, *J* = 2.3 Hz, 1H), 4.61 – 4.57 (m, 1H), 4.11 – 4.07 (m, 1H), 3.99 – 3.87 (m, 3H), 1.54 (s, 3H), 1.43 (s, 3H), 1.16 – 1.06 (m, 21H).
^{13}C-NMR: δ = ($CDCl_3$, 101 MHz, 300 K) δ = 149.48, 135.56, 132.03, 129.26, 128.82, 118.84, 112.65, 103.60, 99.82, 73.13, 70.19, 68.55, 61.92, 29.09, 19.04, 18.20, 18.11, 12.45.

LR-MS (70 eV): m/z [%] = 400.2 (100), 368.1 (8), 342.1 (75), 314.1 (13), 286.1 (68), 270.0 (10), 242.0 (7), 225.9 (5), 207.0 (49), 182.0 (30), 153.0 (6), 130.0 (100), 103.0 (56), 75.0 (91), 59.0 (45).
HR-MS (EI pos): m/z calcd. for ($[C_{24}H_{34}NO_4Si]^+$, $[M-Me^+]$): 428.2257, found: 428.2268.
IR (FT-ATR) $\tilde{\nu}$ [cm^{-1}]: 2993 (w), 2943 (m), 2892 (w), 2866 (m), 2232 (w), 1646 (w), 1480 (w), 1464 (w), 1431 (w), 1418 (w), 1383 (m), 1370 (m), 1339 (w), 1295 (w), 1272 (w), 1259 (m), 1218 (m), 1201 (m), 1171 (m), 1151 (w), 1115 (s), 1101 (vs), 1057 (s), 1032 (m), 1011 (m), 997 (m), 944 (m), 921 (w), 902 (s), 882 (s), 864 (s), 825 (s), 796 (s), 764 (s), 737 (w), 681 (s), 656 (m).

(((4a*R*,8*R*,8a*R*)-2,2-Dimethyl-6-(3-nitrophenyl)-4,4a,8,8a-tetrahydropyrano[3,2-*d*][1,3]dioxin-8-yl)oxy)triisopropylsilane (7j)

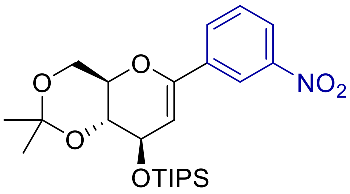

According to GP-B, using *t*BuLi (0.26 mmol, 1.3 equiv.) and stirring for 30 min at -30°C for lithiation and employing 3-nitrophenylboronic acid pinacol ester (57 mg, 0.23 mmol, 1.15 equiv.). Flash column chromatography (SiO_2 – pentane/ EtOAc 99:1 to 19:1) furnished the title compound as a yellow oil (65 mg, 0.14 mmol, 68 %).

^{1}H-NMR: ($CDCl_3$, 400 MHz, 300 K) δ = 8.37 (t, *J* = 2.0 Hz, 1H), 8.15 (ddd, *J* = 8.2, 2.3, 1.0 Hz, 1H), 7.84 (dt, *J* = 8.0, 1.4 Hz, 1H), 7.50 (t, *J* = 8.0 Hz, 1H), 5.36 (d, *J* = 2.3 Hz, 1H), 4.61 (dt, *J* = 5.9, 2.1 Hz, 1H), 4.15 – 4.08 (m, 1H), 4.00 – 3.89 (m, 3H), 1.55 (s, 3H), 1.44 (s, 3H), 1.17 – 1.09 (m, 21H).
^{13}C-NMR: (101 MHz, $CDCl_3$) δ = 149.38, 148.47, 136.08, 130.86, 129.30, 123.40, 120.13, 103.94, 99.82, 73.11, 70.26, 68.57, 61.93, 29.08, 22.76, 19.03, 18.20, 18.11, 12.46.

LR-MS (70 eV): m/z [%] = 421.2 (26), 420.2 (100), 363.1 (21), 362.1 (80), 306.1 (35), 150.0 (11).
HR-MS (EI pos): m/z: calcd. for ($[C_{23}H_{34}NO_6Si]^+$, $[M\text{-}iPr]^+$): 448.2155, found: 448.2147.
IR (FT-ATR) $\tilde{\nu}$ [cm^{-1}]: 2993 (w), 2942 (m), 2924 (m), 2866 (m), 1646 (w), 1532 (s), 1464 (m), 1440 (w), 1383 (m), 1370 (m), 1349 (s), 1312 (w), 1294 (w), 1260 (m), 1241 (w), 1218 (m), 1200 (m), 1169 (m), 1105 (vs), 1090 (s), 1057 (s), 1031 (m), 1014 (m), 998 (m), 945 (m), 924 (w), 905 (s), 882 (s), 868 (s), 853 (m), 805 (s), 774 (s), 761 (m), 741 (s), 714 (w), 679 (vs), 659 (m).

(((4a*R*,8*R*,8a*R*)-6-(Benzo[*d*][1,3]dioxol-4-yl)-2,2-dimethyl-4,4a,8,8a-tetrahydropyrano[3,2-*d*][1,3]dioxin-8-yl)oxy)triisopropylsilane (7k)

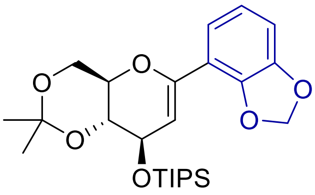

According to GP-B, using *t*BuLi (0.26 mmol, 1.3 equiv.) and stirring for 30 min at -30°C for lithiation and employing 2,3-methylenedioxyphenylboronic acid pinacol ester (57 mg, 0.23 mmol, 1.15 equiv.). Flash column chromatography (SiO_2 – pentane/ EtOAc 99:1 to 19:1) furnished the title compound as a colorless oil (26 mg, 0.06 mmol, 28 %).

^{1}H-NMR: ($CDCl_3$, 400 MHz, 300 K) δ = 7.06 (dd, *J* = 8.2, 1.7 Hz, 1H), 7.00 (d, *J* = 1.7 Hz, 1H), 6.76 (d, *J* = 8.2 Hz, 1H), 5.96 (q, *J* = 1.4 Hz, 2H), 5.09 (d, *J* = 2.2 Hz, 1H), 4.56 (dd, *J* = 7.0, 2.2 Hz, 1H), 4.06 (dd, *J* = 10.8, 5.4 Hz, 1H), 3.95 – 3.83 (m, 3H), 1.53 (s, 3H), 1.43 (s, 3H), 1.14 – 1.08 (m, 21H).

^{13}C-NMR: ($CDCl_3$, 101 MHz, 300 K) δ = 151.28, 148.11, 147.73, 128.74, 119.33, 108.12, 105.89, 101.33, 100.68, 99.67, 73.45, 69.94, 68.79, 62.10, 29.15, 19.10, 18.20, 18.12, 12.49.

HR-MS (EI pos): m/z calcd. for ($[C_{25}H_{38}O_6Si]^+$, $[M]^+$): 462.2438, found: 462.2432.

IR (FT-ATR) $\tilde{\nu}$ [cm^{-1}]: 2994 (w), 2941 (m), 2926 (m), 2892 (m), 2866 (m), 1649 (w), 1643 (w), 1611 (w), 1607 (w), 1504 (m), 1491 (m), 1462 (m), 1445 (m), 1413 (w), 1383 (m), 1370 (m), 1359 (m), 1322 (m), 1289 (m), 1274 (m), 1249 (s), 1215 (s), 1200 (m), 1170 (m), 1140 (m), 1103 (vs), 1087 (s), 1056 (s), 1040 (s), 1032 (s), 1015 (m), 996 (m), 941 (s), 924 (m), 908 (s), 882 (s), 870 (vs), 852 (m), 804 (s), 766 (s), 739 (w), 722 (w), 704 (w), 678 (s), 664 (m), 659 (m).

(*E*)-1-(4-((4a*R*,8*R*,8a*R*)-2,2-Dimethyl-8-((triisopropylsilyl)oxy)-4,4a,8,8a-tetrahydropyrano[3,2-*d*][1,3]dioxin-6-yl)phenyl)-2-phenyldiazene (7l)

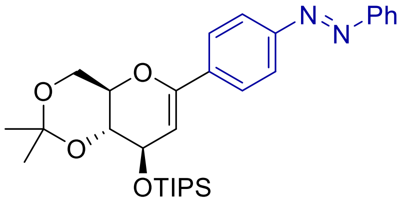

According to GP-B on a 0.1 mmol scale, using *t*BuLi (0.11 mmol, 1.1 equiv.) and stirring for 60 min at -30°C for lithiation and employing (*E*)-1-phenyl-2-(4-(4,4,5,5-tetramethyl-1,3,2-dioxaborolan-2-yl)phenyl)diazene (35.4 mg, 0.115 mmol, 1.15 equiv.). Flash column chromatography (SiO_2 – pentane/ EtOAc 99:1 to 95:5) furnished the title compound as a red oil (36 mg, 0.07 mmol, 68 %).

^{1}H-NMR: (500 MHz, $CDCl_3$) δ = 7.94 – 7.88 (m, 4H), 7.72 – 7.67 (m, 2H), 7.55 – 7.47 (m, 3H), 5.37 (d, *J* = 2.3 Hz, 1H), 4.62 (dd, *J* = 6.9, 2.3 Hz, 1H), 4.11 (dd, *J* = 10.6, 5.0 Hz, 1H), 4.02 – 3.90 (m, 3H), 1.56 (s, 3H), 1.45 (s, 3H), 1.17 – 1.10 (m, 21H).
^{13}C-NMR: (126 MHz, $CDCl_3$) δ = 175.28, 174.58, 164.44, 162.45, 148.20, 132.34, 129.11 (d, *J* = 8.5 Hz), 128.05 (d, *J* = 3.5 Hz), 127.69 (d, *J* = 8.5 Hz), 126.28 (d, *J* = 2.7 Hz), 115.96 (d, *J* = 22.2 Hz), 62.87, 60.52, 55.54, 51.60, 49.58, 47.35, 43.71, 25.00, 21.16.

HR-MS (ESI): m/z calcd. for ($[C_{30}H_{42}N_2O_4Si]^+$, $[H^+]$): 523.2994, found: 523.299.

(((4a*R*,8*R*,8a*R*)-6-(4-Chloro-3-(4-(((S)-tetrahydrofuran-3-yl)oxy)benzyl)phenyl)-2,2-dimethyl-4,4a,8,8a-tetrahydropyrano[3,2-*d*][1,3]dioxin-8-yl)oxy)triisopropylsilane (7m)

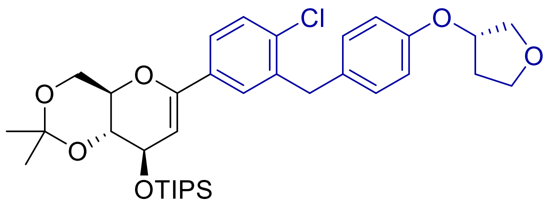

According to GP-B on a 0.1 mmol scale, using *t*BuLi (0.11 mmol, 1.1 equiv.) and stirring for 60 min at -30°C for lithiation and employing (*S*)-2-(4-chloro-3-(4-((tetrahydrofuran-3-yl)oxy)benzyl)phenyl)-4,4,5,5-tetramethyl-1,3,2-dioxaborolane (48 mg, 0.115 mmol, 1.15 equiv.). Flash column chromatography (SiO_2 – pentane/ EtOAc 99:1 to 9:1) furnished the title compound as a colorless oil (47 mg, 0.07 mmol, 74 %).

^{1}H-NMR: (500 MHz, $CDCl_3$) δ = 7.32 (s, 3H), 7.08 (d, *J* = 8.5 Hz, 2H), 6.81 – 6.76 (m, 2H), 5.14 (d, *J* = 2.3 Hz, 1H), 4.91 – 4.86 (m, 1H), 4.55 (dd, *J* = 6.9, 2.3 Hz, 1H), 4.07 – 3.81 (m, 10H), 2.20 – 2.13 (m, 2H), 1.53 (s, 3H), 1.43 (s, 3H), 1.17 – 1.06 (m, 21H).
^{13}C-NMR: (126 MHz, $CDCl_3$) δ = 156.04, 150.68, 138.80, 134.59, 133.14, 131.73, 130.03, 129.48, 127.64, 124.43, 115.46, 102.09, 99.70, 77.38, 73.27, 70.01, 68.68, 67.33, 62.01, 38.51, 33.16, 29.10, 19.07, 18.17, 18.09, 12.45.

HR-MS (ESI): m/z calcd. for ($[C_{35}H_{49}ClO_6Si]^+$, $[H^+]$): 629.3060, found: 629.306.

D-Glucal Series

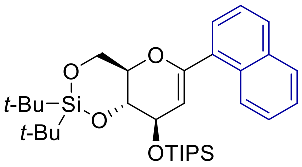

8a (84%)

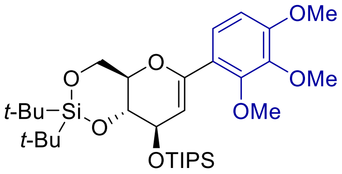

8b (92%)

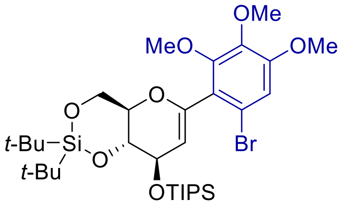

8c (68%)

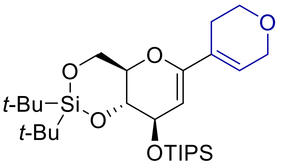

8d (77%)

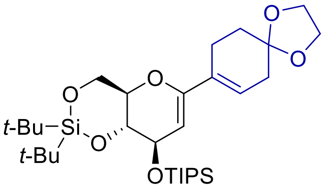

8e (89%)

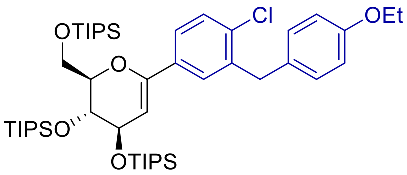

9a (51%)

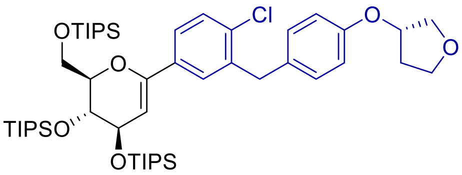

9b (57%)

(4a*R*,8*R*,8a*R*)-8-((l1-silyl)oxy)-2,2-Di-tert-butyl-6-(naphthalen-1-yl)-4,4a,8,8a-tetrahydropyrano[3,2-*d*][1,3,2]dioxasiline (8a)

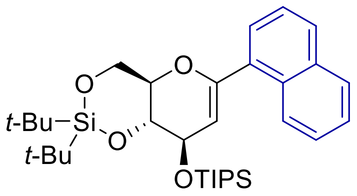

According to GP-B on a 0.1 mmol scale, using *t*BuLi (0.13 mmol, 1.3 equiv.) and stirring for 30 min at -30°C for lithiation and employing 4,4,5,5-tetramethyl-2-(naphthalen-1-yl)-1,3,2-dioxaborolane (29 mg, 0.115 mmol, 1.15 equiv.). Flash column chromatography (SiO_2 – pentane/ EtOAc 999:1 to 98:2) furnished the title compound as a colorless oil, which solidified (48 mg, 0.08 mmol, 84 %).

^{1}H-NMR: (500 MHz, $CDCl_3$) δ = 8.15 (dd, *J* = 8.1, 1.7 Hz, 1H), 7.86 – 7.82 (m, 2H), 7.55 – 7.41 (m, 4H), 5.05 (d, *J* = 2.1 Hz, 1H), 4.66 (dd, *J* = 6.8, 2.1 Hz, 1H), 4.30 – 4.23 (m, 2H), 4.19 – 4.11 (m, 2H), 1.16 – 1.11 (m, 30H), 1.06 (s, 9H).
Note: ^{1}H-NMR shows trace of EtOAc.
^{13}C-NMR: (126 MHz, $CDCl_3$) δ = 152.15, 133.81, 133.12, 131.30, 129.53, 128.43, 127.07, 126.43, 126.04, 125.78, 125.23, 106.51, 77.86, 73.58, 71.96, 66.31, 27.67, 27.17, 22.97, 20.07, 18.33, 14.36, 12.64.

HR-MS (ESI): m/z calcd. for ([$C_{33}H_{52}O_4Si_2$]$^+$, [M^+]): 569.3484, found: 569.348.

(4a*R*,8*R*,8a*R*)-8-((l1-Silyl)oxy)-2,2-di-*tert*-butyl-6-(2,3,4-trimethoxyphenyl)-4,4a,8,8a-tetrahydropyrano[3,2-*d*][1,3,2]dioxasiline (8b)

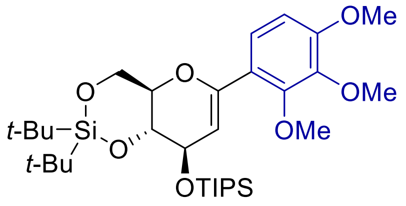

According to GP-B on a 4.3 mmol scale, using *t*BuLi (5.6 mmol, 1.3 equiv.) and stirring for 45 min at -30°C for lithiation and employing 4,4,5,5-tetramethyl-2-(2,3,4-trimethoxyphenyl)-1,3,2-dioxaborolane (1.45 g, 4.95 mmol, 1.15 equiv.). Flash column chromatography (SiO_2 – pentane/ EtOAc 98:2 to 88:12) furnished the title compound as a colorless oil (2.4 g, 3.9 mmol, 92 %).

^{1}H-NMR: (500 MHz, $CDCl_3$) δ = 7.13 (d, *J* = 8.7 Hz, 1H), 6.63 (d, *J* = 8.7 Hz, 1H), 5.31 (s, 1H), 4.58 (d, *J* = 6.8 Hz, 1H), 4.26 (dd, *J* = 10.4, 4.7 Hz, 1H), 4.13 – 4.04 (m, 2H), 4.01 – 3.96 (m, 1H), 3.86 (s, 9H), 1.17 – 1.10 (m, 21H), 1.08 (s, 9H), 1.01 (s, 9H).
Note: ^{1}H-NMR shows trace of aromatic impurity.
^{13}C-NMR: (126 MHz, $CDCl_3$) δ = 154.20, 152.19, 148.65, 142.70, 123.54, 121.85, 107.03, 104.80, 77.74, 73.11, 72.10, 66.29, 61.05, 61.02, 56.12, 27.63, 27.12, 22.92, 20.03, 18.33, 12.63.

HR-MS (ESI): m/z calcd. for ([$C_{32}H_{56}O_7Si_2$]$^+$, [H^+]): 609.3645, found: 609.364.

(4a*R*,8*R*,8a*R*)-6-(6-Bromo-2,3,4-trimethoxyphenyl)-2,2-di-*tert*-butyl-8-((triisopropylsilyl)oxy)-4,4a,8,8a-tetrahydropyrano[3,2-*d*][1,3,2]dioxasiline (8c)

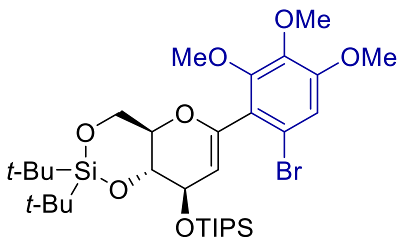

According to GP-B on a 0.1 mmol scale, using *t*BuLi (0.13 mmol, 1.3 equiv.) and stirring for 30 min at -30°C for lithiation and employing 2-(6-bromo-2,3,4-trimethoxyphenyl)-4,4,5,5-tetramethyl-1,3,2-dioxaborolane (43 mg, 0.115 mmol, 1.15 equiv.). Flash column chromatography (SiO_2 – pentane/ EtOAc 98:2 to 91:9) furnished the title compound as a colorless oil (47 mg, 0.07 mmol, 68 %).

^{1}H-NMR (500 MHz, $CDCl_3$) δ = 6.85 (s, 1H), 4.90 (d, *J* = 10.1 Hz, 1H), 4.30 (t, *J* = 9.3 Hz, 1H), 4.19 (dd, *J* = 10.4, 5.3 Hz, 1H), 4.15 (s, 3H), 4.01 – 3.94 (m, 2H), 3.82 (s, 3H), 3.72 (s, 3H), 3.68 (dd, *J* = 9.9, 5.1 Hz, 1H), 1.34 – 1.31 (m, 3H), 1.19 (d, *J* = 7.5 Hz, 18H), 1.08 (s, 9H), 1.01 (s, 9H).
^{13}C-NMR (126 MHz, $CDCl_3$) δ = 153.28, 152.18, 139.33, 126.33, 115.61, 114.73, 78.71, 73.07, 66.74, 62.40, 60.53, 56.31, 37.30, 27.93, 27.15, 23.27, 20.17, 19.12, 18.82, 14.30.

HR-MS (ESI): m/z calcd. for ([$C_{32}H_{56}BrO_7Si_2$]$^+$, [H^+]): 687.2750, found: 687.275.

(4a*R*,8*R*,8a*R*)-8-((l1-Silyl)oxy)-2,2-di-*tert*-butyl-6-(3,6-dihydro-2*H*-pyran-4-yl)-4,4a,8,8a-tetrahydropyrano[3,2-*d*][1,3,2]dioxasiline (8d)

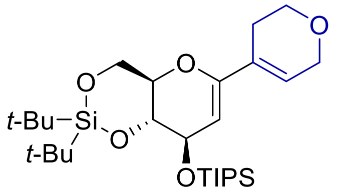

According to GP-B on a 0.1 mmol scale, using *t*BuLi (0.13 mmol, 1.3 equiv.) and stirring for 30 min at -30°C for lithiation and employing 2-(3,6-dihydro-2*H*-pyran-4-yl)-4,4,5,5-tetramethyl-1,3,2-dioxaborolane (24.1 mg, 0.115 mmol, 1.15 equiv.). Flash column chromatography (SiO_2 – pentane/ EtOAc 96:4) furnished the title compound as a colorless oil (40 mg, 0.08 mmol, 77 %).

^{1}H-NMR: (500 MHz, $CDCl_3$) δ = 6.11 (d, *J* = 3.1 Hz, 1H), 4.74 (d, *J* = 2.4 Hz, 1H), 4.50 (dd, *J* = 7.0, 2.4 Hz, 1H), 4.25 – 4.20 (m, 3H), 4.06 – 3.98 (m, 2H), 3.87 – 3.74 (m, 3H), 1.30 (d, *J* = 7.5 Hz, 2H), 1.14 – 1.09 (m, 21H), 1.06 (s, 9H), 0.99 (s, 9H).
^{13}C-NMR: (126 MHz, $CDCl_3$) δ = 150.33, 127.94, 123.62, 101.13, 77.72, 72.76, 71.87, 66.25, 65.63, 64.18, 27.64, 27.59, 27.08, 25.40, 25.21, 24.69, 22.90, 20.01, 18.31, 18.28, 12.61.

HR-MS (ESI): m/z calcd. for ($[C_{28}H_{52}O_5Si_2]^+$, $[H^+]$): 525.3433, found: 525.342.

(4a*R*,8*R*,8a*R*)-8-((l1-Silyl)oxy)-2,2-di-*tert*-butyl-6-(1,4-dioxaspiro[4.5]dec-7-en-8-yl)-4,4a,8,8a-tetrahydropyrano[3,2-*d*][1,3,2]dioxasiline (8e)

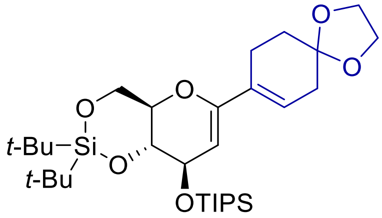

According to GP-B on a 0.1 mmol scale, using *t*BuLi (0.13 mmol, 1.3 equiv.) and stirring for 30 min at -30°C for lithiation and employing 4,4,5,5-tetramethyl-2-(1,4-dioxaspiro[4.5]dec-7-en-8-yl)-1,3,2-dioxaborolane (31 mg, 0.115 mmol, 1.15 equiv.). Flash column chromatography (SiO_2 – pentane/ EtOAc 93:7) furnished the title compound as a colorless oil (52 mg, 0.09 mmol, 89 %).

^{1}H-NMR: (500 MHz, $CDCl_3$) δ = 6.09 – 6.06 (m, 1H), 4.77 (d, *J* = 2.4 Hz, 1H), 4.47 (dd, *J* = 7.0, 2.4 Hz, 1H), 4.20 (dd, *J* = 10.3, 5.0 Hz, 1H), 4.03 – 3.95 (m, 6H), 3.80 (td, *J* = 10.3, 5.0 Hz, 1H), 2.36 (d, *J* = 4.6 Hz, 4H), 1.30 – 1.24 (m, 2H), 1.14 – 1.08 (m, 21H), 1.05 (s, 9H), 0.98 (s, 9H).
Note: ^{1}H-NMR shows trace of EtOAc.
^{13}C-NMR: (126 MHz, $CDCl_3$) δ = 150.82, 129.67, 122.80, 107.88, 101.11, 77.71, 72.69, 71.99, 66.29, 64.60, 35.75, 31.09, 27.59, 27.08, 24.15, 22.89, 20.00, 18.31, 18.28, 12.60.

HR-MS (ESI): m/z calcd. for ($[C_{31}H_{56}O_6Si_2]^+$, $[H^+]$): 581.3695, found: 581.370.

(4a*R*,8*R*,8a*R*)-2,2-di-*tert*-butyl-6-(1,4-dioxaspiro[4.5]dec-7-en-8-yl)-8-((triisopropylsilyl)oxy)-4,4a,8,8a-tetrahydropyrano[3,2-*d*][1,3,2]dioxasiline (9a)

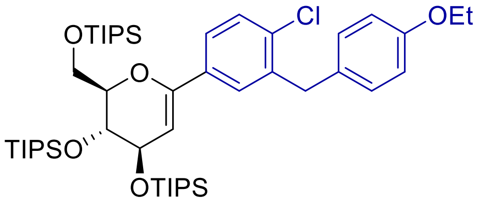

According to GP-B on a 0.5 mmol scale, using *t*BuLi (0.65 mmol, 1.3 equiv.,) and stirring for 60 min at -30°C for lithiation and employing 2-(4-chloro-3-(4-ethoxybenzyl)phenyl)-4,4,5,5-tetramethyl-1,3,2-dioxaborolane (214 mg, 0.575 mmol, 1.15 equiv.). Flash column chromatography (SiO_2 – pentane/ EtOAc 999:1 to 93:7) furnished the title compound as a colorless oil (0.22 g, 0.26 mmol, 51 %).

^{1}H-NMR: (500 MHz, $CDCl_3$) δ = 7.45 – 7.40 (m, 2H), 7.30 (d, *J* = 8.3 Hz, 1H), 7.11 – 7.07 (m, 2H), 6.82 – 6.79 (m, 2H), 5.26 (dd, *J* = 5.3, 1.5 Hz, 1H), 4.43 (ddt, *J* = 7.9, 3.9, 1.9 Hz, 1H), 4.14 – 4.07 (m, 3H), 4.03 – 3.98 (m, 4H), 3.84 (dd, *J* = 11.3, 3.9 Hz, 1H), 1.40 (t, *J* = 7.0 Hz, 3H), 1.12 – 0.98 (m, 63H).
^{13}C-NMR: (126 MHz, $CDCl_3$) δ = 157.52, 149.48, 138.61, 135.12, 134.11, 131.48, 129.96, 129.22, 127.99, 124.65, 114.52, 97.17, 81.54, 70.11, 66.73, 63.47, 62.07, 38.66, 18.33, 18.26, 18.20, 18.14, 18.11, 15.04, 12.68, 12.56, 12.15.

HR-MS (ESI): m/z calcd. for ([$C_{48}H_{83}ClO_5Si_3$]$^+$, [H^+]): 859.5317, found: 859.532.

(((2*R*,3*R*,4*R*)-2-(((l1-Silyl)oxy)methyl)-6-(4-chloro-3-(4-(((*S*)-tetrahydrofuran-3-yl)oxy)benzyl)phenyl)-3,4-dihydro-2*H*-pyran-3,4-diyl)bis(oxy))bis(l1-silane) (9b)

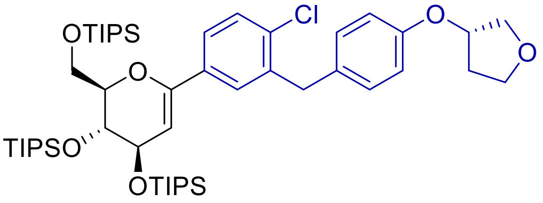

According to GP-B on a 0.5 mmol scale, using *t*BuLi (0.65 mmol, 1.3 equiv.,) and stirring for 60 min at -30°C for lithiation and employing (*S*)-2-(4-chloro-3-(4-((tetrahydrofuran-3-yl)oxy)benzyl)phenyl)-4,4,5,5-tetramethyl-1,3,2-dioxaborolane (238 mg, 0.575 mmol, 1.15 equiv.). Flash column chromatography (SiO_2 – pentane/ EtOAc 92:8) furnished the title compound as a colorless oil (0.26 g, 0.29 mmol, 57 %).

^{1}H-NMR: (500 MHz, $CDCl_3$) δ = 7.47 (d, *J* = 2.2 Hz, 1H), 7.42 (dd, *J* = 8.4, 2.2 Hz, 1H), 7.30 (d, *J* = 8.3 Hz, 1H), 7.12 – 7.08 (m, 2H), 6.79 – 6.75 (m, 2H), 5.27 (dd, *J* = 5.4, 1.5 Hz, 1H), 4.88 (tt, *J* = 4.9, 2.2 Hz, 1H), 4.44 (ddt, *J* = 7.8, 3.9, 1.9 Hz, 1H), 4.16 – 4.11 (m, 2H), 4.11 – 4.07 (m, 2H), 4.03 (d, *J* = 4.9 Hz, 2H), 4.00 – 3.95 (m, 3H), 3.91 – 3.87 (m, 1H), 3.84 (dd, *J* = 11.3, 3.9 Hz, 1H), 2.20 – 2.12 (m, 2H), 1.08 – 0.99 (m, 63H).

^{13}C-NMR: (126 MHz, $CDCl_3$) δ = 155.96, 149.44, 138.43, 135.17, 134.11, 132.04, 130.04, 129.26, 128.04, 124.72, 115.40, 97.20, 81.56, 77.39, 73.32, 70.11, 67.34, 66.71, 62.06, 38.64, 33.18, 18.33, 18.27, 18.25, 18.20, 18.14, 18.11, 12.68, 12.55, 12.15.

HR-MS (ESI): m/z calcd. for ($[C_{50}H_{85}ClO_6Si_3]^+$, $[H^+]$): 901.5422, found: 901.543.

L-Rhamnal Series

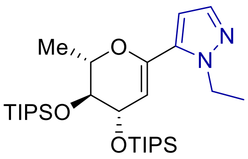

11a (68%)

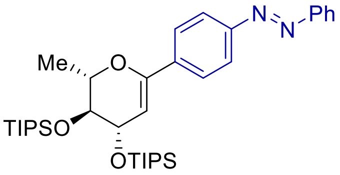

11b (57%)

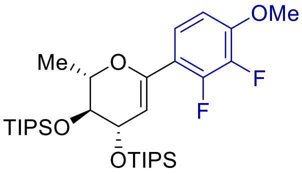

11c (53%)

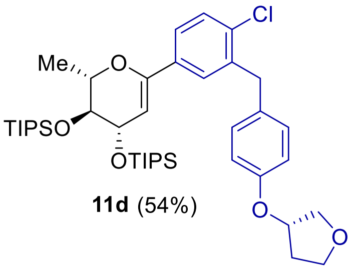

11d (54%)

1-Ethyl-5-((2*S*,3*S*,4*S*)-2-methyl-3,4-bis((triisopropylsilyl)oxy)-3,4-dihydro-2*H*-pyran-6-yl)-1*H*-pyrazole (11a)

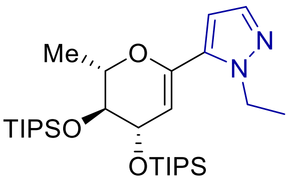

According to GP-B on a 0.1 mmol scale, using *t*BuLi (0.13 mmol, 1.3 equiv.) and stirring for 30 min at -30°C for lithiation and employing 1-ethyl-5-(4,4,5,5-tetramethyl-1,3,2-dioxaborolan-2-yl)-1*H*-pyrazole (26 mg, 0.115 mmol, 1.15 equiv.). Preparative TLC purification (SiO_2 – hexane/ EtOAc 99:1 -> 97:3) afforded the title compound as a pale yellow oil (36 mg, 0.07 mmol, 68 %).

^{1}H-NMR: (500 MHz, $CDCl_3$) δ = 7.40 (d, *J* = 1.9 Hz, 1H), 6.25 (d, *J* = 1.9 Hz, 1H), 5.19 (dd, *J* = 5.2, 1.5 Hz, 1H), 4.48 (qt, *J* = 7.0, 2.0 Hz, 1H), 4.35 – 4.23 (m, 2H), 4.18 (dt, *J* = 5.2, 2.0 Hz, 1H), 3.98 (q, *J* = 2.0 Hz, 1H), 1.47 (d, *J* = 7.2 Hz, 3H), 1.40 (d, *J* = 7.2 Hz, 3H), 1.09 – 1.05 (m, 42H).
^{13}C-NMR: (126 MHz, $CDCl_3$) δ = 142.77, 138.11, 106.04, 101.69, 75.79, 72.69, 66.76, 46.00, 18.29, 18.24, 18.23, 18.19, 16.17, 15.96, 12.61, 12.59.

HR-MS (ESI): m/z calcd. for ($[C_{29}H_{56}N_2O_3Si_2]^+$, $[H^+]$): 537.3909, found: 537.3909.

(*E*)-1-(4-((2*S*,3*S*,4*S*)-2-Methyl-3,4-bis((triisopropylsilyl)oxy)-3,4-dihydro-2*H*-pyran-6-yl)phenyl)-2-phenyldiazene (11b)

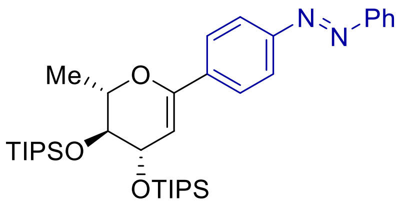

According to GP-B on a 0.1 mmol scale, using *t*BuLi (0.13 mmol, 1.3 equiv.) and stirring for 30 min at -30°C for lithiation and employing (*E*)-1-phenyl-2-(4-(4,4,5,5-tetramethyl-1,3,2-dioxaborolan-2-yl)phenyl)diazene (36 mg, 0.115 mmol, 1.15 equiv.). Preparative TLC purification (SiO_2 – hexane/ EtOAc 99:1) afforded the title compound as a pale red oil (35 mg, 0.06 mmol, 57 %).

^{1}H-NMR: (300 MHz, $CDCl_3$) δ = 7.92 (td, *J* = 6.3, 1.8 Hz, 4H), 7.76 (d, *J* = 8.3 Hz, 2H), 7.56 – 7.47 (m, 3H), 5.50 (d, *J* = 5.1 Hz, 1H), 4.53 (d, *J* = 7.3 Hz, 1H), 4.27 (d, *J* = 5.2 Hz, 1H), 4.05 – 3.99 (m, 1H), 1.47 (d, *J* = 7.0 Hz, 3H), 1.14 – 1.05 (m, 42H).
Note: ^{1}H-NMR shows trace of EtOAc.
^{13}C-NMR: (126 MHz, $CDCl_3$) δ = 152.92, 152.51, 149.14, 139.09, 131.05, 129.21, 125.99, 123.00, 122.86, 98.61, 75.49, 73.35, 67.61, 31.41, 18.36, 18.30, 18.28, 18.22, 16.19, 12.75, 12.71.

HR-MS (ESI): m/z calcd. for ($[C_{36}H_{58}N_2O_3Si_2]^+$, $[H^+]$): 623.4066, found: 623.406.

(((2*S*,3*S*,4*S*)-6-(2,3-Difluoro-4-methoxyphenyl)-2-methyl-3,4-dihydro-2*H*-pyran-3,4-diyl)bis(oxy))bis(triisopropylsilane) (11c)

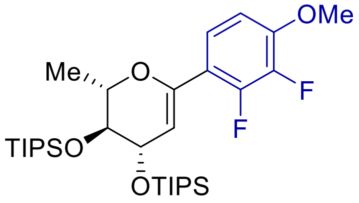

According to GP-B on a 0.1 mmol scale, using *t*BuLi (0.13 mmol, 1.3 equiv.) and stirring for 30 min at -30°C for lithiation and employing 2-(2,3-difluoro-4-methoxyphenyl)-4,4,5,5-tetramethyl-1,3,2-dioxaborolane (31 mg, 0.115 mmol, 1.15 equiv.). Flash column chromatography (SiO_2 – hexane/ EtOAc 99:1 -> 93:7) afforded the title compound as a colorless oil (31 mg, 0.05 mmol, 53 %).

^{1}H-NMR: (500 MHz, $CDCl_3$) δ = 7.29 – 7.26 (m, 1H), 6.71 (ddd, *J* = 9.2, 7.5, 1.9 Hz, 1H), 5.40 (d, *J* = 5.1 Hz, 1H), 4.45 (qt, *J* = 7.1, 2.1 Hz, 1H), 4.21 (dt, *J* = 4.9, 2.1 Hz, 1H), 3.99 – 3.96 (m, 1H), 3.90 (s, 3H), 1.45 (d, *J* = 7.1 Hz, 3H), 1.07 (dd, *J* = 9.1, 5.0 Hz, 42H).
^{13}C-NMR: (126 MHz, $CDCl_3$) δ = 144.32 (t, J = 3.1 Hz), 142.50, 122.29 (t, J = 4.0 Hz), 118.95 (d, J = 9.3 Hz), 107.66 (d, *J* = 3.4 Hz), 101.59 (d, *J* = 9.2 Hz), 100.52, 75.55, 73.24, 67.32, 56.75, 18.26 (t, *J* = 7.0 Hz), 16.08, 12.73, 12.63.
^{19}F-NMR: (471 MHz, $CDCl_3$) δ = -138.46, -138.50, -160.26, -160.30.

HR-MS (ESI): m/z calcd. for ($[C_{31}H_{54}F_2O_4Si_2]^+$, $[H^+]$): 585.3609, found: 585.3608.

(((2*S*,3*S*,4*S*)-6-(4-chloro-3-(4-(((*S*)-tetrahydrofuran-3-yl)oxy)benzyl)phenyl)-2-methyl-3,4-dihydro-2*H*-pyran-3,4-diyl)bis(oxy))bis(triisopropylsilane) (11d)

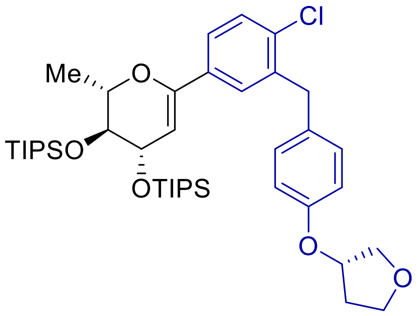

According to GP-B on a 1 mmol scale, using *t*BuLi (1.3 mmol, 1.3 equiv.) and stirring for 45 min at -30°C for lithiation and employing (*S*)-2-(4-chloro-3-(4-((tetrahydrofuran-3-yl)oxy)benzyl)phenyl)-4,4,5,5-tetramethyl-1,3,2-dioxaborolane (0.477 g, 1.15 mmol, 1.15 equiv.). Flash column chromatography (SiO_2 – hexane/ EtOAc 99:1 -> 9:1) afforded the title compound as a colorless oil (0.39 g, 0.54 mmol, 54 %).

^{1}H-NMR: (500 MHz, $CDCl_3$) δ = 7.41 – 7.37 (m, 2H), 7.33 – 7.29 (m, 1H), 7.13 – 7.09 (m, 2H), 6.81 – 6.76 (m, 2H), 5.27 (dd, *J* = 5.2, 1.4 Hz, 1H), 4.89 (ddt, *J* = 6.6, 4.8, 2.3 Hz, 1H), 4.45 (qt, *J* = 7.0, 2.0 Hz, 1H), 4.20 (dt, *J* = 5.2, 2.1 Hz, 1H), 4.04 (d, *J* = 3.2 Hz, 2H), 4.01 – 3.94 (m, 4H), 3.89 (td, *J* = 8.2, 4.5 Hz, 1H), 2.20 – 2.12 (m, 2H), 1.41 (d, *J* = 7.1 Hz, 3H), 1.09 – 1.03 (m, 42H).
Note: ^{1}H-NMR shows trace of EtOAc.
^{13}C-NMR: (126 MHz, $CDCl_3$) δ = 156.00, 148.91, 138.56, 135.38, 134.06, 131.99, 130.10, 129.36, 127.77, 124.57, 115.45, 97.46, 75.42, 73.31, 67.55, 67.35, 38.62, 33.17, 18.33, 18.27, 18.20, 16.19, 12.73, 12.68.

HR-MS (ESI): m/z calcd. for ($[C_{41}H_{65}ClO_5Si_2]^+$, $[H^+]$): 729.4139, found: 729.413.

D-Xylal Series

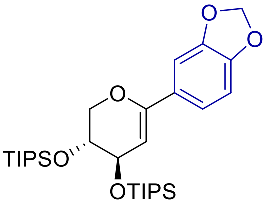

14a (52%)

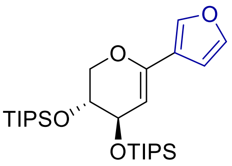

14b (42%)

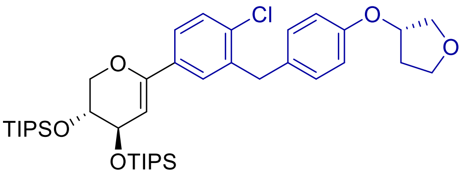

14c (49%)

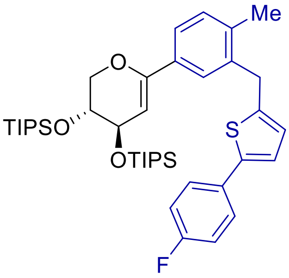

14d (53%)

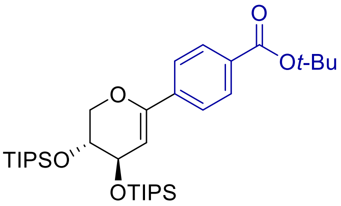

14e (36%)

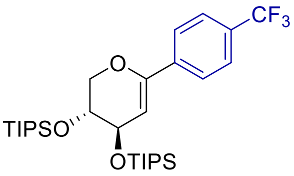

14f (48%)

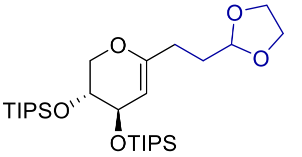

14g (53%)

(((3*R*,4*R*)-6-(Benzo[*d*][1,3]dioxol-5-yl)-3,4-dihydro-2*H*-pyran-3,4-diyl)bis(oxy))bis(triisopropylsilane) (14a)

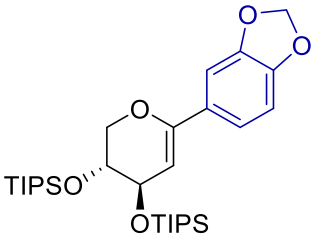

According to GP-B, using *t*BuLi (0.13 mmol, 1.3 equiv.) and stirring for 60 min at -30°C for lithiation and employing 2-(benzo[*d*][1,3]dioxol-5-yl)-4,4,5,5-tetramethyl-1,3,2-dioxaborolane (29 mg, 0.115 mmol, 1.15 equiv.). Flash column chromatography (SiO_2 – pentane/ EtOAc 999:1 to 98:2) furnished the title compound as a colorless oil (28 mg, 0.05 mmol, 52 %).

^{1}H-NMR: (500 MHz, $CDCl_3$) δ = 7.10 (dd, *J* = 8.2, 1.8 Hz, 1H), 7.05 (d, *J* = 1.8 Hz, 1H), 6.77 (d, *J* = 8.2 Hz, 1H), 5.95 (s, 2H), 5.22 (dd, *J* = 5.5, 1.5 Hz, 1H), 4.21 – 4.09 (m, 3H), 3.94 – 3.87 (m, 1H), 1.11 – 1.01 (m, 42H).
^{13}C-NMR: (126 MHz, $CDCl_3$) δ = 152.59, 147.79, 147.61, 130.65, 119.22, 108.06, 106.04, 101.19, 96.52, 69.34, 66.65, 65.80, 18.33, 18.26, 18.19, 18.14, 12.71, 12.60.

HR-MS (ESI): m/z calcd. for ($[C_{30}H_{52}O_5Si_2]^+$, $[H^+]$): 549.3433, found: 549.342.

(((3*R*,4*R*)-6-(Furan-3-yl)-3,4-dihydro-2*H*-pyran-3,4-diyl)bis(oxy))bis(triisopropylsilane) (14b)

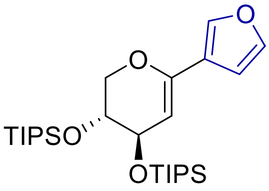

According to GP-B, using *t*BuLi (0.13 mmol, 1.3 equiv.) and stirring for 60 min at -30°C for lithiation and employing 2-(furan-3-yl)-4,4,5,5-tetramethyl-1,3,2-dioxaborolane (22 mg, 0.115 mmol, 1.15 equiv.). Preparative TLC purification (SiO_2 – pentane/ EtOAc 99:1 -> 98:2) furnished the title compound as a colorless oil (21 mg, 0.04 mmol, 42 %).

^{1}H-NMR: (500 MHz, $CDCl_3$) δ = 7.57 (d, *J* = 1.5 Hz, 1H), 7.34 (t, *J* = 1.8 Hz, 1H), 6.47 (d, *J* = 1.8 Hz, 1H), 5.10 (dd, *J* = 5.6, 1.5 Hz, 1H), 4.12 (q, *J* = 1.7 Hz, 1H), 4.09 (dd, *J* = 5.0, 2.5 Hz, 1H), 3.98 (d, *J* = 1.7 Hz, 1H), 3.90 (td, *J* = 2.5, 1.4 Hz, 1H), 1.05 (td, *J* = 8.3, 4.4 Hz, 42H).
^{13}C-NMR: (126 MHz, $CDCl_3$) δ = 143.01, 140.02, 107.76, 101.28, 97.05, 69.49, 66.33, 65.43, 18.31, 18.24, 18.17, 18.13, 12.68, 12.61.

HR-MS (ESI): m/z calcd. for ($[C_{27}H_{50}O_4Si_2]^+$, $[H^+]$): 495.3328, found: 495.332.

(((3*R*,4*R*)-6-(4-Chloro-3-(4-(((*S*)-tetrahydrofuran-3-yl)oxy)benzyl)phenyl)-3,4-dihydro-2*H*-pyran-3,4-diyl)bis(oxy))bis(triisopropylsilane) (14c)

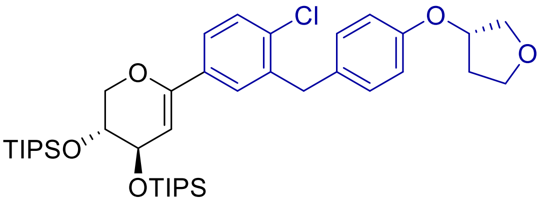

According to GP-B, using *t*BuLi (0.13 mmol, 1.3 equiv.) and stirring for 60 min at -30°C for lithiation and employing (*S*)-2-(4-chloro-3-(4-((tetrahydrofuran-3-yl)oxy)benzyl)phenyl)-4,4,5,5-tetramethyl-1,3,2-dioxaborolane (48 mg, 0.115 mmol, 1.15 equiv.). Flash column chromatography (SiO_2 – pentane/ EtOAc 93:7) furnished the title compound as a colorless oil (35 mg, 0.05 mmol, 49 %).

^{1}H-NMR: (500 MHz, $CDCl_3$) δ = 7.41 – 7.35 (m, 2H), 7.31 (d, *J* = 8.3 Hz, 1H), 7.11 (d, *J* = 8.6 Hz, 2H), 6.81 – 6.76 (m, 2H), 5.28 (dd, *J* = 5.5, 1.5 Hz, 1H), 4.88 (dp, *J* = 6.7, 2.3 Hz, 1H), 4.19 – 4.09 (m, 3H), 4.03 (s, 2H), 4.00 – 3.95 (m, 3H), 3.92 – 3.88 (m, 2H), 2.19 – 2.12 (m, 2H), 1.08 – 1.03 (m, 42H).
Note: ^{1}H-NMR shows trace of EtOAc.
^{13}C-NMR: (126 MHz, $CDCl_3$) δ = 155.99, 151.96, 138.62, 134.90, 134.11, 131.98, 130.10, 129.37, 127.71, 124.43, 115.45, 97.79, 73.30, 69.17, 67.34, 66.61, 65.55, 38.59, 33.17, 18.30, 18.22, 18.16, 18.11, 12.66, 12.58.

HR-MS (ESI): m/z calcd. for ($[C_{40}H_{63}ClO_5Si_2]^+$, $[H^+]$): 715.3983, found: 715.398.

(((3*R*,4*R*)-6-(4-Chloro-3-((5-(4-fluorophenyl)thiophen-2-yl)methyl)phenyl)-3,4-dihydro-2*H*-pyran-3,4-diyl)bis(oxy))bis(triisopropylsilane) (<u>14d</u>)

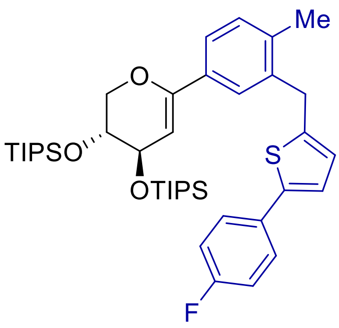

According to GP-B on a 0.1 mmol scale, using *t*BuLi (0.13 mmol, 1.3 equiv.) and stirring for 60 min at -30°C for lithiation and employing 2-(4-chloro-3-((5-(4-fluorophenyl)thiophen-2-yl)methyl)phenyl)-4,4,5,5-tetramethyl-1,3,2-dioxaborolane (49 mg, 0.115 mmol, 1.15 equiv.). Flash column chromatography (SiO_2 – pentane/ EtOAc 999:1 -> 95:5) furnished the title compound as a colorless oil (39 mg, 0.05 mmol, 53 %).

^{1}H-NMR: (500 MHz, $CDCl_3$) δ = 7.51 – 7.45 (m, 3H), 7.41 (dd, *J* = 7.9, 1.9 Hz, 1H), 7.14 (d, *J* = 7.9 Hz, 1H), 7.05 – 6.99 (m, 3H), 6.67 (d, *J* = 3.5 Hz, 1H), 5.32 (dd, *J* = 5.5, 1.4 Hz, 1H), 4.20 – 4.12 (m, 5H), 3.96 – 3.91 (m, 1H), 2.31 (s, 3H), 1.10 – 1.04 (m, 42H).
^{13}C-NMR: (126 MHz, $CDCl_3$) δ = 163.18, 161.22, 152.77, 143.55, 141.60, 137.91, 136.65, 134.25, 131.07 (d, *J* = 3.3 Hz), 130.38, 127.23 (d, *J* = 7.8 Hz), 126.41, 126.10, 123.80, 122.78, 115.81 (d, *J* = 21.7 Hz), 96.96, 69.39, 66.60, 65.81, 34.37, 19.43, 18.33, 18.25, 18.20, 18.15, 12.70, 12.62.
^{19}F-NMR: (471 MHz, $CDCl_3$) δ = -115.99.

HR-MS (ESI): m/z calcd. for ($[C_{41}H_{61}FO_3SSi_2]^+$, $[H^+]$): 709.3944, found: 709.394.

tert-butyl 4-((3R,4R)-3,4-bis((triisopropylsilyl)oxy)-3,4-dihydro-2H-pyran-6-yl)benzoate (14e)

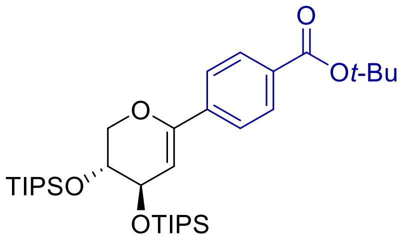

According to GP-B on a 0.1 mmol scale, using *t*BuLi (0.13 mmol, 1.3 equiv.) and stirring for 60 min at -30°C for lithiation and employing *tert*-butyl 4-(4,4,5,5-tetramethyl-1,3,2-dioxaborolan-2-yl)benzoate (37 mg, 0.115 mmol, 1.15 equiv.). Flash column chromatography (SiO_2 – pentane/ EtOAc 99:1 -> 93:7) furnished the title compound as a colorless oil (22 mg, 0.04 mmol, 36 %).

^{1}H-NMR: (500 MHz, $CDCl_3$) δ = 7.97 – 7.92 (m, 2H), 7.67 – 7.58 (m, 2H), 5.47 (dd, *J* = 5.5, 1.5 Hz, 1H), 4.26 – 4.20 (m, 1H), 4.19 – 4.13 (m, 2H), 3.95 – 3.93 (m, 1H), 1.59 (s, 9H), 1.10 – 1.02 (m, 42H).
^{13}C-NMR: (126 MHz, $CDCl_3$) δ = 165.75, 152.08, 139.89, 131.72, 129.41, 124.86, 99.09, 81.06, 69.18, 66.65, 65.52, 28.36, 18.31, 18.23, 18.16, 18.11, 12.68, 12.59.

HR-MS (ESI): m/z calcd. for ([$C_{34}H_{60}O_5Si_2$]$^+$, [H^+]): 605.4059, found: 605.405.

(((3*R*,4*R*)-6-(4-(trifluoromethyl)phenyl)-3,4-dihydro-2*H*-pyran-3,4-diyl)bis(oxy))bis(triisopropylsilane) (14f)

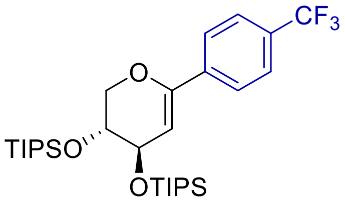

According to GP-B on a 0.1 mmol scale, using *t*BuLi (0.13 mmol, 1.3 equiv.) and stirring for 60 min at -30°C for lithiation and employing 4,4,5,5-tetramethyl-2-(4-(trifluoromethyl)phenyl)-1,3,2-dioxaborolane (33 mg, 0.115 mmol, 1.15 equiv.). Flash column chromatography (SiO_2 – pentane/ EtOAc 999:1 -> 98:2) furnished the title compound as a colorless oil (27 mg, 0.05 mmol, 48 %).

^{1}H-NMR: (500 MHz, $CDCl_3$) δ = 7.69 (d, *J* = 8.2 Hz, 2H), 7.58 (d, *J* = 8.2 Hz, 2H), 5.46 (dd, *J* = 5.6, 1.5 Hz, 1H), 4.26 – 4.22 (m, 1H), 4.20 – 4.13 (m, 2H), 3.96 – 3.93 (m, 1H), 1.10 – 1.03 (m, 42H).
^{13}C-NMR: (126 MHz, $CDCl_3$) δ = 151.66, 139.49 (d, J = 1.1 Hz), 130.21 (q, J = 32.1 Hz), 125.40, 125.23 (q, *J* = 3.8 Hz), 99.18, 69.13, 66.71, 65.40, 18.30, 18.23, 18.16, 18.11, 12.67, 12.57.
^{19}F NMR: (471 MHz, $CDCl_3$) δ = -62.57.

HR-MS (ESI): m/z calcd. for ($[C_{30}H_{51}F_3O_3Si_2]^+$, $[H^+]$): 573.3409, found: 573.348.

(((3*R*,4*R*)-6-(2-(1,3-Dioxolan-2-yl)ethyl)-3,4-dihydro-2H-pyran-3,4-diyl)bis(oxy))bis(triisopropylsilane) (<u>14g</u>)

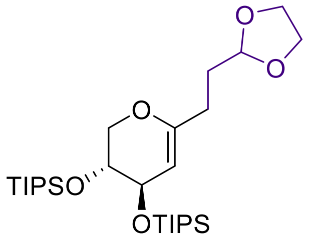

According to GP-B on a 0.1 mmol scale, using *t*BuLi (0.13 mmol, 1.3 equiv.) and stirring for 60 min at -30°C for lithiation and employing 2-(2-(1,3-dioxolan-2-yl)ethyl)-4,4,5,5-tetramethyl-1,3,2-dioxaborolane **<u>SI-12</u>** (27 mg, 0.12 mmol, 1.2 equiv.). Purification of the crude mixture by preparative TLC (SiO_2 – hexane/ EtOAc 98:2) furnished the title compound as a colorless oil (28 mg, 0.05 mmol, 53 %).

^{1}H-NMR: (500 MHz, $CDCl_3$) δ = 5.96 (dd, J = 9.9, 1.6 Hz, 1H), 5.82 (dd, J = 9.9, 2.7 Hz, 1H), 4.89 (t, J = 4.9 Hz, 1H), 4.62 (t, J = 7.4 Hz, 1H), 4.51 – 4.46 (m, 1H), 4.14 (dd, J = 9.9, 4.9 Hz, 1H), 3.99 – 3.96 (m, 2H), 3.86 – 3.84 (m, 2H), 3.71 – 3.60 (m, 2H), 2.57 – 2.49 (m, 2H), 1.08 – 1.05 (m, 42H).
^{13}C-NMR: (126 MHz, $CDCl_3$) δ = 149.68, 129.81, 124.40, 104.00, 103.42, 70.31, 65.12, 65.10, 63.70, 29.93, 18.11, 17.85, 12.44, 12.39.

HR-MS (ESI): m/z calcd. for ($[C_{28}H_{56}O_5Si_2]^+$, $[H^+]$): 529.3746, found: 529.3744.

L-Arabinal Series

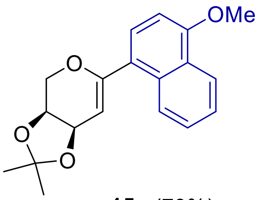

15a (79%)

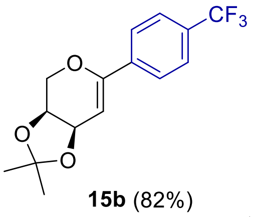

15b (82%)

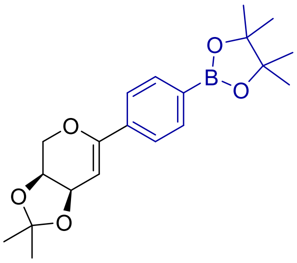

+

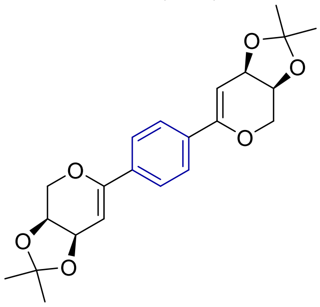

15c (51%) **15d** (36%)

(3a*S*,7a*R*)-6-(4-Methoxynaphthalen-1-yl)-2,2-dimethyl-3a,7a-dihydro-4*H*-[1,3]dioxolo[4,5-*c*]pyran (<u>15a</u>)

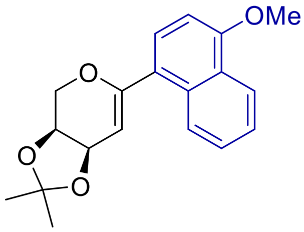

According to GP-B on a 0.1 mmol scale, using *t*BuLi (0.13 mmol, 1.3 equiv.) and stirring for 30 min at -30°C for lithiation and employing 2-(4-methoxynaphthalen-1-yl)-4,4,5,5-tetramethyl-1,3,2-dioxaborolane (33 mg, 0.115 mmol, 1.15 equiv.). Flash column chromatography (SiO_2 – pentane/ EtOAc 8:2) furnished the title compound as a colorless oil (25 mg, 0.05 mmol, 79 %).

^{1}H-NMR: (500 MHz, $CDCl_3$) δ = 8.28 (d, *J* = 8.3 Hz, 1H), 8.03 (d, *J* = 8.3 Hz, 1H), 7.54 – 7.43 (m, 3H), 6.77 (d, *J* = 7.9 Hz, 1H), 5.32 (dd, *J* = 4.1, 1.7 Hz, 1H), 4.76 (t, *J* = 5.0 Hz, 1H), 4.41 – 4.35 (m, 1H), 4.28 (dd, *J* = 11.0, 4.1 Hz, 1H), 4.01 (s, 3H), 4.00 – 3.94 (m, 1H), 1.58 (s, 3H), 1.47 (s, 3H).
^{13}C-NMR: (126 MHz, $CDCl_3$) δ = 157.62, 156.51, 132.24, 127.43, 126.97, 126.23, 125.71, 125.57, 125.40, 122.31, 109.17, 102.99, 100.11, 70.68, 69.04, 66.10, 55.71, 28.70, 26.33.

HR-MS (ESI): m/z calcd. for ($[C_{19}H_{20}O_4]^+$, $[H^+]$): 313.1442, found: 313.143.

(3a*S*,7a*R*)-2,2-Dimethyl-6-(4-(trifluoromethyl)phenyl)-3a,7a-dihydro-4*H*-[1,3]dioxolo[4,5-*c*]pyran (15b)

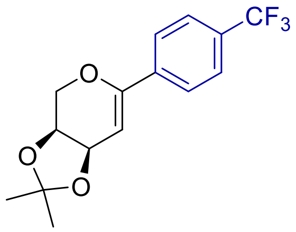

According to GP-B on a 0.1 mmol scale, using *t*BuLi (0.13 mmol, 1.3 equiv.) and stirring for 30 min at -30°C for lithiation and employing 4,4,5,5-tetramethyl-2-(4-(trifluoromethyl)phenyl)-1,3,2-dioxaborolane (33 mg, 0.115 mmol, 1.15 equiv.). Flash column chromatography (SiO_2 – pentane/ EtOAc 8:2) furnished the title compound as a colorless oil (25 mg, 0.08 mmol, 82 %).

^{1}H-NMR: (500 MHz, $CDCl_3$) δ = 7.71 (d, *J* = 8.3 Hz, 2H), 7.60 (d, *J* = 8.3 Hz, 2H), 5.67 (d, *J* = 4.3 Hz, 1H), 4.72 (dd, *J* = 5.9, 4.3 Hz, 1H), 4.32 (ddd, *J* = 7.7, 5.9, 3.9 Hz, 1H), 4.23 (dd, *J* = 11.1, 3.9 Hz, 1H), 3.90 (dd, *J* = 11.1, 7.7 Hz, 1H), 1.48 (s, 3H), 1.43 (s, 3H).

Note: ^{1}H-NMR shows trace of residual glycal.

^{13}C-NMR: (126 MHz, $CDCl_3$) δ = 154.19, 137.87 (q, *J* = 1.4 Hz), 125.59, 125.40 (q, *J* = 3.8 Hz, 109.49, 97.90, 70.64, 68.72, 66.25, 28.49, 26.27.

HR-MS (ESI): m/z calcd. for ($[C_{15}H_{15}F_3O_3]^+$, $[H^+]$): 301.1053, found: 301.104.

2-(4-((3a*S*,7a*R*)-2,2-Dimethyl-3a,7a-dihydro-4*H*-[1,3]dioxolo[4,5-*c*]pyran-6-yl)phenyl)-4,4,5,5-tetramethyl-1,3,2-dioxaborolane (15c)

1,4-bis((3a*S*,7a*R*)-2,2-Dimethyl-3a,7a-dihydro-4*H*-[1,3]dioxolo[4,5-*c*]pyran-6-yl)benzene (15d)

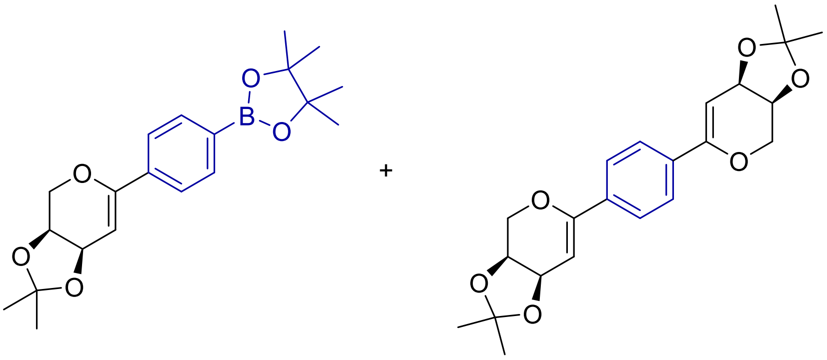

A B

According to GP-B, using *t*BuLi (0.26 mmol, 1.3 equiv.) and stirring for 30 min at -30°C for lithiation and employing 1,4-bis(4,4,5,5-tetramethyl-1,3,2-dioxaborolan-2-yl)benzene (33 mg, 0.10 mmol, 0.5 equiv.). Flash column chromatography (SiO_2 – pentane/ EtOAc 99:1 to 88:12) furnished the title compounds as colorless oils (**15c** = 18 mg, 0.05 mmol, 50 % | **15d** = 14 mg, 0.04 mmol, 36 %).

^{1}H NMR: 15c (500 MHz, $CDCl_3$) δ = 7.78 (d, *J* = 7.7 Hz, 2H), 7.60 (d, *J* = 7.7 Hz, 2H), 5.64 (d, *J* = 4.3 Hz, 1H), 4.70 (t, *J* = 5.1 Hz, 1H), 4.30 (dt, *J* = 9.4, 5.1 Hz, 1H), 4.22 (dd, *J* = 11.0, 4.1 Hz, 1H), 3.84 (dd, *J* = 11.0, 8.2 Hz, 1H), 1.48 (s, 3H), 1.43 (s, 3H), 1.34 (s, 12H).

^{13}C-NMR: 15c (126 MHz, $CDCl_3$) δ = 155.59, 136.97, 134.84, 124.53, 109.23, 96.58, 84.02, 70.68, 68.98, 66.17, 28.52, 26.27, 25.01.

^{1}H NMR: 15d (500 MHz, $CDCl_3$) δ = 7.58 (d, *J* = 1.6 Hz, 4H), 5.61 (d, *J* = 4.2 Hz, 2H), 4.70 (t, *J* = 5.2 Hz, 2H), 4.33 – 4.27 (m, 2H), 4.22 (dd, *J* = 10.9, 4.0 Hz, 2H), 3.84 (dd, *J* = 10.9, 8.1 Hz, 2H), 1.48 (s, 6H), 1.43 (s, 6H).

^{13}C NMR: 15d (126 MHz, $CDCl_3$) δ = 155.16, 134.97, 125.22, 109.25, 96.29, 70.67, 68.95, 66.18, 28.52, 26.26.

HR-MS (ESI): **15c** m/z calcd. for ([$C_{20}H_{28}BO_5$]$^+$, [M^+]): 359.2032, found: 359.203.

HR-MS (ESI): **15d** m/z calcd. for ([$C_{22}H_{27}O_6$]$^+$, [M^+]): 387.1809, found: 387.181.

Derivatizations

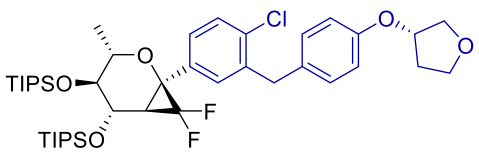

16 (82%)

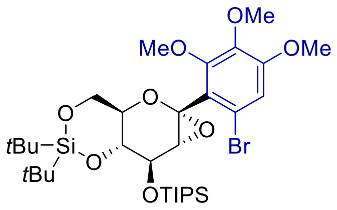

17 (62%)

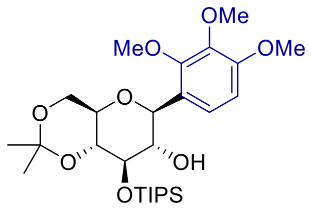

18 (31%)

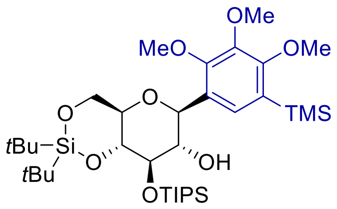

19a (32%)

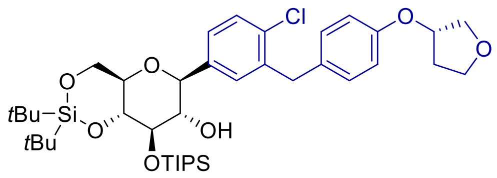

19b (39%)

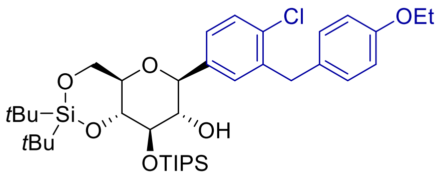

19c (43%)

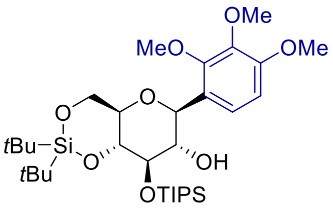

19d (61%)

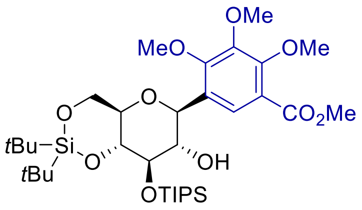

19e (82%)

(((3*S*,4*S*,5*R*,6*R*)-1-(4-Chloro-3-(4-(((*S*)-tetrahydrofuran-3-yl)oxy)benzyl)phenyl)-7,7-difluoro-3-methyl-2-oxabicyclo[4.1.0]heptane-4,5-diyl)bis(oxy))bis(triisopropylsilane) (16)

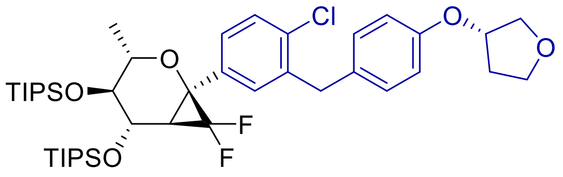

(((2*S*,3*S*,4*R*)-6-(4-Chloro-3-(4-(((*S*)-tetrahydrofuran-3-yl)oxy)benzyl)phenyl)-2-methyl-3,4-dihydro-2H-pyran-3,4-diyl)bis(oxy))bis(triisopropylsilane) **11d** (73 mg, 0.1 mmol, 1.0 equiv.) was charged to an Ace pressure tube containing NaI (8 mg, 0.05 mmol, 0.5 equiv.) and dry THF (2 mL) was added. $TMSCF_3$ (37 µL, 0.25 mmol, 2.5 equiv.) was added in one portion and the vial was sealed. The reaction mixture was heated to 65 °C for 6 h, the solvent was removed *in vacuo* and the crude product was purified by flash column chromatography (SiO_2; pentane/ EtOAc: 999:1 to 92:8) to obtain the title compound as a colorless oil (64 mg, 0.08 mmol, 82 %).

^{1}H-NMR: (500 MHz, $CDCl_3$) δ = 7.34 (d, *J* = 8.3 Hz, 1H), 7.26 (m, 1H), 7.21 (dd, *J* = 8.3, 2.3 Hz, 1H), 7.10 – 7.07 (m, 2H), 6.79 – 6.76 (m, 2H), 4.89 (ddt, *J* = 6.4, 4.6, 2.3 Hz, 1H), 4.29 (s, 1H), 4.10 (q, *J* = 7.2 Hz, 1H), 4.04 (s, 2H), 4.01 – 3.94 (m, 3H), 3.92 – 3.86 (m, 1H), 3.62 (t, *J* = 1.8 Hz, 1H), 2.22 – 2.10 (m, 3H), 1.15 – 1.01 (m, 45H).
^{13}C-NMR: (126 MHz, $CDCl_3$) δ = 155.87, 138.56, 135.70 (d, J = 1.8 Hz), 133.29, 131.72, 129.91, 129.25, 128.73, 125.21, 73.83 (d, J = 2.4 Hz), 73.17, 72.75 (d, J = 1.7 Hz), 67.21, 64.96, 38.53, 33.03, 18.13, 18.03, 16.67, 12.58, 12.28.
^{19}F NMR: (471 MHz, $CDCl_3$) δ = -132.80, -133.13, -144.02, -144.35.

HR-MS (ESI): m/z calcd. for ($[C_{42}H_{65}ClF_2O_5Si_2]^+$, $[H^+]$): 779.4107, found: 779.411.

(4a*R*,5a*S*,6a*R*,7*R*,7a*R*)-5a-(6-Bromo-2,3,4-trimethoxyphenyl)-2,2-di-*tert*-butyl-7-((triisopropylsilyl)oxy)hexahydrooxireno[2',3':5,6]pyrano[3,2-*d*][1,3,2]dioxasiline (17)

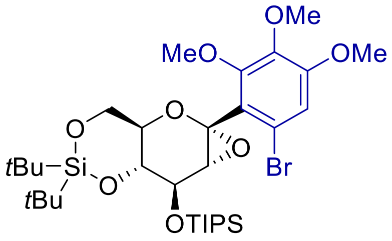

Compound **8c** (0.2 mmol, 1.0 equiv.) was dissolved in dry DCM (5 mL), and the solution was cooled to -78°C. A freshly prepared solution of DMDO in acetone (2 mmol, 10 equiv.) was added dropwise and the reaction was allowed to stir for 1h at the aforementioned temperature. The cooling bath was removed and the reaction mixture was allowed to warm to rt. for 1h. After that, the solvents were removed *in vacuo*, and the crude product was purified *via* flash column chromatography (SiO_2; pentane/ EtOAc: 98:2 -> 85:15) to obtain the title compound as a colorless oil, which solidified in the fridge (88 mg, 0.12 mmol, 62 %)

^{1}H-NMR: (500 MHz, $CDCl_3$) δ = 6.89 (s, 1H), 4.79 (d, *J* = 2.2 Hz, 1H), 4.58 (dd, *J* = 6.8, 2.2 Hz, 1H), 4.21 – 4.10 (m, 2H), 4.05 – 4.00 (m, 2H), 3.88 – 3.83 (m, 9H), 1.13 – 1.07 (m, 30H), 1.02 (s, 9H).

^{13}C-NMR: (126 MHz, $CDCl_3$) δ = 154.42, 153.30, 147.91, 141.95, 124.36, 118.04, 112.02, 107.70, 77.92, 73.54, 71.90, 66.16, 62.08, 61.04, 56.41, 27.65, 27.15, 22.92, 20.03, 18.30, 12.61.

HR-MS (ESI): m/z calcd. for ($[C_{32}H_{56}BrO_8Si_2]^+$, $[H^+]$): 703.2699, found: 703.269.

(4a*R*,6*S*,7*S*,8*R*,8a*R*)-2,2-Dimethyl-8-((triisopropylsilyl)oxy)-6-(2,3,4-trimethoxyphenyl)hexahydropyrano[3,2-*d*][1,3]dioxin-7-ol (18)

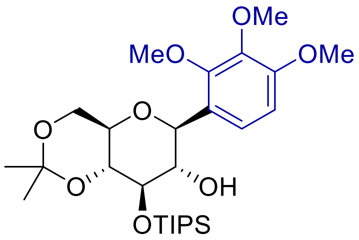

Compound **7c** (0.35 g, 0.66 mmol, 1.0 equiv.) was suspended in dry THF (10 mL) and cooled to 0°C. Borane THF complex (6.6 mmol, 10 equiv.) was added dropwise at 0°C and the reaction mixture was allowed to stir for 22 h at rt. Following this, the reaction mixture was cooled to 0°C and a 1:1 mixture of H_2O_2 (30 %, 5 mL) and 2 M NaOH (5 mL) was added carefully dropwise and the ice-bath was removed. After stirring at ambient temperature for 4 h, sat. aq. NH_4Cl (20 mL) was added and the reaction mixture was transferred to a separatory funnel. H_2O (40 mL) was added and the aq. fraction was extracted with EtOAc (3 × 50 mL). The combined organic fractions were washed with Brine, dried over anhydr. $MgSO_4$ and concentrated *in vacuo*. Flash column chromatography (SiO_2; pentane/ EtOAc: 9:1 -> 8:2) yielded the title compound as a colorless amorphous solid (0.11 g, 0.2 mmol, 31 %).

^{1}H-NMR: (500 MHz, $CDCl_3$) δ = 7.10 (d, *J* = 8.6 Hz, 1H), 6.73 (d, *J* = 8.6 Hz, 1H), 4.67 (d, *J* = 9.7 Hz, 1H), 3.93 – 3.84 (m, 11H), 3.74 (t, *J* = 10.5 Hz, 1H), 3.70 – 3.66 (m, 1H), 3.63 (t, *J* = 9.3 Hz, 1H), 3.47 – 3.42 (m, 1H), 1.51 (s, 3H), 1.41 (s, 3H), 1.21 – 1.14 (m, 3H), 1.09 (t, *J* = 7.5 Hz, 18H).

Note: ^{1}H-NMR shows trace of DCM.

^{13}C-NMR: (126 MHz, $CDCl_3$) δ = 153.92, 152.61, 142.31, 124.75, 122.31, 108.09, 99.42, 77.71, 76.49, 76.17, 74.35, 72.48, 62.57, 61.80, 60.93, 56.18, 29.19, 18.99, 18.41, 18.34, 12.73.

HR-MS (ESI): m/z calcd. for ($[C_{27}H_{46}O_8Si]^+$, $[Na^+]$): 549.2859, found: 549.286.

(4a*R*,6*S*,7*S*,8*R*,8a*R*)-2,2-Di-*tert*-butyl-8-((triisopropylsilyl)oxy)-6-(2,3,4-trimethoxy-5-(trimethylsilyl)phenyl)hexahydropyrano[3,2-*d*][1,3,2]dioxasilin-7-ol (19a)

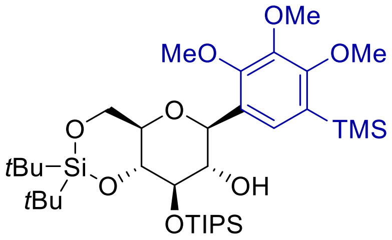

According to GP-B on a 0.9 mmol scale, using *t*BuLi (0.99 mmol, 1.1 equiv.) and stirring for 60 min at -30°C for lithiation and employing trimethyl(2,3,4-trimethoxy-5-(4,4,5,5-tetramethyl-1,3,2-dioxaborolan-2-yl)phenyl)silane (0.38 g, 1.0 mmol, 1.15 equiv.). After extraction, the crude glycal was dried under high vacuum and directly submitted to hydroboration - oxidation. Dry THF (10 mL) was added, and borane THF complex (5 mmol, 5 equiv.) was added dropwise at 0°C and the reaction mixture was allowed to stir at rt. for 24 h. Following this, the reaction mixture was cooled to 0°C and a 1:1 mixture of H_2O_2 (30 %, 5 mL) and 2 M NaOH (5 mL) was added carefully dropwise and the ice-bath was removed. After stirring at ambient temperature for 2 h, sat. aq. NH_4Cl (30 mL) was added and the reaction mixture was transferred to a seperatory funnel. H_2O (50 mL) was added and the aq. fraction was extracted with EtOAc (3 × 50 mL). The combined organic fractions were washed with Brine, dried over anhydr. $MgSO_4$ and concentrated *in vacuo*. Flash column chromatography (SiO_2; pentane/ EtOAc: 98:2 -> 9:1) yielded the title compound as a colorless oil (0.2 g, 0.29 mmol, 32 % overall yield).

^{1}H-NMR: (500 MHz, $CDCl_3$) δ = 7.04 (d, *J* = 8.1 Hz, 1H), 6.67 (d, *J* = 8.1 Hz, 1H), 4.61 (d, *J* = 9.7 Hz, 1H), 4.17 (dd, *J* = 10.2, 4.9 Hz, 1H), 3.91 (s, 3H), 3.89 – 3.85 (m, 8H), 3.65 (t, *J* = 9.0 Hz, 1H), 3.56 (td, *J* = 9.7, 4.9 Hz, 1H), 1.29 – 1.23 (m, 3H), 1.13 (t, *J* = 6.9 Hz, 18H), 1.08 (s, 9H), 1.03 (s, 9H), 0.26 (s, 9H).
^{13}C-NMR: (126 MHz, $CDCl_3$) δ = 158.79, 155.32, 154.16, 145.27, 129.37, 128.87, 127.77, 126.77, 107.36, 80.40, 78.30, 75.72, 75.70, 66.83, 61.73, 60.72, 60.63, 60.56, 60.46, 56.08, 27.70, 27.19, 22.93, 20.14, 18.67, 18.55, 13.14, -0.34, -0.36.
HR-MS (ESI): m/z calcd. for ($[C_{35}H_{66}O_8Si_3]^+$, $[H^+]$): 699.4145, found: 699.414.

(4a*R*,6*S*,7*S*,8*R*,8a*R*)-2,2-Di-*tert*-butyl-6-(4-chloro-3-(4-(((*S*)-tetrahydrofuran-3-yl)oxy)benzyl)phenyl)-8-((triisopropylsilyl)oxy)hexahydropyrano[3,2-*d*][1,3,2]dioxasilin-7-ol (19b)

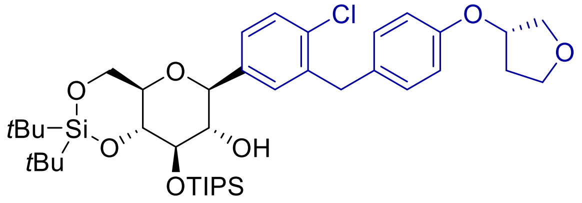

According to GP-B on a 0.2 mmol scale, using *t*BuLi (0.22 mmol, 1.1 equiv.) and stirring for 60 min at -30°C for lithiation and employing (*S*)-2-(4-chloro-3-(4-((tetrahydrofuran-3-yl)oxy)benzyl)phenyl)-4,4,5,5-tetramethyl-1,3,2-dioxaborolane (95 mg, 0.23 mmol, 1.15 equiv.). After extraction, the crude glycal was dried under high vacuum and directly submitted to hydroboration - oxidation. Dry THF (2 mL) was added, and borane THF complex (1 mmol, 5 equiv.) was added dropwise at 0°C and the reaction mixture was allowed to stir at rt. for 24 h. Following this, the reaction mixture was cooled to 0°C and a 1:1 mixture of H_2O_2 (30 %, 1 mL) and 2 M NaOH (1 mL) was added carefully dropwise and the ice-bath was removed. After stirring at ambient temperature for 1 h, sat. aq. NH_4Cl (10 mL) was added and the reaction mixture was transferred to a seperatory funnel. H_2O (20 mL) was added and the aq. fraction was extracted with EtOAc (3 × 20 mL). The combined organic fractions were washed with Brine, dried over anhydr. $MgSO_4$ and concentrated *in vacuo*. Flash column chromatography (SiO_2; pentane/ EtOAc: 9:1 -> 8:2) yielded the title compound as a colorless amorphous solid (58 mg, 0.08 mmol, 39 % overall yield).

^{1}H-NMR: (500 MHz, $CDCl_3$) δ = 7.37 (d, *J* = 8.1 Hz, 1H), 7.22 – 7.18 (m, 2H), 7.10 (d, *J* = 8.1 Hz, 2H), 6.78 (d, *J* = 8.5 Hz, 2H), 4.88 (dp, *J* = 6.6, 2.2 Hz, 1H), 4.20 – 4.14 (m, 2H), 4.11 – 4.06 (m, 1H), 4.01 – 3.94 (m, 4H), 3.91 – 3.80 (m, 4H), 3.53 (td, *J* = 9.7, 4.9 Hz, 1H), 3.43 (t, *J* = 8.8 Hz, 1H), 2.21 – 2.10 (m, 2H), 1.26 – 1.21 (m, 3H), 1.12 (t, *J* = 7.4 Hz, 18H), 1.08 (s, 9H), 1.01 (s, 9H).
^{13}C-NMR: (126 MHz, $CDCl_3$) δ = 155.99, 139.05, 137.58, 134.36, 131.87, 130.36, 130.08, 129.85, 126.49, 115.49, 81.52, 79.92, 78.13, 76.62, 75.45, 73.26, 67.32, 66.67, 38.54, 33.14, 27.63, 27.13, 22.91, 20.10, 18.62, 18.50, 13.13.
HR-MS (ESI): m/z calcd. for ([$C_{40}H_{63}ClO_7Si_2$]$^+$, [H^+]): 747.3881, found: 747.389.

(4a*R*,6*S*,7*S*,8*R*,8a*R*)-2,2-Di-*tert*-butyl-6-(4-chloro-3-(4-ethoxybenzyl)phenyl)-8-((triisopropylsilyl)oxy)hexahydropyrano[3,2-*d*][1,3,2]dioxasilin-7-ol (19c)

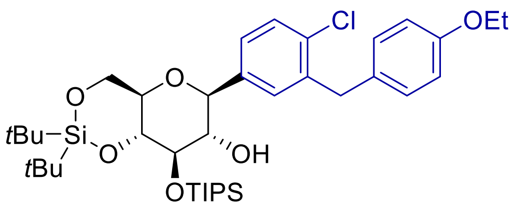

According to GP-B on a 0.2 mmol scale, using *t*BuLi (0.22 mmol, 1.1 equiv.) and stirring for 60 min at -30°C for lithiation and employing 2-(4-chloro-3-(4-ethoxybenzyl)phenyl)-4,4,5,5-tetramethyl-1,3,2-dioxaborolane (86 mg, 0.23 mmol, 1.15 equiv.). After extraction, the crude glycal was dried under high vacuum and directly submitted to hydroboration - oxidation. Dry THF (2 mL) was added, and borane THF complex (1 mmol, 5 equiv.) was added dropwise at 0°C and the reaction mixture was allowed to stir for 16 h at rt. Following this, the reaction mixture was cooled to 0°C and a 1:1 mixture of H_2O_2 (30 %, 1 mL) and 2 M NaOH (1 mL) was added carefully dropwise and the ice-bath was removed. After stirring at ambient temperature for 1 h, sat. aq. NH_4Cl (10 mL) was added and the reaction mixture was transferred to a seperatory funnel. H_2O (20 mL) was added and the aq. fraction was extracted with EtOAc (3 × 20 mL). The combined organic fractions were washed with Brine, dried over anhydr. $MgSO_4$ and concentrated *in vacuo*. Flash column chromatography (SiO_2; pentane/ EtOAc: 96:4 -> 88:12) yielded the title compound as a colorless amorphous solid (61 mg, 0.09 mmol, 43 % overall yield).

^{1}H-NMR: (500 MHz, $CDCl_3$) δ = 7.36 (d, *J* = 8.0 Hz, 1H), 7.22 – 7.16 (m, 2H), 7.09 (d, *J* = 8.0 Hz, 2H), 6.81 (d, *J* = 8.0 Hz, 2H), 4.16 (dd, *J* = 9.7, 6.8 Hz, 2H), 4.08 (d, *J* = 15.4 Hz, 1H), 4.03 – 3.96 (m, 3H), 3.91 – 3.79 (m, 3H), 3.52 (td, *J* = 9.7, 4.8 Hz, 1H), 3.46 – 3.39 (m, 1H), 1.40 (t, *J* = 7.0 Hz, 3H), 1.23 (dt, *J* = 15.0, 7.8 Hz, 3H), 1.12 (t, *J* = 7.4 Hz, 18H), 1.07 (s, 9H), 1.01 (s, 9H).

^{13}C-NMR: (126 MHz, $CDCl_3$) δ = 157.56, 139.24, 137.51, 134.39, 131.36, 130.37, 130.00, 129.83, 126.42, 114.60, 81.55, 79.94, 78.14, 76.60, 75.46, 66.68, 63.54, 38.56, 27.64, 27.14, 22.92, 20.12, 18.63, 18.50, 15.02, 13.13.

HR-MS (ESI): m/z calcd. for ($[C_{38}H_{61}ClO_6Si_2]^+$, $[H^+]$): 705.3775, found: 705.377.

(4a*R*,6*S*,7*S*,8*R*,8a*R*)-2,2-Di-*tert*-butyl-8-((triisopropylsilyl)oxy)-6-(2,3,4-trimethoxyphenyl)hexahydropyrano[3,2-*d*][1,3,2]dioxasilin-7-ol (19d)

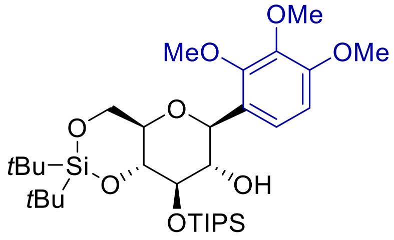

Zweifel Protocol according to GP-B on a 1.6 mmol scale, using *t*BuLi (2.1 mmol, 1.3 equiv.) and stirring for 60 min at -30°C for lithiation and employing 4,4,5,5-tetramethyl-2-(2,3,4-trimethoxyphenyl)-1,3,2-dioxaborolane (0.54 g, 1.8 mmol, 1.15 equiv.). After extraction, the crude glycal was dried under high vacuum and directly submitted to hydroboration - oxidation. Dry THF (15 mL) was added, and borane THF complex (8 mmol, 5 equiv.) was added dropwise at 0°C and the reaction mixture was allowed to stir at ambient temperature for 24 h. Following this, the reaction mixture was cooled to 0°C and a 1:1 mixture of H_2O_2 (30 %, 5 mL) and 2 M NaOH (5 mL) was added carefully dropwise and the ice-bath was removed. After stirring at ambient temperature for 5 h, sat. aq. NH_4Cl (30 mL) was added and the reaction mixture was transferred to a seperatory funnel. H_2O (50 mL) was added and the aq. fraction was extracted with EtOAc (3 × 50 mL). The combined organic fractions were washed with Brine, dried over anhydr. $MgSO_4$ and concentrated *in vacuo*. Flash column chromatography (SiO_2; pentane/ EtOAc: 95:5 -> 8:2) yielded the title compound as a colorless amorphous solid (0.61 g, 0.98 mmol, 61 % overall yield).

^{1}H NMR (500 MHz, $CDCl_3$) δ = 7.07 (d, *J* = 8.7 Hz, 1H), 6.72 (d, *J* = 8.7 Hz, 1H), 4.63 (d, *J* = 9.7 Hz, 1H), 4.16 (dd, *J* = 10.1, 5.0 Hz, 1H), 3.92 (s, 3H), 3.86 (d, *J* = 10.1 Hz, 9H), 3.64 – 3.53 (m, 2H), 1.24 (p, *J* = 7.4 Hz, 3H), 1.12 (t, *J* = 6.8 Hz, 18H), 1.07 (s, 9H), 1.02 (s, 9H).

^{13}C NMR (126 MHz, $CDCl_3$) δ = 153.92, 152.58, 142.28, 124.73, 122.25, 108.15, 80.36, 78.37, 76.03, 75.70, 75.57, 66.84, 61.85, 60.93, 56.18, 27.67, 27.19, 22.95, 20.13, 18.66, 18.53, 13.15.

HR-MS (ESI): m/z calcd. for ($[C_{32}H_{59}O_8Si_2]^+$, $[H^+]$): 627.3750, found: 627.375.

Methyl 5-((4a*R*,6*S*,7*S*,8*R*,8a*R*)-2,2-di-*tert*-butyl-7-hydroxy-8-((triisopropylsilyl)oxy)hexahydropyrano[3,2-*d*][1,3,2]dioxasilin-6-yl)-2,3,4-trimethoxybenzoate (19e)

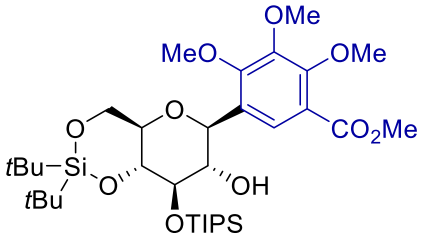

Compound **19d** (0.53 g, 0.85 mmol, 1.0 equiv.) was charged to a flame dried flask and dissolved in dry THF (8 mL). A freshly prepared solution of TMPLi (1.8 mmol, 2.1 equiv.) in THF (2 mL) was added dropwise to the mixture at -40 °C, and the solution was allowed to stir at that temperature for 1h. Methyl chloroformate (0.2 mL, 2.5 mmol, 3.0 equiv.) was added dropwise and the mixture stirred at -78°C for 1h, before warming to ambient temperature and stirring at this temperature for 30 min. The reaction was quenched by addition of sat. aq. NH_4Cl (3 mL) and the mixture was transferred to a seperatory funnel. Water (50 mL) and EtOAc (50 mL) was added and the mixture was extracted twice more with EtOAc (50 mL). The combined organic fractions were washed with Brine (50 mL), dried over anhydr. $MgSO_4$ and concentrated. Flash column chromatography (SiO_2; pentane – EtOAc | 95:5 -> 8:2) afforded the title compound as a bright yellow solid (0.47 g, 0.69 mmol, 82 %).

^{1}H-NMR (500 MHz, $CDCl_3$) δ = 7.10 (d, *J* = 8.7 Hz, 1H), 6.68 (d, *J* = 8.7 Hz, 1H), 4.88 (t, *J* = 9.4 Hz, 1H), 4.71 (d, *J* = 10.0 Hz, 1H), 4.17 (dd, *J* = 10.0, 4.9 Hz, 1H), 4.01 (t, *J* = 8.6 Hz, 1H), 3.93 (d, *J* = 9.0 Hz, 1H), 3.88 (s, 3H), 3.83 (d, *J* = 6.6 Hz, 6H), 3.55 (td, *J* = 10.0, 4.9 Hz, 1H), 3.51 (s, 3H), 1.18 – 1.13 (m, 3H), 1.12 – 1.06 (m, 27H), 1.02 (s, 9H).

^{13}C-NMR (126 MHz, $CDCl_3$) δ = 154.75, 153.99, 152.58, 141.84, 123.41, 122.76, 107.70, 79.31, 78.65, 75.52, 66.68, 61.72, 60.81, 56.05, 54.71, 27.65, 27.16, 22.96, 20.13, 18.54, 18.41, 13.34.

HR-MS (ESI): m/z calcd. for ($[C_{34}H_{61}O_{10}Si_2]^+$, $[H^+]$): 685.3805, found: 685.381.

5. References

[1] B. Fraser-Reid, D. L. Walker, S. Y.-K. Tam, N. L. Holder, *Can. J. Chem.* **1973**, *51*, 3950–3954.

[2] S. E. Denmark, T. Kobayashi, C. S. Regens, *Tetrahedron* **2010**, *66*, 4745–4759.

[3] K. A. Parker, A. T. Georges, *Org. Lett.* **2000**, *2*, 497–499.

[4] R. W. Friesen, C. F. Sturino, A. K. Daljeet, A. Kolaczewska, *J. Org. Chem.* **1991**, *56*, 1944–1947.

[5] G. Gabrielli et al., *J. Carbohydr. Chem.* **2009**, *28*, 124–141.

[6] Y. Kobayashi, S. Masakado, Y. Takemoto, *Angew. Chem. Int. Ed.* **2018**, *57*, 693– 69.

[7] K. Parkan, R. Pohl, M. Kotora, *Chem. - Eur. J.* **2014**, *20*, 4414–4419.

[8] T. Shinozuka*, ACS Omega* **2020**, 5, *51*, 33196–33205

[9] *O. Kjolberg, K. Neumann, Acta.Chem.Scand*, **1993**, *47*, 843-845.

[10] L. Pan, M. M. Deckert, M. V. Cooke, A. R. Bleeke, S. Laulhé*, Org. Lett.* **2022**, *24*, 35, 6466–6471.

[11] S. Jin, Hang. T. Dang, G. C. Haug, R. He, V. D. Nguyen, V. T. Nguyen, H. D. Arman, K. S. Schanze, O. V. Larionov, *J. Am. Chem. Soc.* **2020** *142* (3), 1603-1613.

[12] S. Pan, Q. Xie, X. Wang, Q. Wang, C. Nia, J. Hu*, Chem. Commun.*, **2022**, *58*, 5156-5159.

[13] G. Das, T. Prakasam, M. A. Addicoat, S. K. Sharma, F. Ravaux, R. Mathew, M. Baias, R. Jagannathan, M. A. Olson, A. Trabolsi *J. Am. Chem. Soc.* **2019**, *141*, 48, 19078–19087.
