# Supplementary material for: Zweifel olefination for C-glycosylation
Source: Commun Chem. 2024 Dec 21;7:306. doi: 10.1038/s42004-024-01339-4 (PMC11663222; doi:10.1038/s42004-024-01339-4)

NMR Spectra

(((3*R*,4*R*)-3,4-Dihydro-2*H*-pyran-3,4-diyl)bis(oxy))bis(triisopropylsilane) (11)

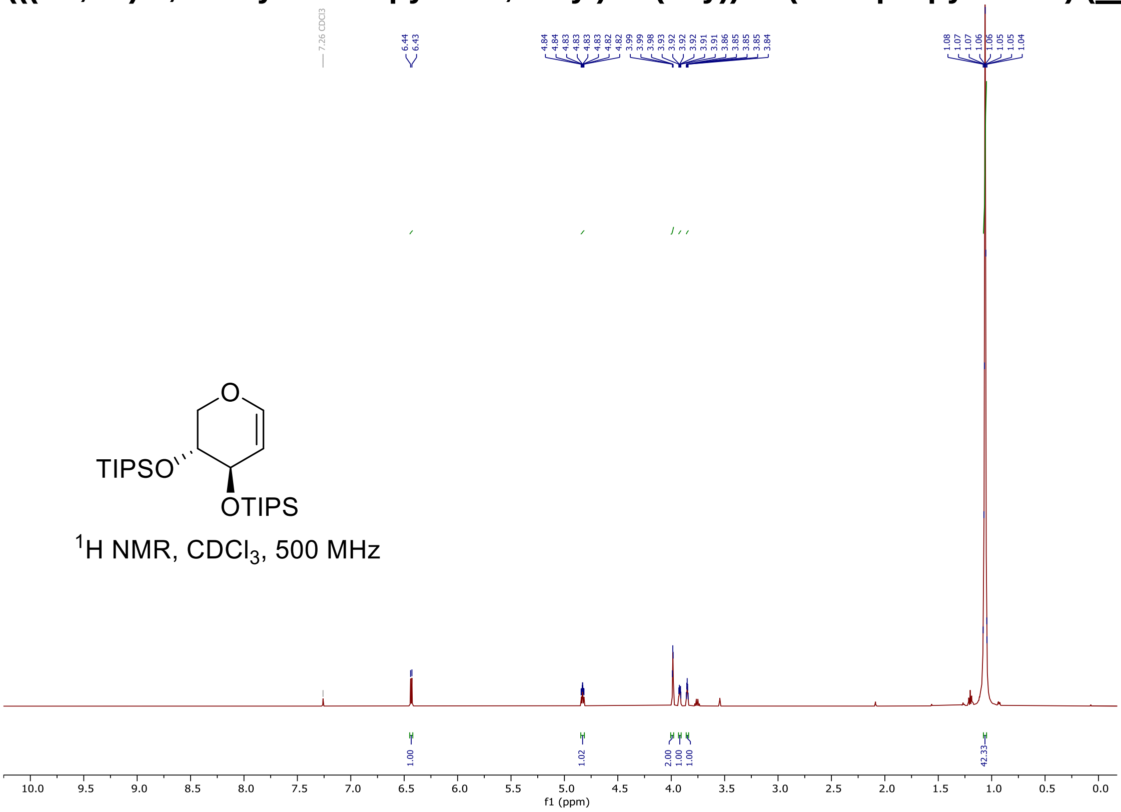

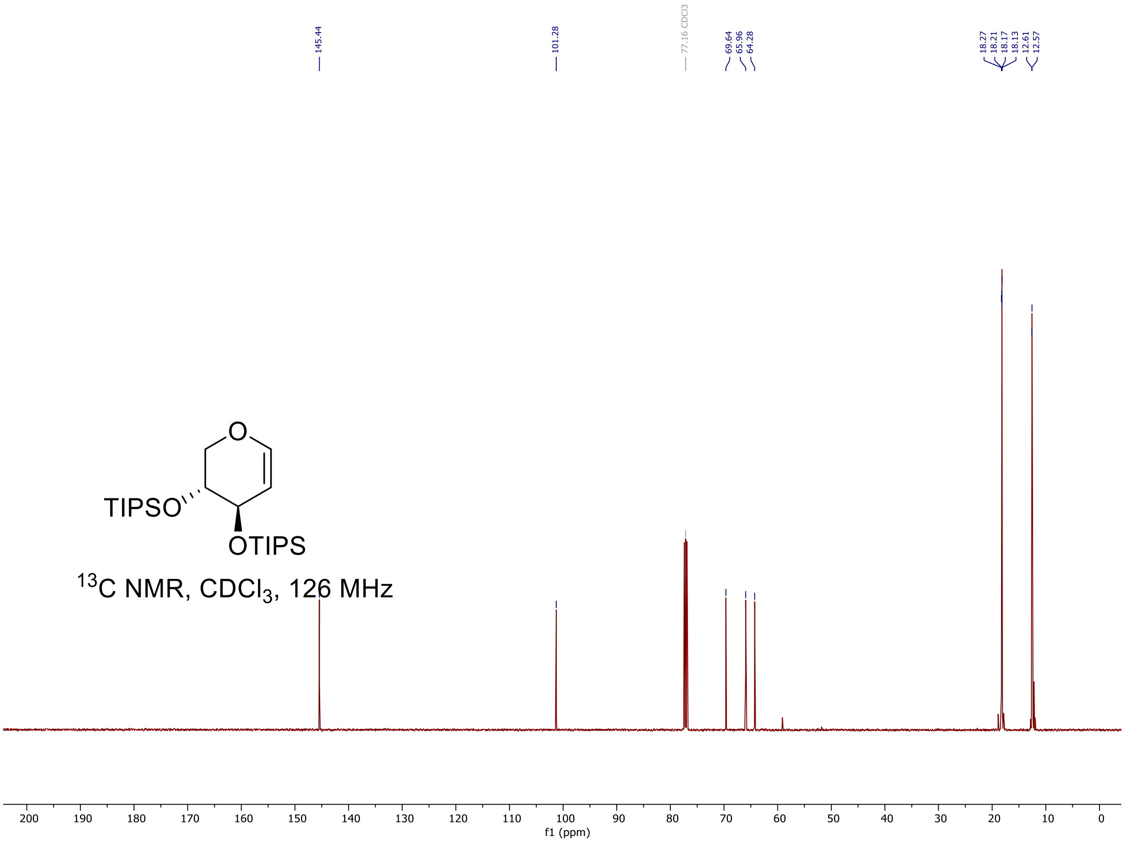

Ethyl 4-(2-chloro-5-(4,4,5,5-tetramethyl-1,3,2-dioxaborolan-2-yl)benzyl)benzoate (<u>SI-8</u>)

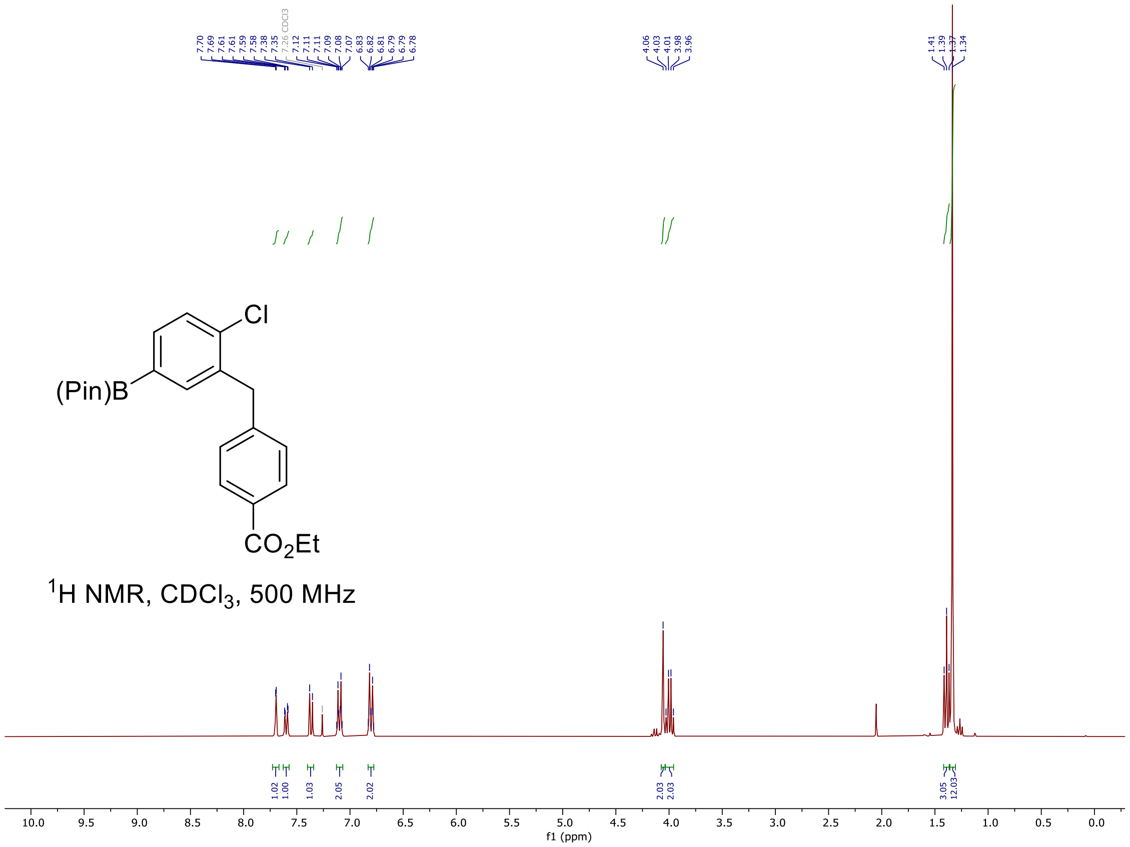

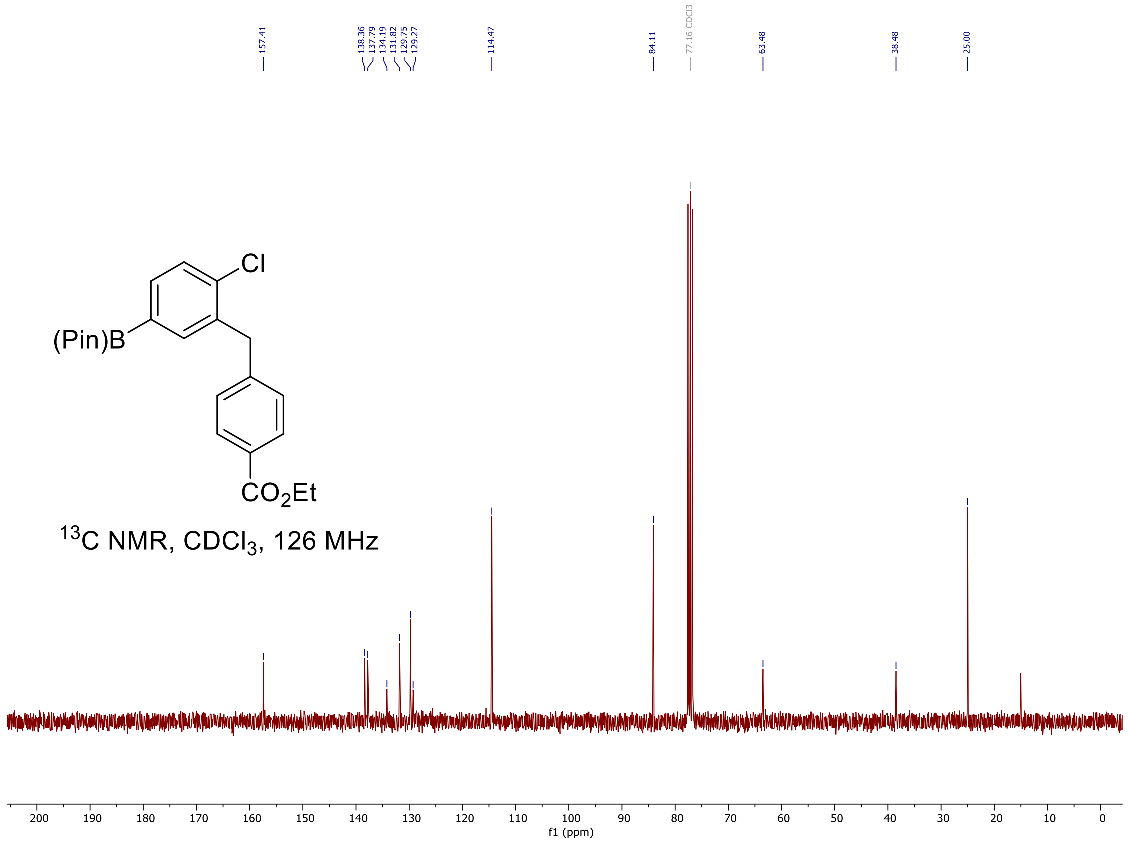

2-(6-Bromo-2,3,4-trimethoxyphenyl)-4,4,5,5-tetramethyl-1,3,2-dioxaborolane (SI-9)

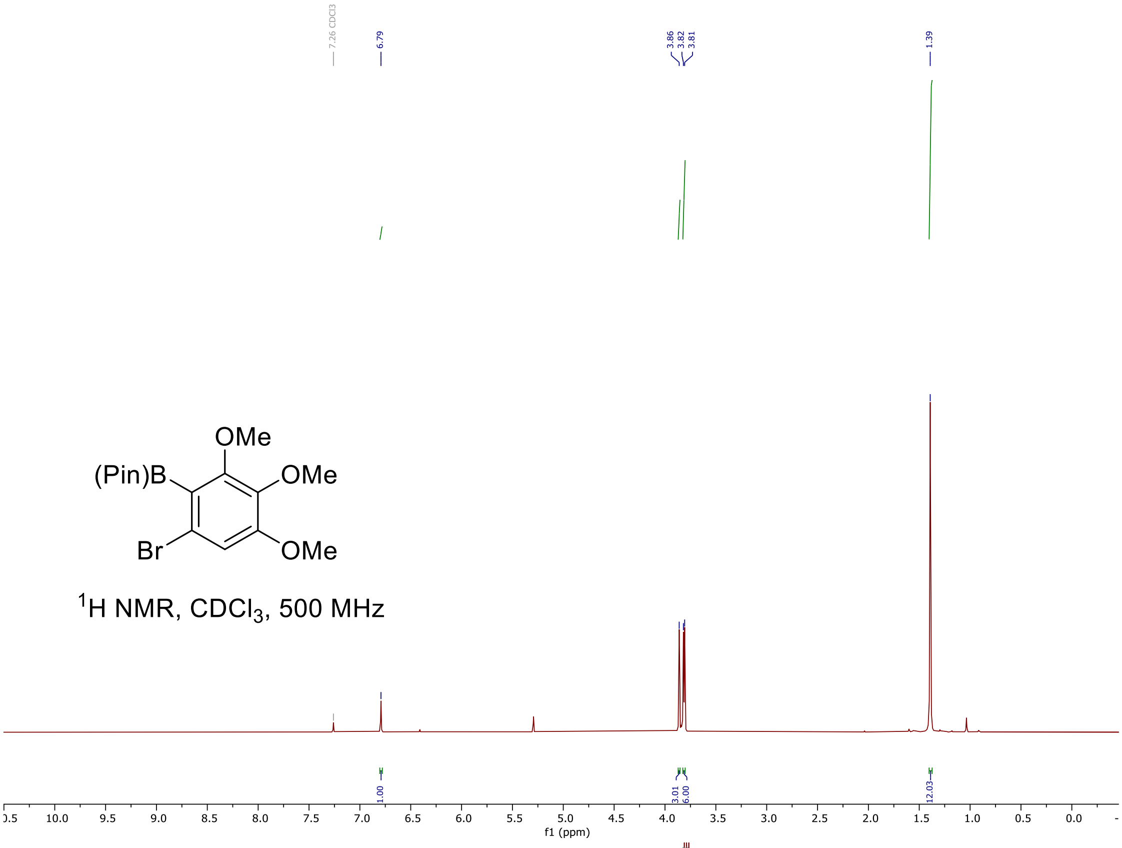

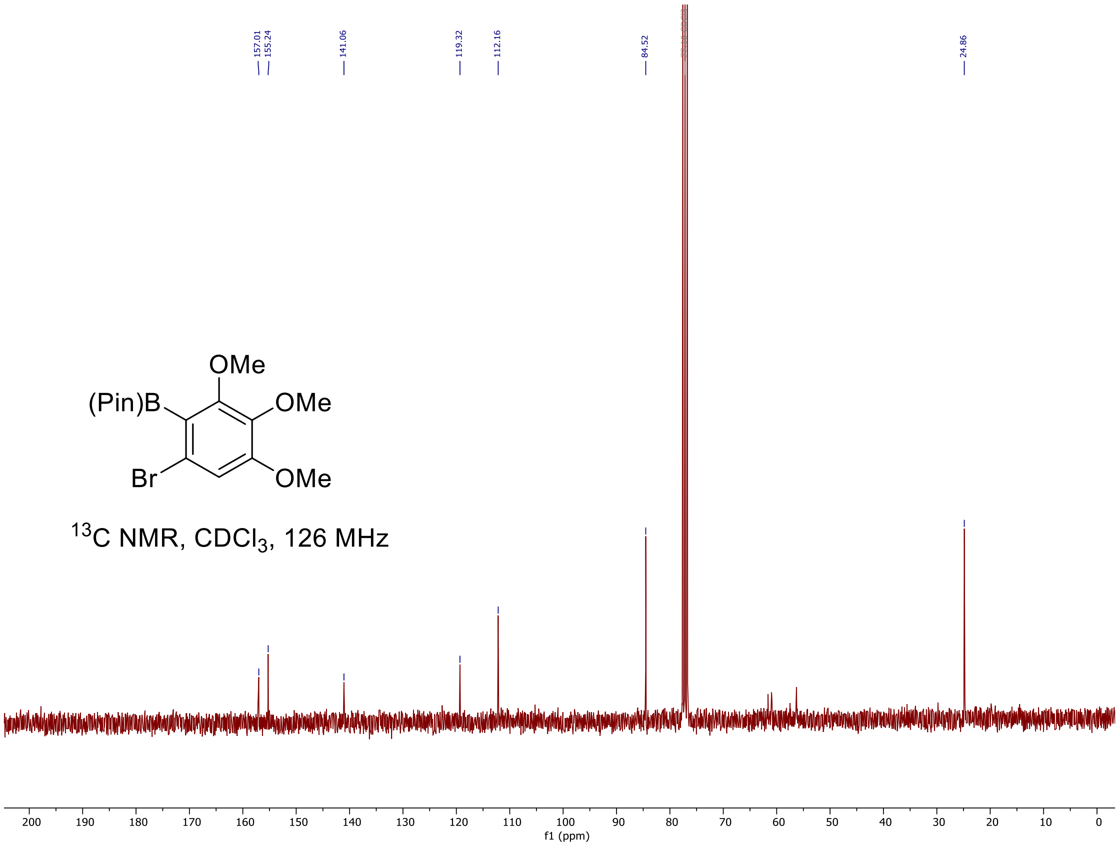

2-(2-(1,3-Dioxolan-2-yl)ethyl)-4,4,5,5-tetramethyl-1,3,2-dioxaborolane (SI-12)

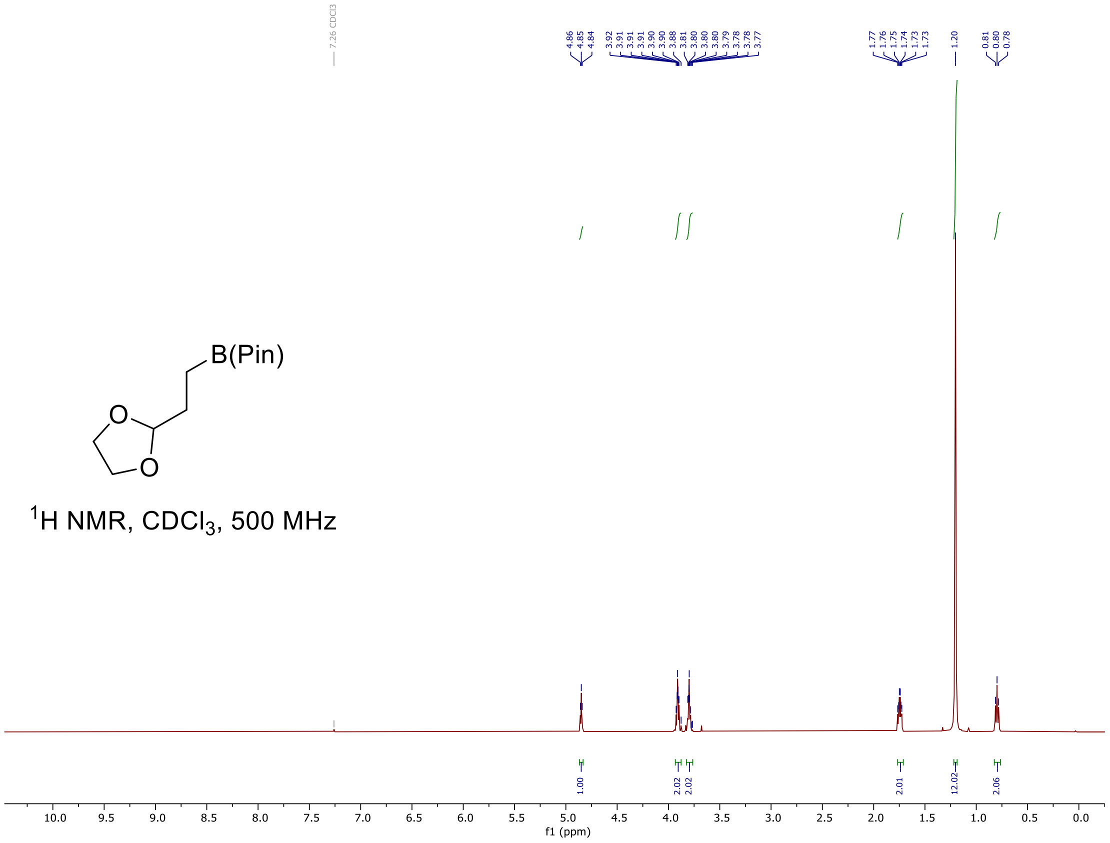

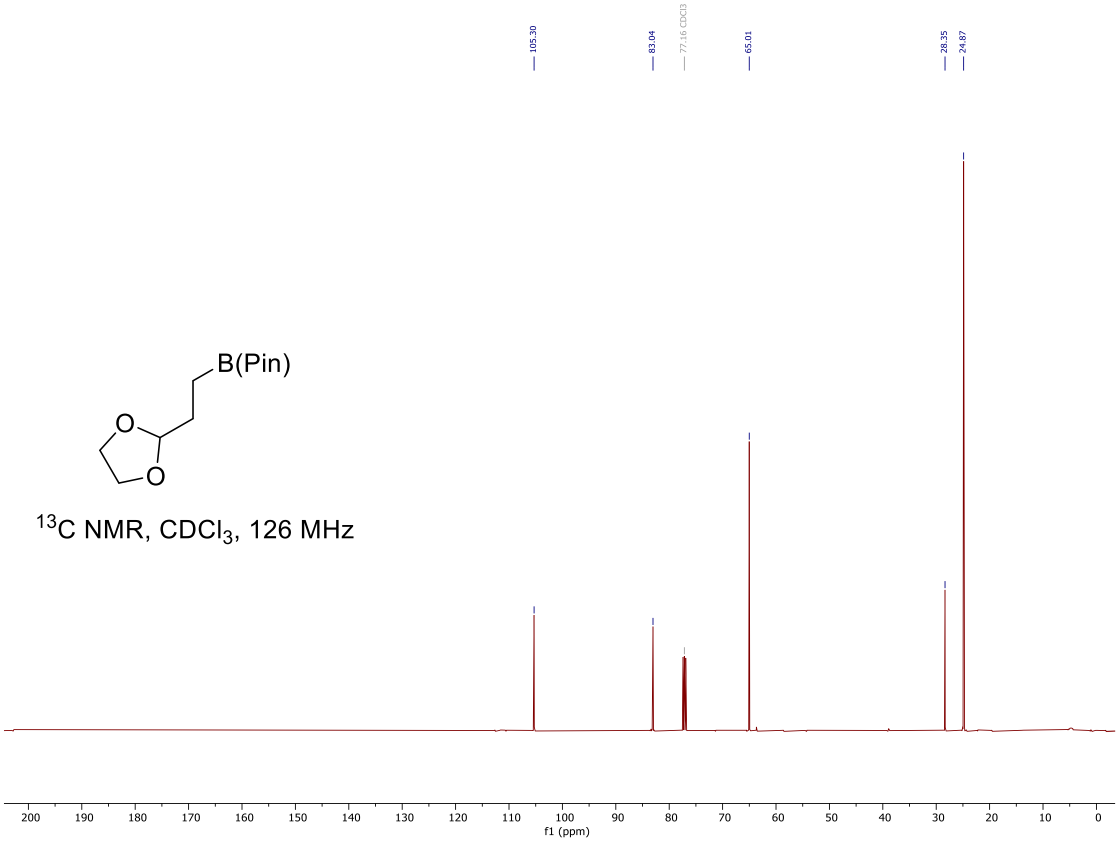

(((4a*R*,8*R*,8a*R*)-6-(3,4-Dimethoxyphenyl)-2,2-dimethyl-4,4a,8,8a-tetrahydropyrano[3,2-*d*][1,3]dioxin-8-yl)oxy)triisopropylsilane (7a)

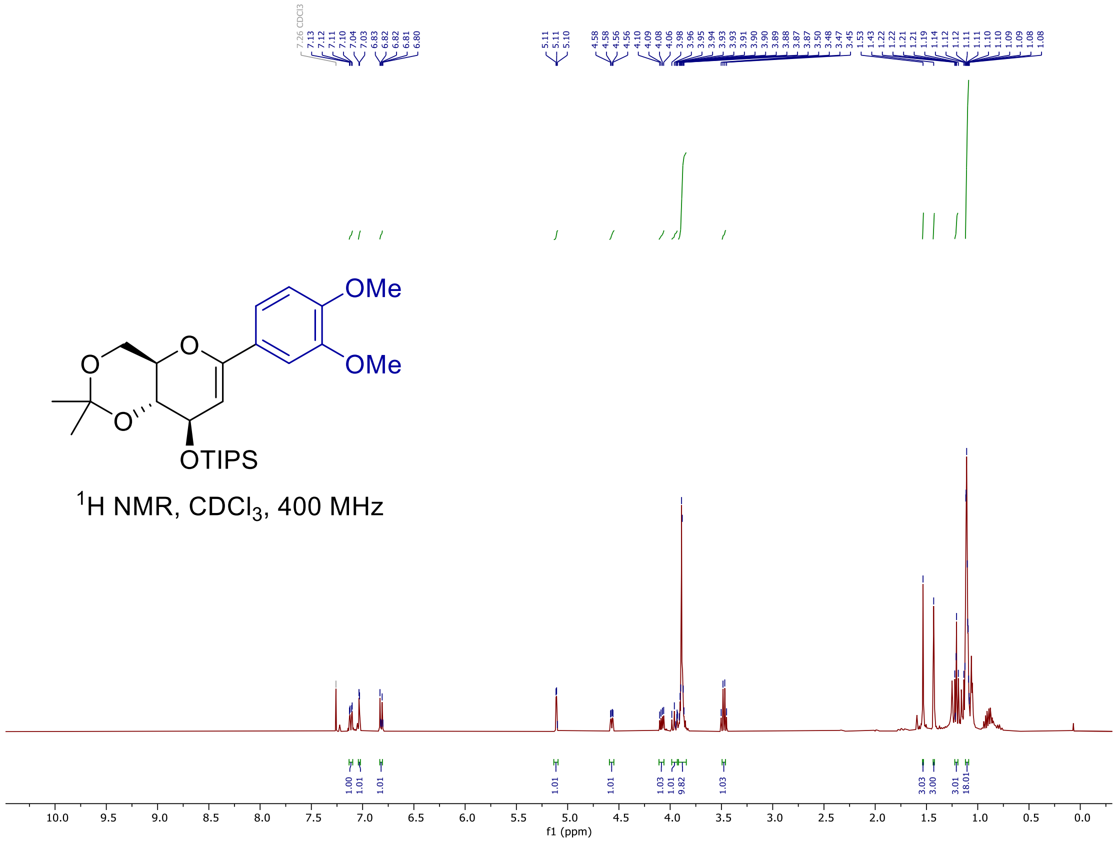

^{1}H NMR, $CDCl_3$, 400 MHz

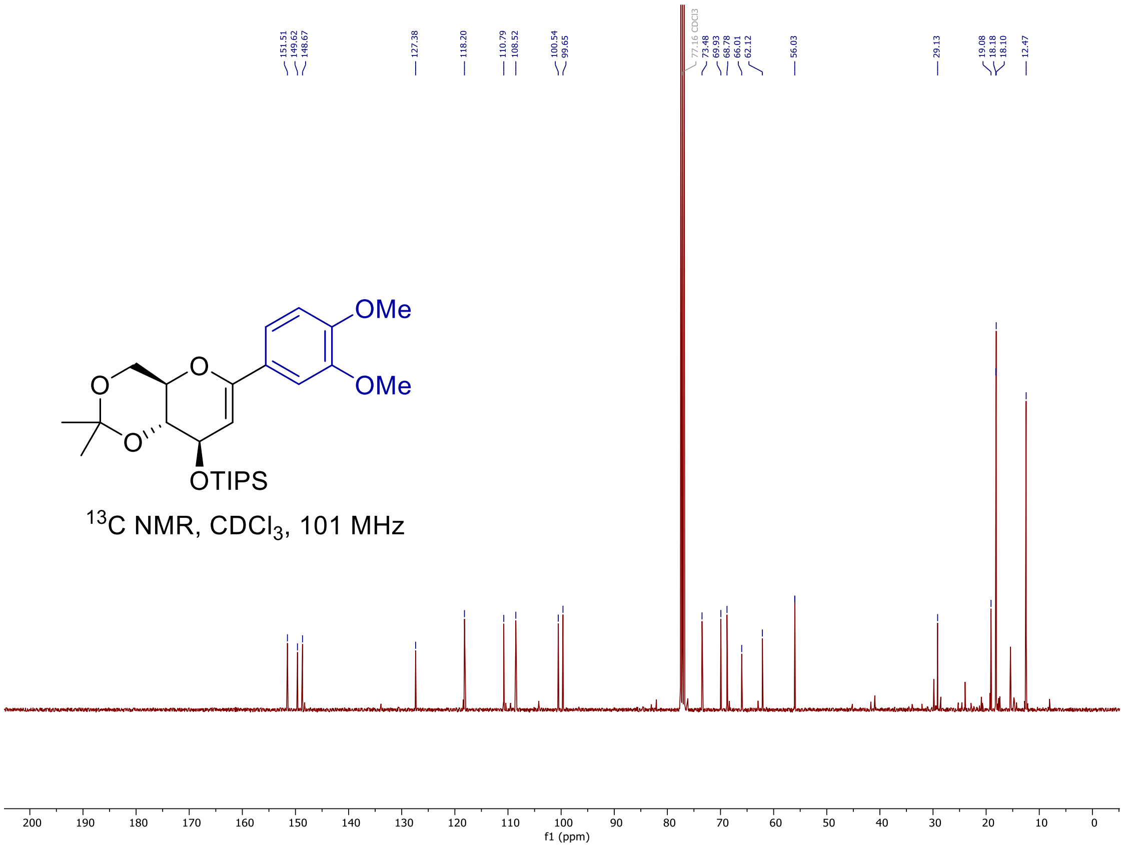

^{13}C NMR, $CDCl_3$, 101 MHz

(((4a*R*,8*R*,8a*R*)-6-(3-Fluoro-4-methoxyphenyl)-2,2-dimethyl-4,4a,8,8a-tetrahydropyrano[3,2-*d*][1,3]dioxin-8-yl)oxy)triisopropylsilane (7b)

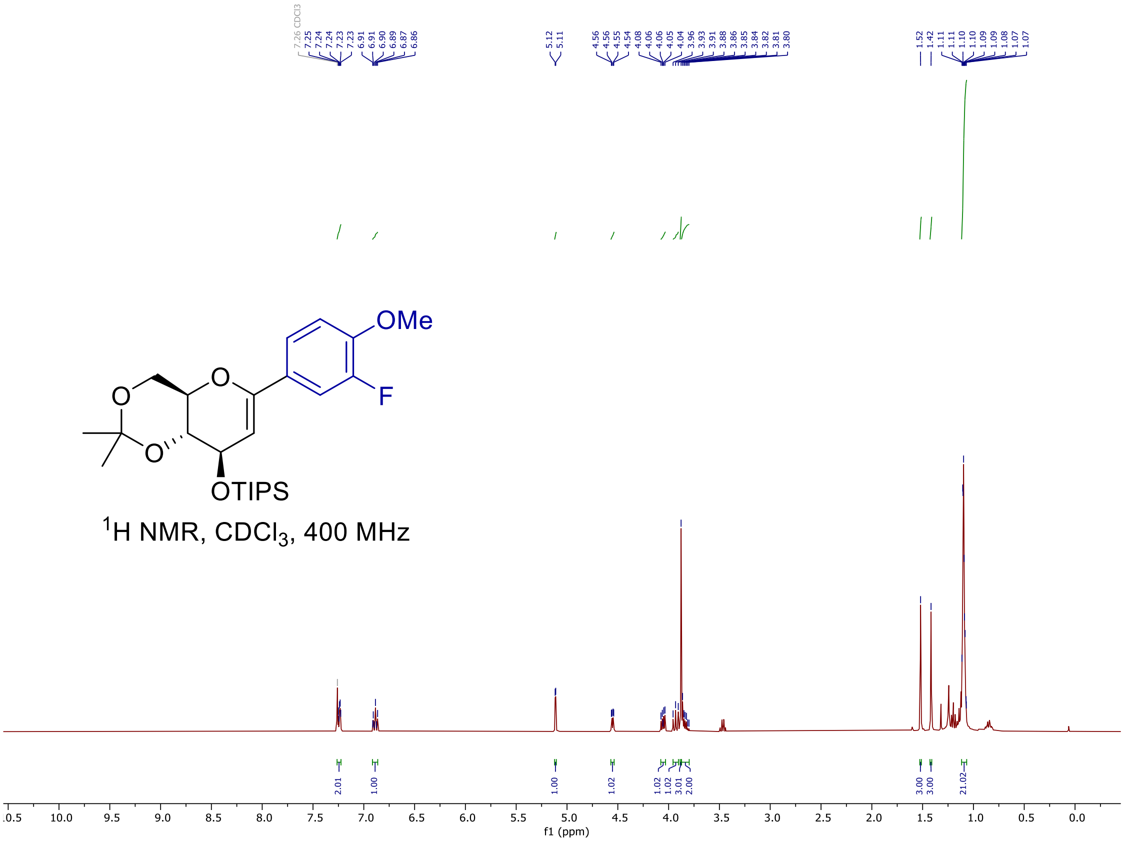

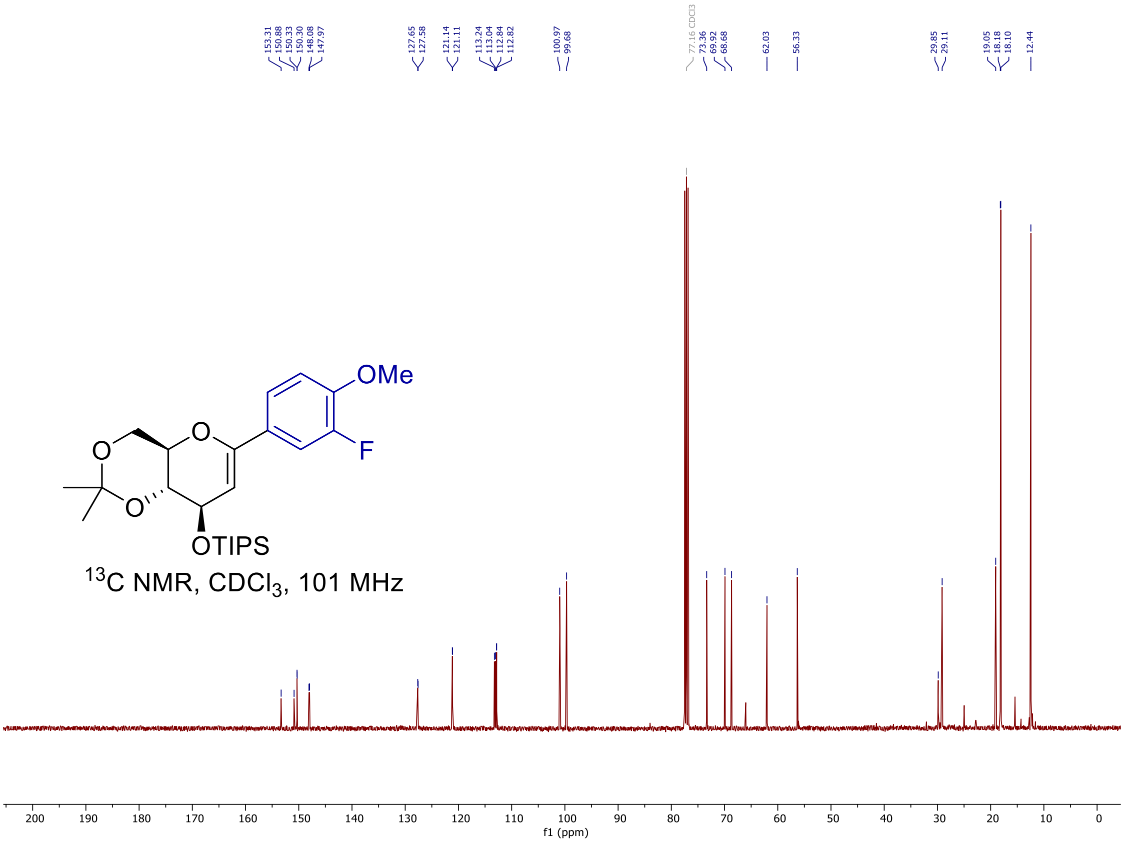

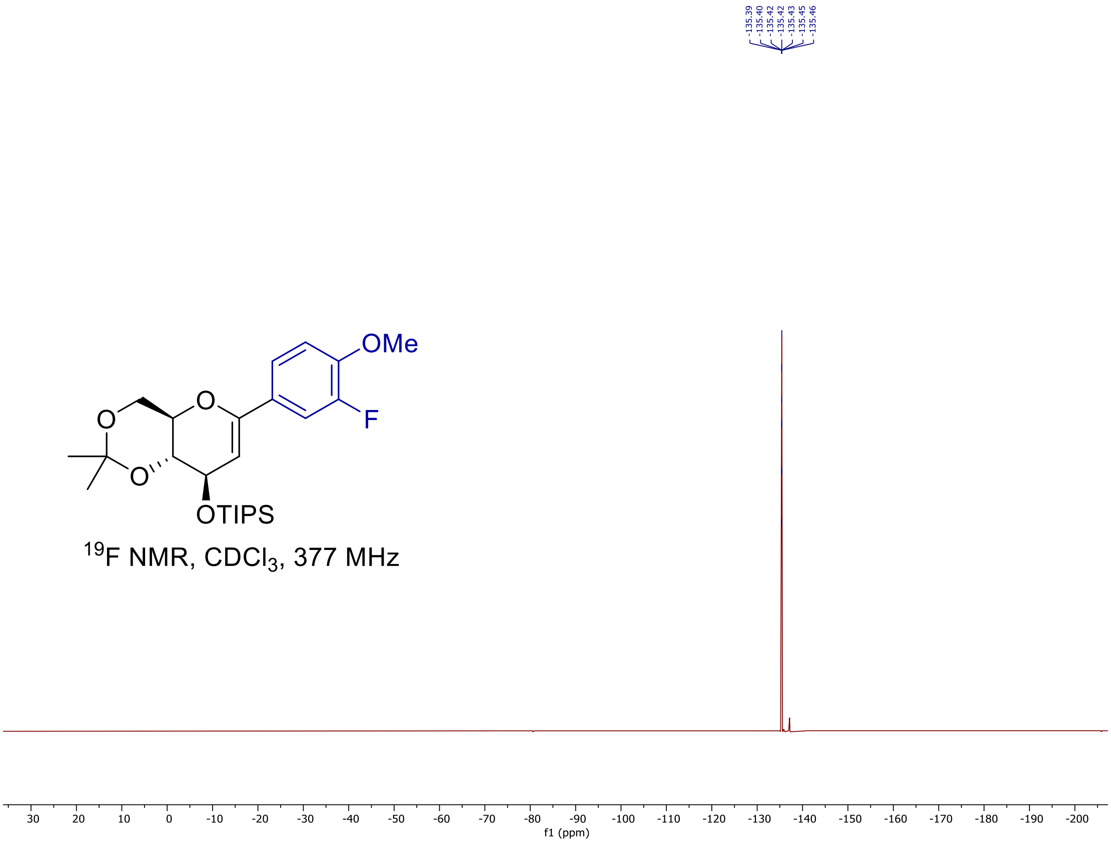
-135.39
-135.40
-135.42
-135.42
-135.43
-135.45
-135.46
OMe
F
O
O
O
OTIPS
^{19}F NMR, CDCl$_3$, 377 MHz
30 20 10 0 -10 -20 -30 -40 -50 -60 -70 -80 -90 -100 -110 -120 -130 -140 -150 -160 -170 -180 -190 -200
f1 (ppm)

(((4a*R*,8*R*,8a*R*)-2,2-Dimethyl-6-(2,3,4-trimethoxyphenyl)-4,4a,8,8a-tetrahydropyrano[3,2-*d*][1,3]dioxin-8-yl)oxy)triisopropylsilane (7c)

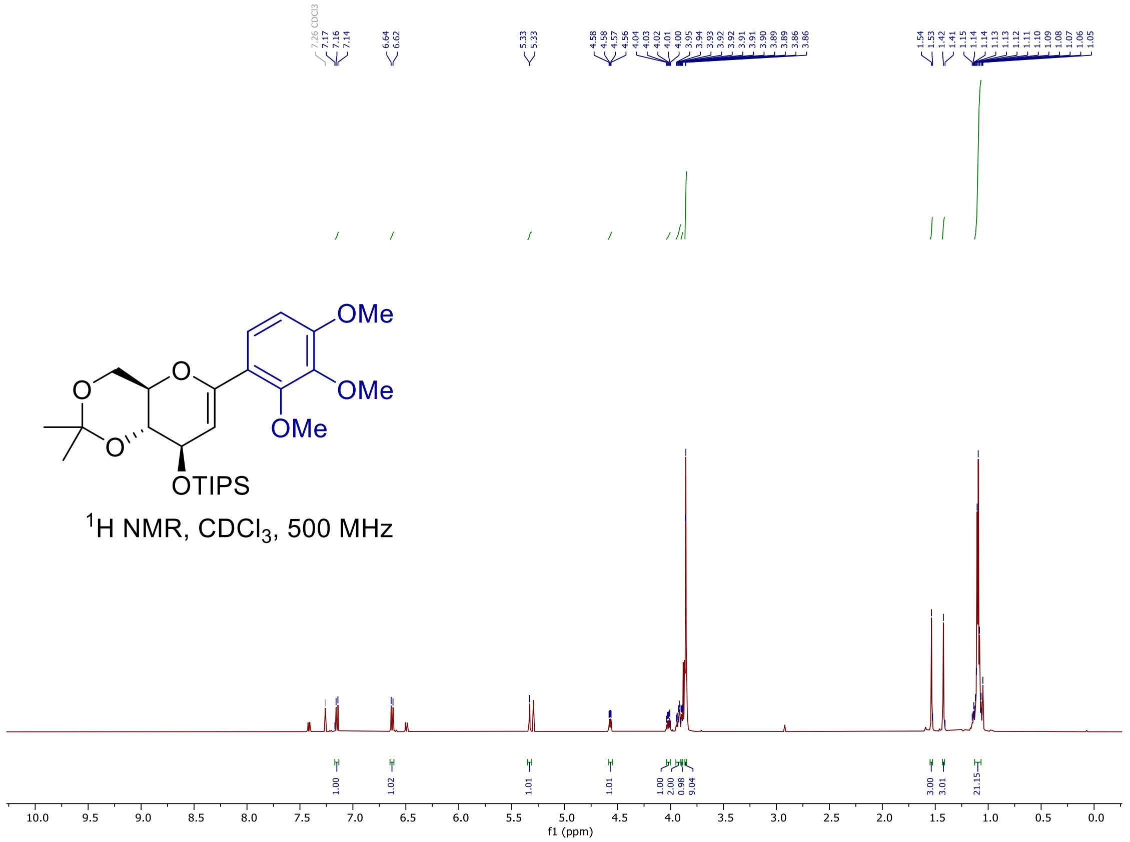

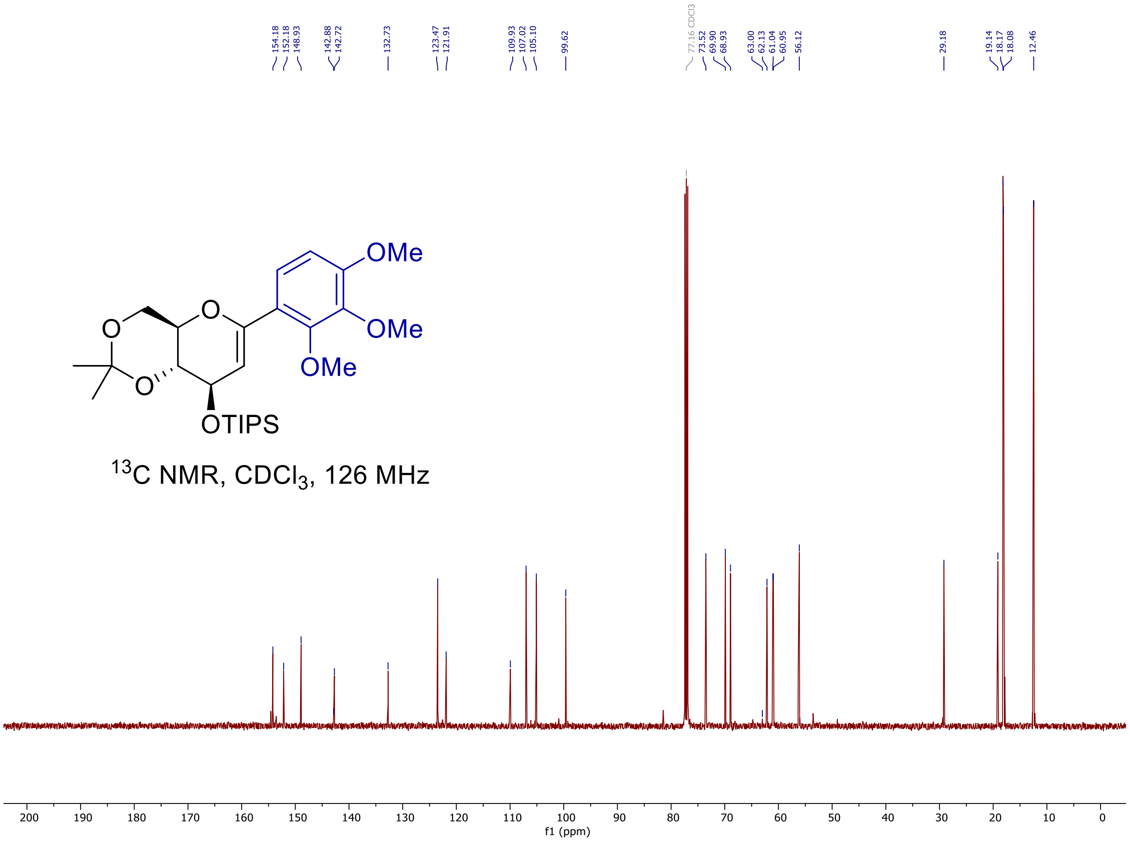

(((4a*R*,8*R*,8a*R*)-2,2-Dimethyl-6-phenyl-4,4a,8,8a-tetrahydropyrano[3,2-*d*][1,3]dioxin-8-yl)oxy)triisopropylsilane (7d)

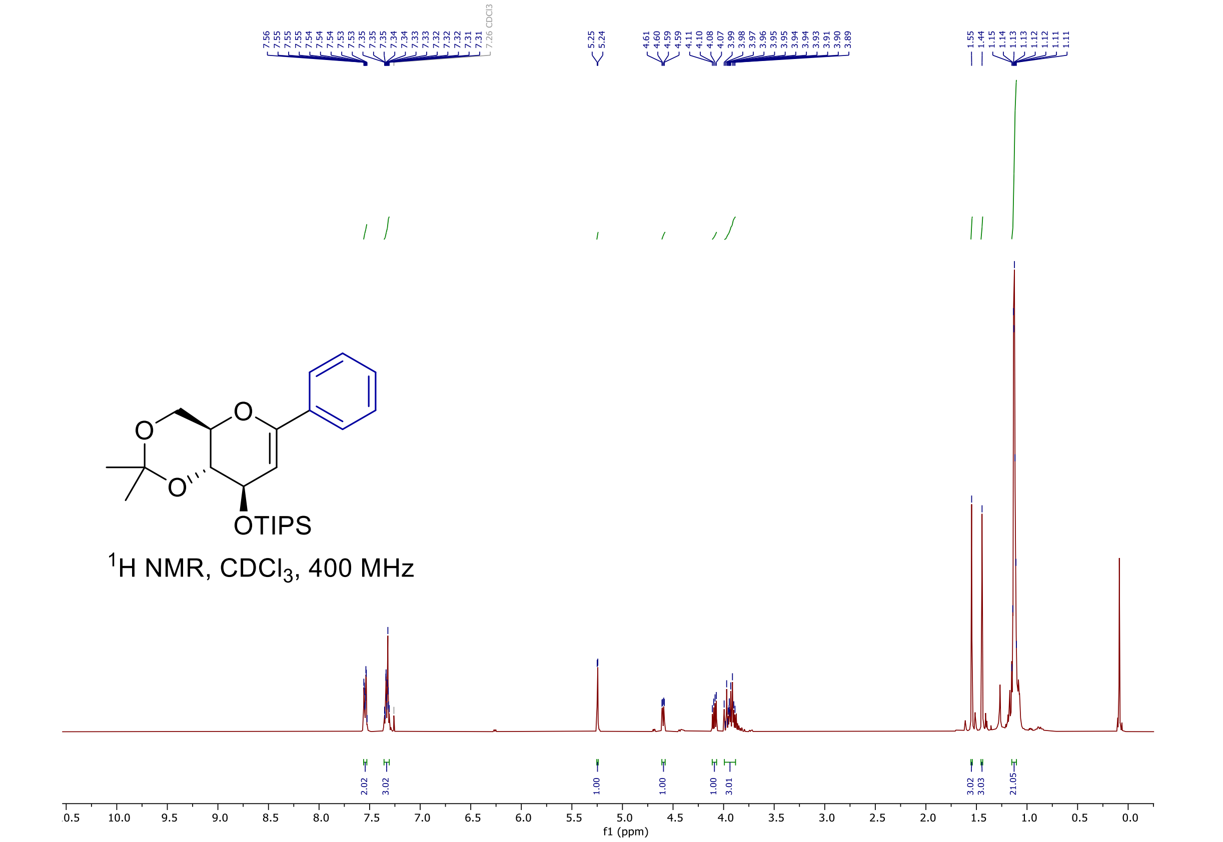

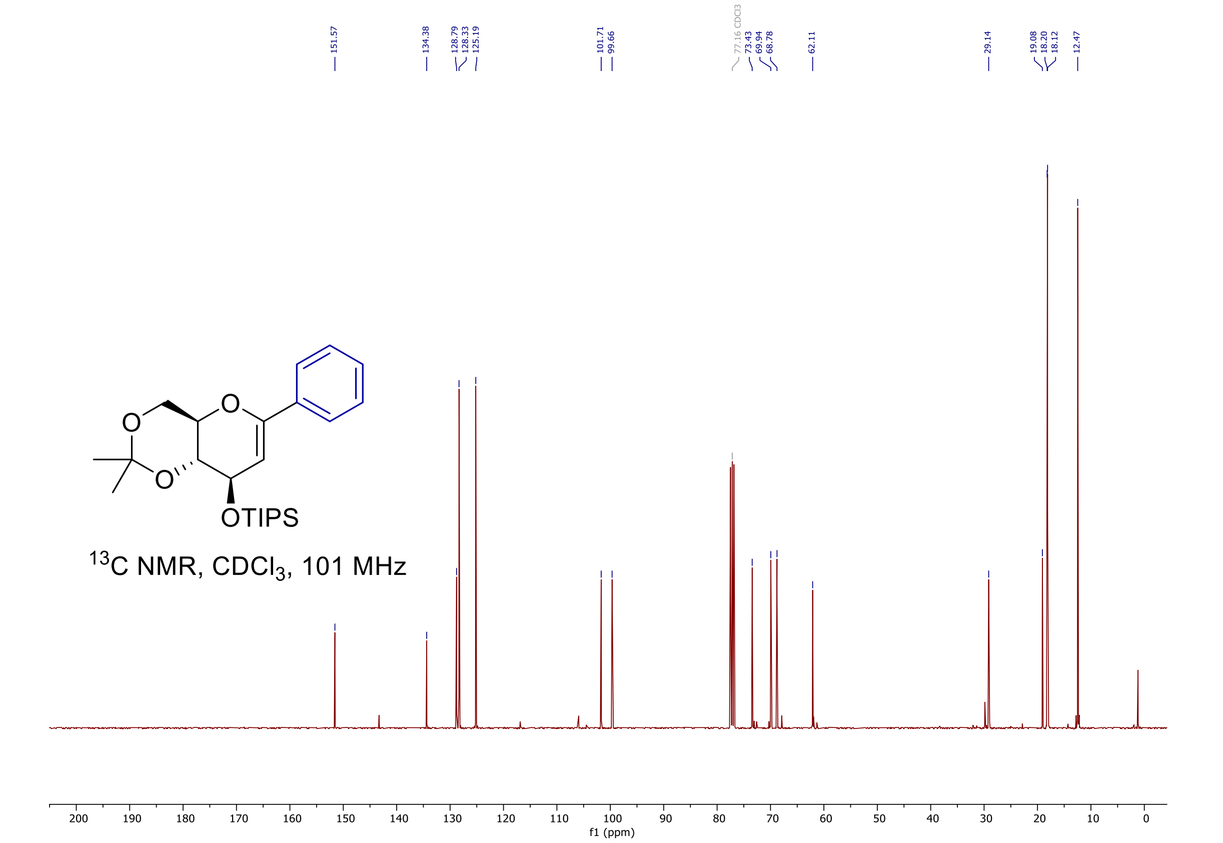

(((4a*R*,8*R*,8a*R*)-6-(6-Bromo-2,3,4-trimethoxyphenyl)-2,2-dimethyl-4,4a,8,8a-tetrahydropyrano[3,2-*d*][1,3]dioxin-8-yl)oxy)triisopropylsilane (7e)

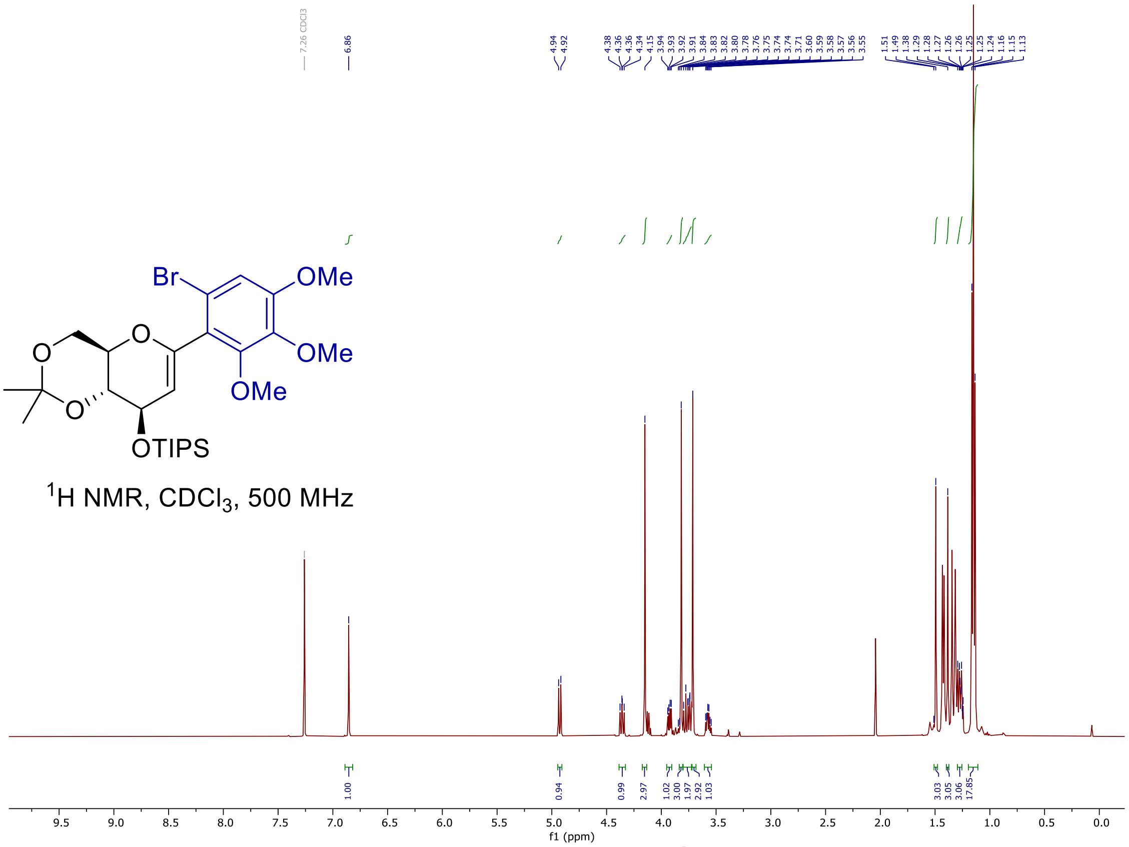

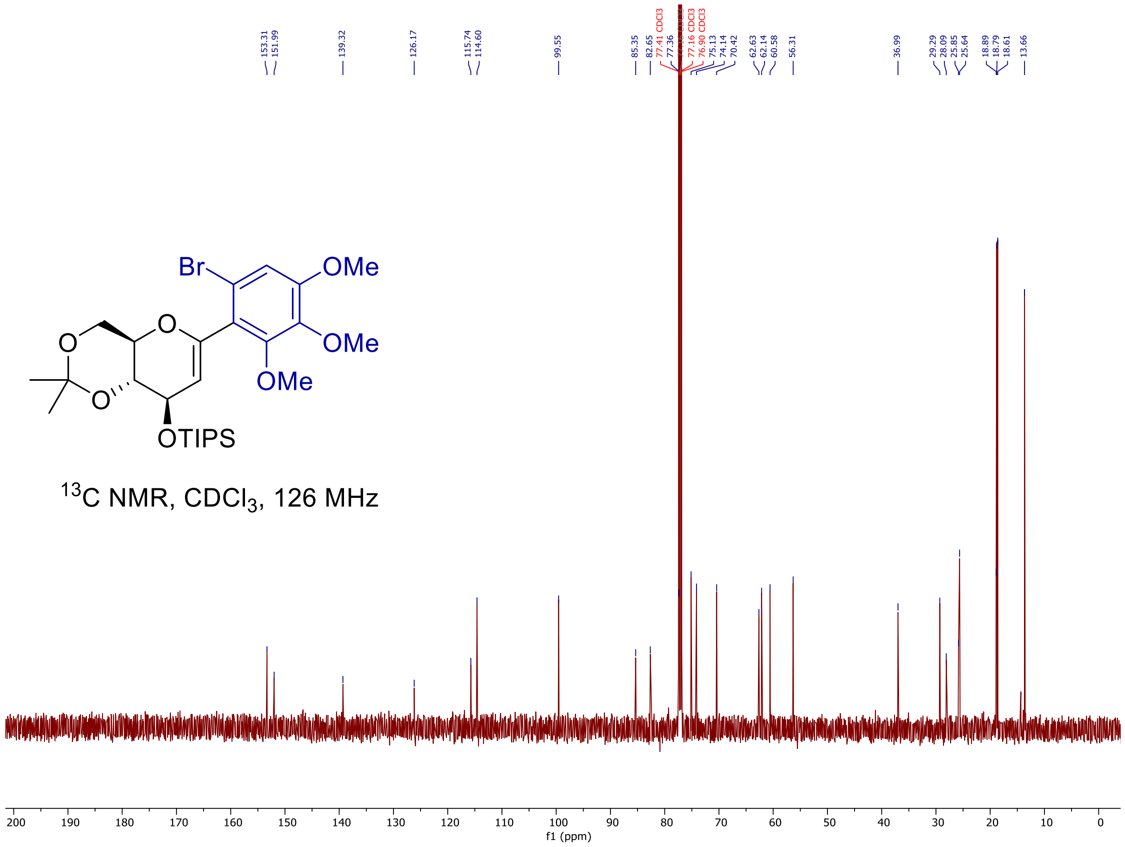

(((4a*R*,8*R*,8a*R*)-2,2-Dimethyl-6-vinyl-4,4a,8,8a-tetrahydropyrano[3,2-*d*][1,3]dioxin-8-yl)oxy)triisopropylsilane (7f)

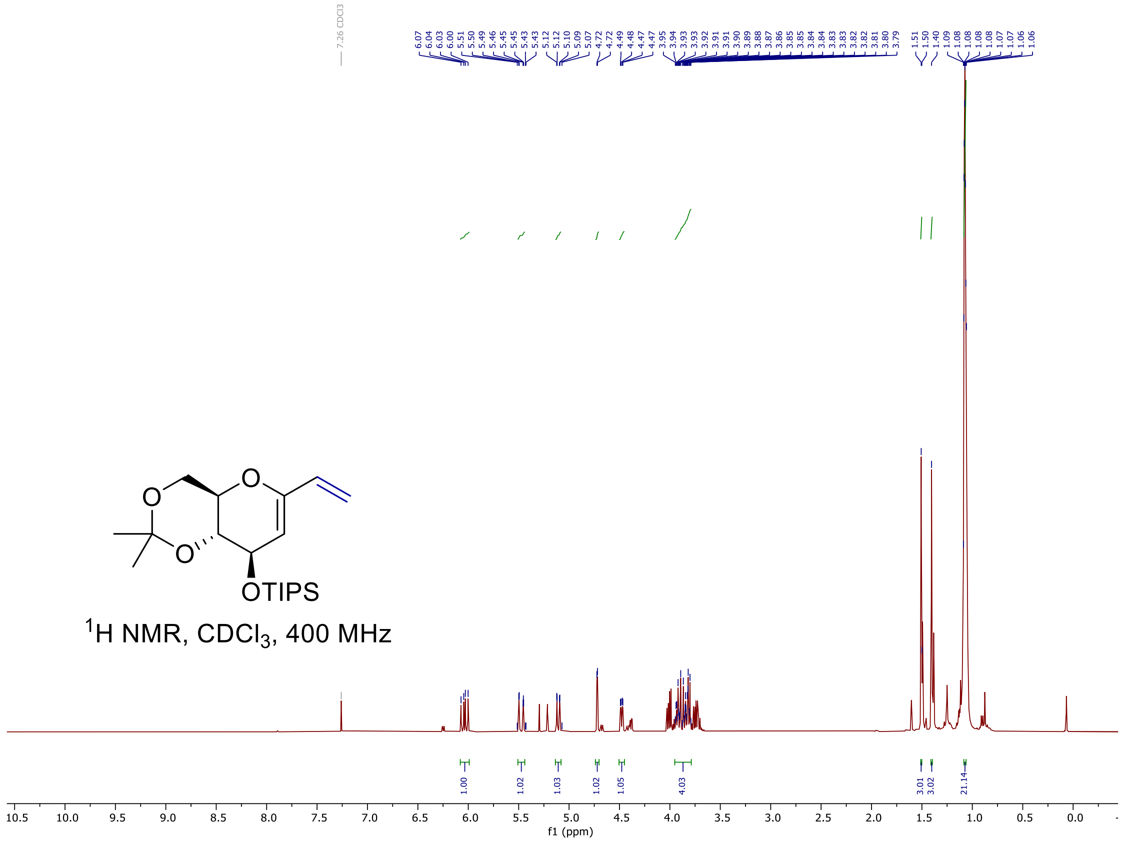

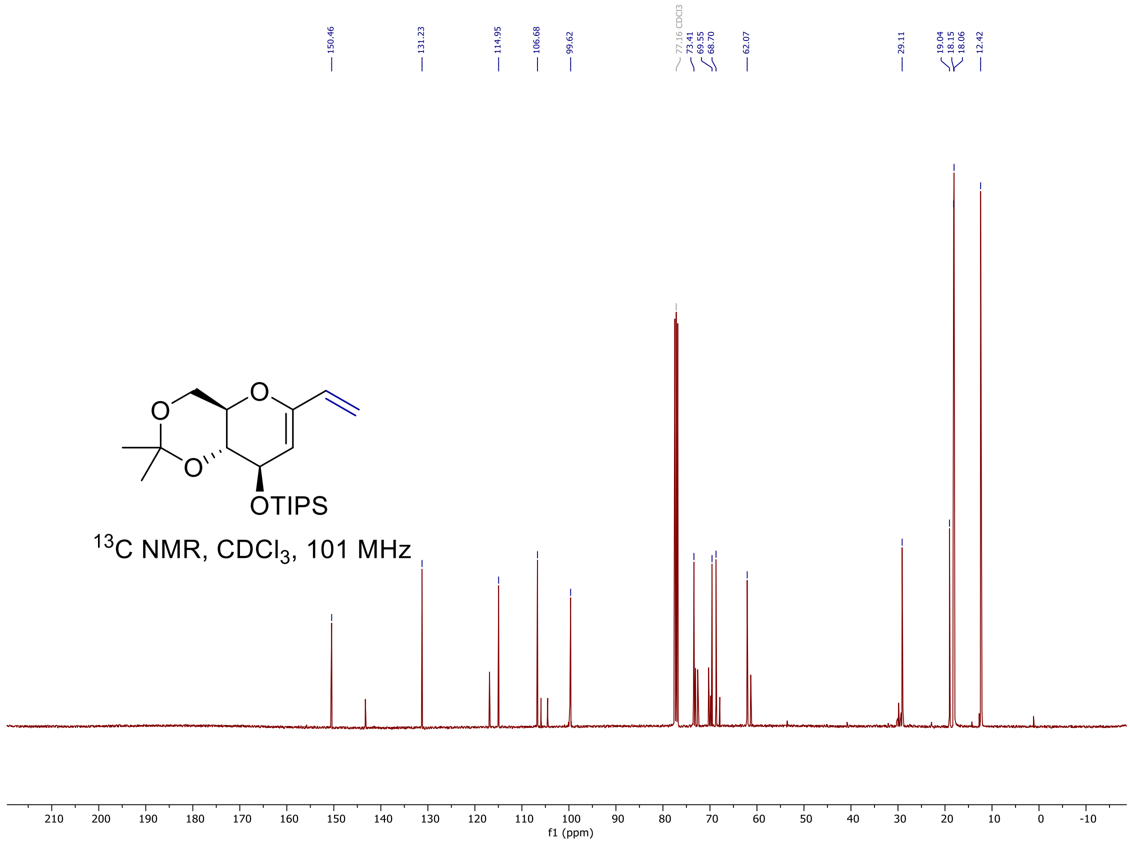

(((4a*R*,8*R*,8a*R*)-6-(3,5-Dichlorophenyl)-2,2-dimethyl-4,4a,8,8a-tetrahydropyrano[3,2-*d*][1,3]dioxin-8-yl)oxy)triisopropylsilane (7g)

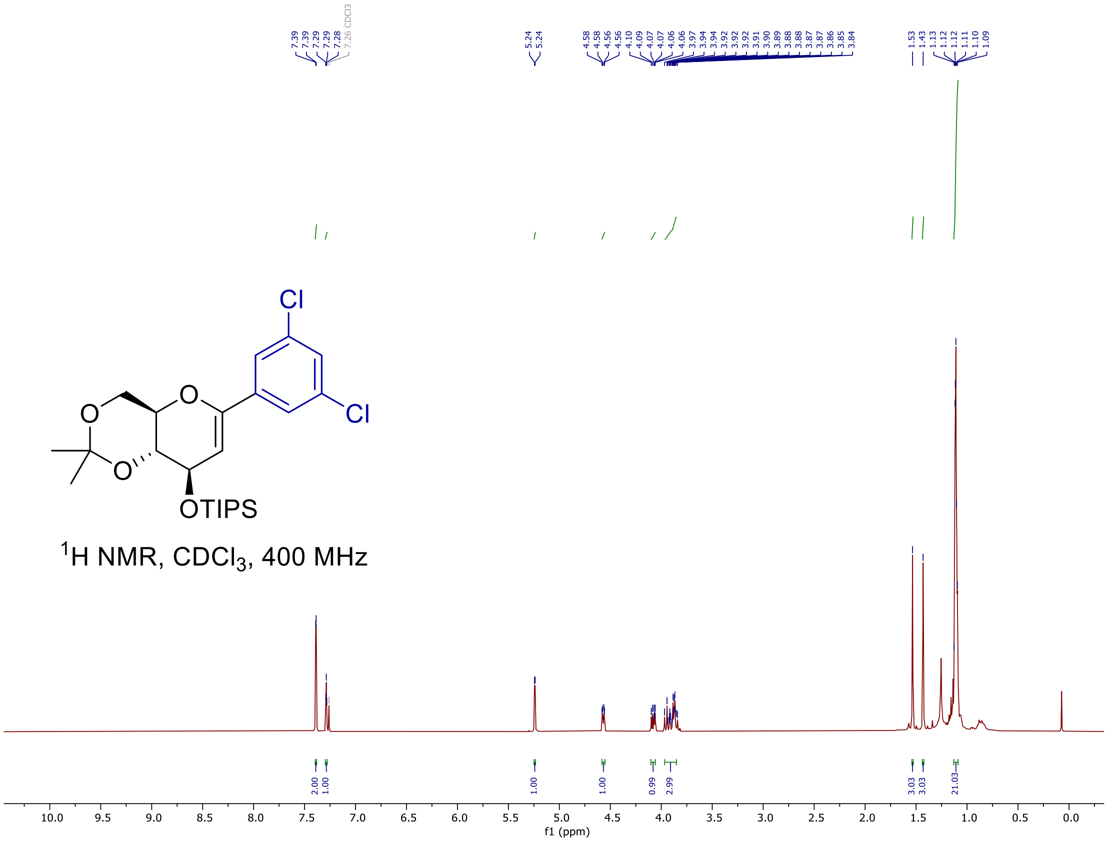

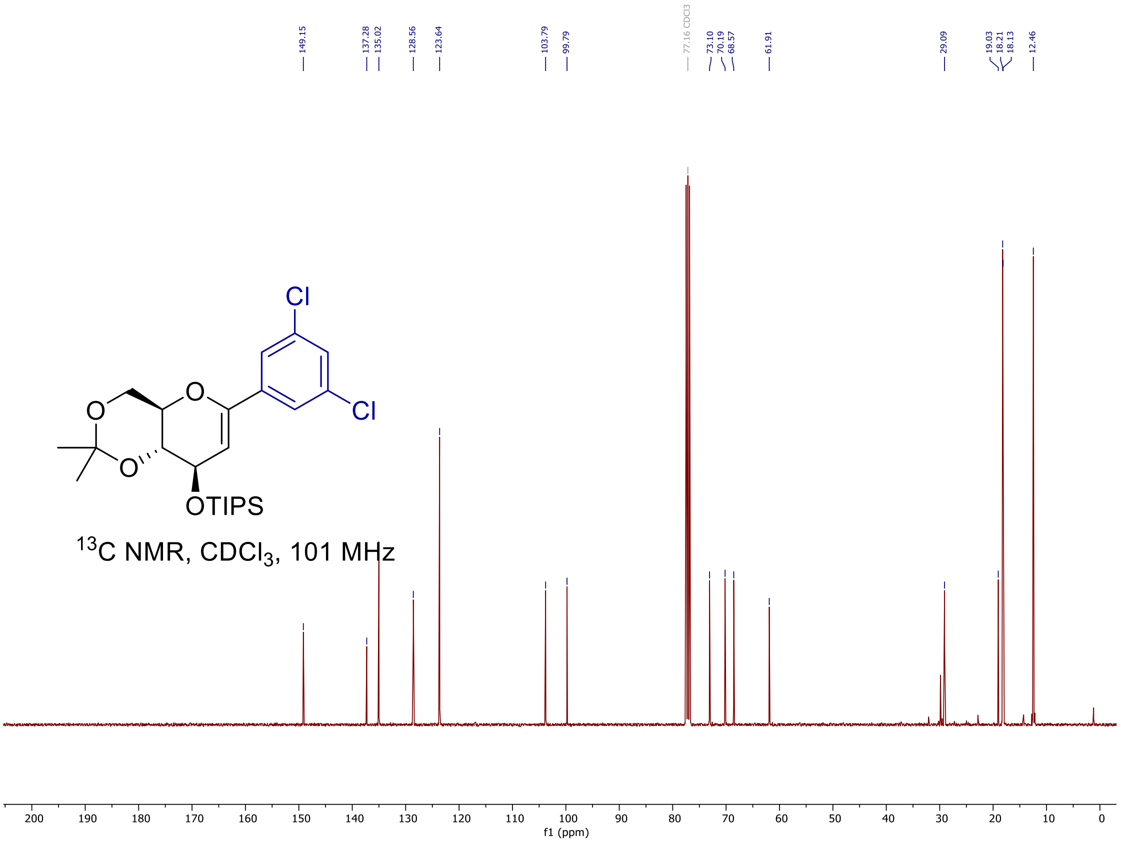

(((4a*R*,8*R*,8a*R*)-6-(4-Fluorophenyl)-2,2-dimethyl-4,4a,8,8a-tetrahydropyrano[3,2-*d*][1,3]dioxin-8-yl)oxy)triisopropylsilane (7h)

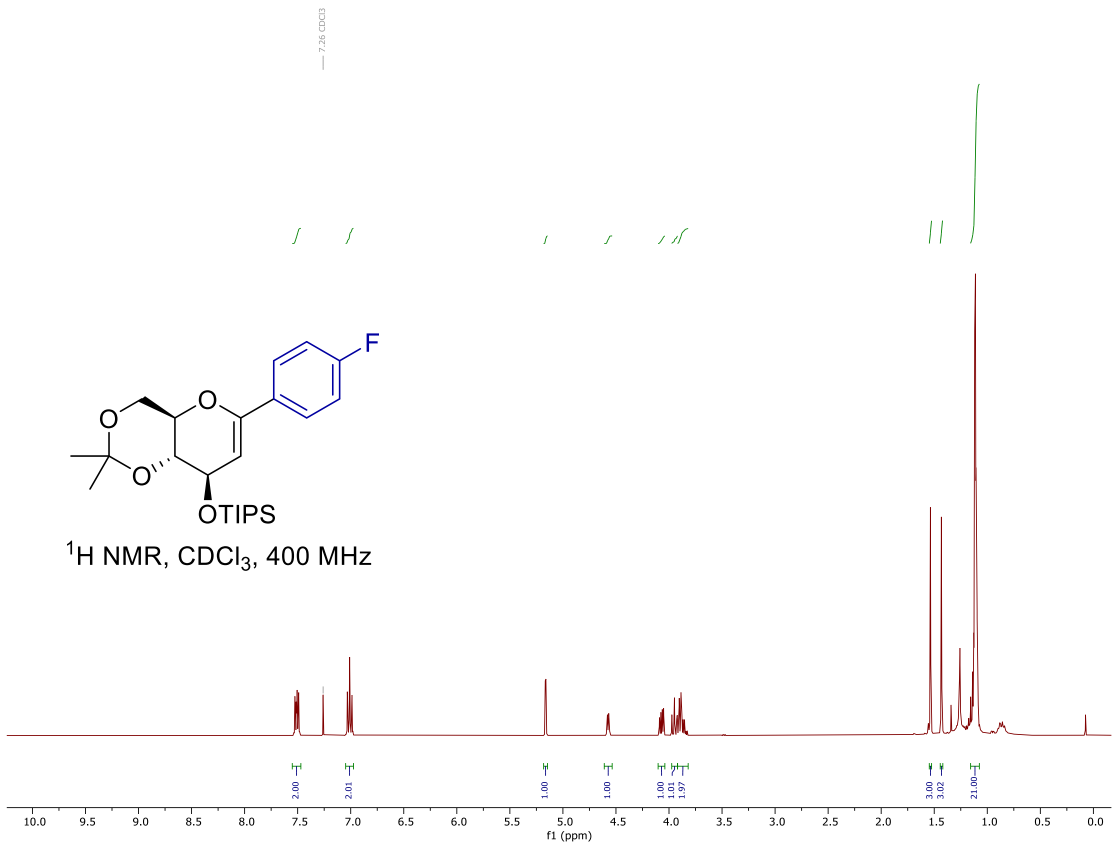

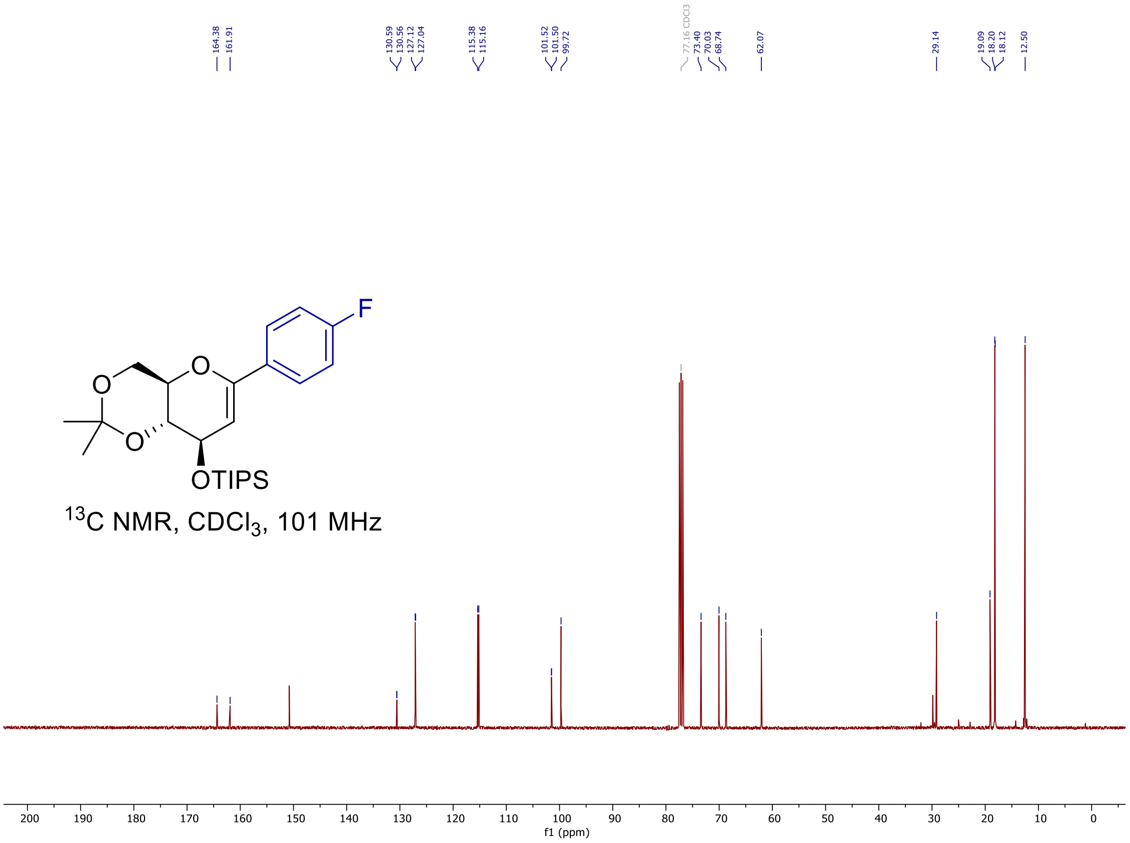

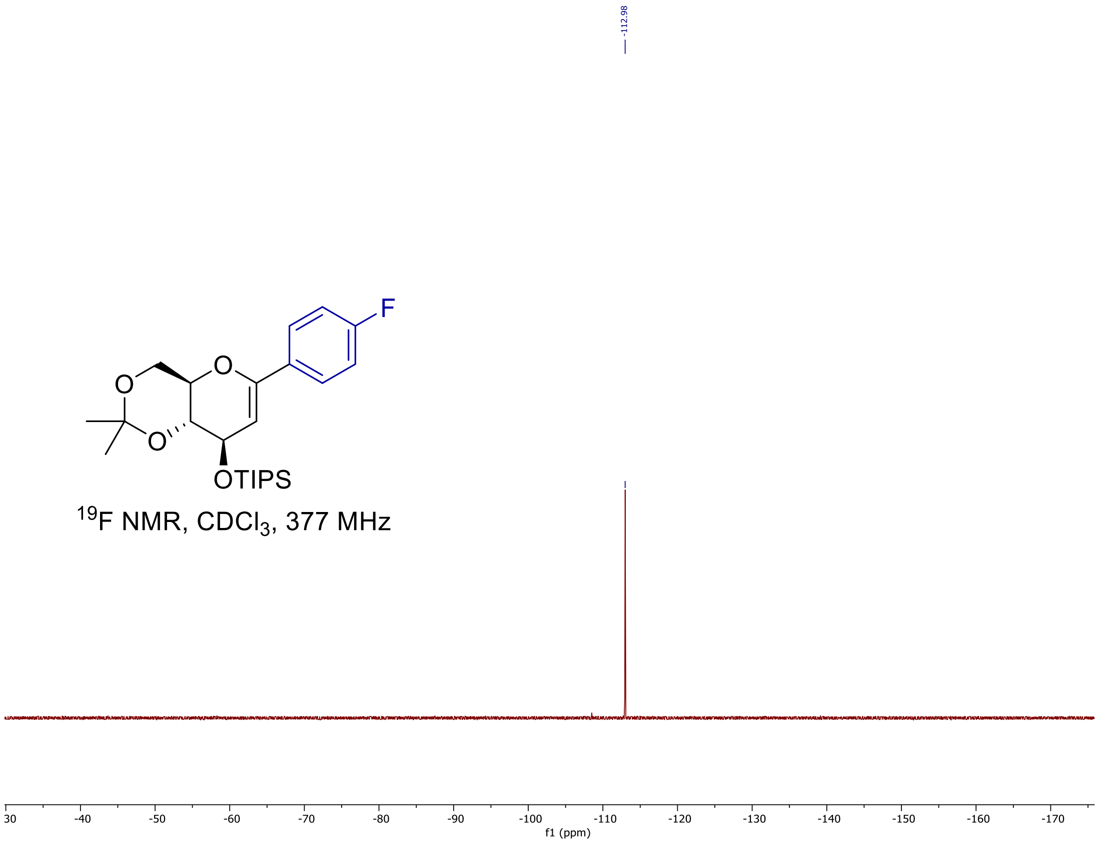

-112.98
F
O
O
O
OTIPS
19F NMR, CDCl3, 377 MHz
30
-40
-50
-60
-70
-80
-90
-100
-110
-120
-130
-140
-150
-160
-170
f1 (ppm)

3-((4a*R*,8*R*,8a*R*)-2,2-Dimethyl-8-((triisopropylsilyl)oxy)-4,4a,8,8a-tetrahydropyrano[3,2-*d*][1,3]dioxin-6-yl)benzonitrile (7i)

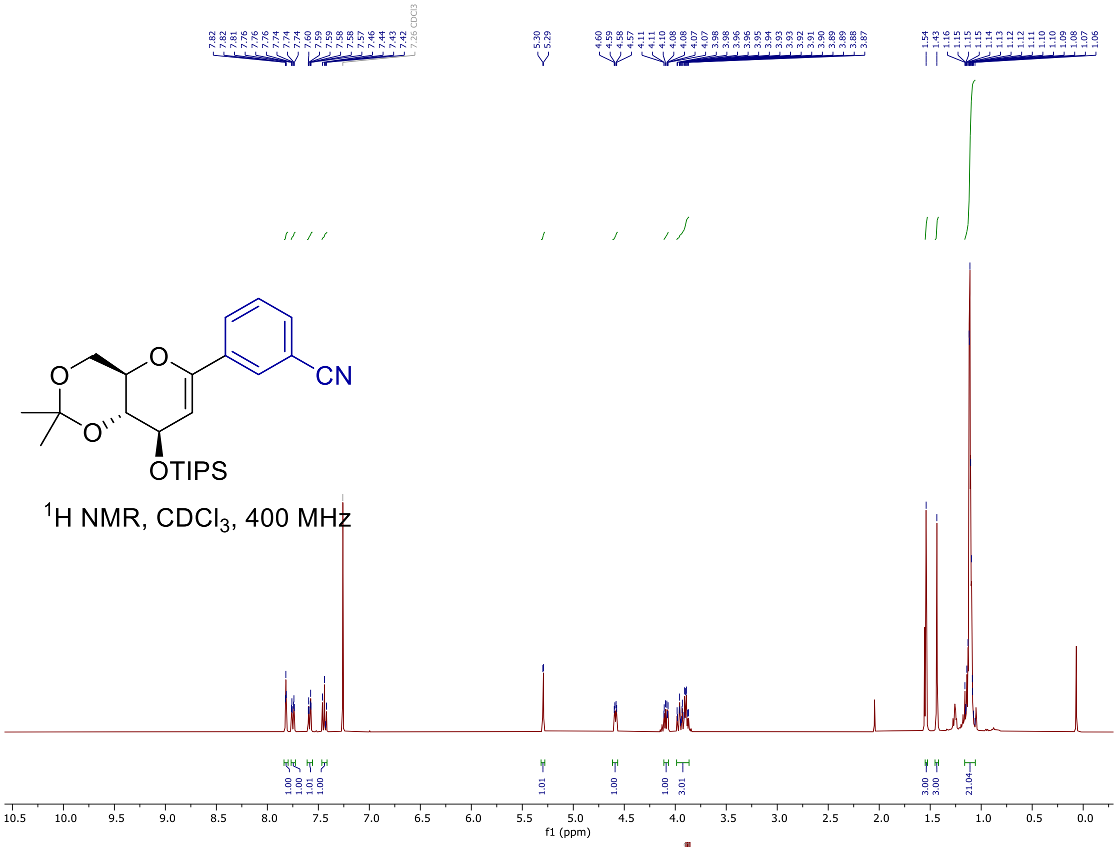

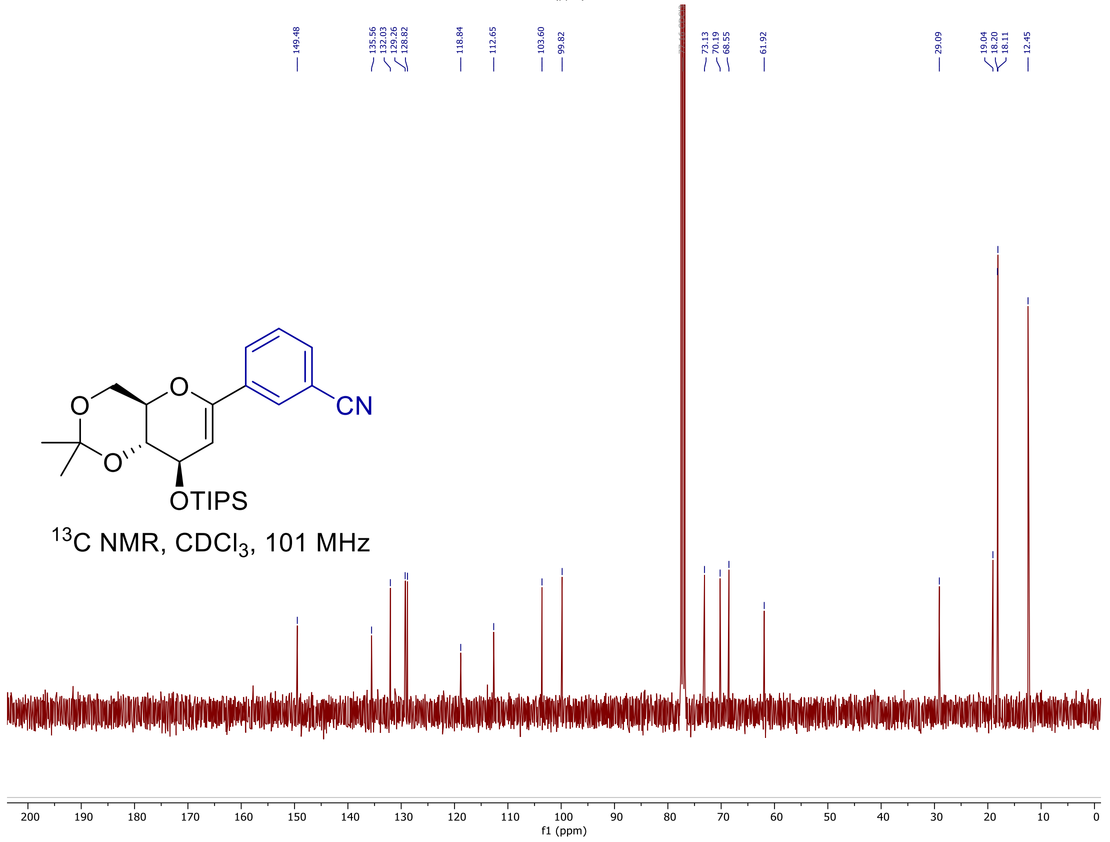

(((4a*R*,8*R*,8a*R*)-2,2-Dimethyl-6-(3-nitrophenyl)-4,4a,8,8a-tetrahydropyrano[3,2-*d*][1,3]dioxin-8-yl)oxy)triisopropylsilane (7j)

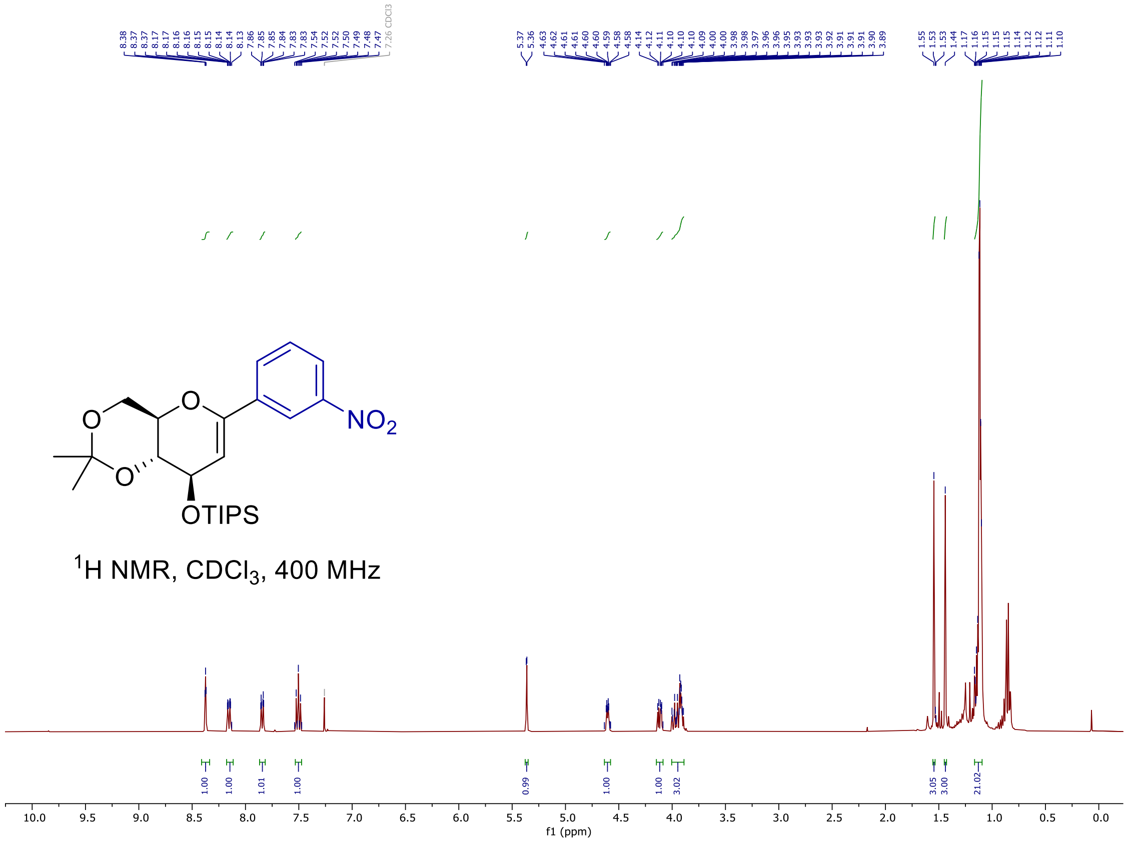

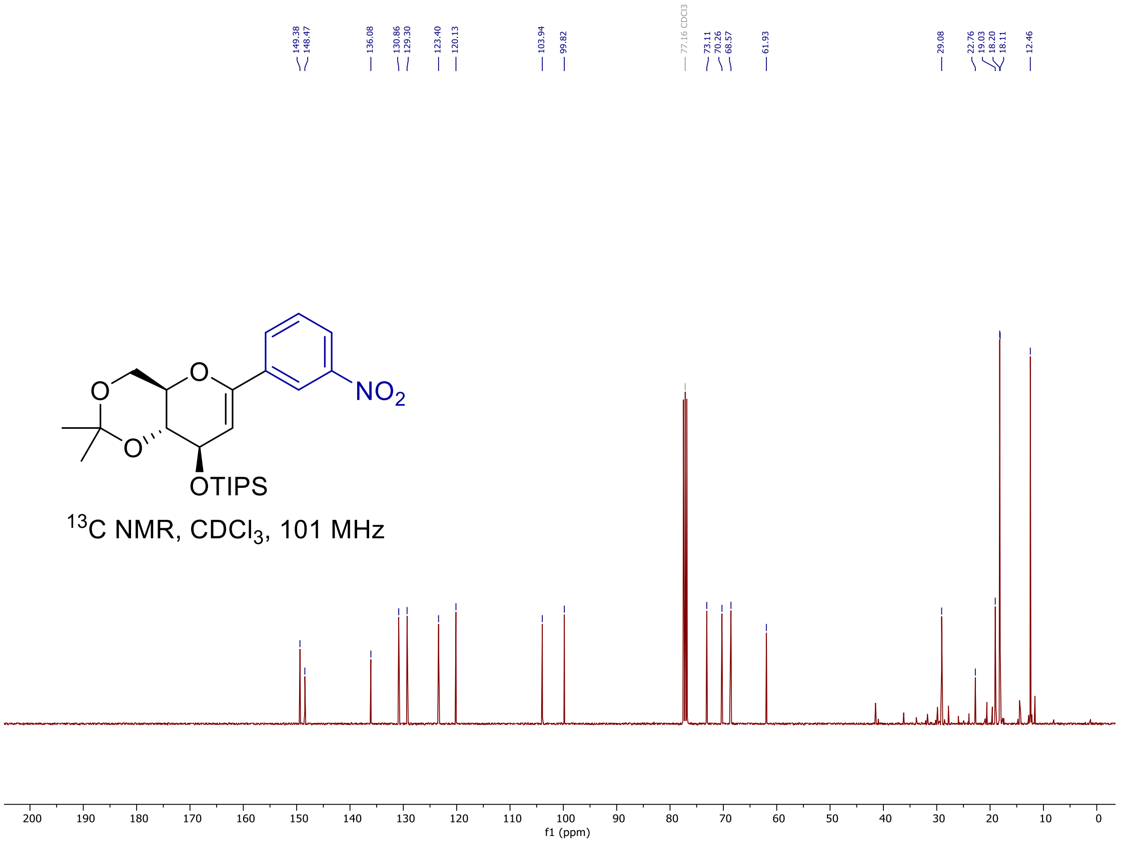

(((4a*R*,8*R*,8a*R*)-6-(Benzo[*d*][1,3]dioxol-4-yl)-2,2-dimethyl-4,4a,8,8a-tetrahydropyrano[3,2-*d*][1,3]dioxin-8-yl)oxy)triisopropylsilane (7k)

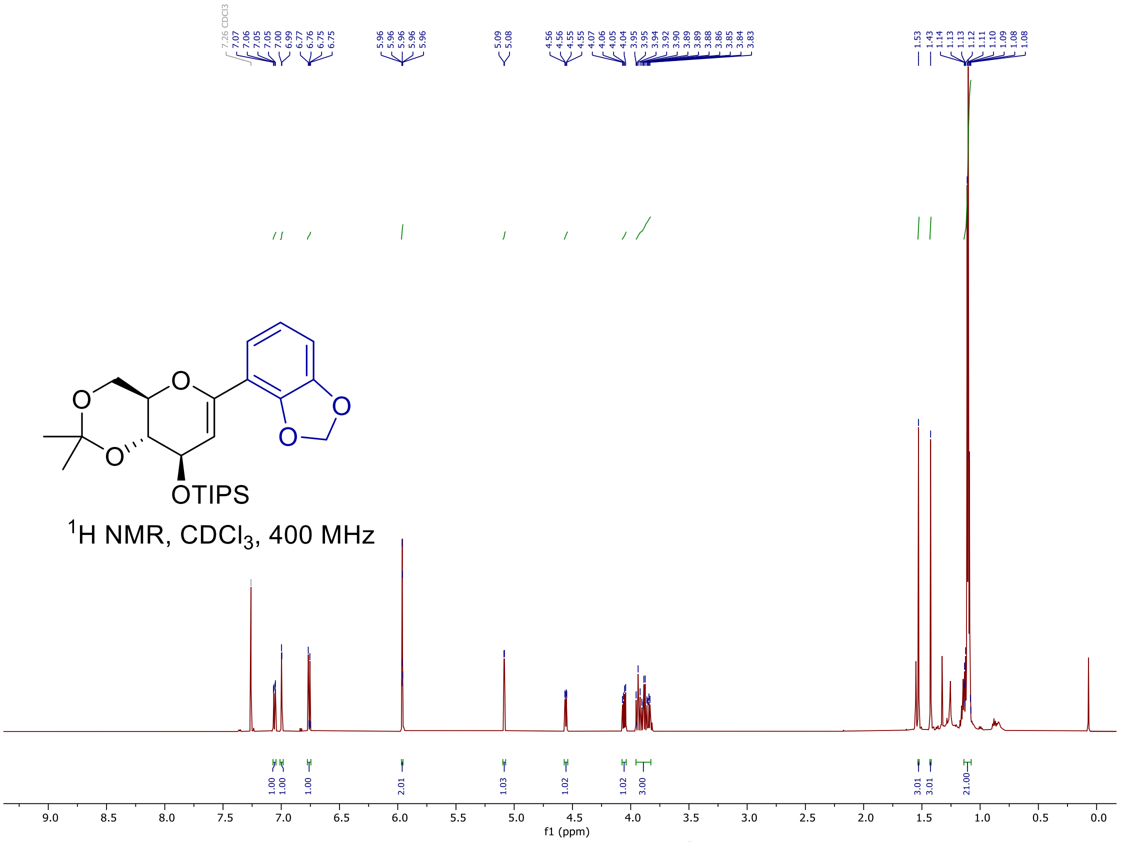

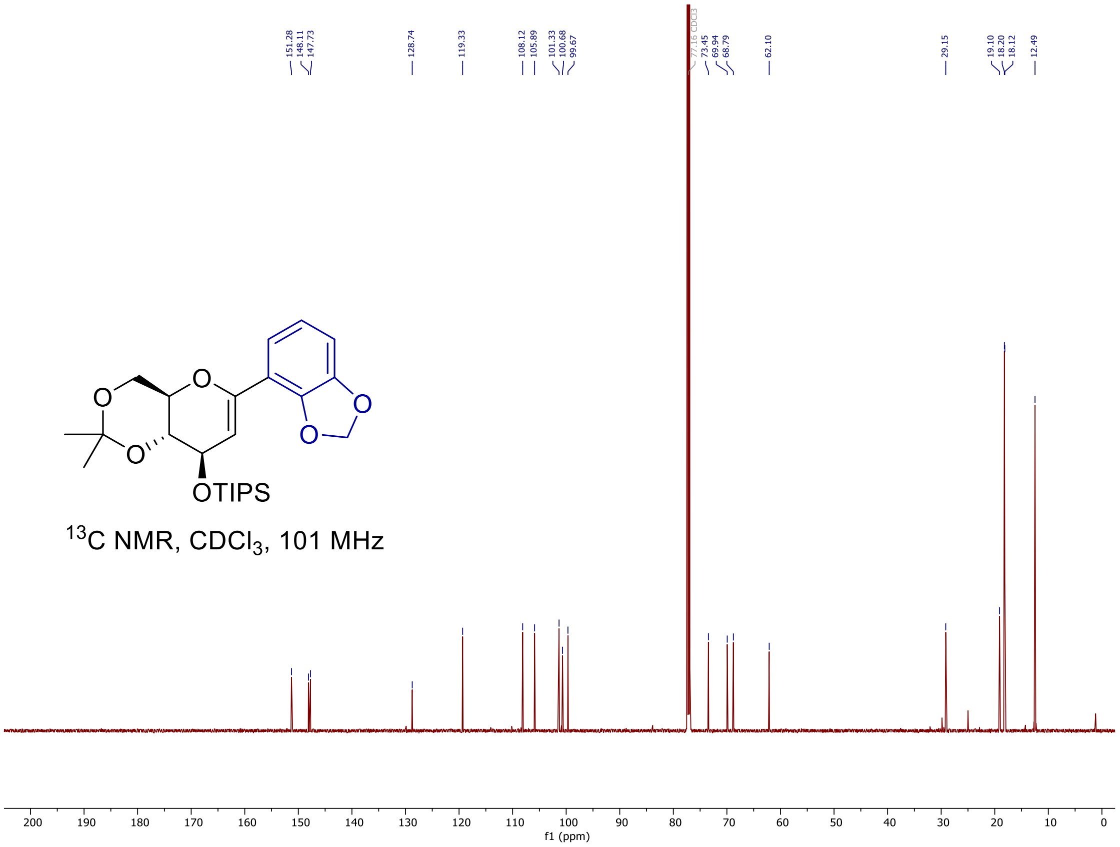

(*E*)-1-(4-((4a*R*,8*R*,8a*R*)-2,2-Dimethyl-8-((triisopropylsilyl)oxy)-4,4a,8,8a-tetrahydropyrano[3,2-*d*][1,3]dioxin-6-yl)phenyl)-2-phenyldiazene (<u>7l</u>)

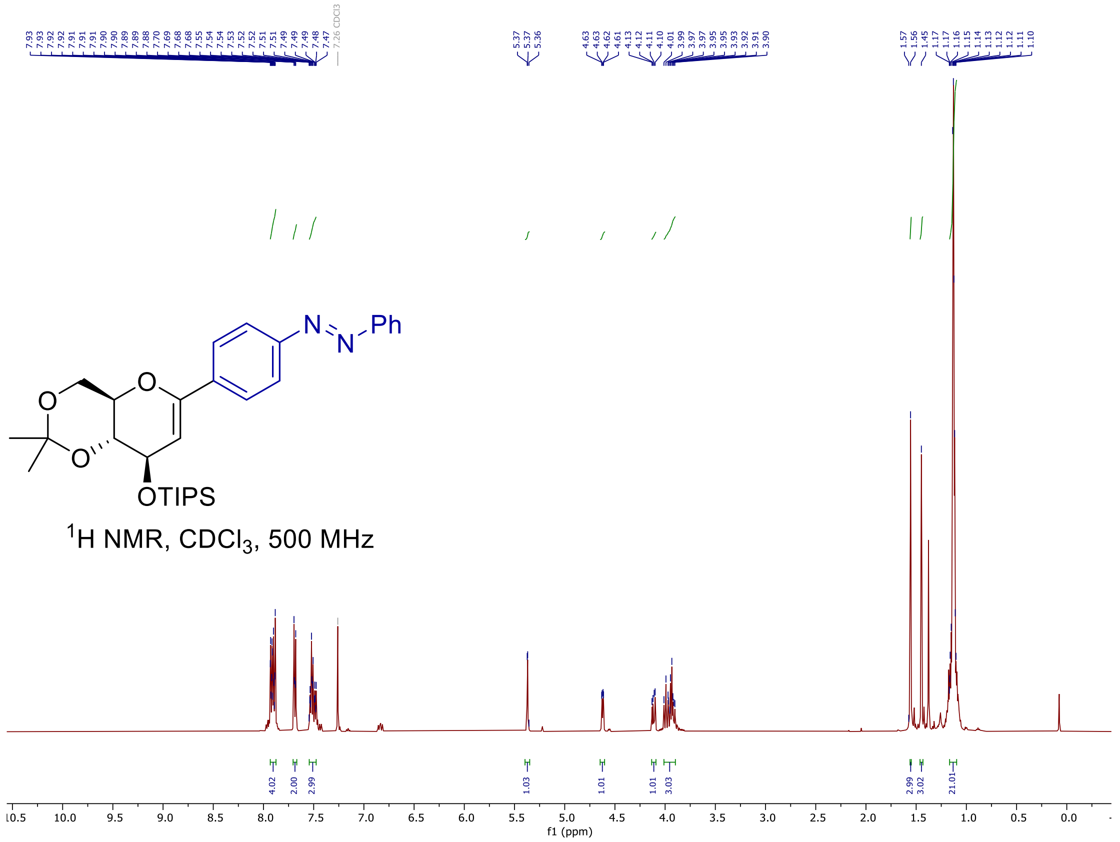

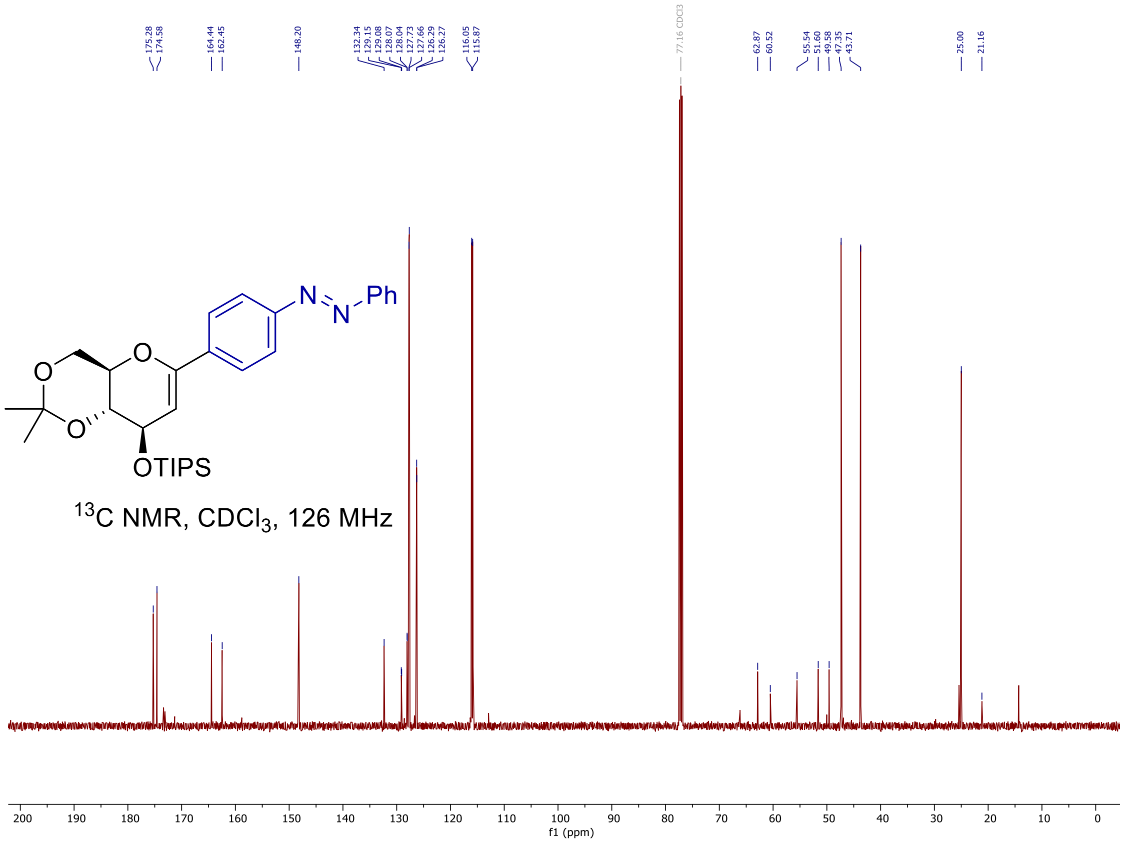

(((4a*R*,8*R*,8a*R*)-6-(4-Chloro-3-(4-(((S)-tetrahydrofuran-3-yl)oxy)benzyl)phenyl)-2,2-dimethyl-4,4a,8,8a-tetrahydropyrano[3,2-*d*][1,3]dioxin-8-yl)oxy)-triisopropylsilane (7m)

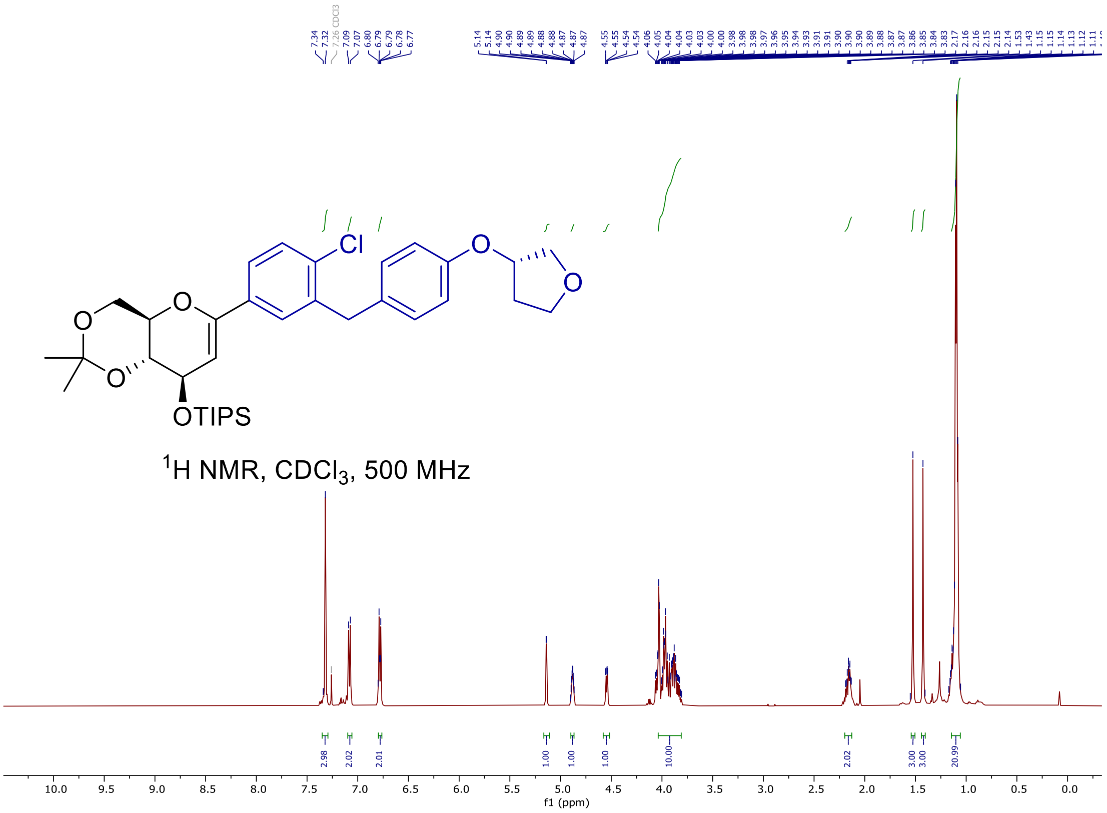

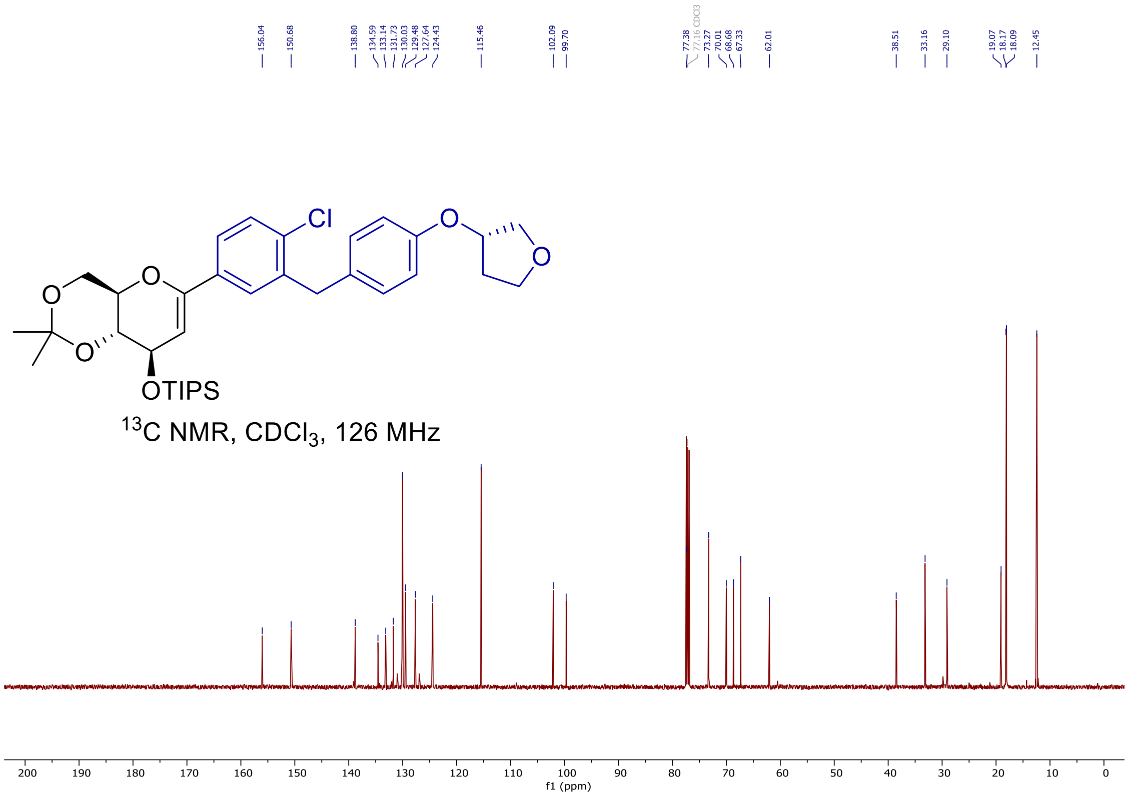

(4a*R*,8*R*,8a*R*)-8-((l1-silyl)oxy)-2,2-Di-*tert*-butyl-6-(naphthalen-1-yl)-4,4a,8,8a-tetrahydropyrano[3,2-*d*][1,3,2]dioxasiline (8a)

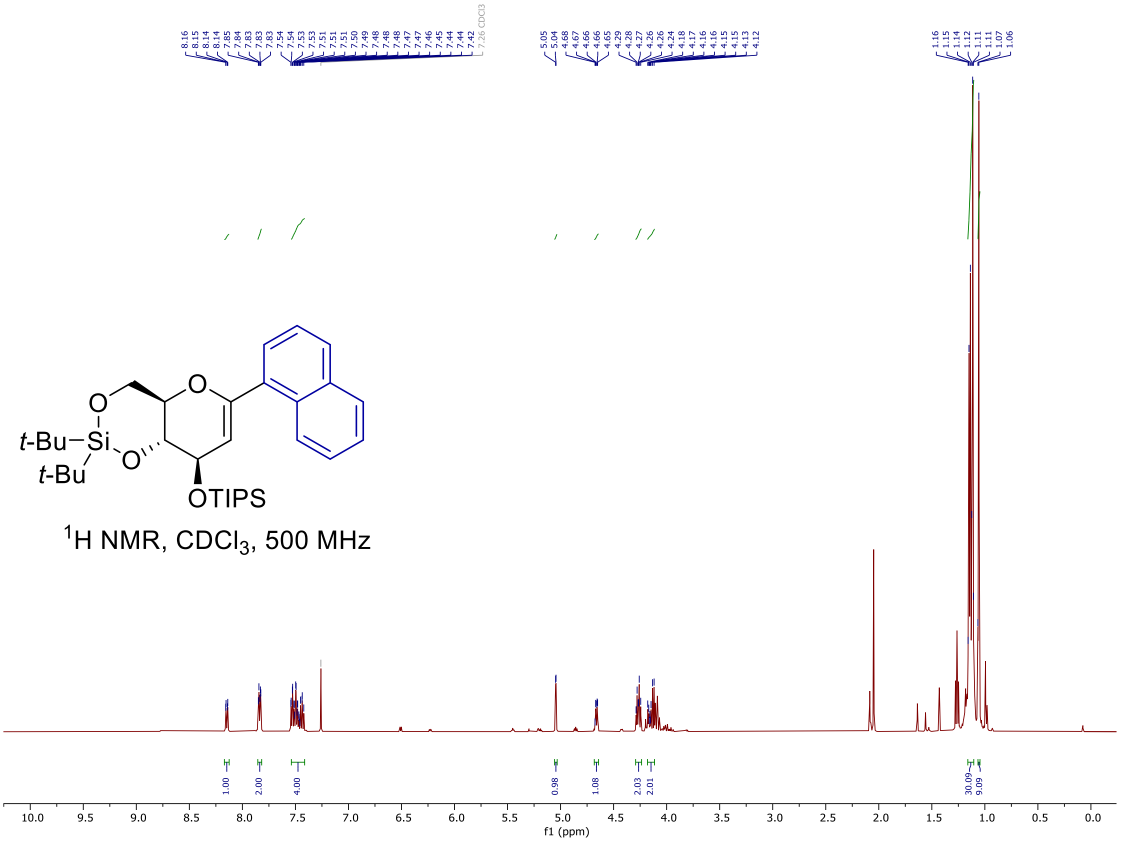

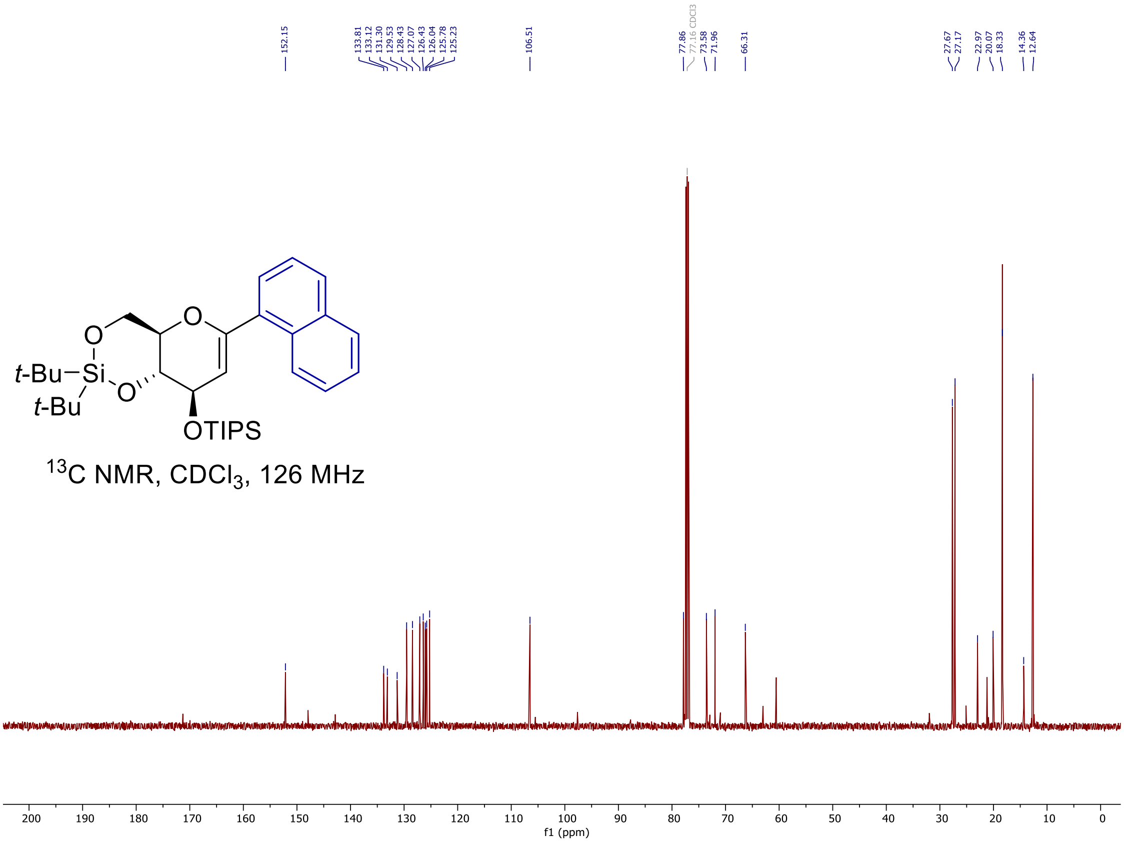

(4a*R*,8*R*,8a*R*)-8-((l1-Silyl)oxy)-2,2-di-*tert*-butyl-6-(2,3,4-trimethoxyphenyl)-4,4a,8,8a-tetrahydropyrano[3,2-*d*][1,3,2]dioxasiline (8b)

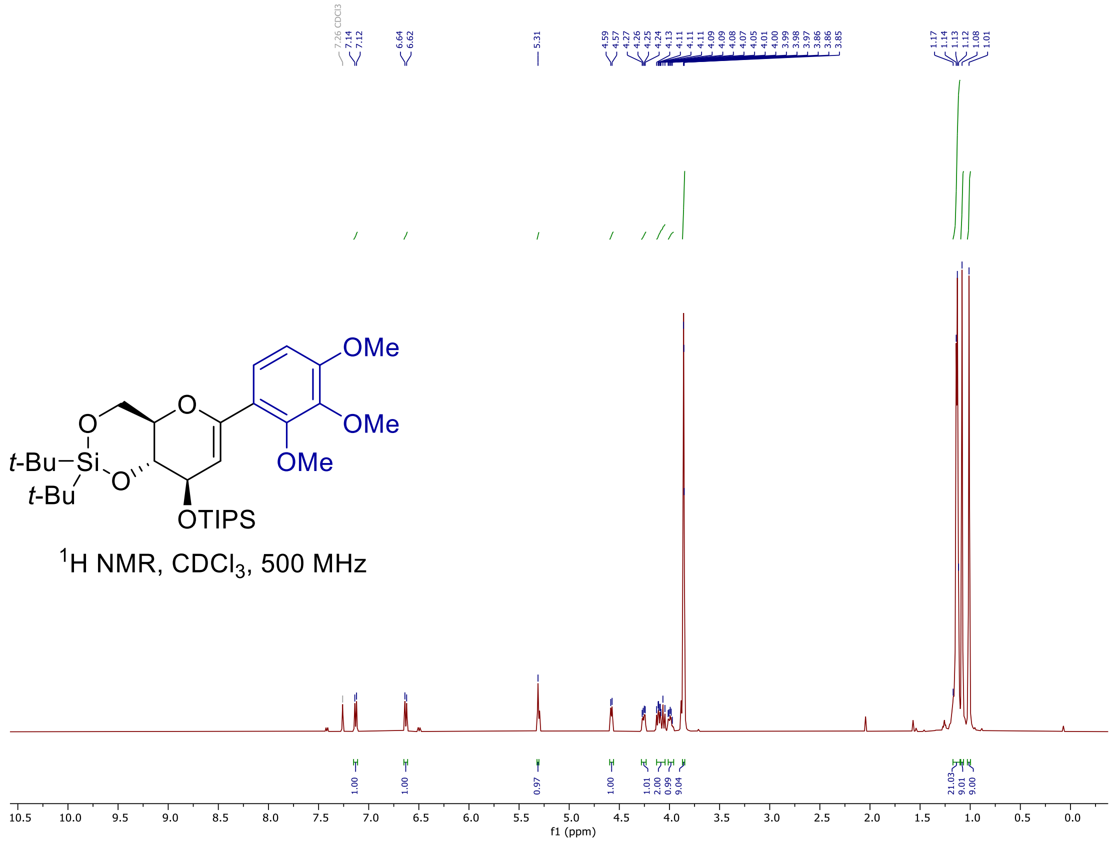

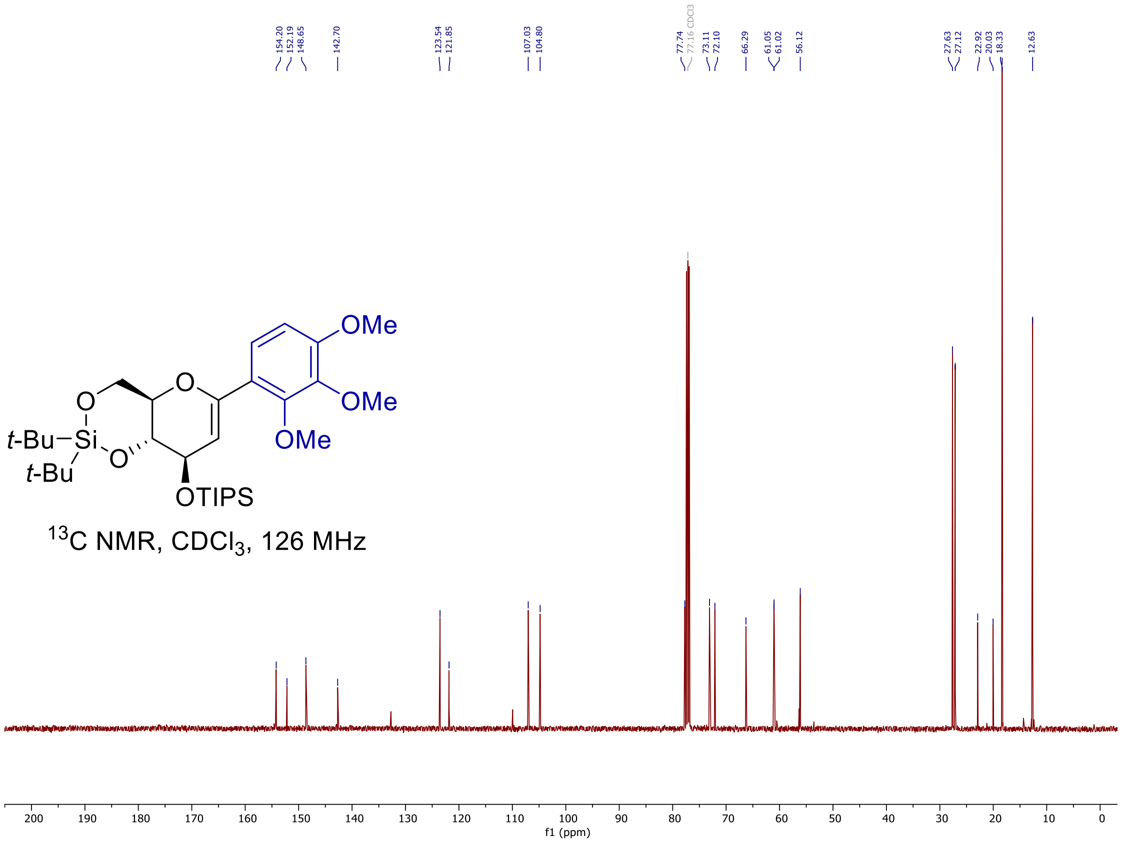

(4a*R*,8*R*,8a*R*)-6-(6-Bromo-2,3,4-trimethoxyphenyl)-2,2-di-*tert*-butyl-8-((triisopropylsilyl)oxy)-4,4a,8,8a-tetrahydropyrano[3,2-*d*][1,3,2]dioxasiline (8c)

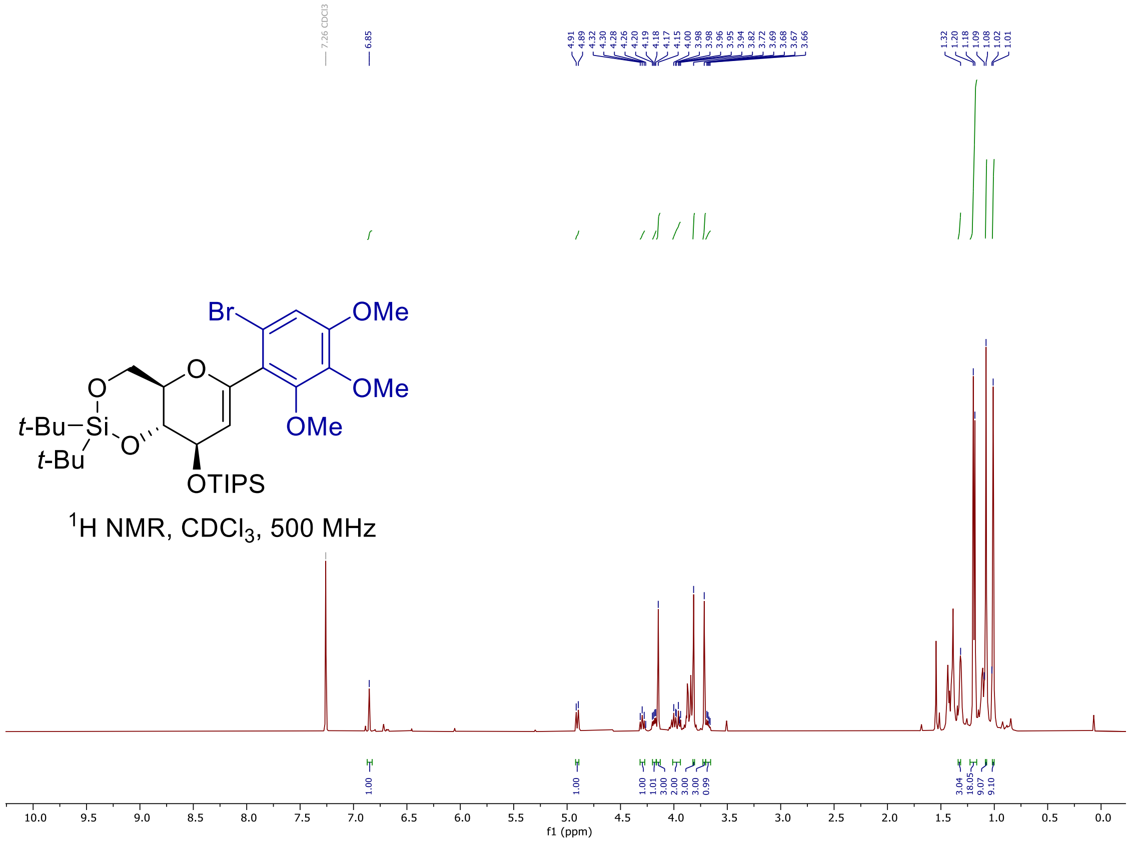

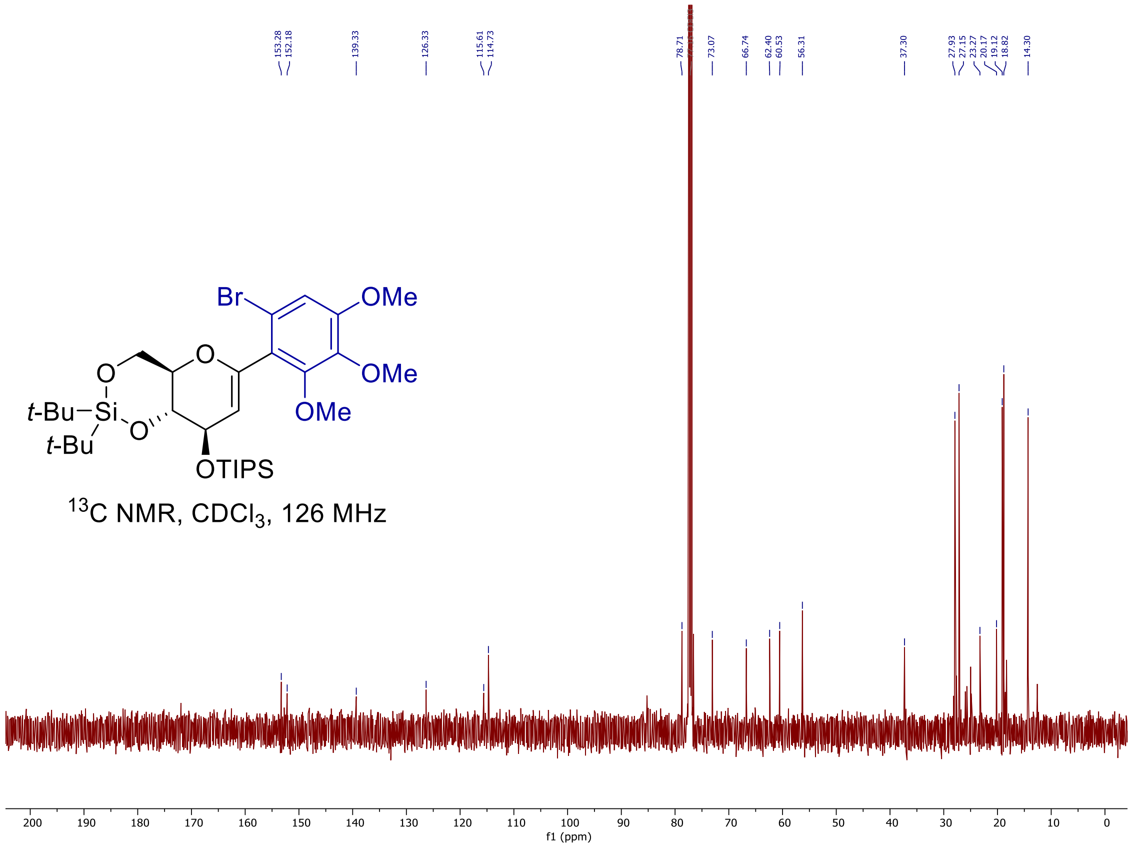

(4a*R*,8*R*,8a*R*)-8-((l1-Silyl)oxy)-2,2-di-*tert*-butyl-6-(3,6-dihydro-2*H*-pyran-4-yl)-4,4a,8,8a-tetrahydropyrano[3,2-*d*][1,3,2]dioxasiline (<u>8d</u>)

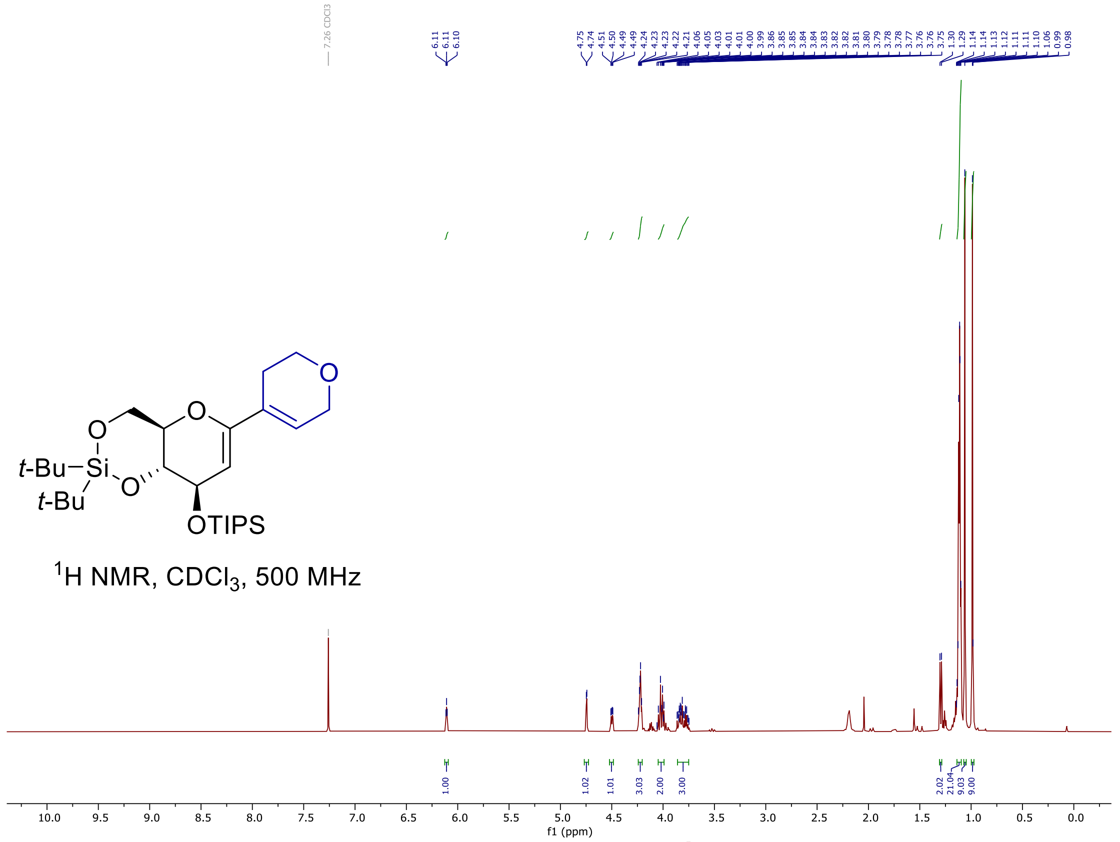

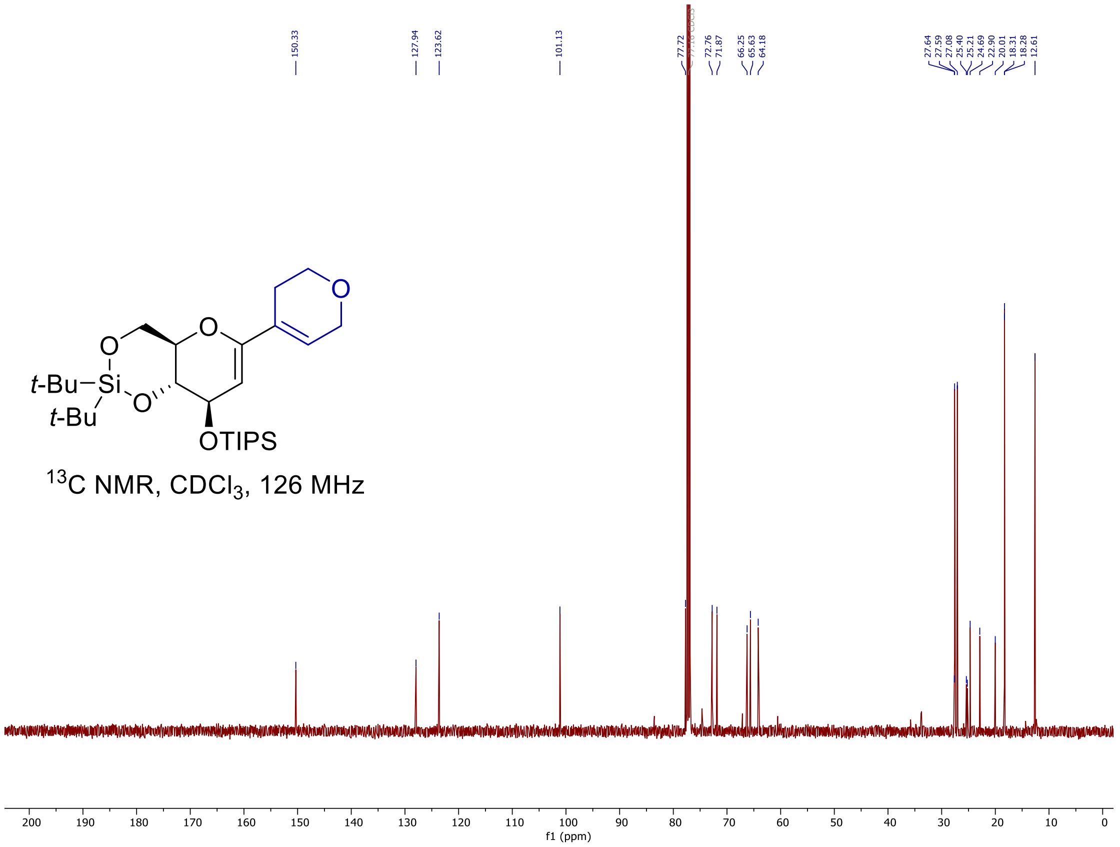

(4a*R*,8*R*,8a*R*)-8-((l1-Silyl)oxy)-2,2-di-*tert*-butyl-6-(1,4-dioxaspiro[4.5]dec-7-en-8-yl)-4,4a,8,8a-tetrahydropyrano[3,2-*d*][1,3,2]dioxasiline (8e)

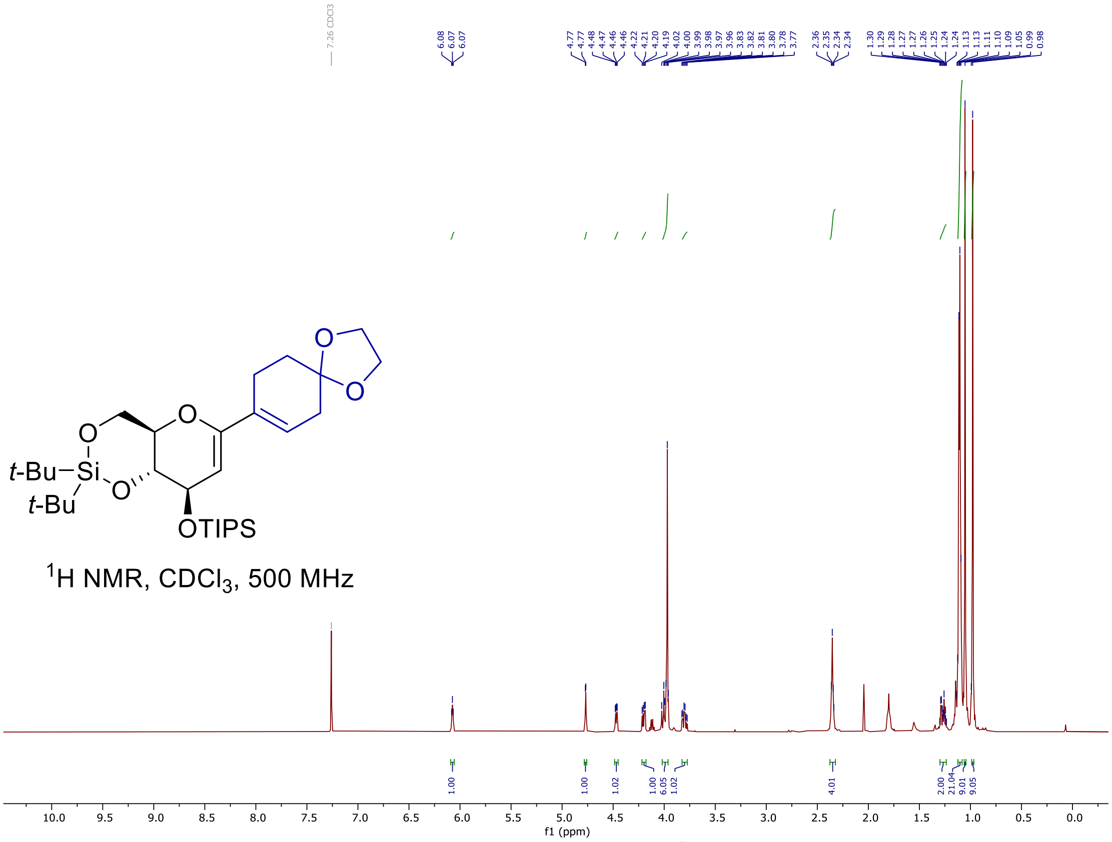

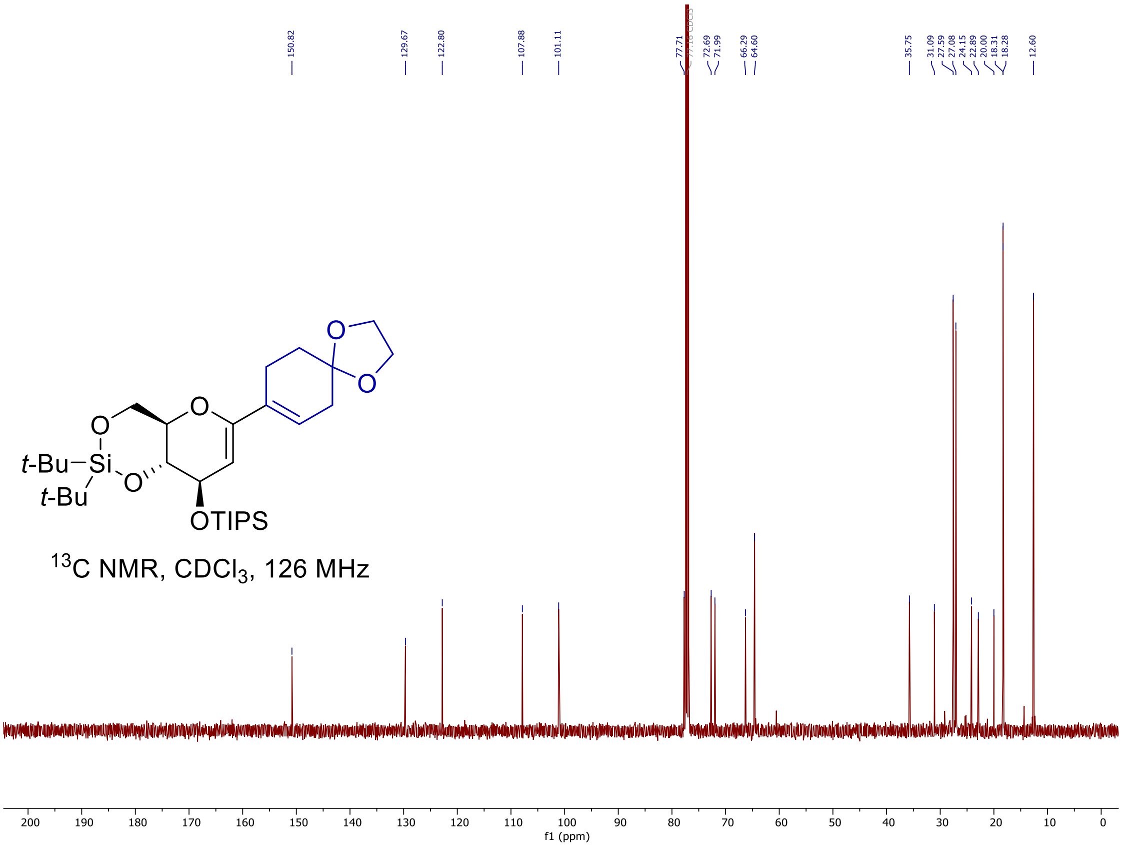

(4a*R*,8*R*,8a*R*)-2,2-di-*tert*-butyl-6-(1,4-dioxaspiro[4.5]dec-7-en-8-yl)-8-((triisopropylsilyl)oxy)-4,4a,8,8a-tetrahydropyrano[3,2-*d*][1,3,2]dioxasiline (9a)

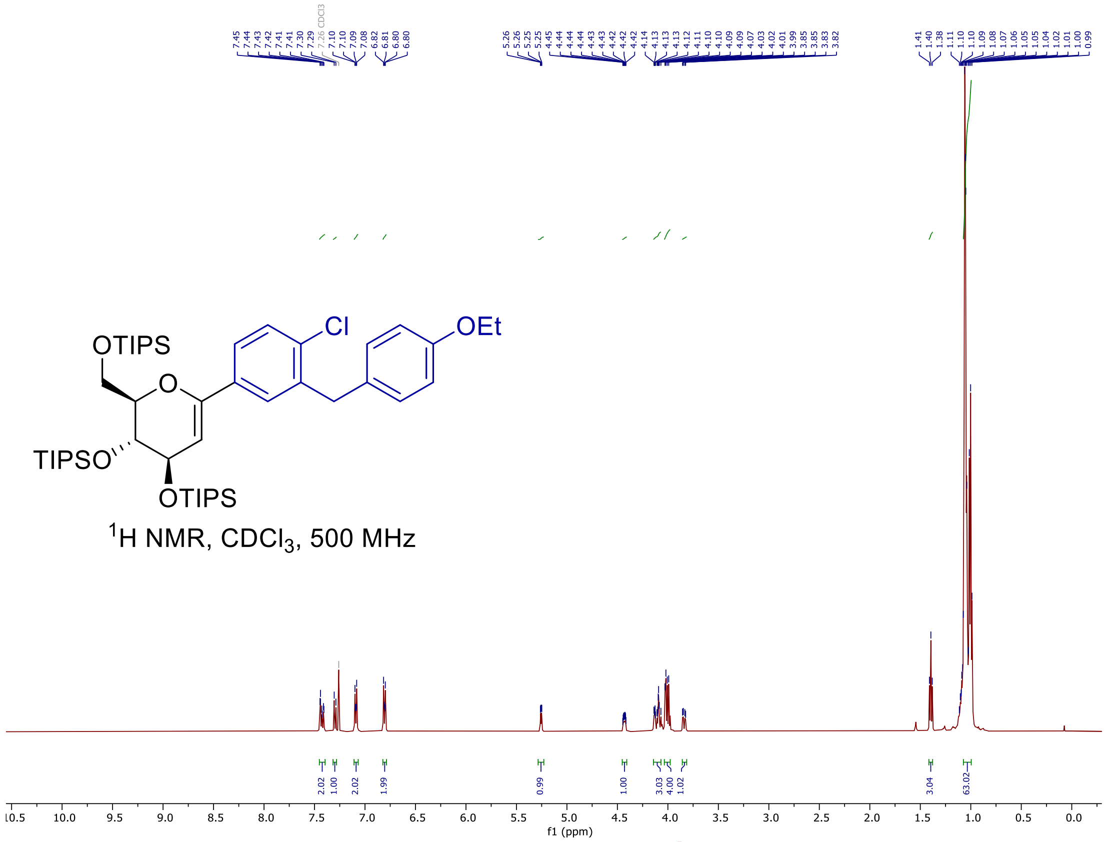

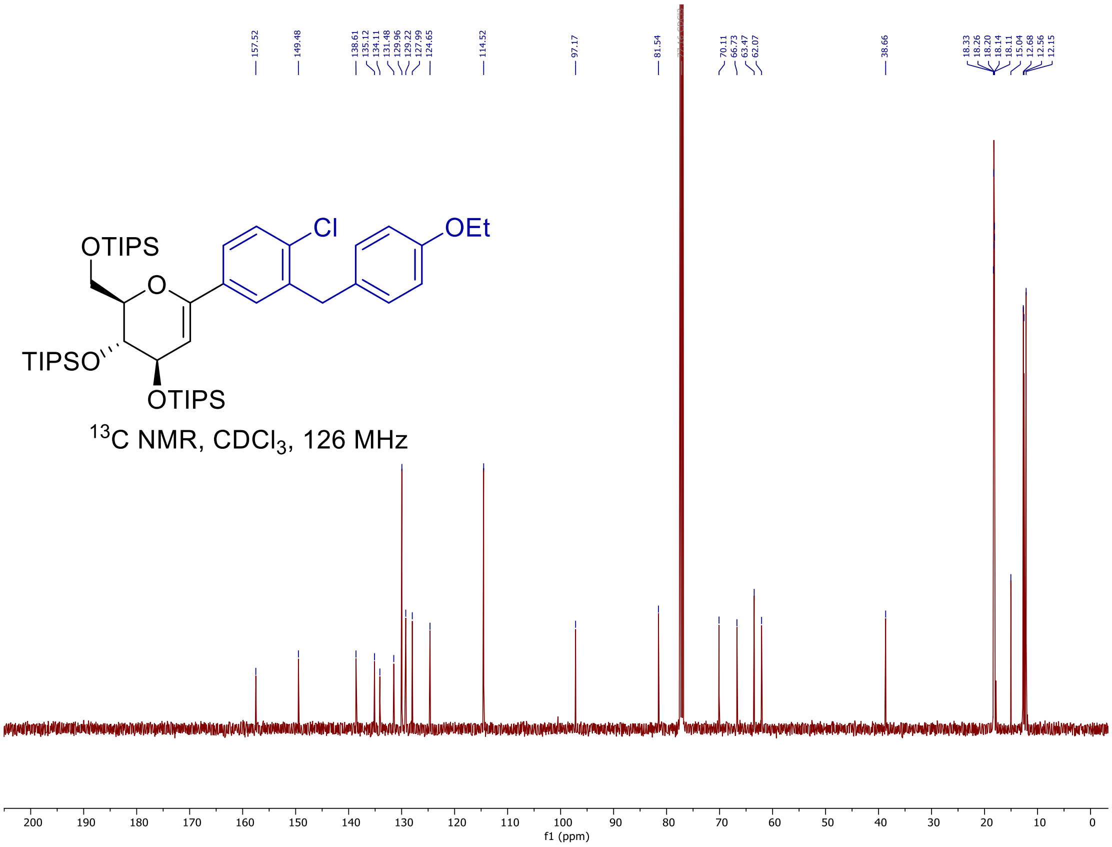

(((2*R*,3*R*,4*R*)-2-(((l1-Silyl)oxy)methyl)-6-(4-chloro-3-(4-(((*S*)-tetrahydrofuran-3-yl)oxy)benzyl)phenyl)-3,4-dihydro-2*H*-pyran-3,4-diyl)bis(oxy))bis(l1-silane) (9b)

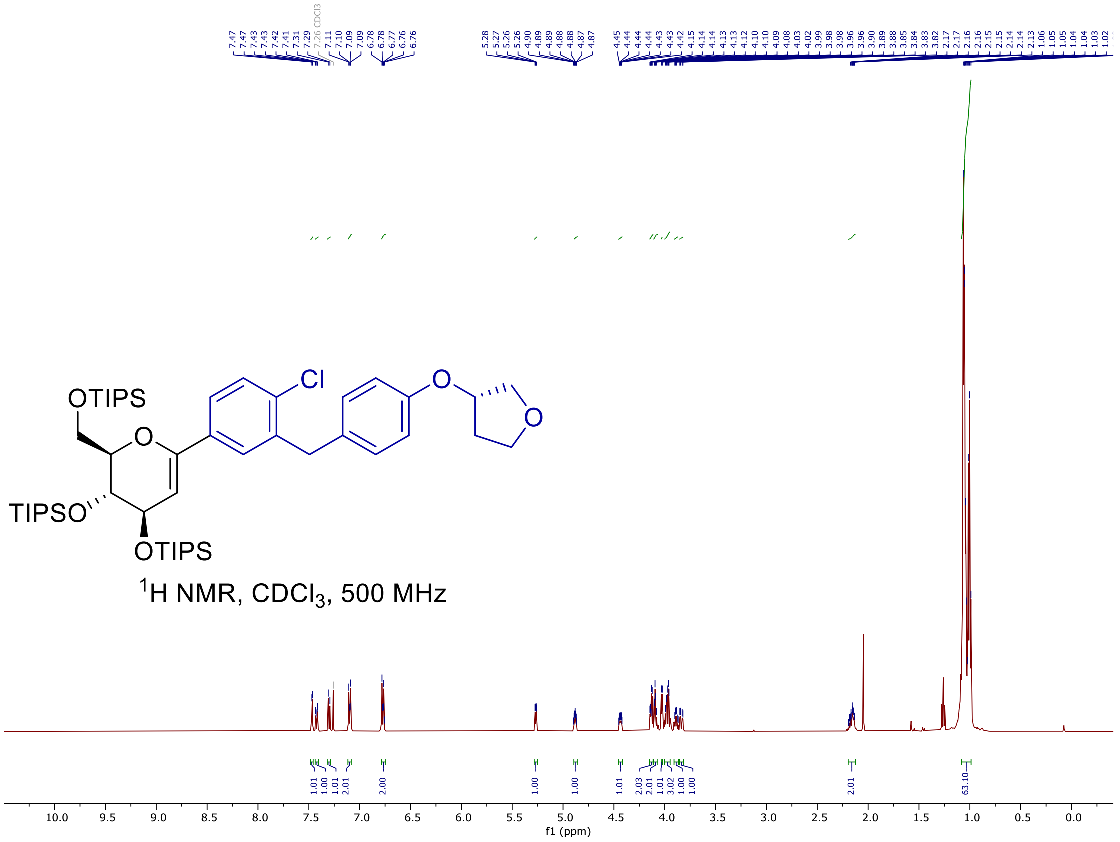

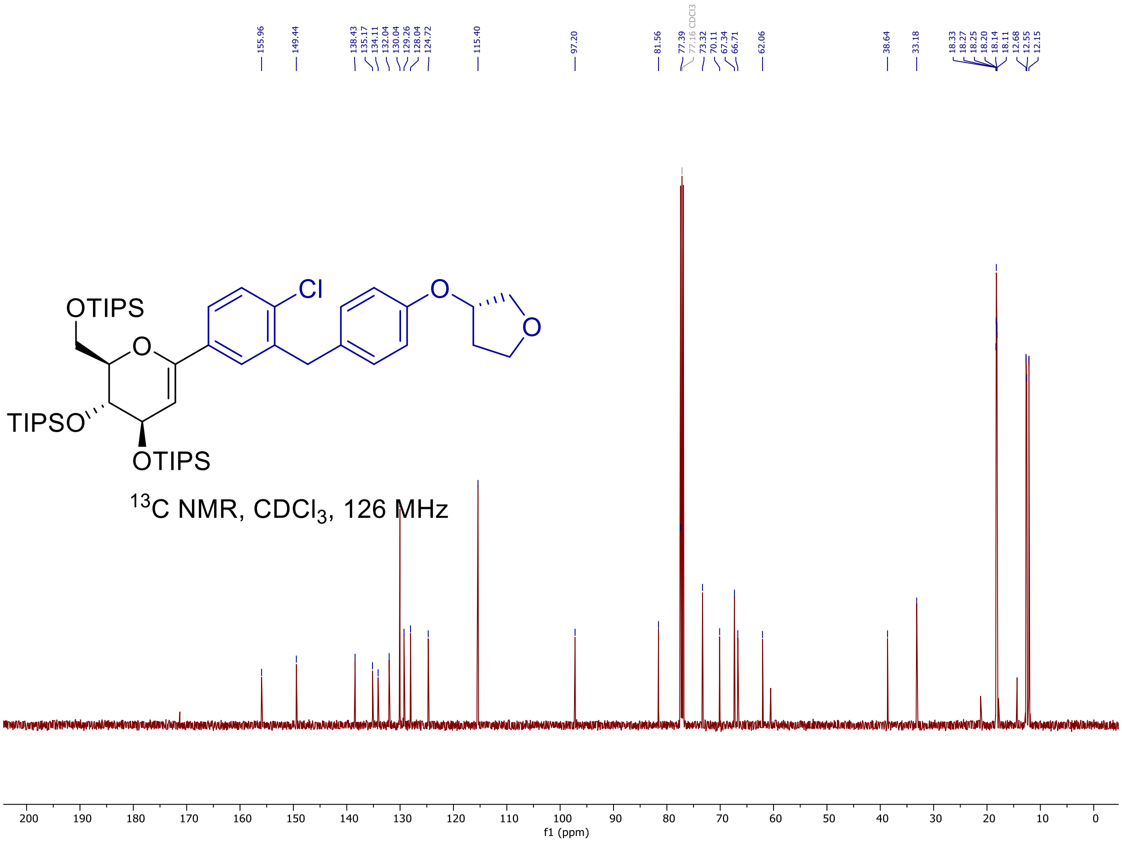

1-Ethyl-5-((2*S*,3*S*,4*S*)-2-methyl-3,4-bis((triisopropylsilyl)oxy)-3,4-dihydro-2*H*-pyran-6-yl)-1*H*-pyrazole (11a)

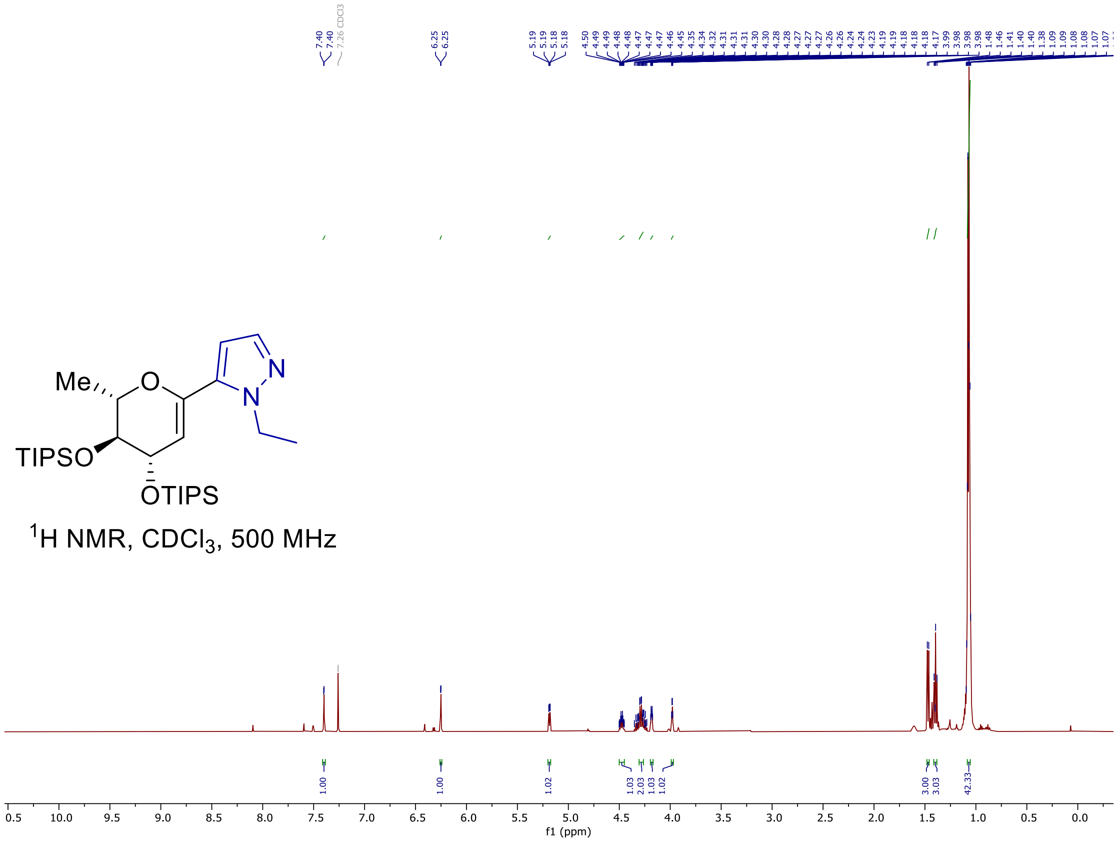

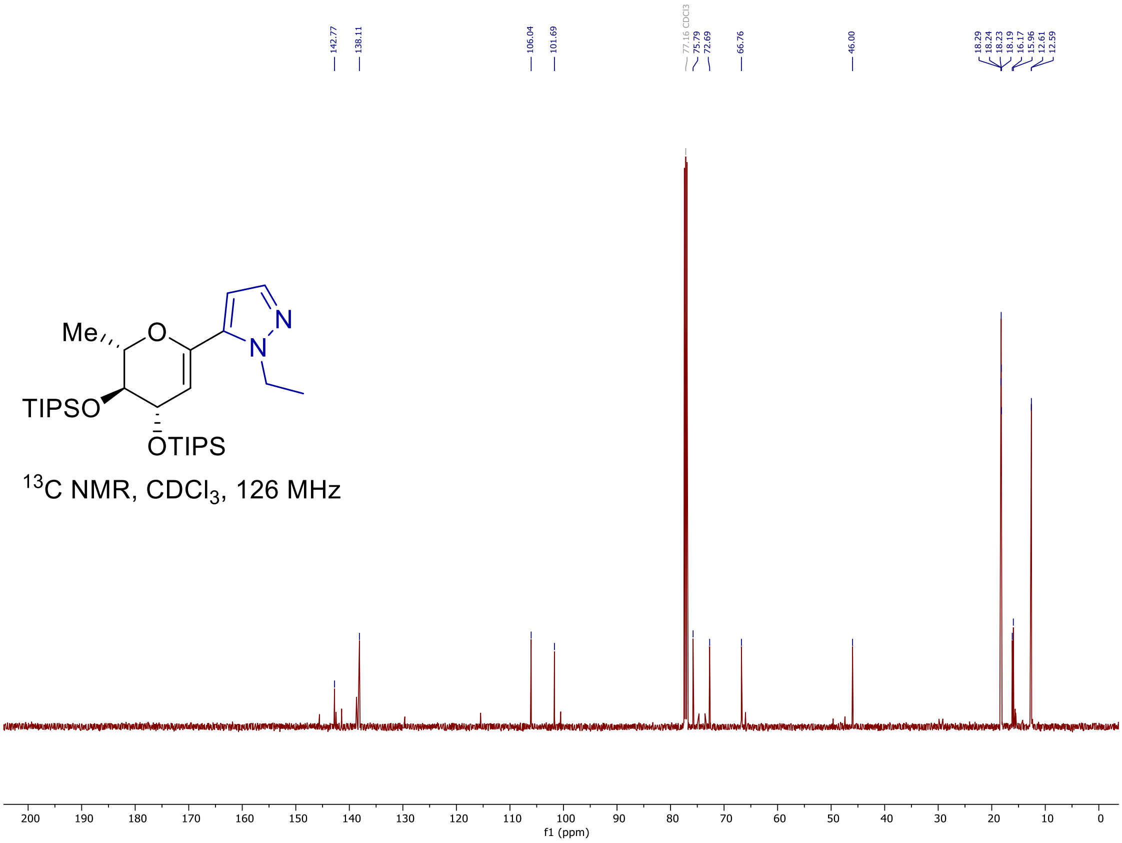

(*E*)-1-(4-((2*S*,3*S*,4*S*)-2-Methyl-3,4-bis((triisopropylsilyl)oxy)-3,4-dihydro-2*H*-pyran-6-yl)phenyl)-2-phenyldiazene (<u>11b</u>)

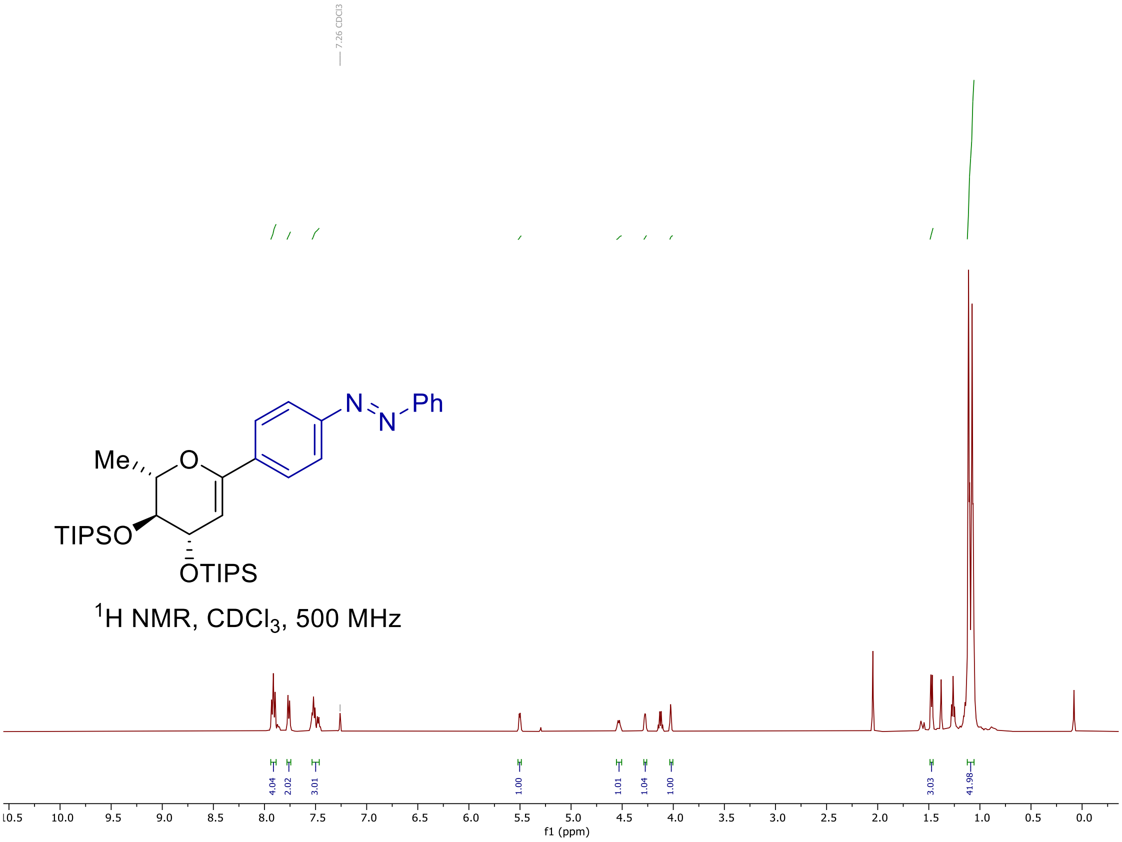

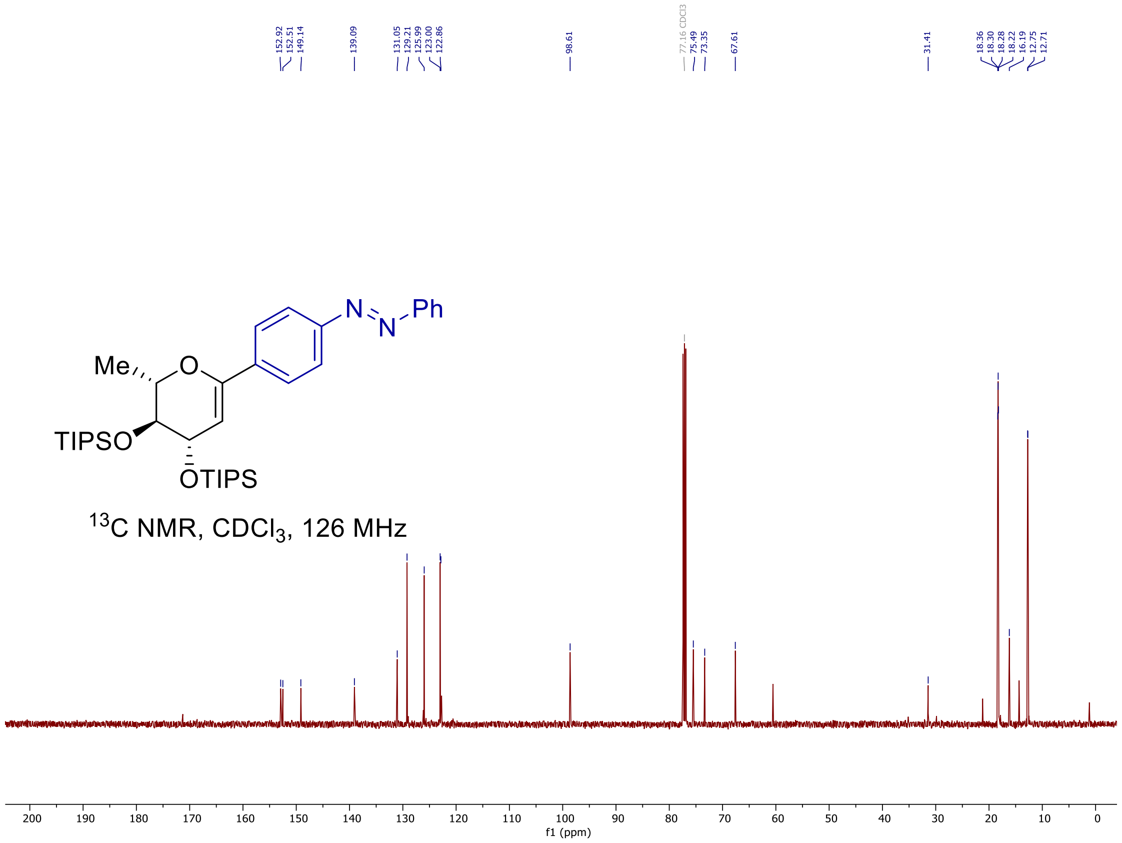

(((2*S*,3*S*,4*S*)-6-(2,3-Difluoro-4-methoxyphenyl)-2-methyl-3,4-dihydro-2*H*-pyran-3,4-diyl)bis(oxy))bis(triisopropylsilane) (11c)

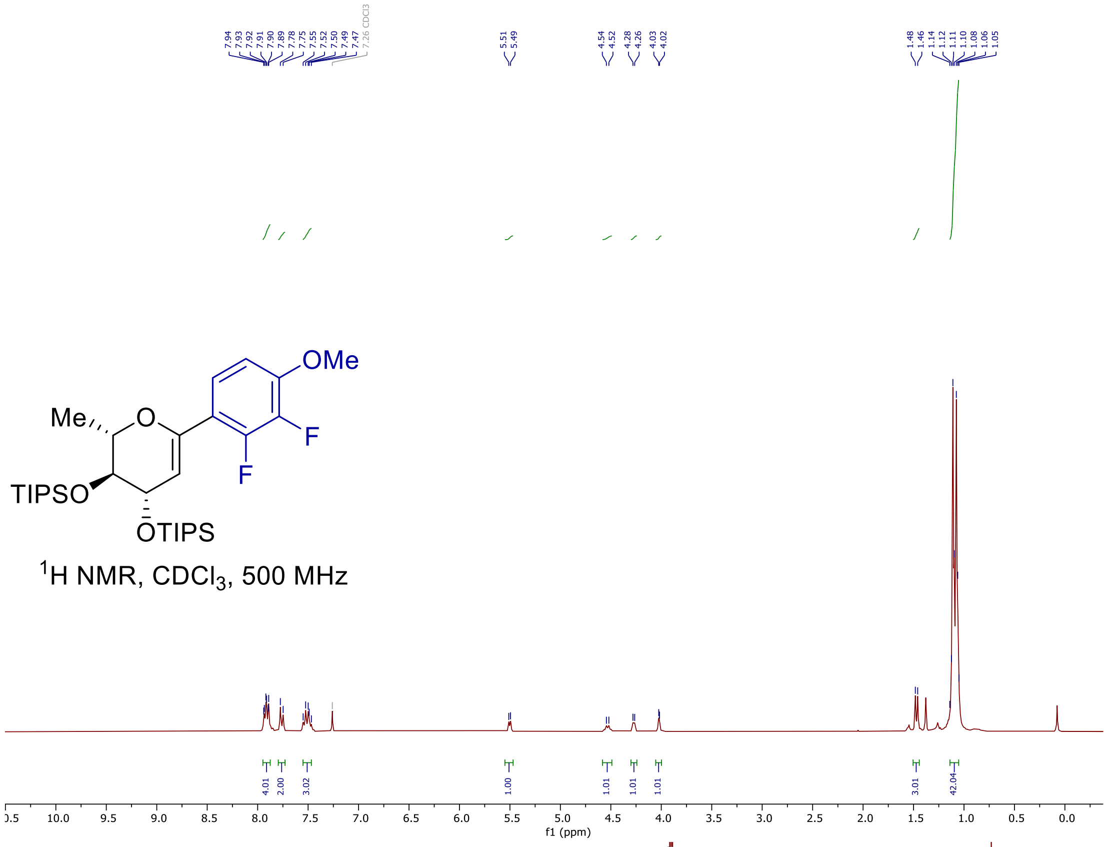

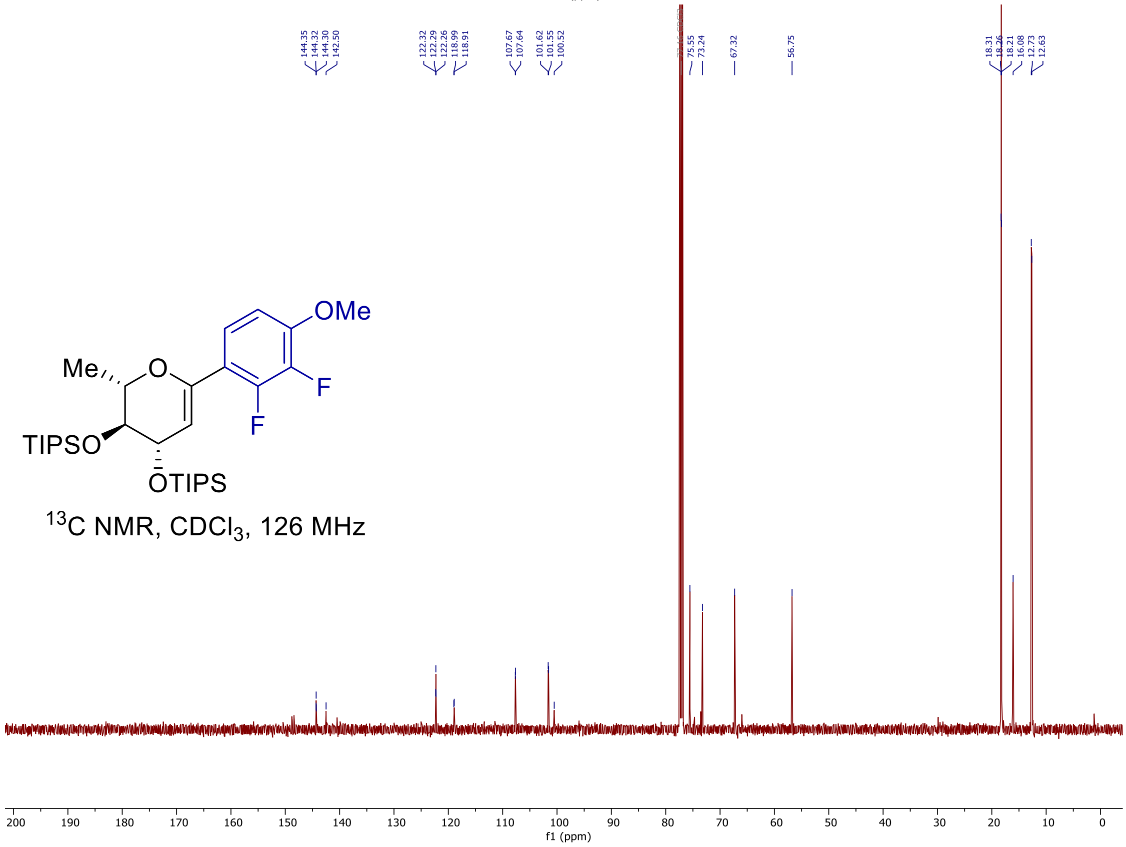

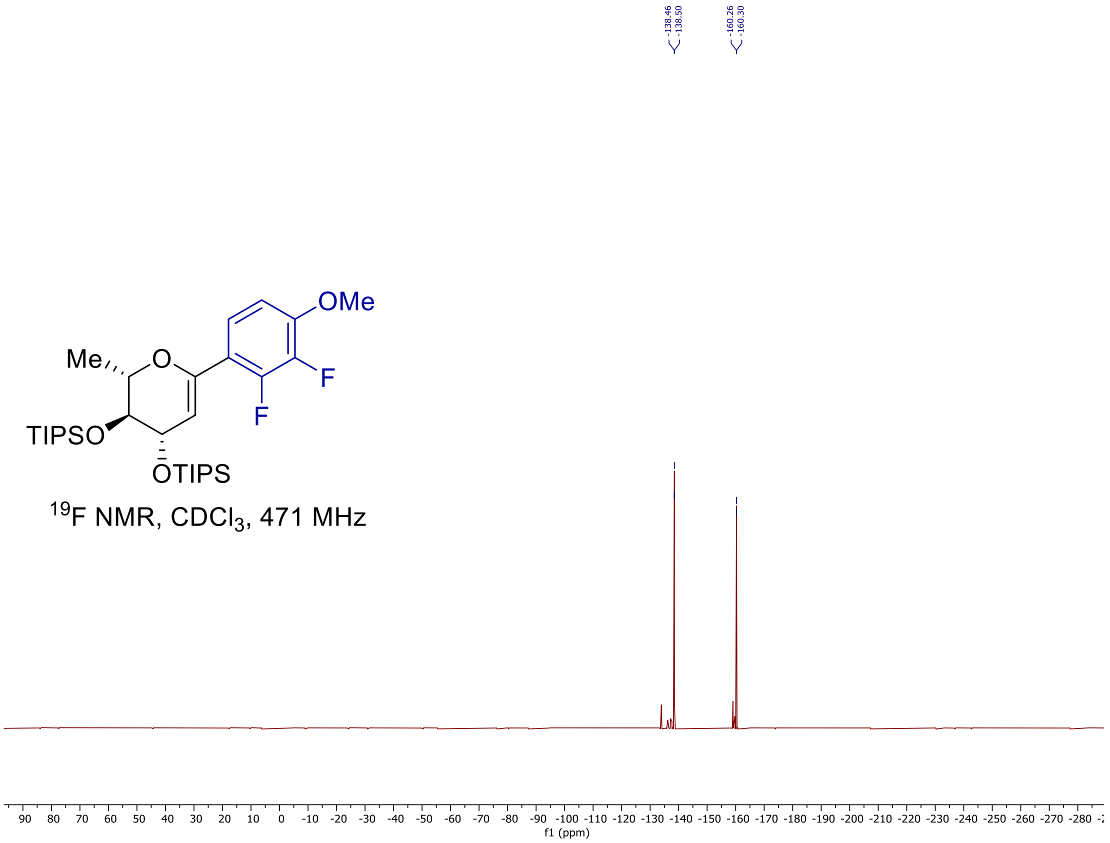

-138.46
-138.50
-160.26
-160.30
OMe
Me
O
F
F
TIPSO
OTIPS
19F NMR, CDCl3, 471 MHz
90 80 70 60 50 40 30 20 10 0 -10 -20 -30 -40 -50 -60 -70 -80 -90 -100 -110 -120 -130 -140 -150 -160 -170 -180 -190 -200 -210 -220 -230 -240 -250 -260 -270 -280
f1 (ppm)

(((2*S*,3*S*,4*S*)-6-(4-chloro-3-(4-(((*S*)-tetrahydrofuran-3-yl)oxy)benzyl)phenyl)-2-methyl-3,4-dihydro-2*H*-pyran-3,4-diyl)bis(oxy))bis(triisopropylsilane) (11d)

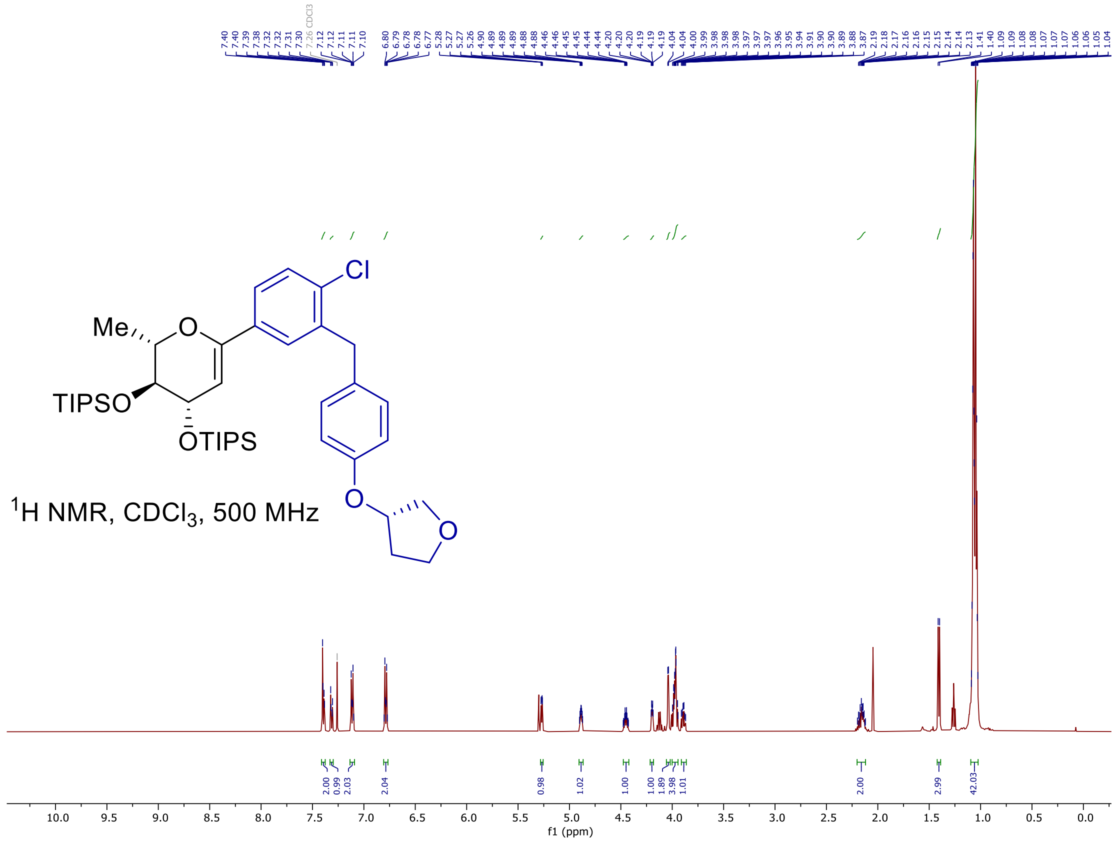

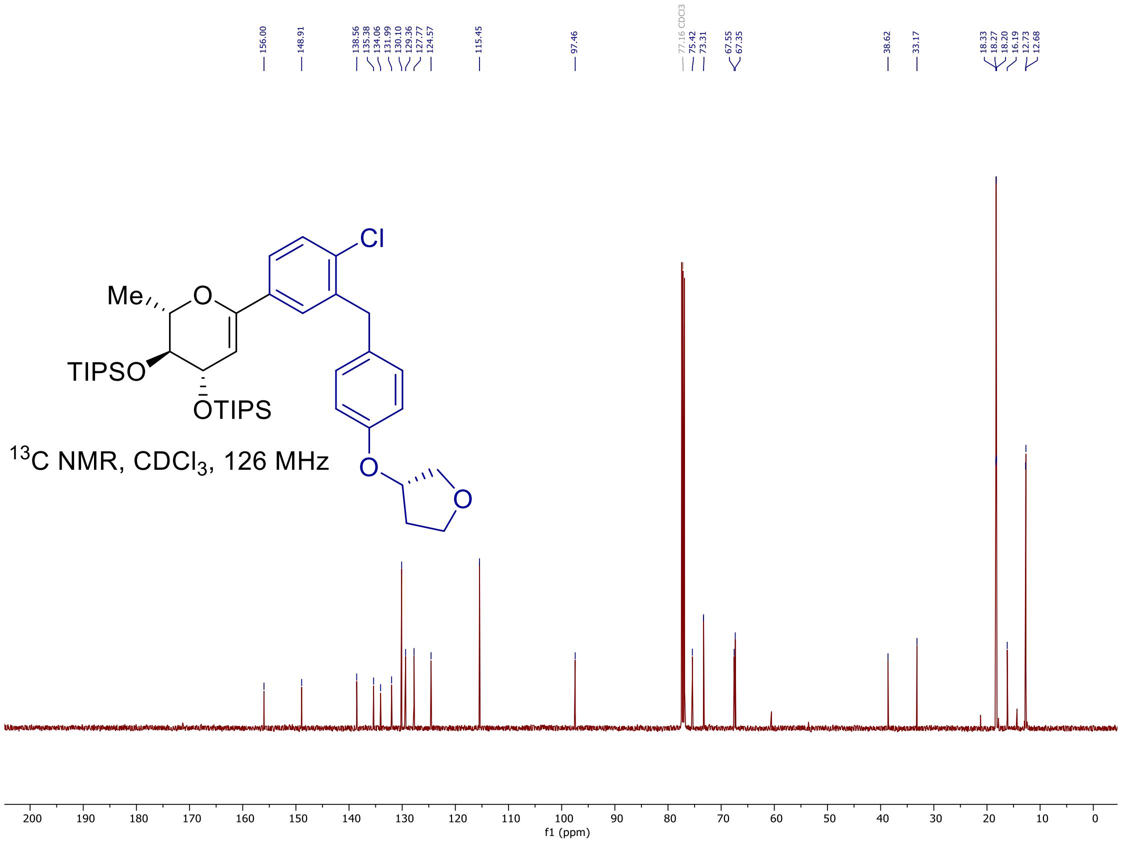

(((3*R*,4*R*)-6-(benzo[*d*][1,3]dioxol-5-yl)-3,4-dihydro-2*H*-pyran-3,4-diyl)bis(oxy))bis(triisopropylsilane) (<u>14a</u>)

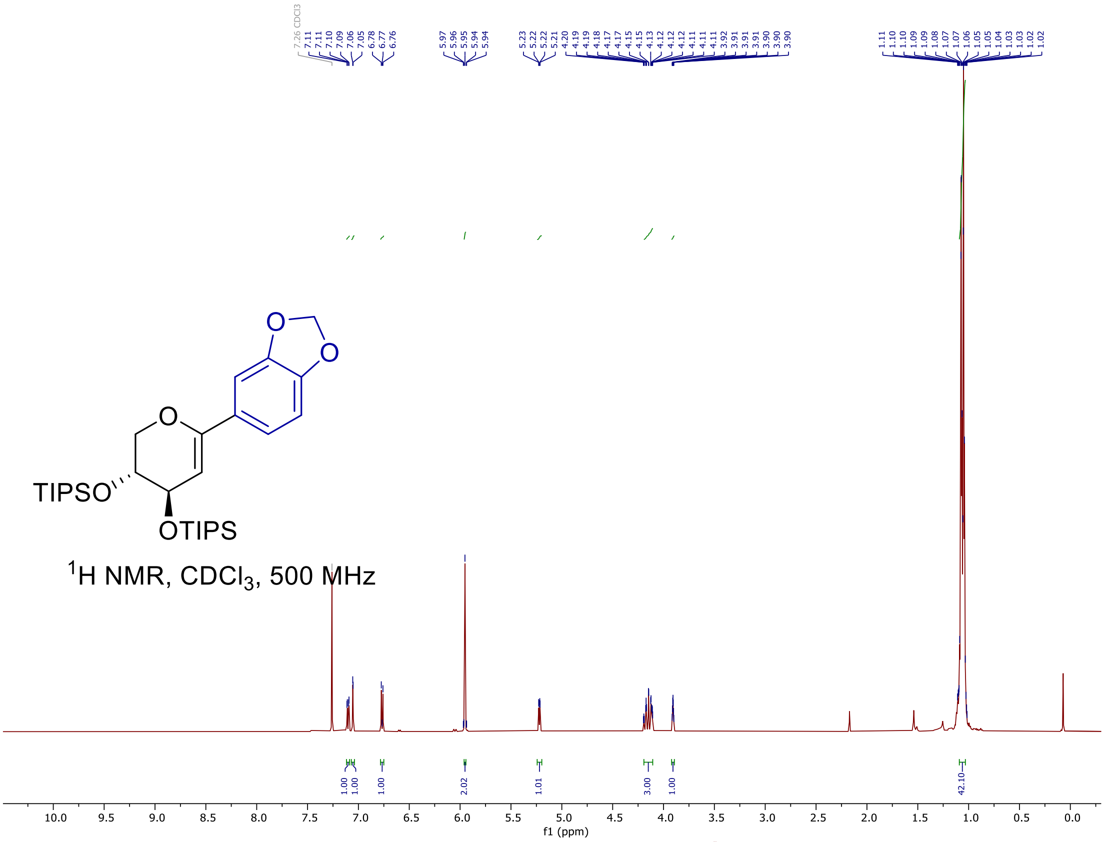

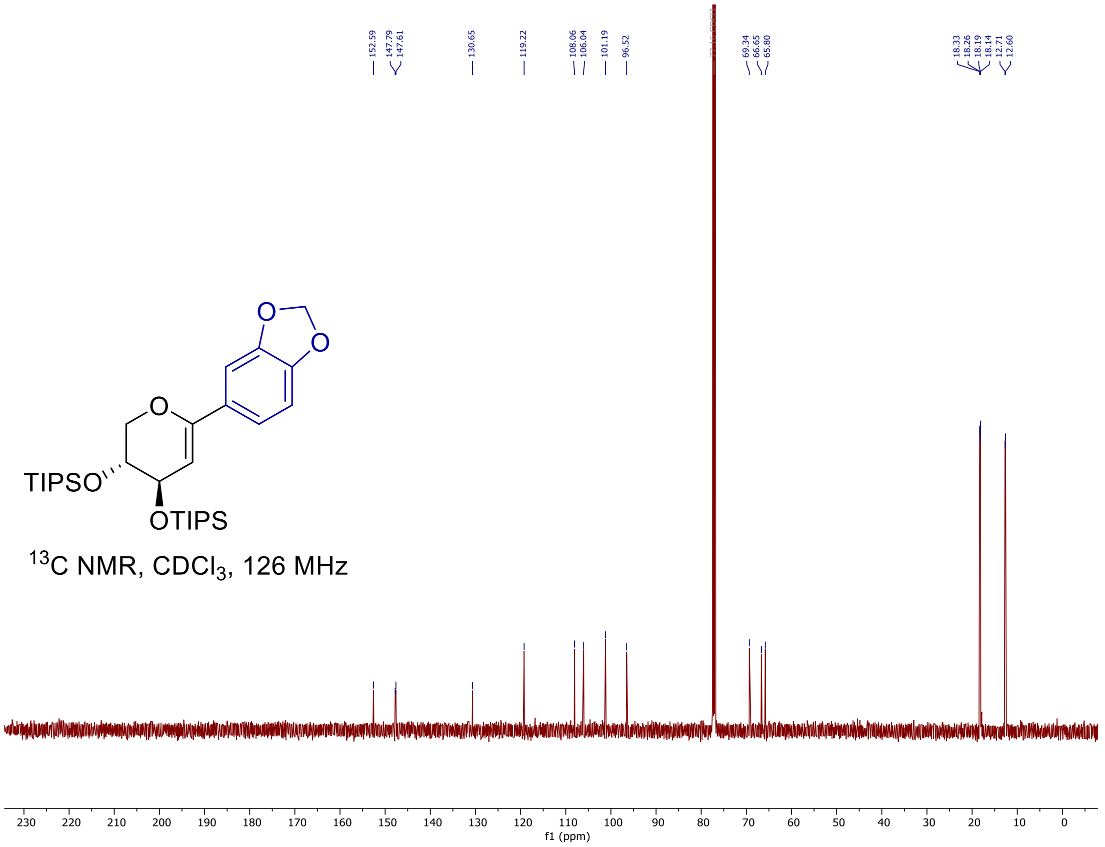

(((3*R*,4*R*)-6-(Furan-3-yl)-3,4-dihydro-2*H*-pyran-3,4-diyl)bis(oxy))bis(triisopropylsilane) (14b)

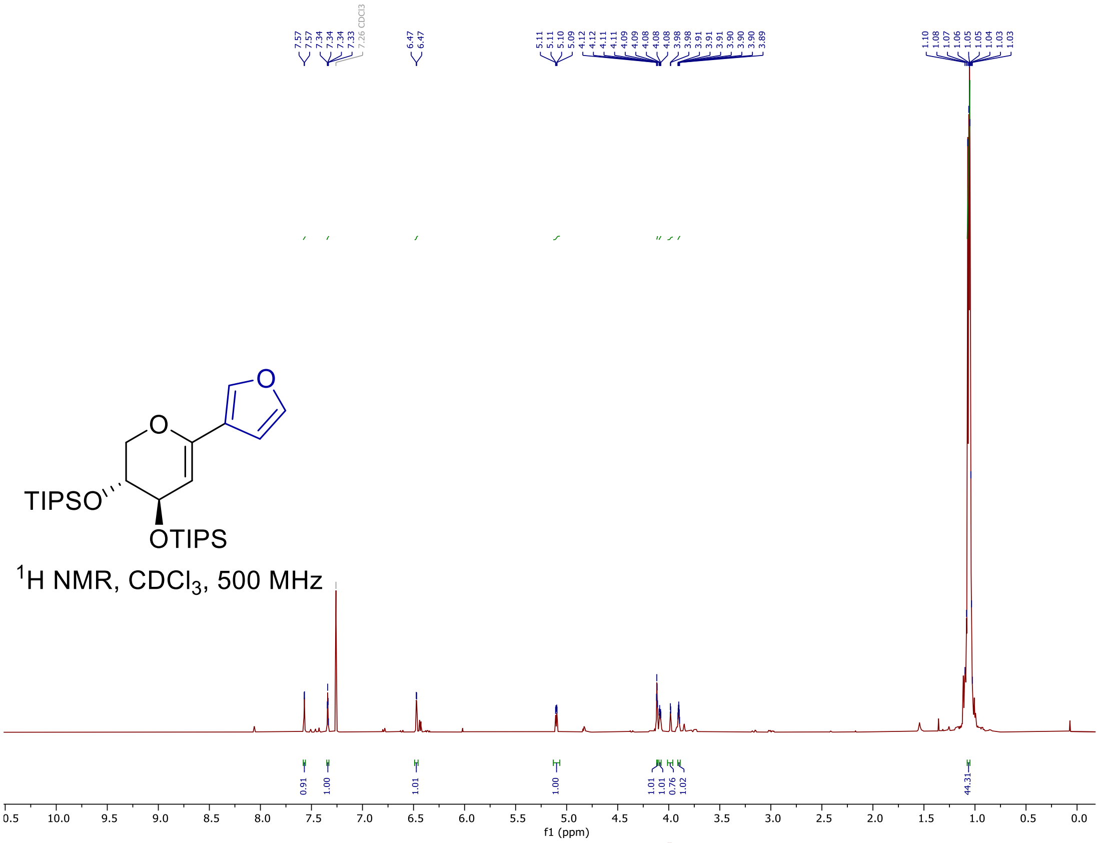

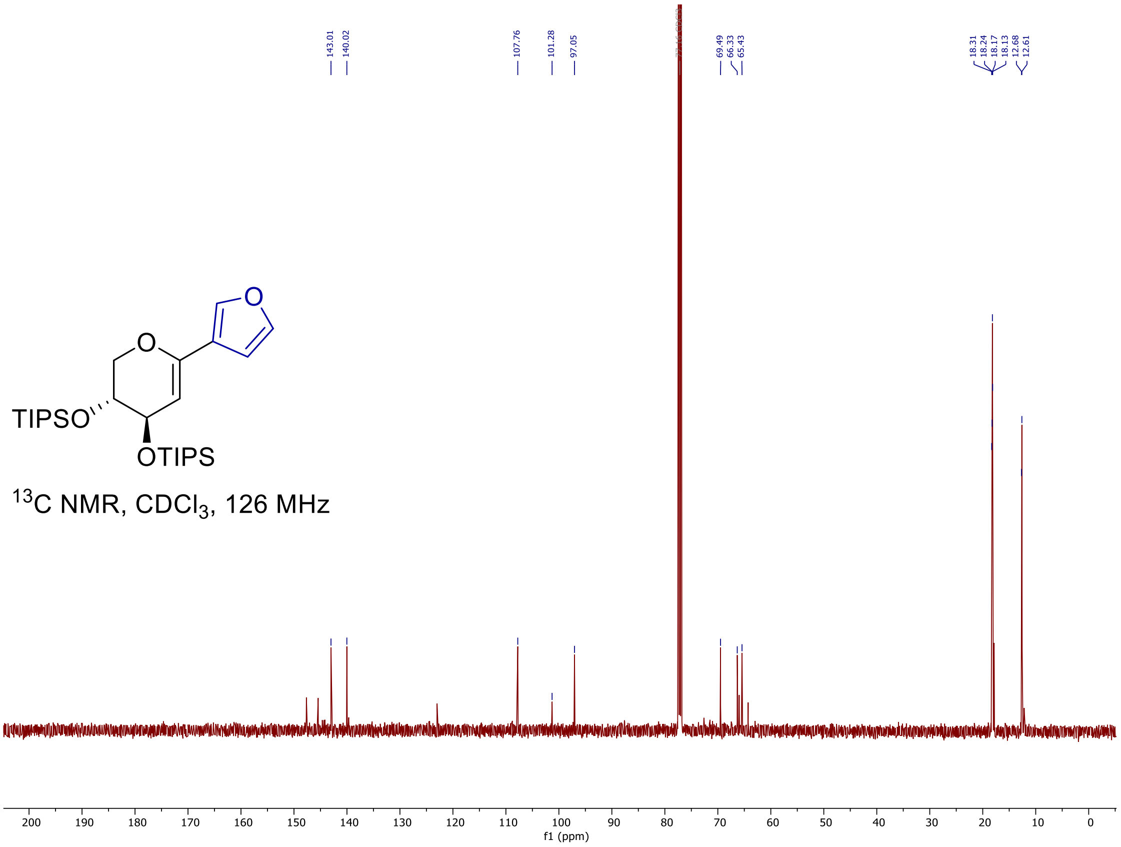

(((3*R*,4*R*)-6-(4-Chloro-3-(4-(((*S*)-tetrahydrofuran-3-yl)oxy)benzyl)phenyl)-3,4-dihydro-2*H*-pyran-3,4-diyl)bis(oxy))bis(triisopropylsilane) (<u>14c</u>)

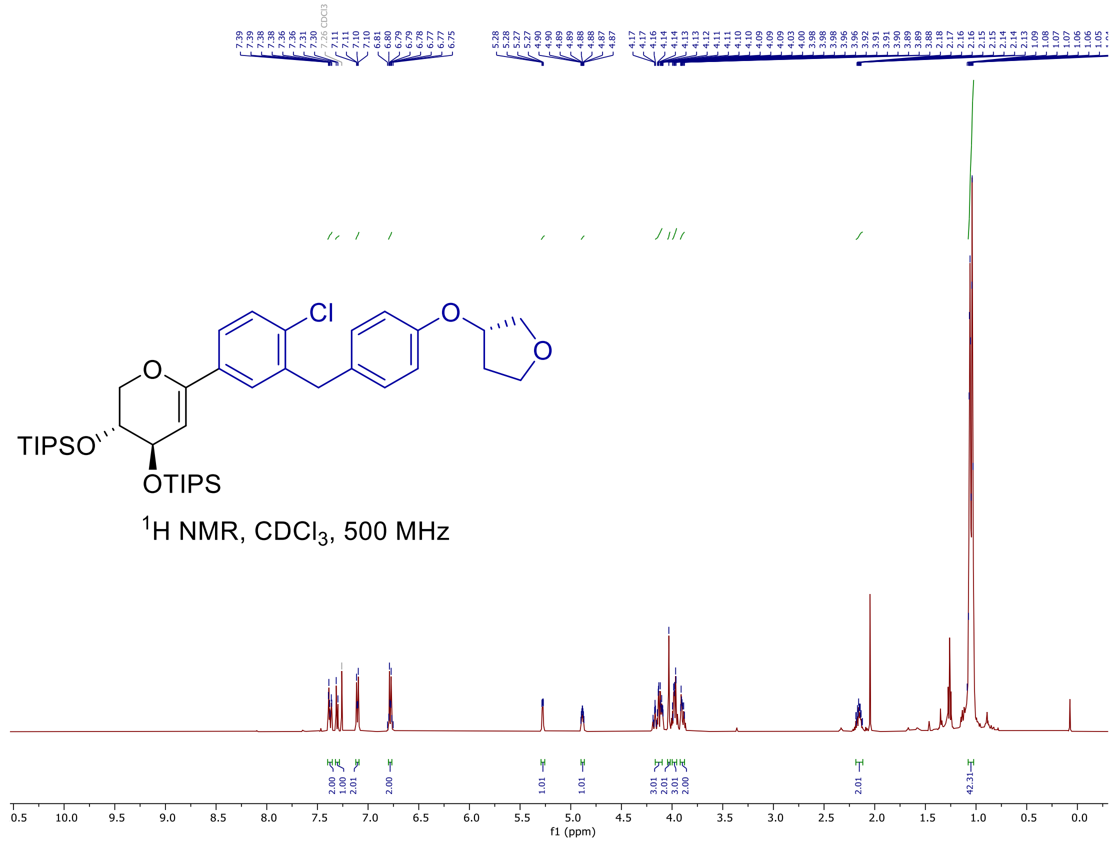

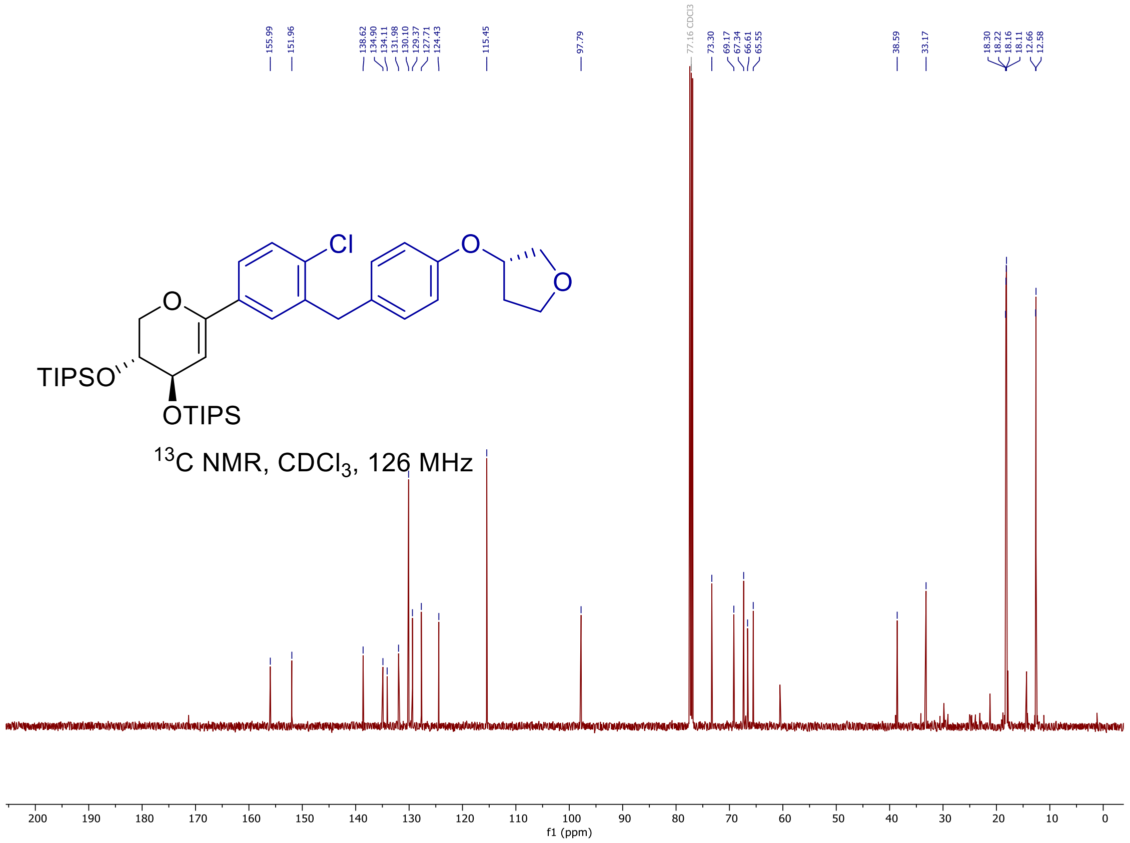

(((3*R*,4*R*)-6-(4-Chloro-3-((5-(4-fluorophenyl)thiophen-2-yl)methyl)phenyl)-3,4-dihydro-2*H*-pyran-3,4-diyl)bis(oxy))bis(triisopropylsilane) (<u>14d</u>)

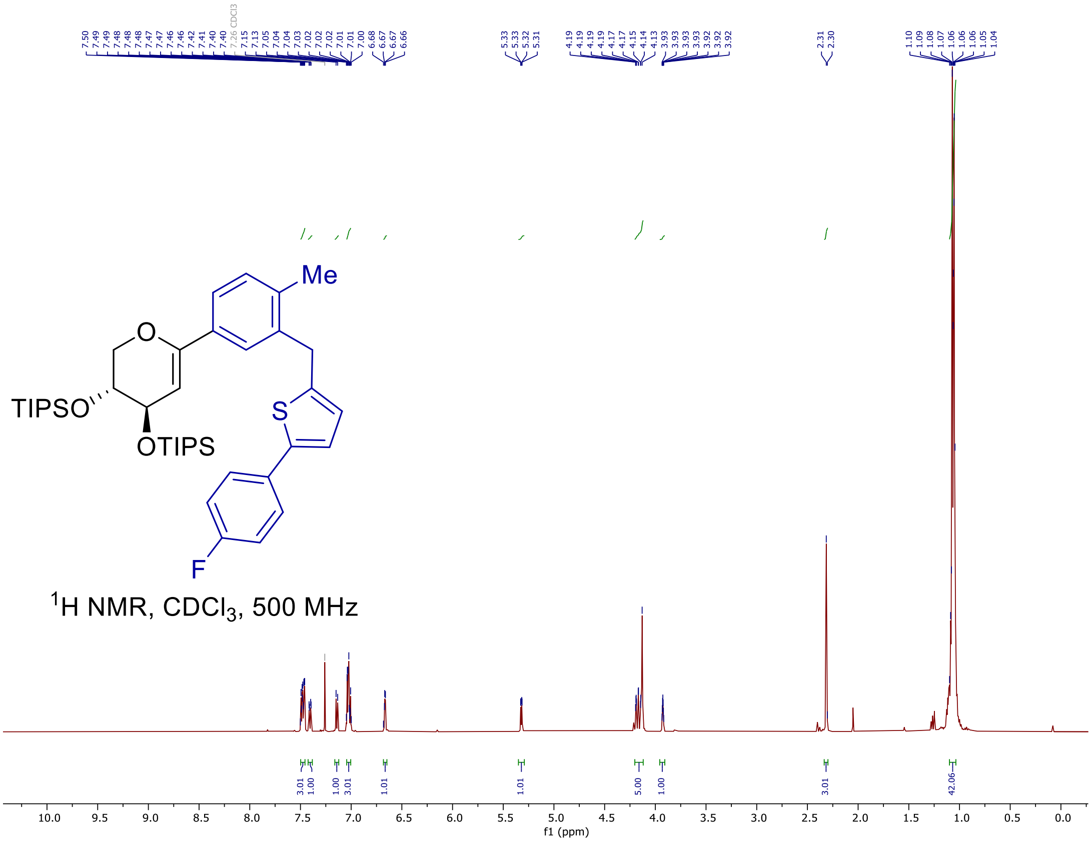

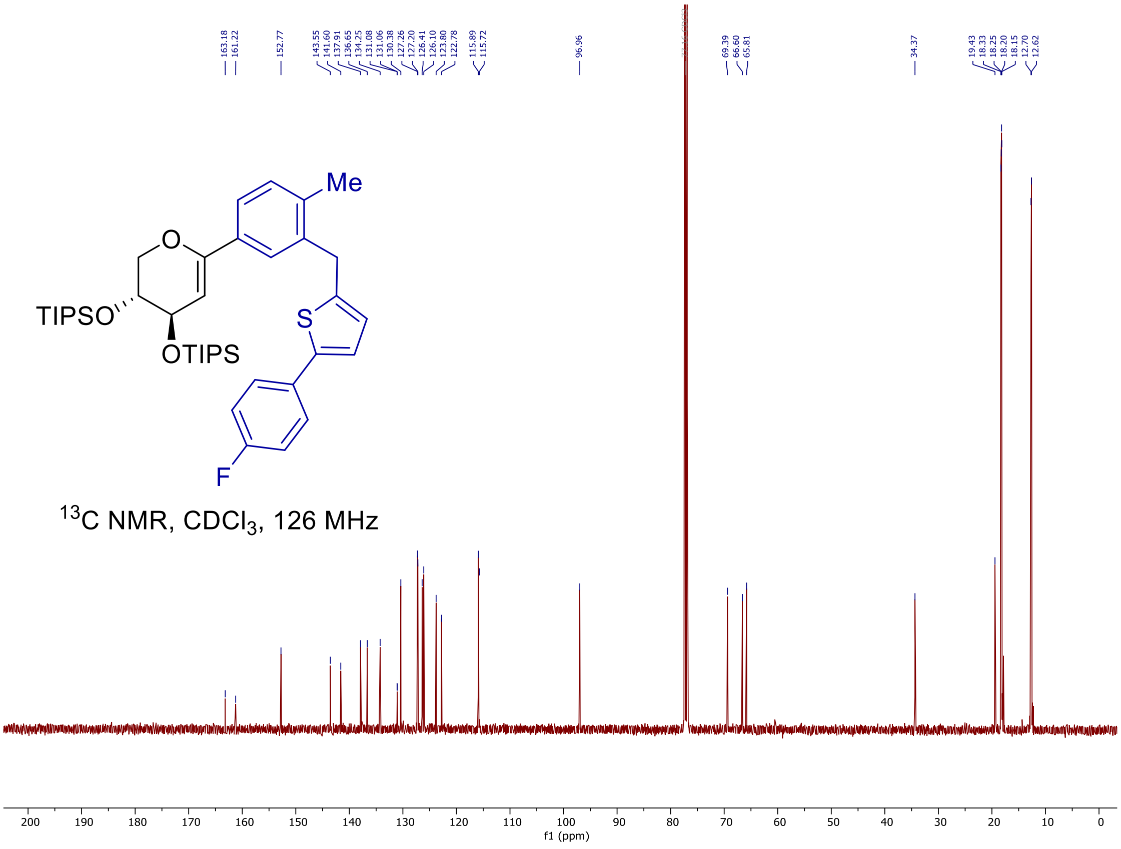

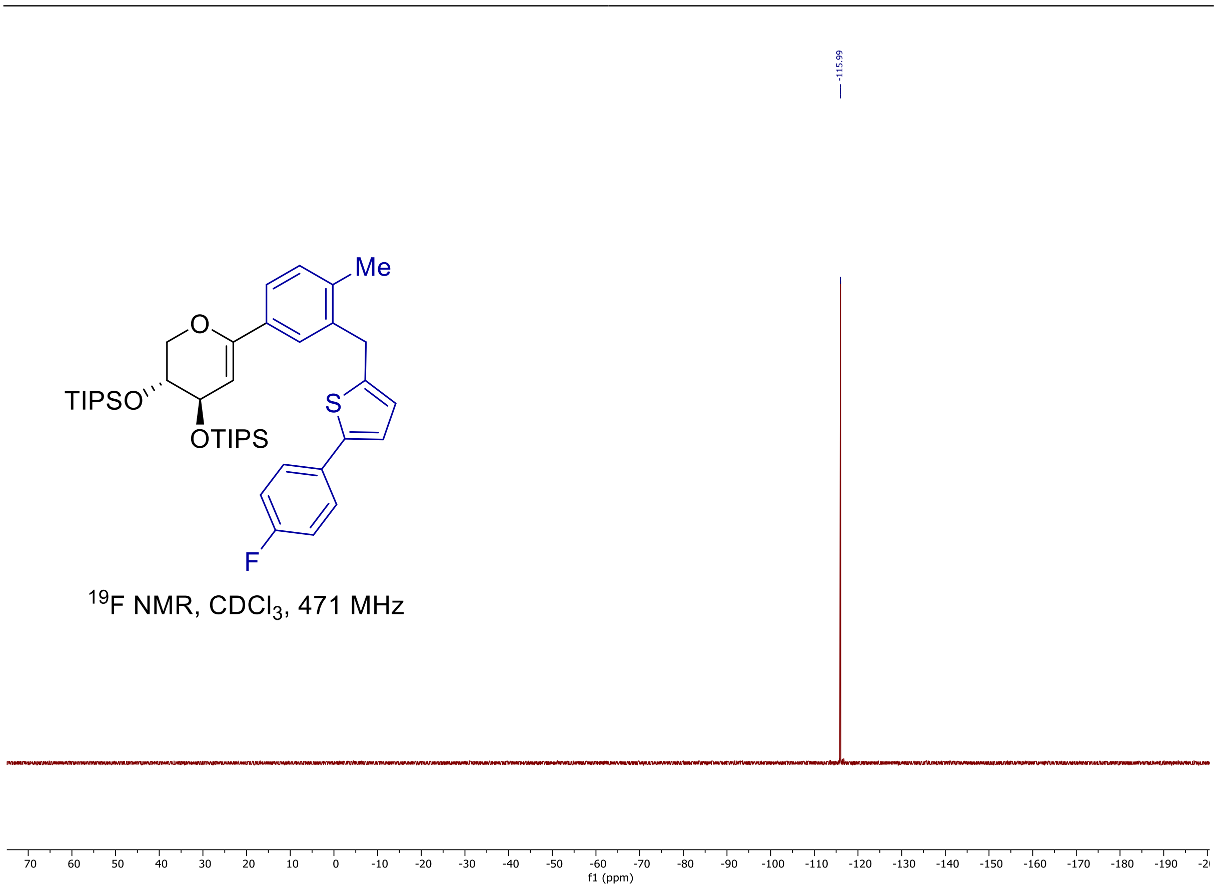

^{19}F NMR, $CDCl_3$, 471 MHz

***tert*-butyl 4-((3*R*,4*R*)-3,4-bis((triisopropylsilyl)oxy)-3,4-dihydro-2*H*-pyran-6-yl)benzoate (<u>14e</u>)**

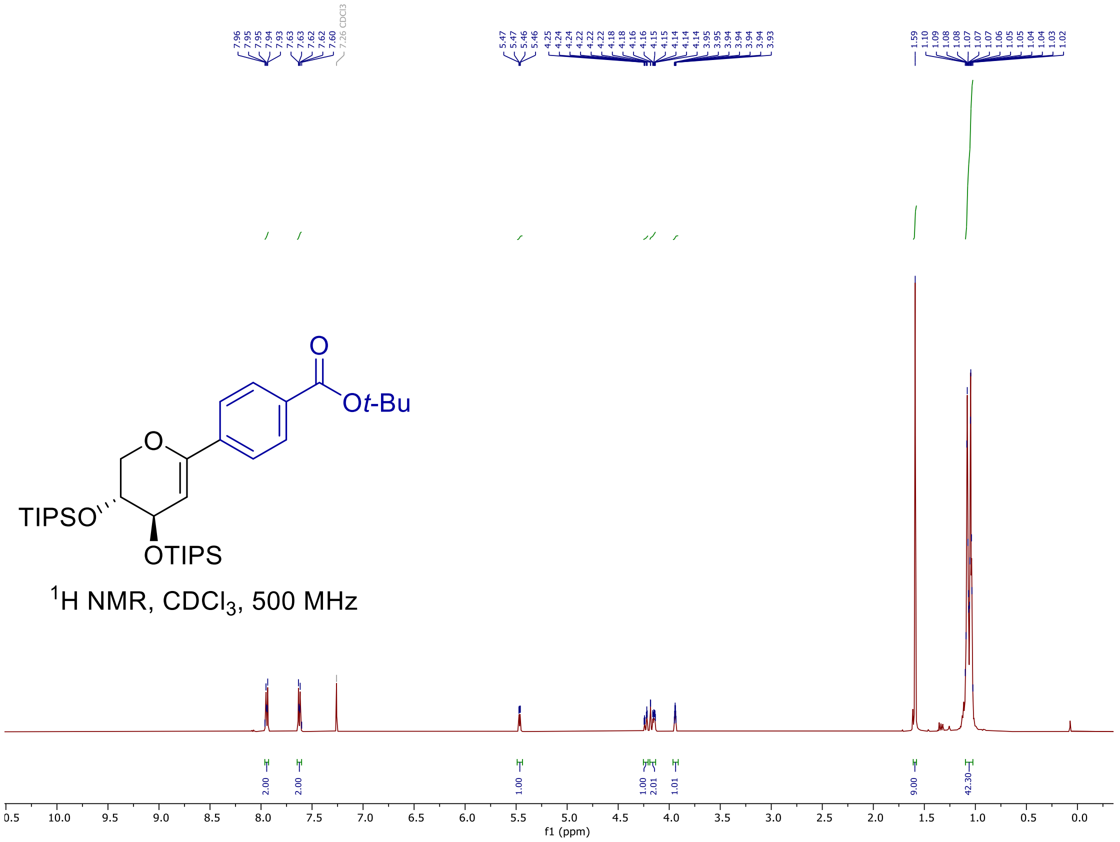

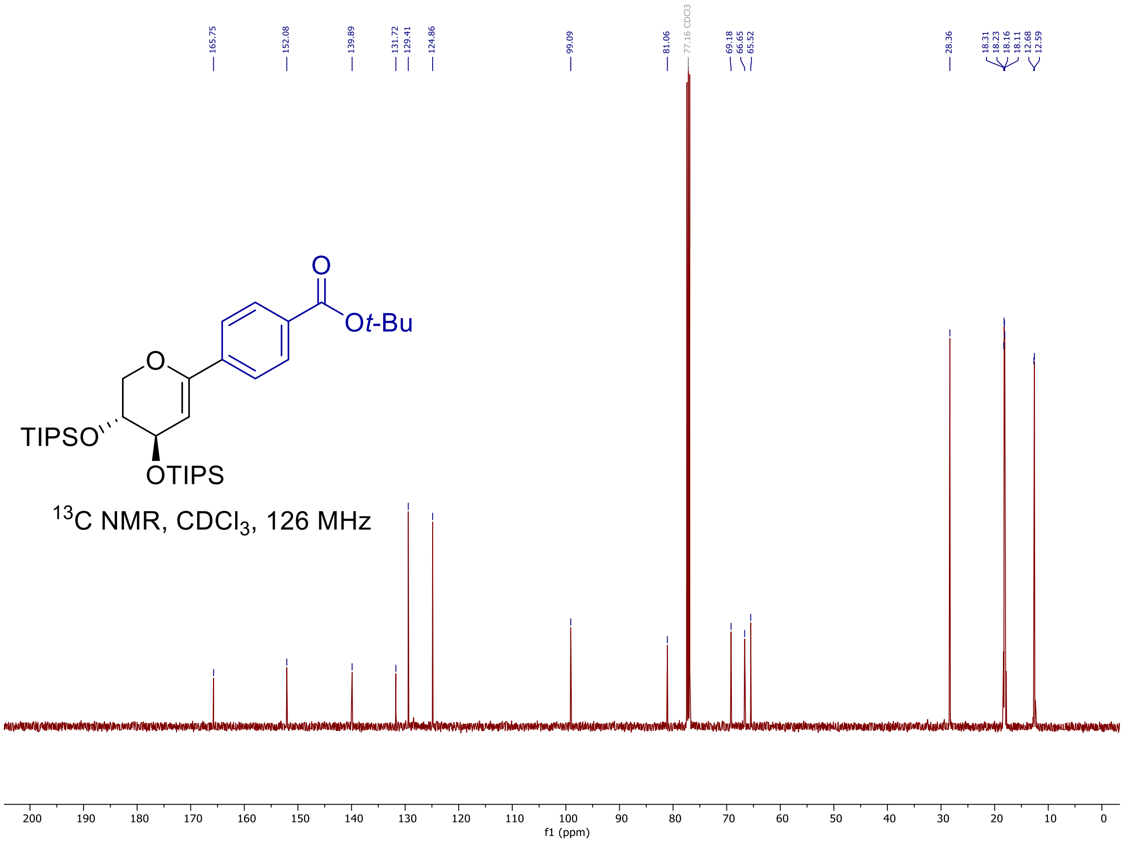

(((3*R*,4*R*)-6-(4-(trifluoromethyl)phenyl)-3,4-dihydro-2*H*-pyran-3,4-diyl)bis(oxy))bis(triisopropylsilane) (<u>14f</u>)

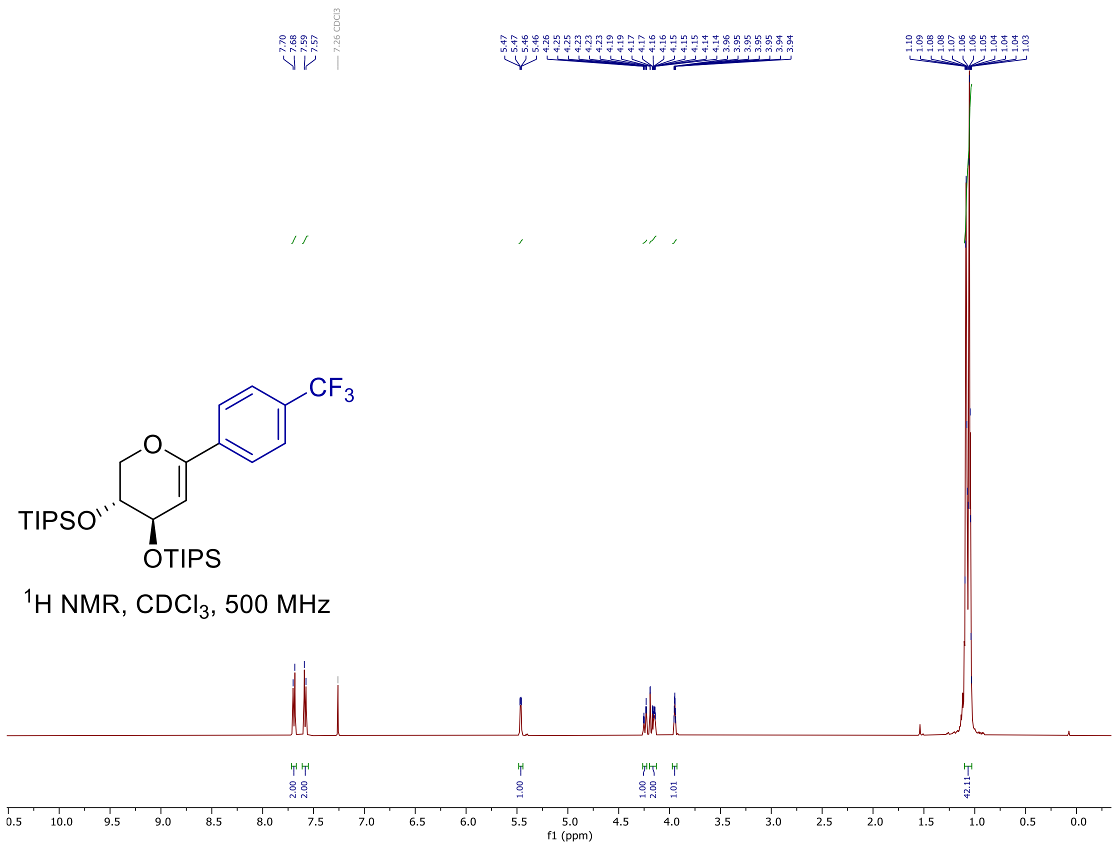

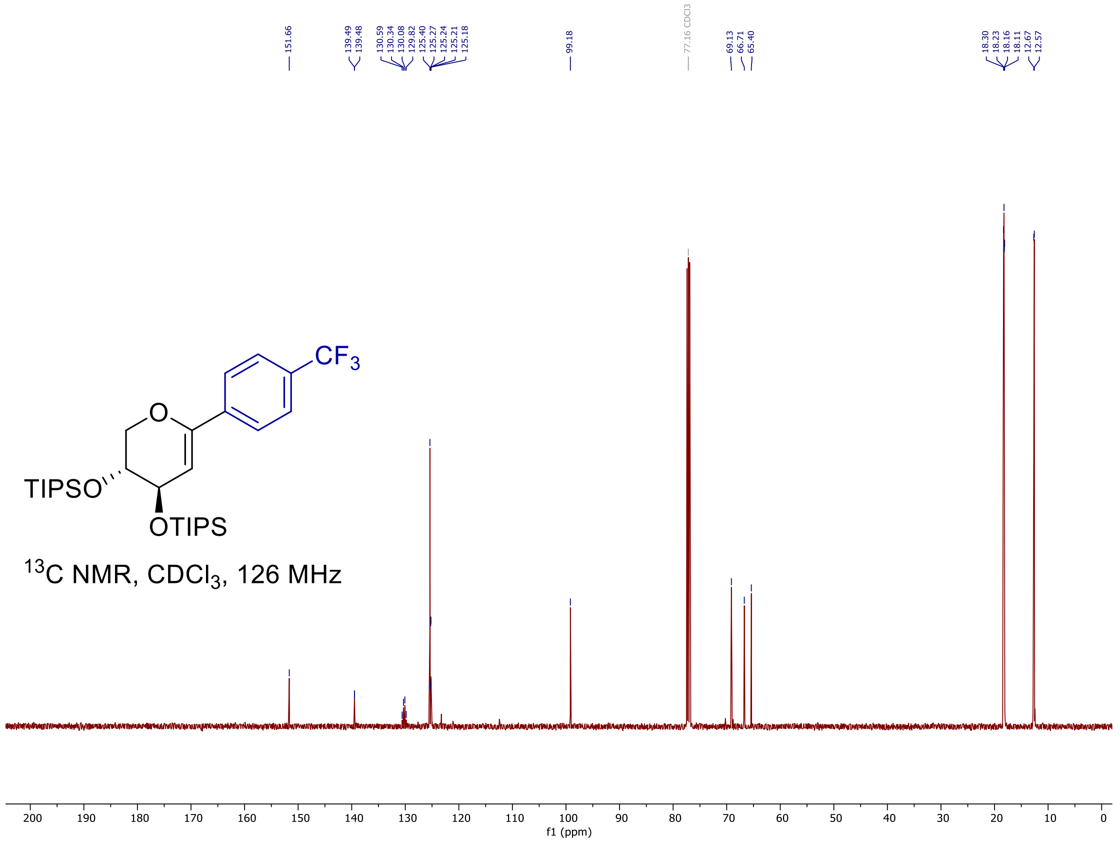

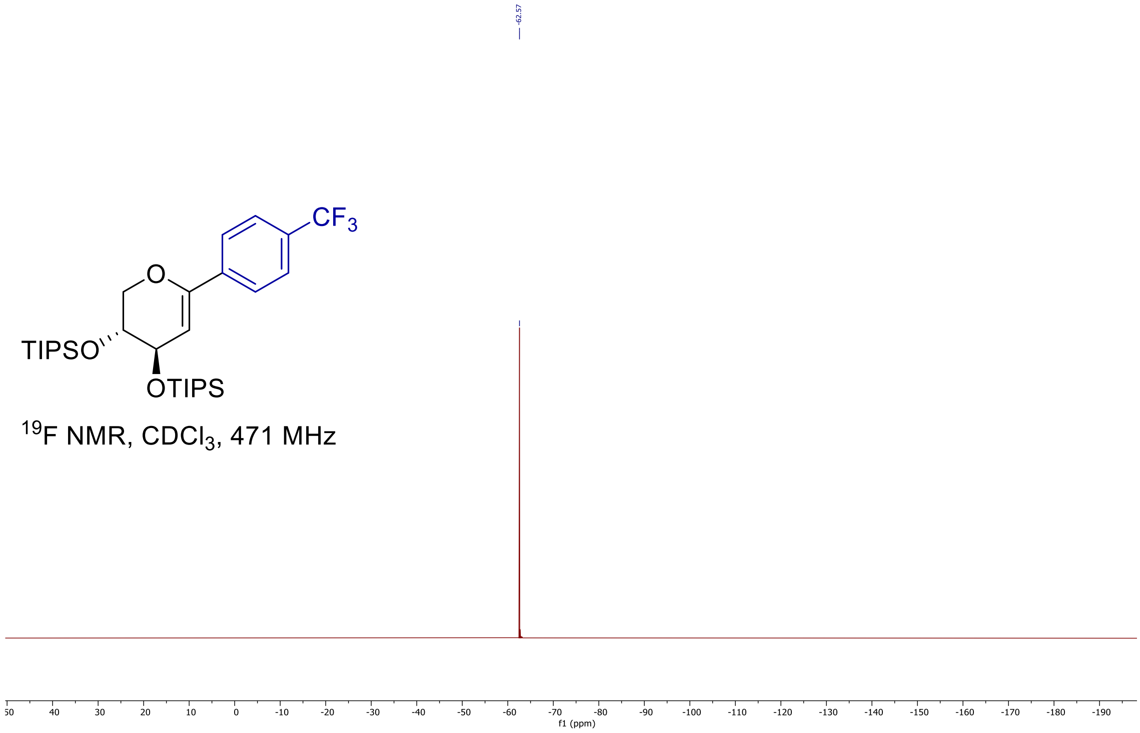
-62.57
CF3
O
TIPSO
OTIPS
19F NMR, CDCl3, 471 MHz
f1 (ppm)

(((3*R*,4*R*)-6-(2-(1,3-Dioxolan-2-yl)ethyl)-3,4-dihydro-2H-pyran-3,4-diyl)bis(oxy))bis(triisopropylsilane) (14g)

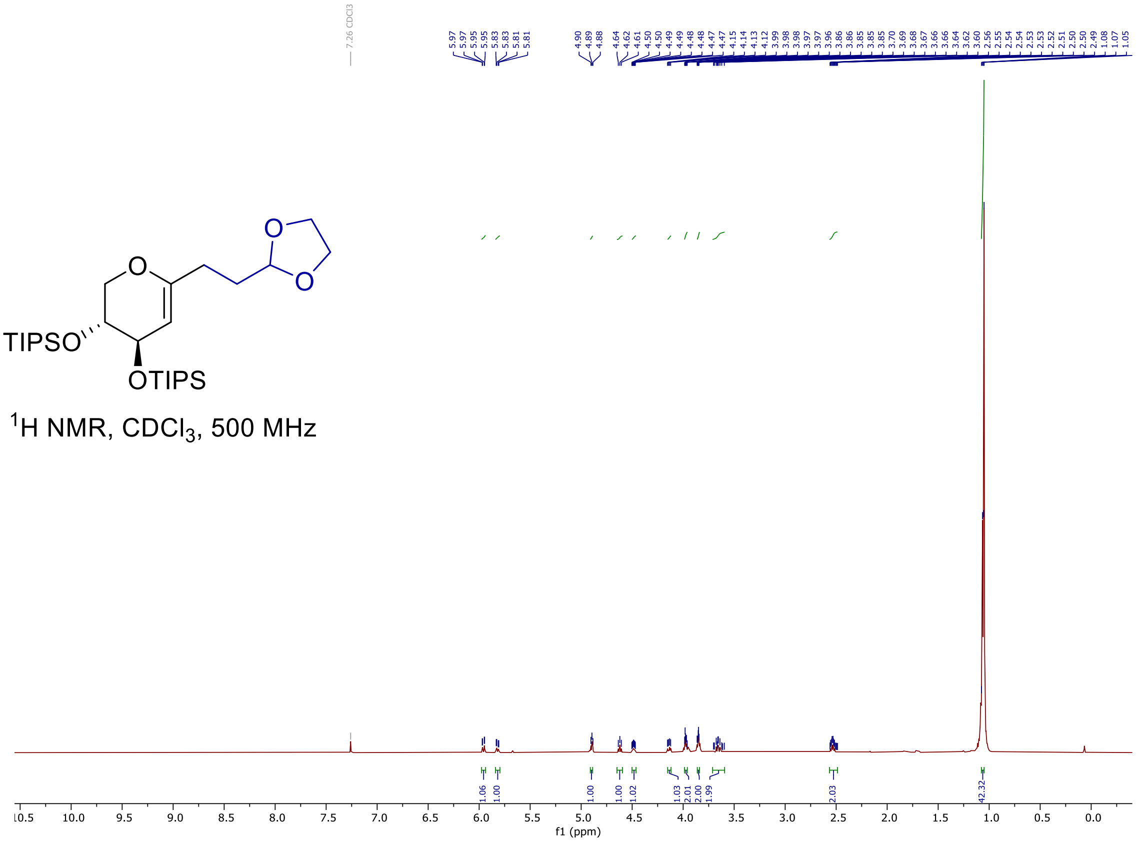

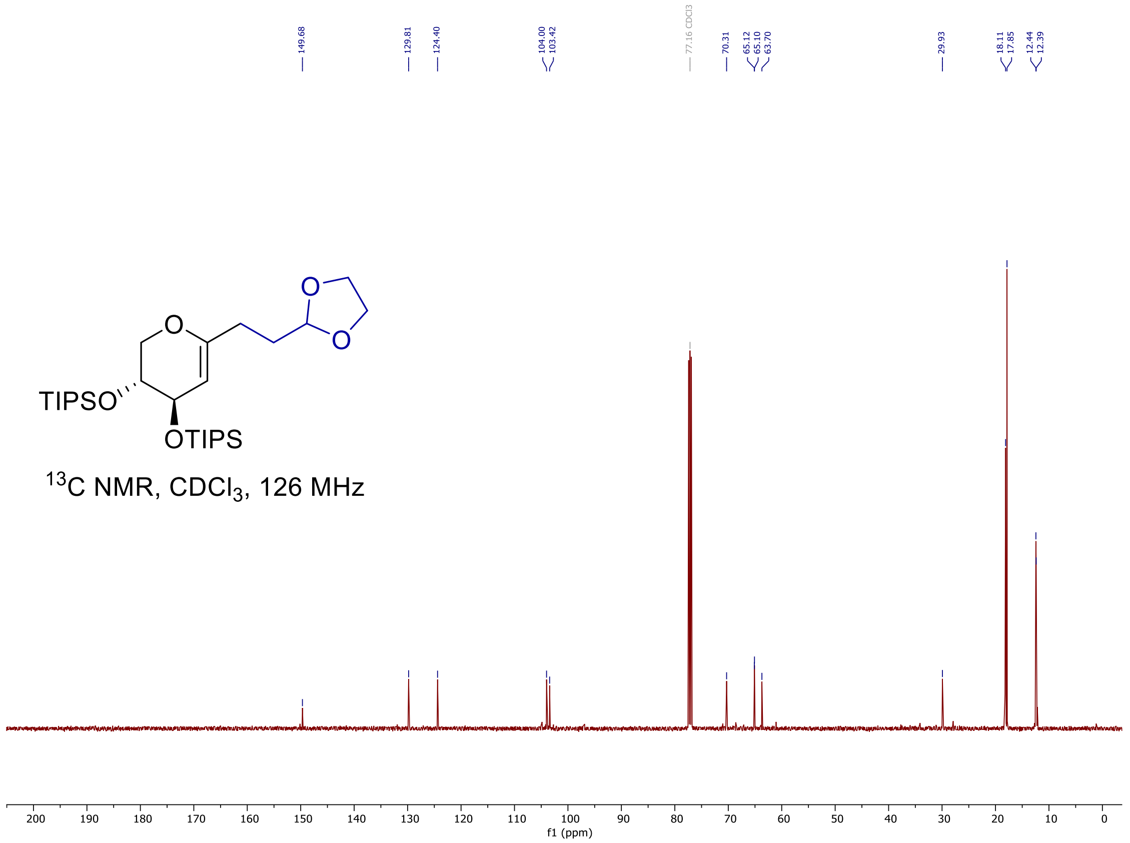

(3a*S*,7a*R*)-6-(4-Methoxynaphthalen-1-yl)-2,2-dimethyl-3a,7a-dihydro-4*H*-[1,3]dioxolo[4,5-*c*]pyran (15a)

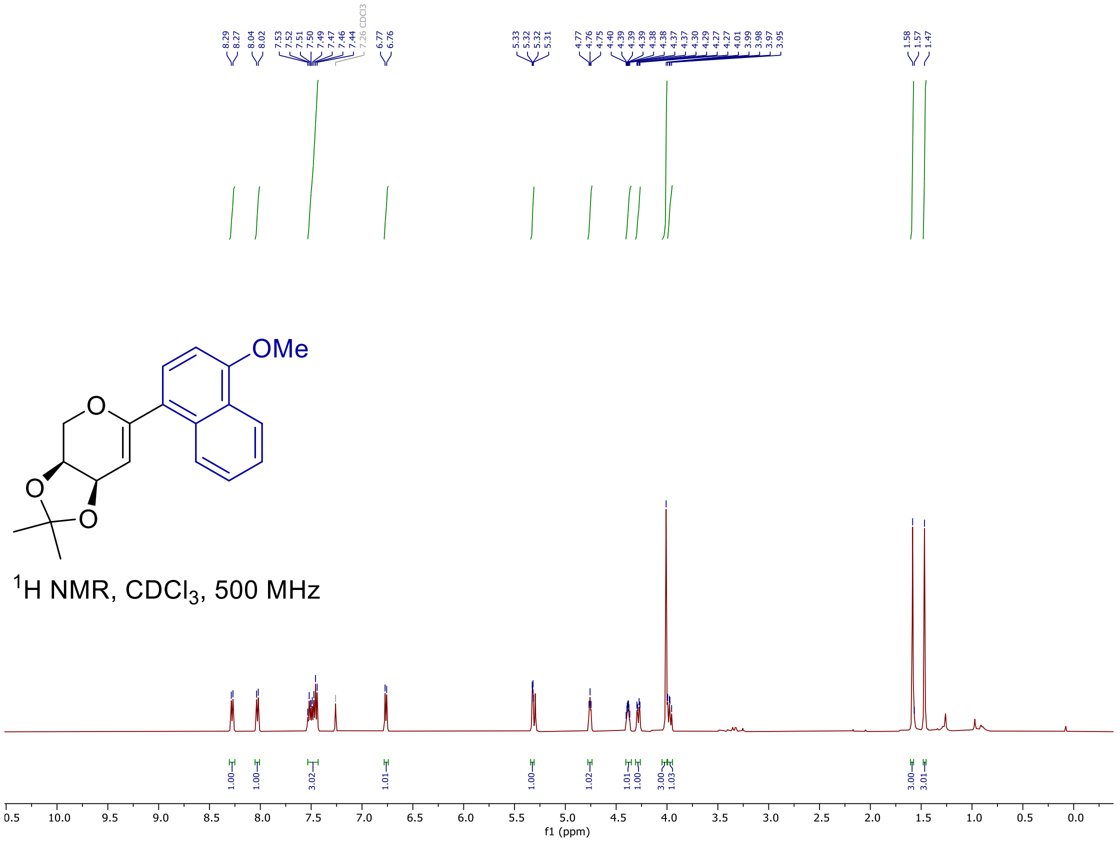

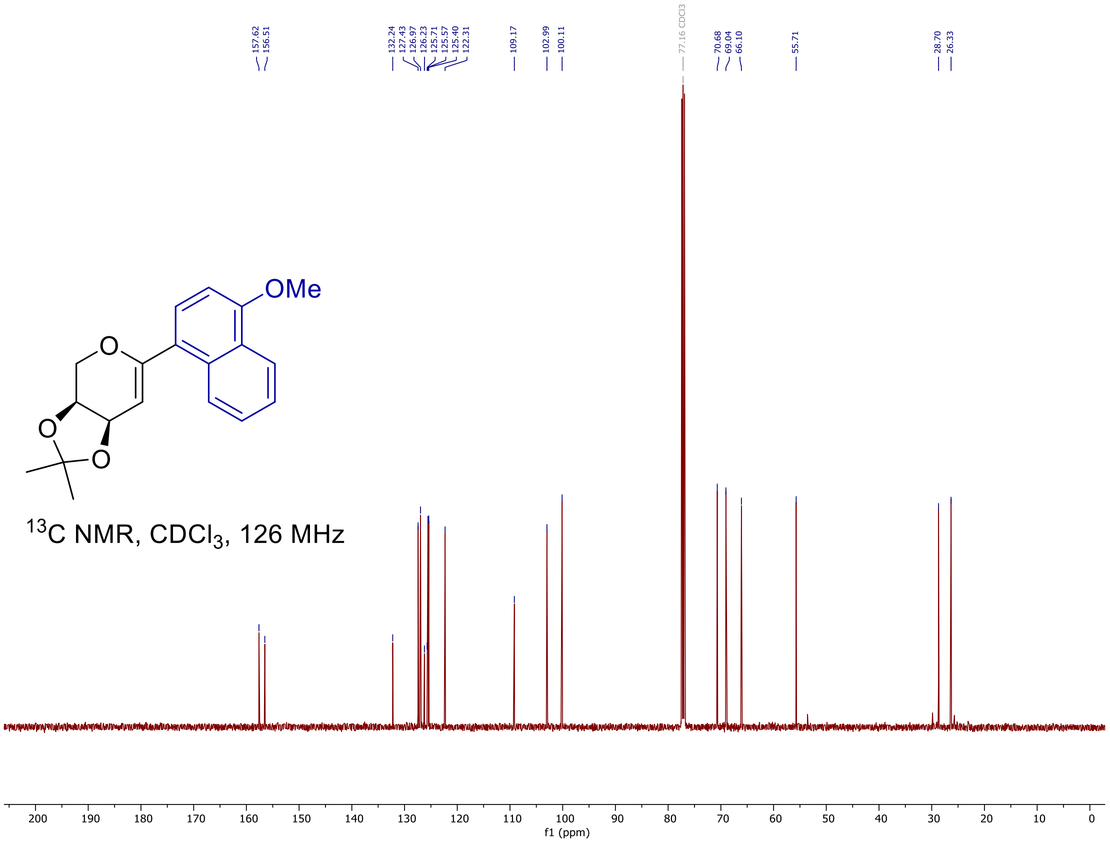

(3a*S*,7a*R*)-2,2-Dimethyl-6-(4-(trifluoromethyl)phenyl)-3a,7a-dihydro-4*H*-[1,3]dioxolo[4,5-*c*]pyran (15b)

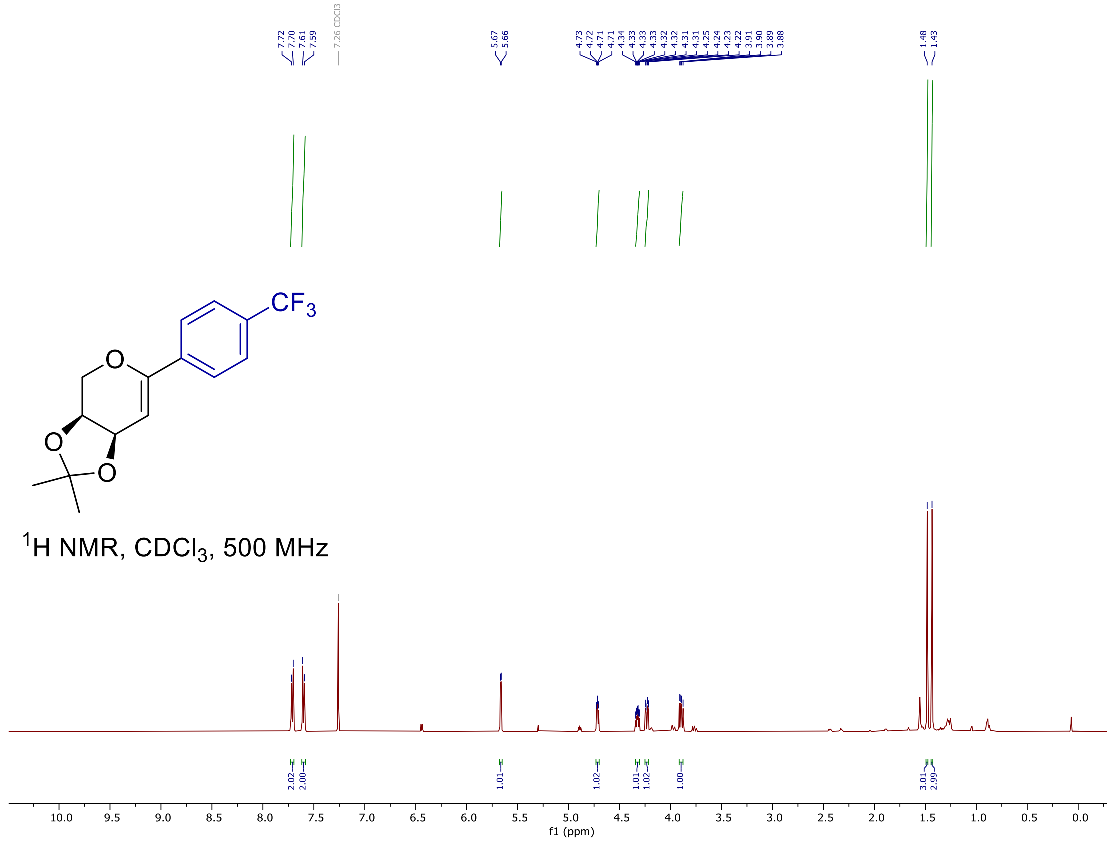

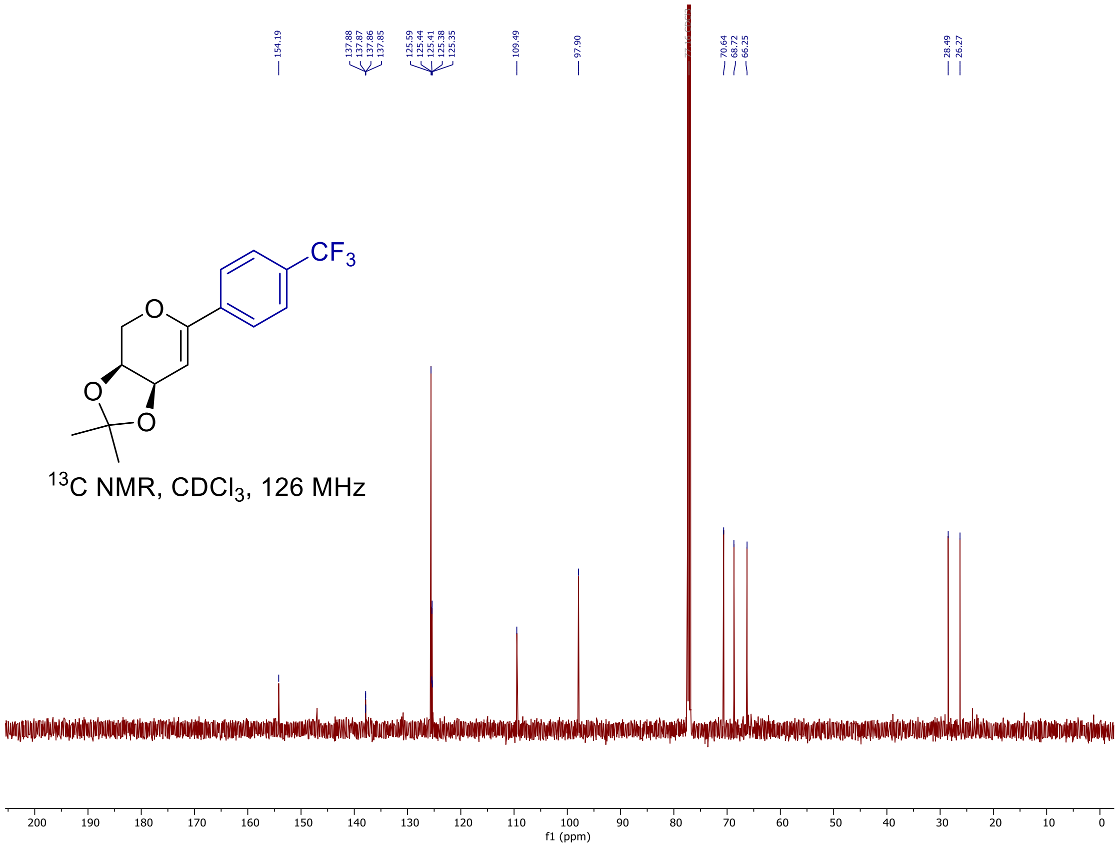

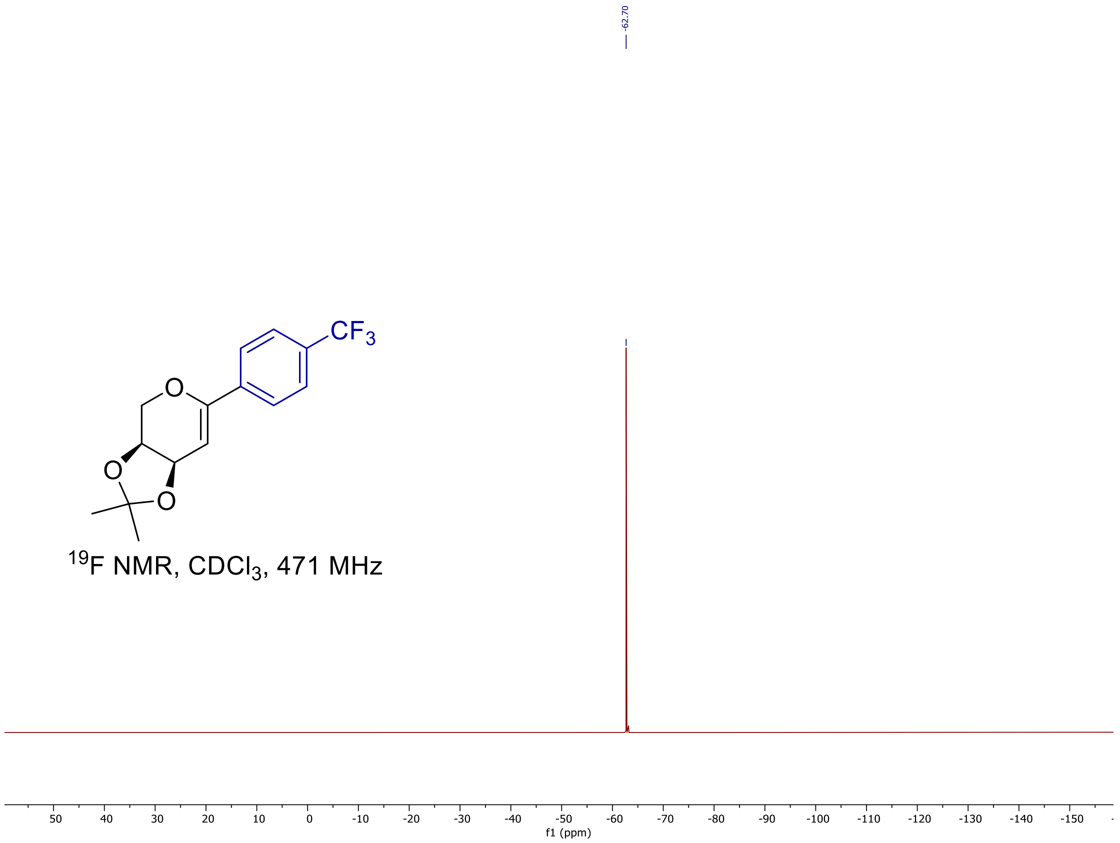
-62.70
CF3
O
O
O
19F NMR, CDCl3, 471 MHz
50 40 30 20 10 0 -10 -20 -30 -40 -50 -60 -70 -80 -90 -100 -110 -120 -130 -140 -150
f1 (ppm)

2-(4-((3a*S*,7a*R*)-2,2-Dimethyl-3a,7a-dihydro-4*H*-[1,3]dioxolo[4,5-*c*]pyran-6-yl)phenyl)-4,4,5,5-tetramethyl-1,3,2-dioxaborolane (15c)

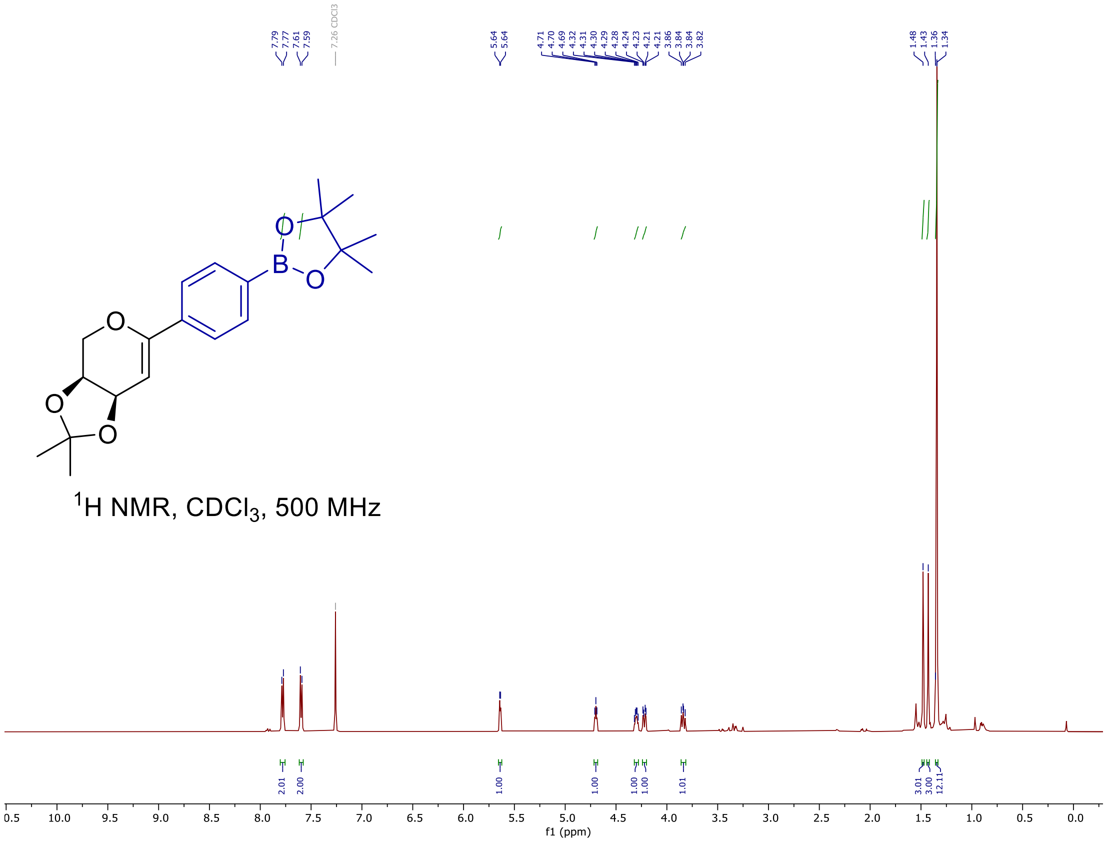

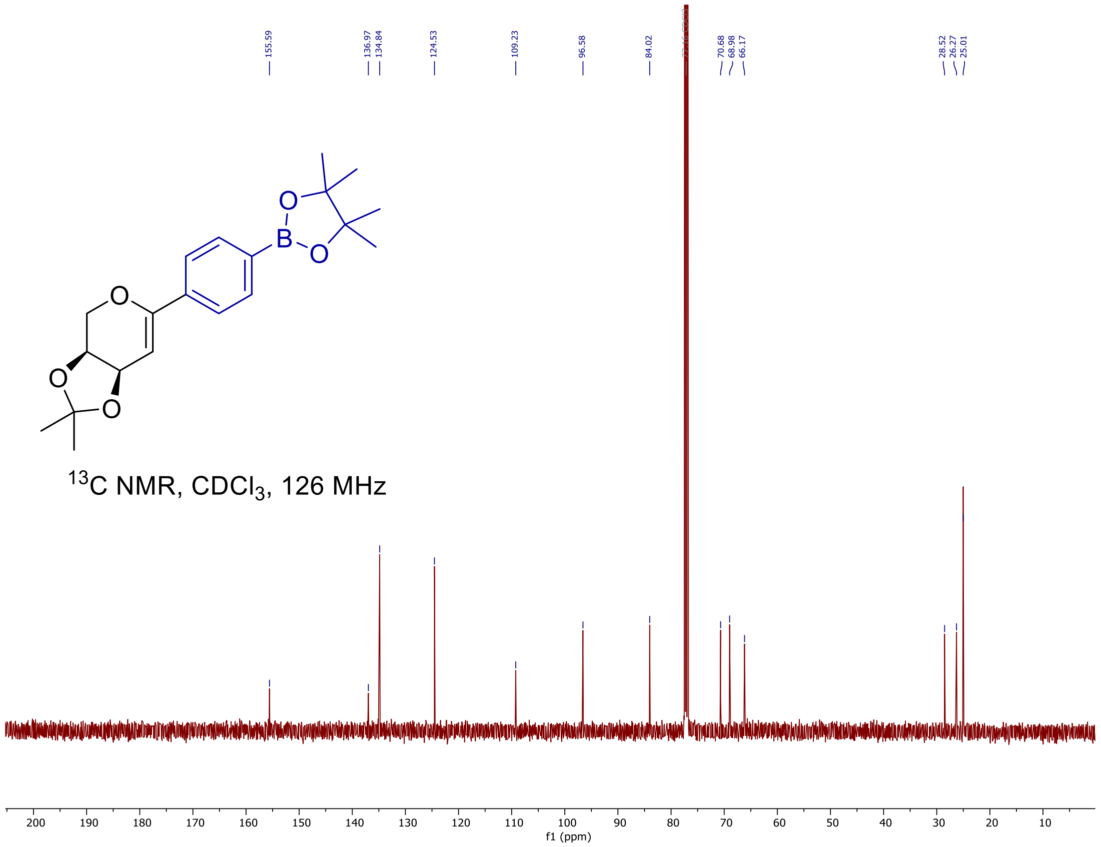

1,4-bis((3a*S*,7a*R*)-2,2-Dimethyl-3a,7a-dihydro-4*H*-[1,3]dioxolo[4,5-*c*]pyran-6-yl)benzene (15d)

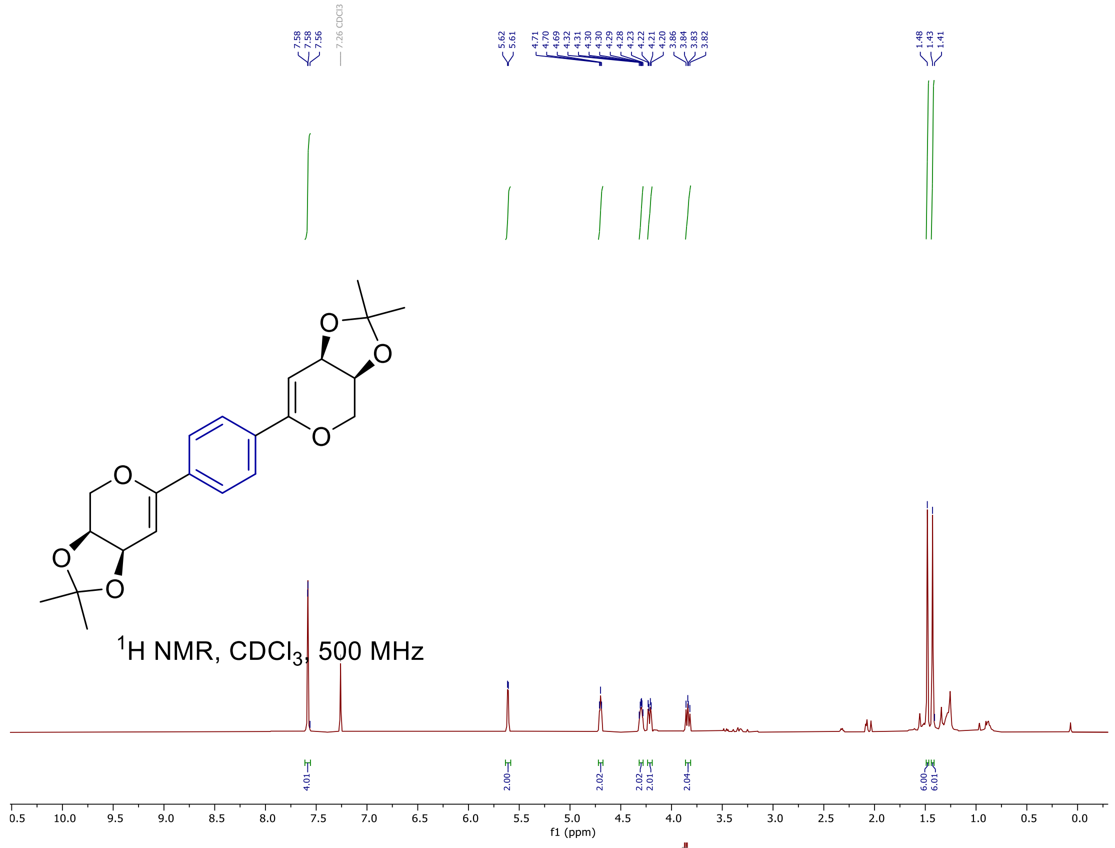

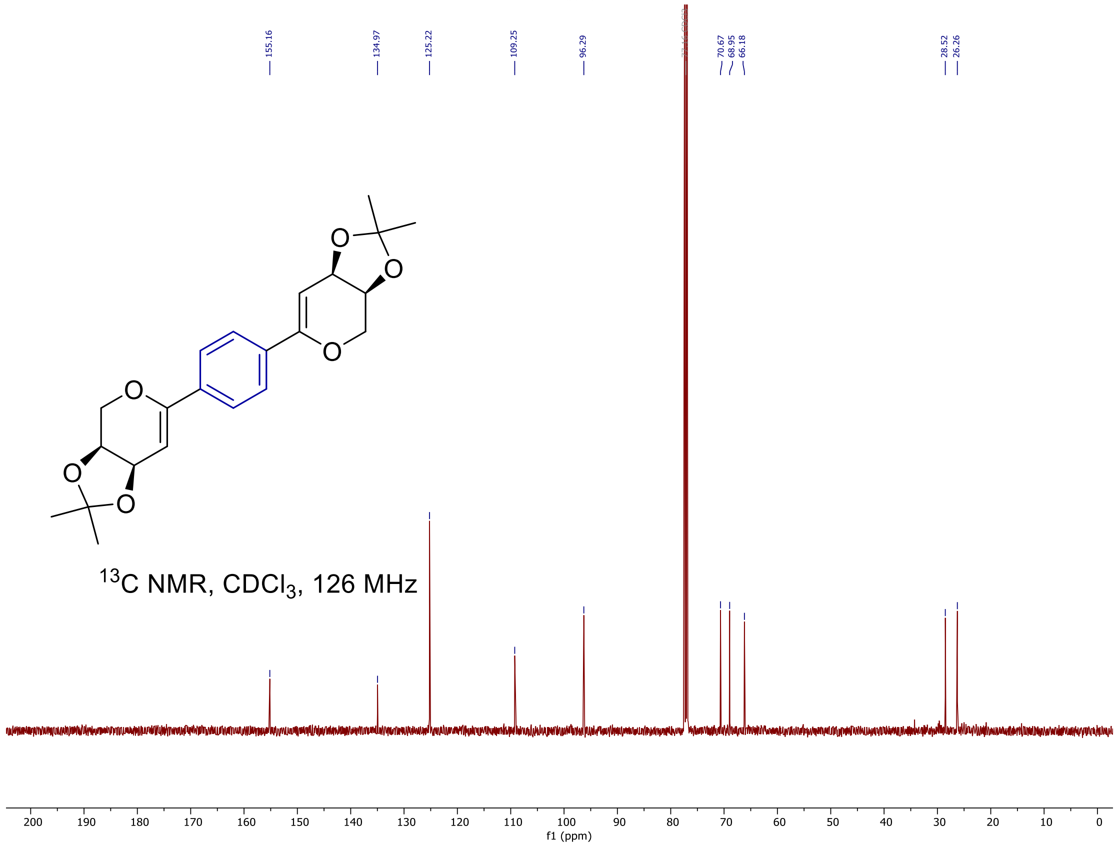

(((3*S*,4*S*,5*R*,6*R*)-1-(4-Chloro-3-(4-(((*S*)-tetrahydrofuran-3-yl)oxy)benzyl)phenyl)-7,7-difluoro-3-methyl-2-oxabicyclo[4.1.0]heptane-4,5-diyl)bis-(oxy))bis-(triisopropylsilane) (16)

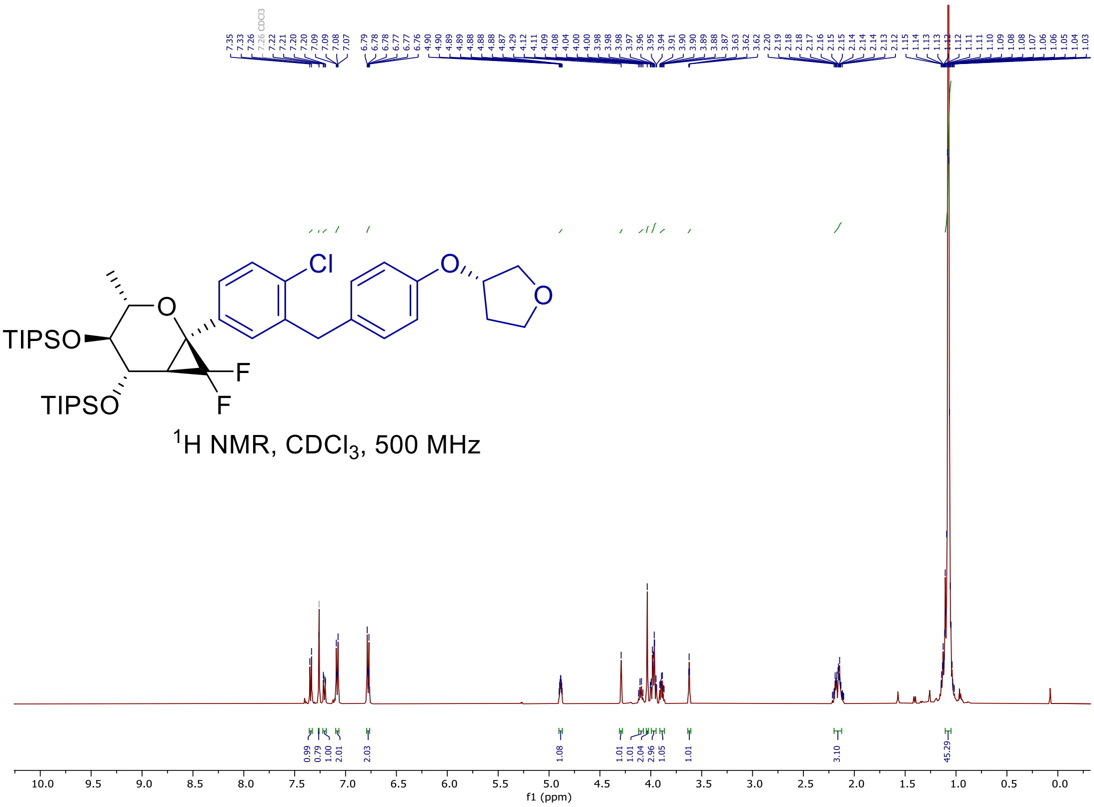

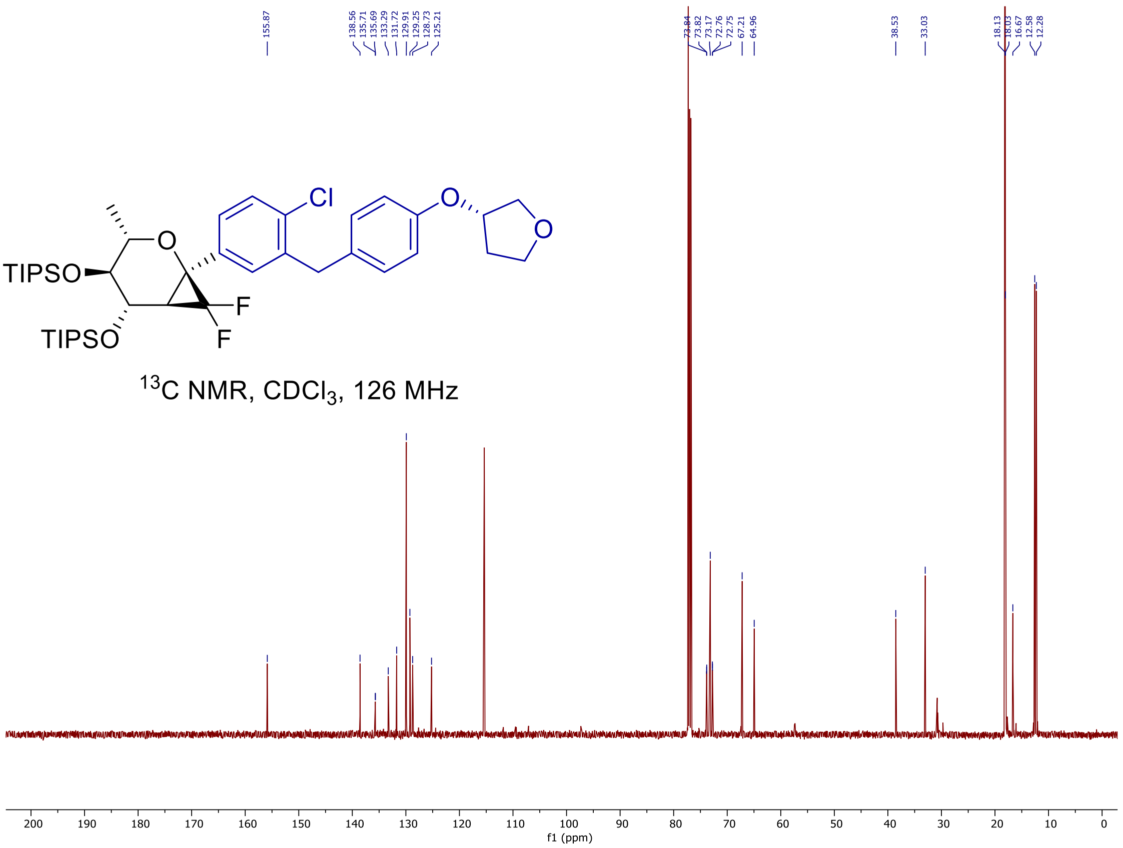
155.87
138.56
135.71
135.69
133.29
131.72
129.91
129.25
128.73
125.21
73.84
73.82
73.17
72.76
72.75
67.21
64.96
38.53
33.03
18.13
18.03
16.67
12.58
12.28
Cl
O
O
O
TIPSO
TIPSO
F
F
13C NMR, CDCl3, 126 MHz
f1 (ppm)

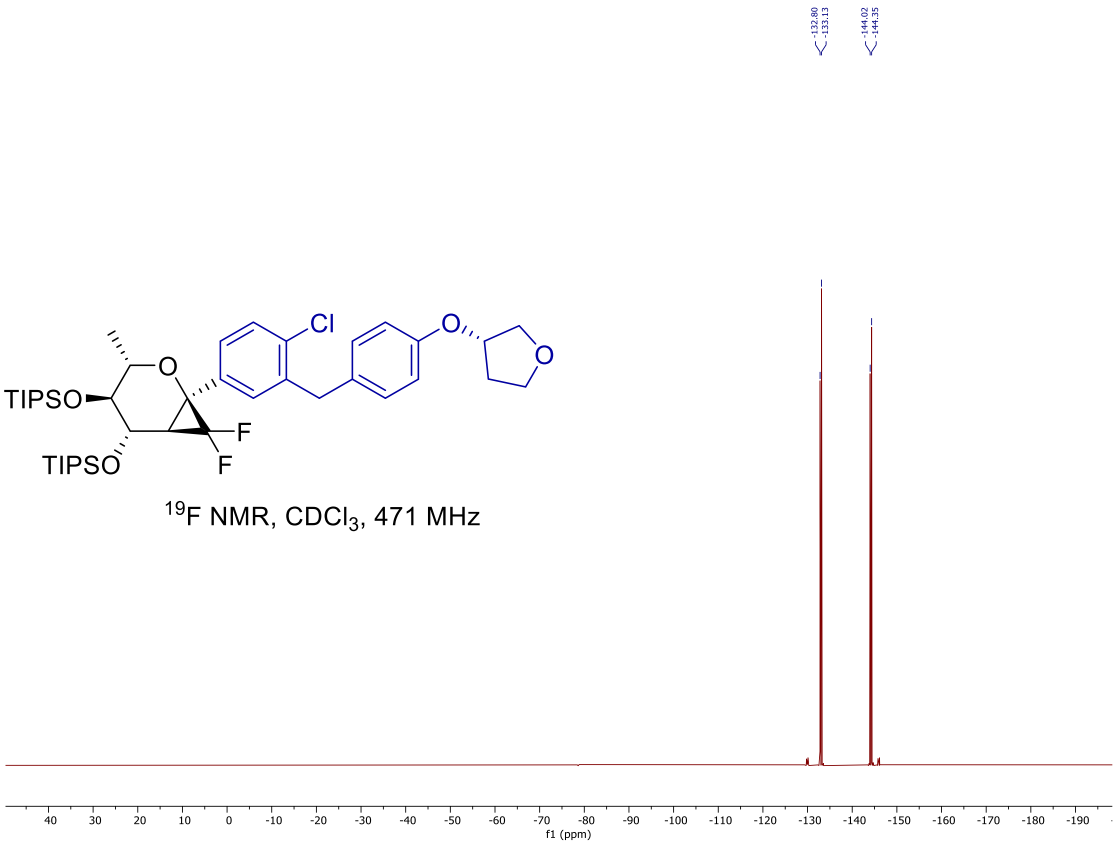
-132.80
-133.13
-144.02
-144.35
Cl
O
O
O
TIPSO
TIPSO
F
F
19F NMR, CDCl3, 471 MHz
f1 (ppm)

(4a*R*,5a*S*,6a*R*,7*R*,7a*R*)-5a-(6-Bromo-2,3,4-trimethoxyphenyl)-2,2-di-*tert*-butyl-7-((triisopropylsilyl)oxy)hexahydrooxireno[2',3':5,6]pyrano[3,2-*d*][1,3,2]dioxasiline (17)

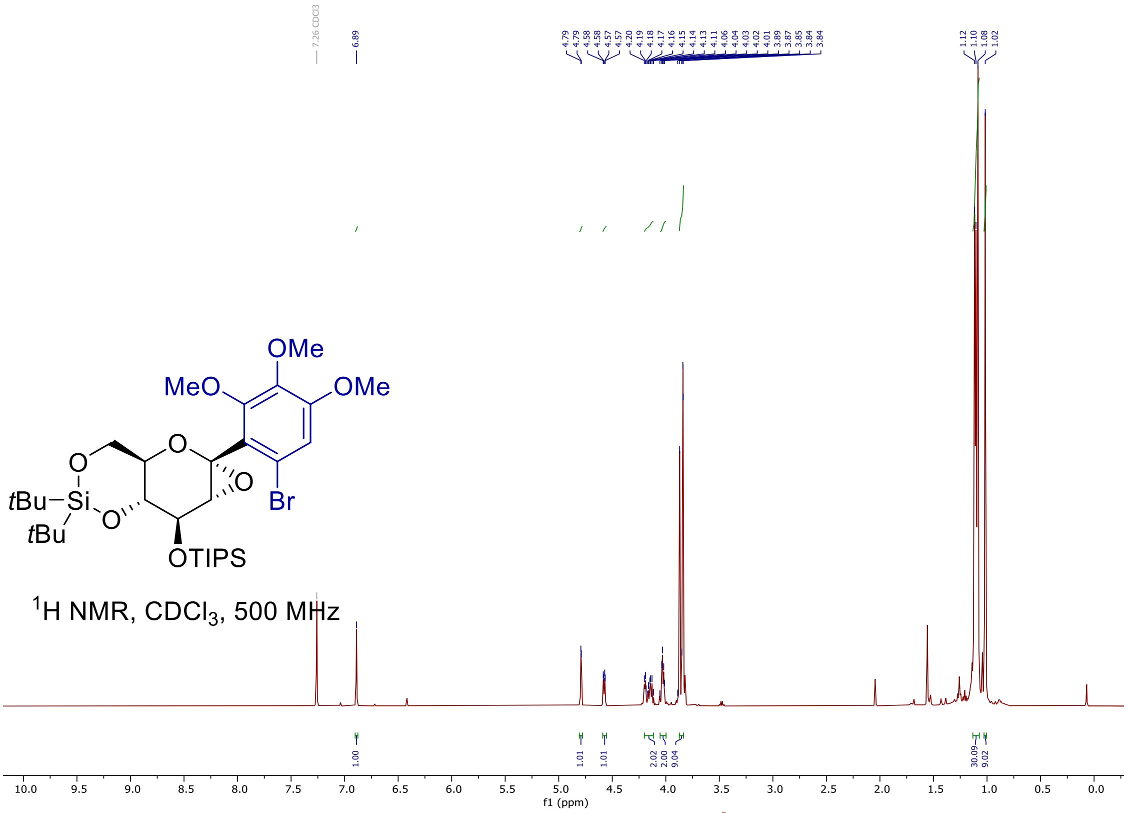

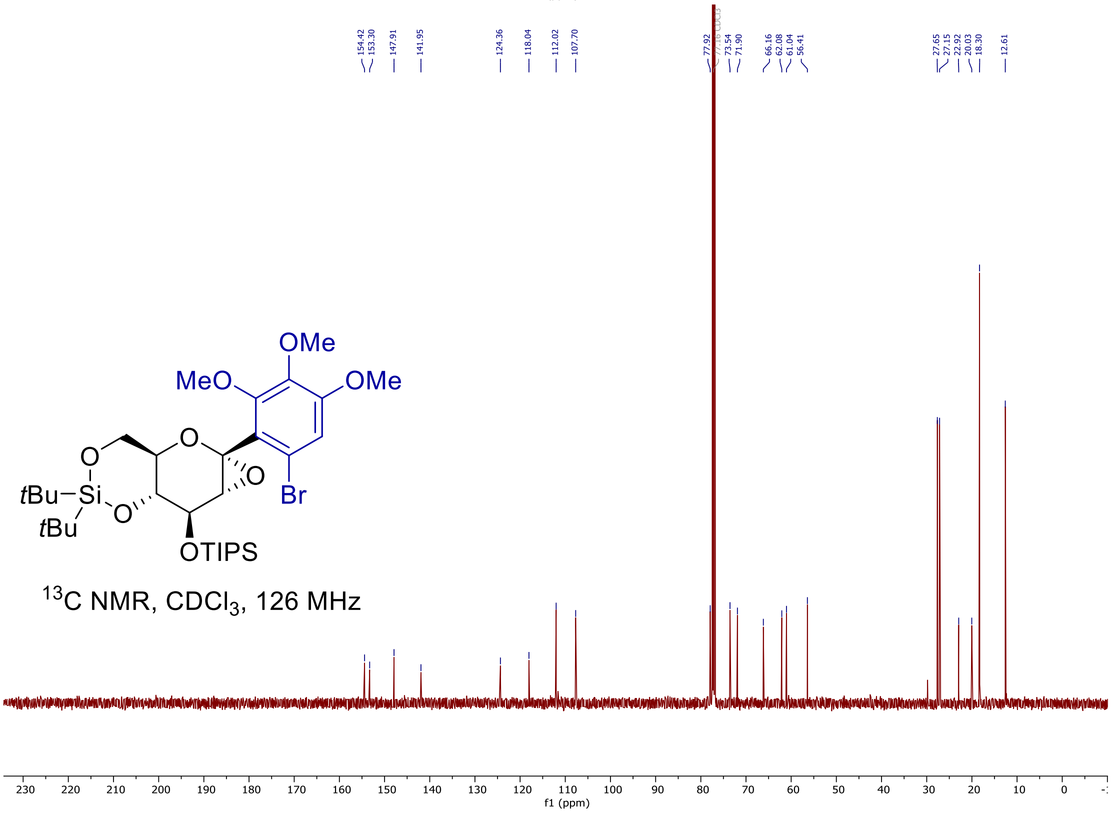

(4a*R*,6*S*,7*S*,8*R*,8a*R*)-2,2-Dimethyl-8-((triisopropylsilyl)oxy)-6-(2,3,4-trimethoxyphenyl)hexahydropyrano[3,2-*d*][1,3]dioxin-7-ol (18)

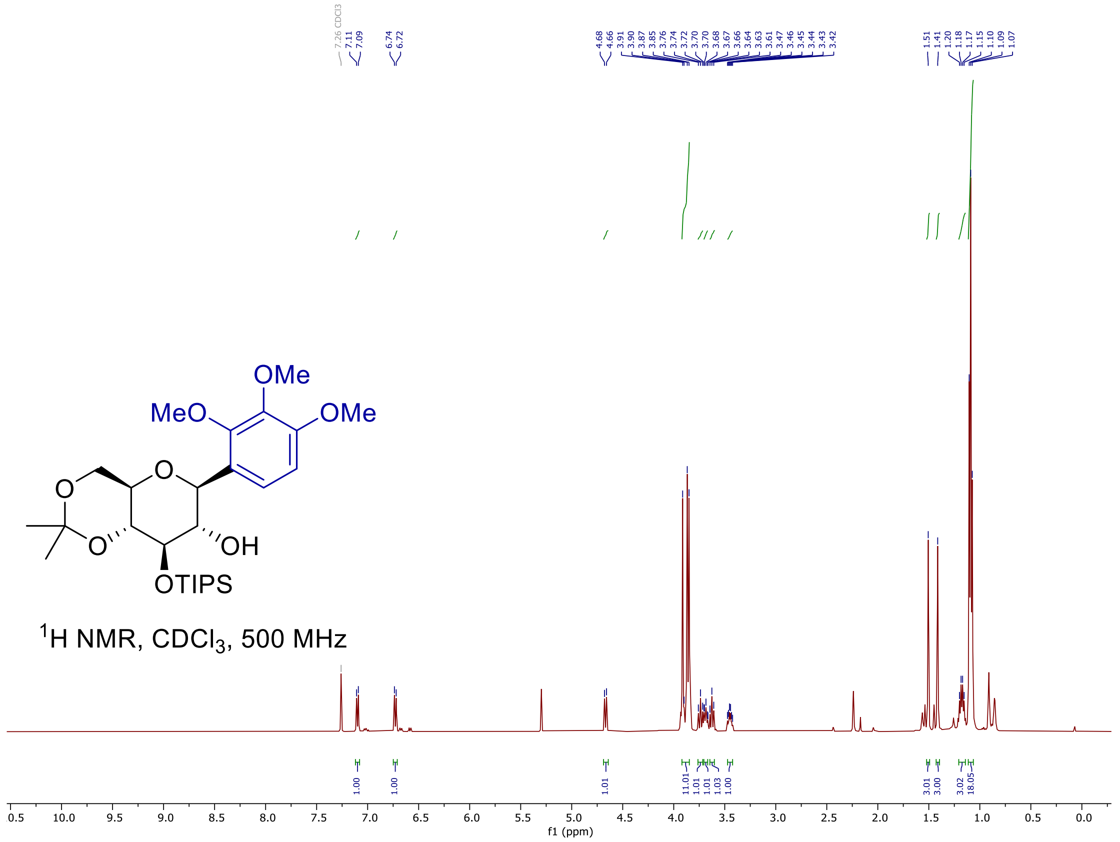

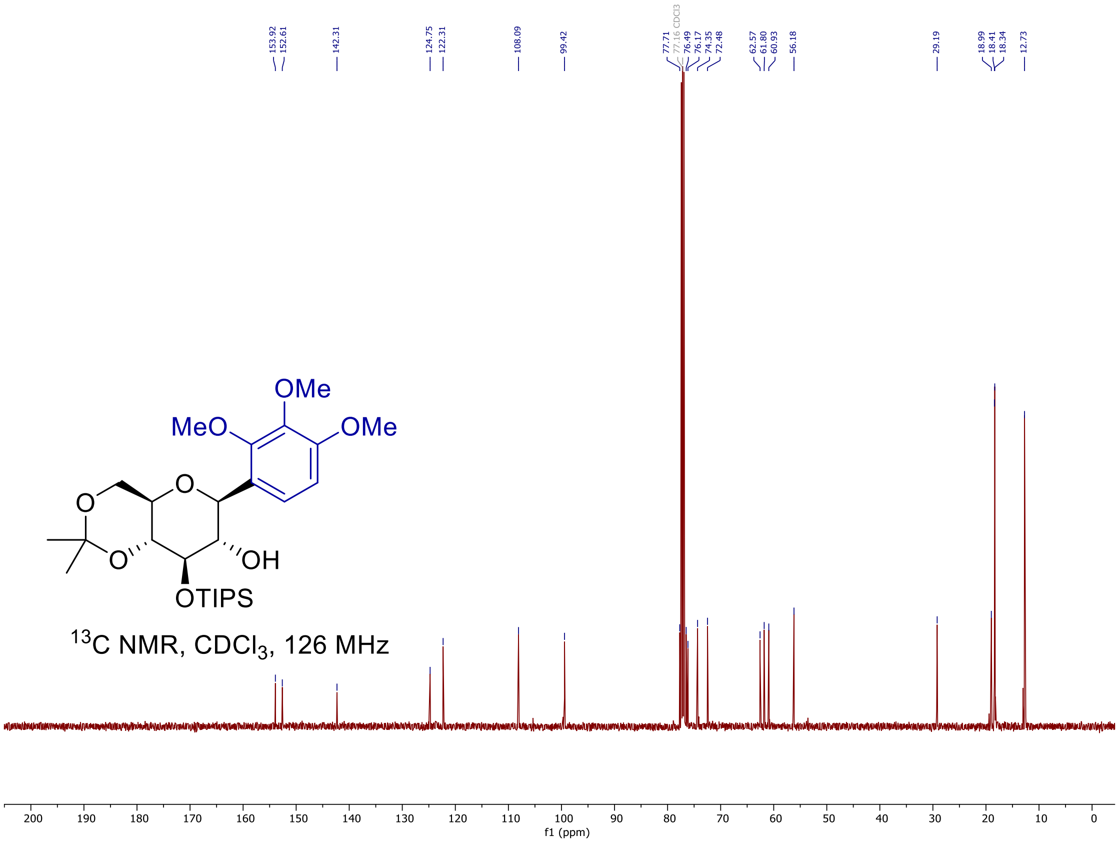

(4a*R*,6*S*,7*S*,8*R*,8a*R*)-2,2-Di-*tert*-butyl-8-((triisopropylsilyl)oxy)-6-(2,3,4-trimethoxy-5-(trimethylsilyl)phenyl)hexahydropyrano[3,2-*d*][1,3,2]dioxasilin-7-ol (<u>19a</u>)

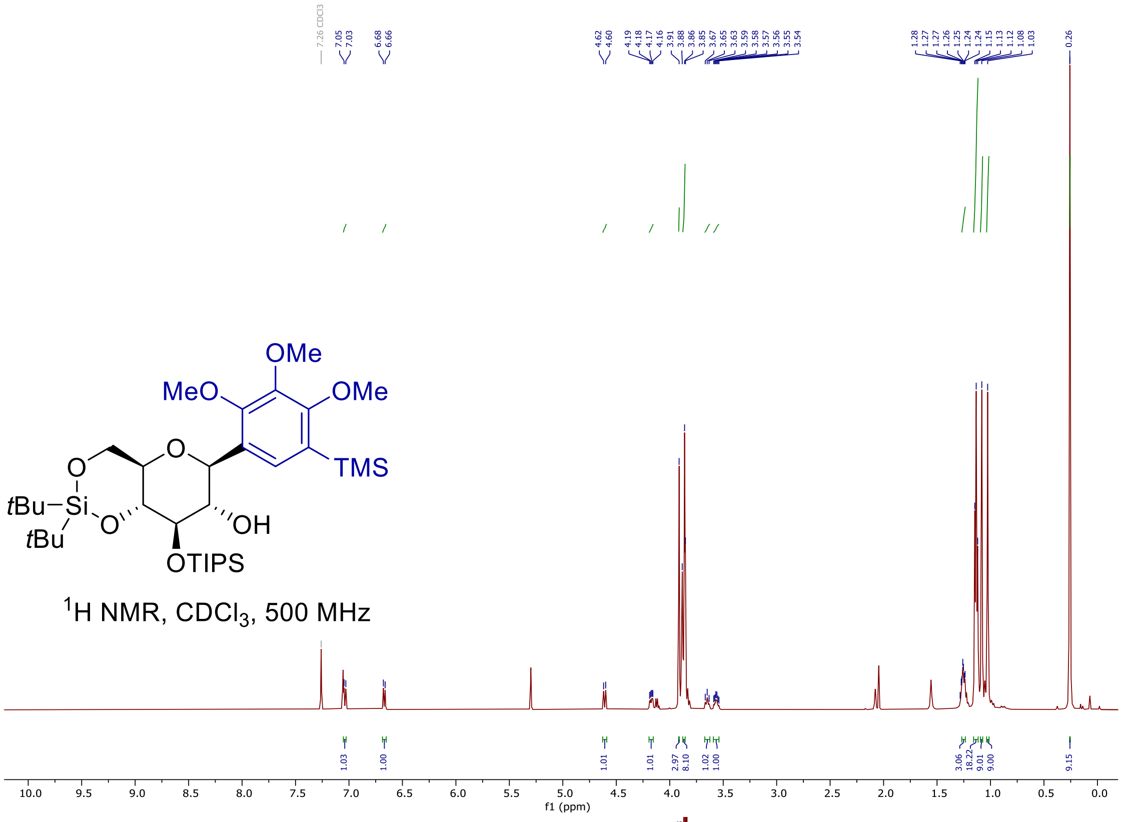

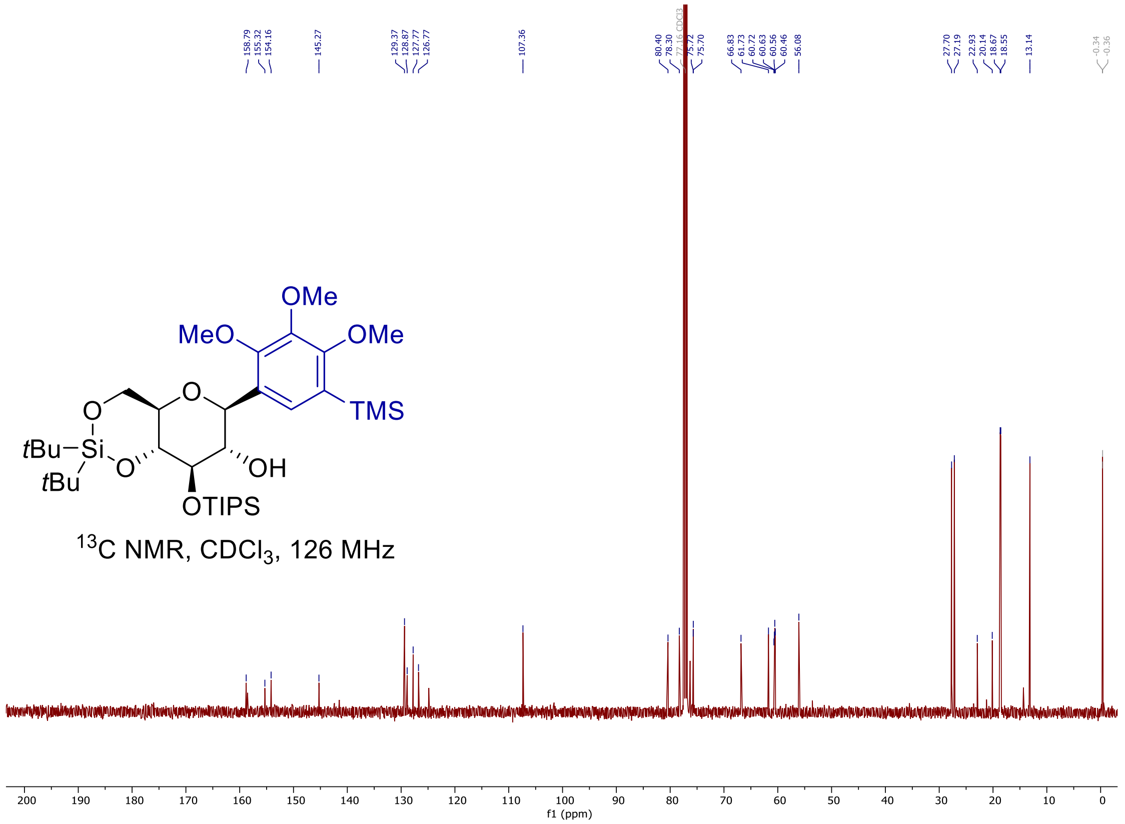

(4a*R*,6*S*,7*S*,8*R*,8a*R*)-2,2-Di-*tert*-butyl-6-(4-chloro-3-(4-(((*S*)-tetrahydrofuran-3-yl)oxy)benzyl)phenyl)-8-((triisopropylsilyl)oxy)hexahydropyrano[3,2-*d*][1,3,2]dioxasilin-7-ol (<u>19b</u>)

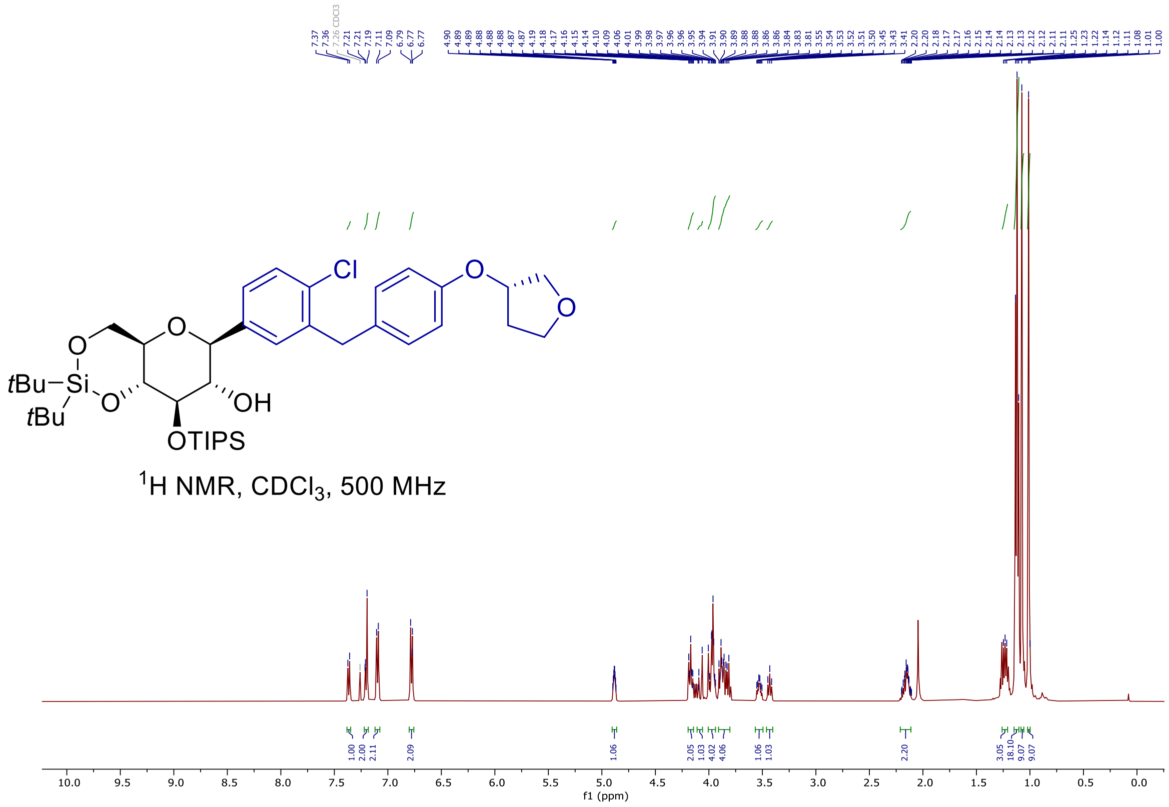

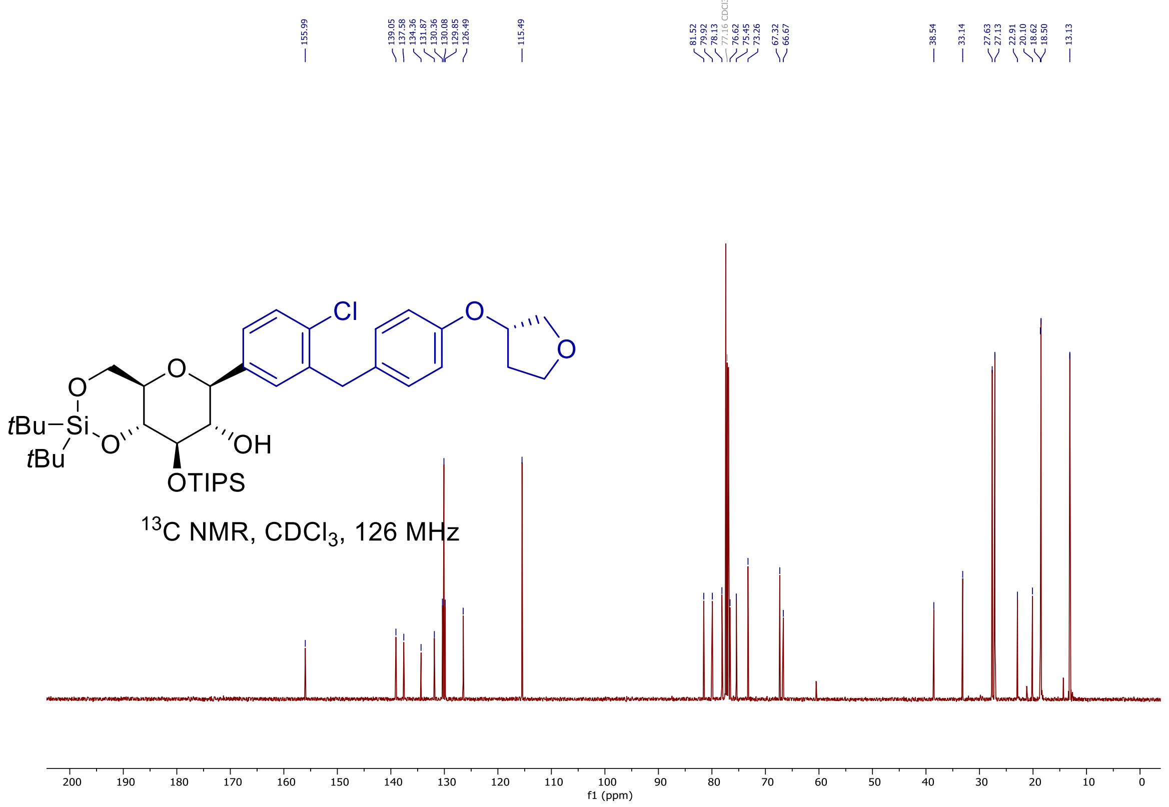

(4a*R*,6*S*,7*S*,8*R*,8a*R*)-2,2-Di-*tert*-butyl-6-(4-chloro-3-(4-ethoxybenzyl)phenyl)-8-((triisopropylsilyl)oxy)hexahydropyrano[3,2-*d*][1,3,2]dioxasilin-7-ol (19c)

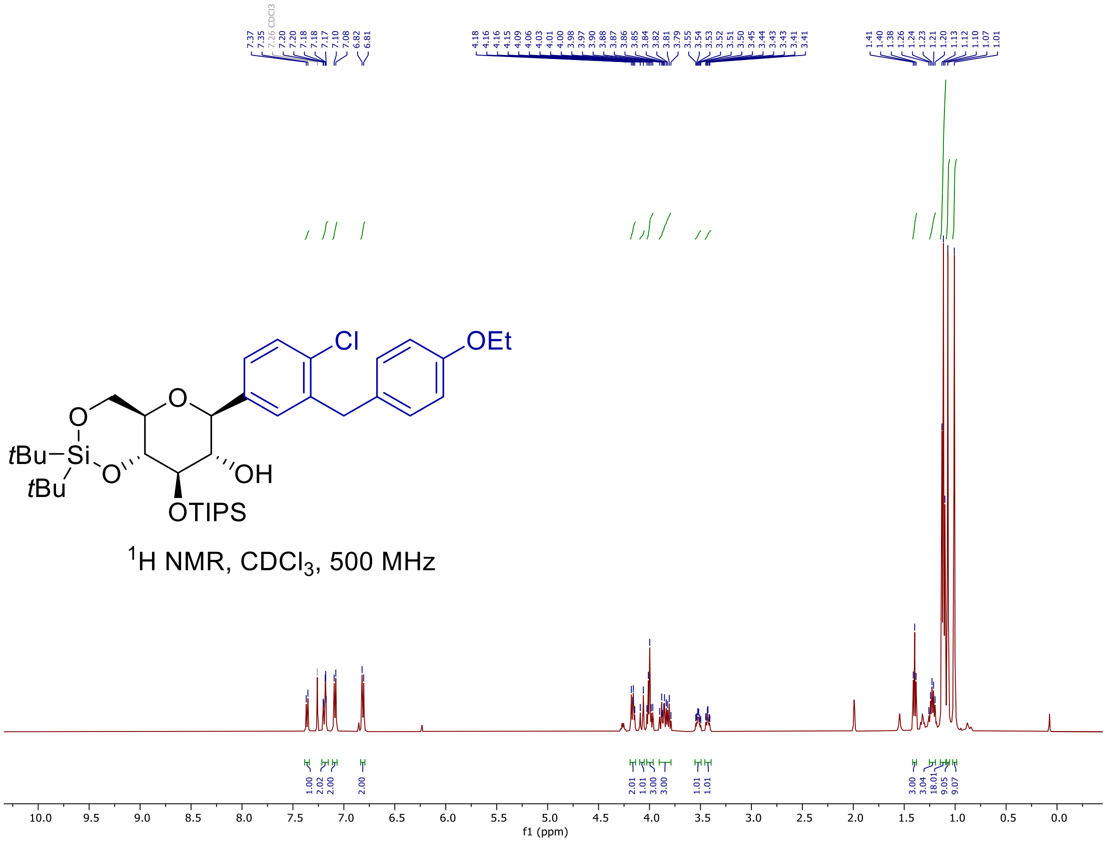

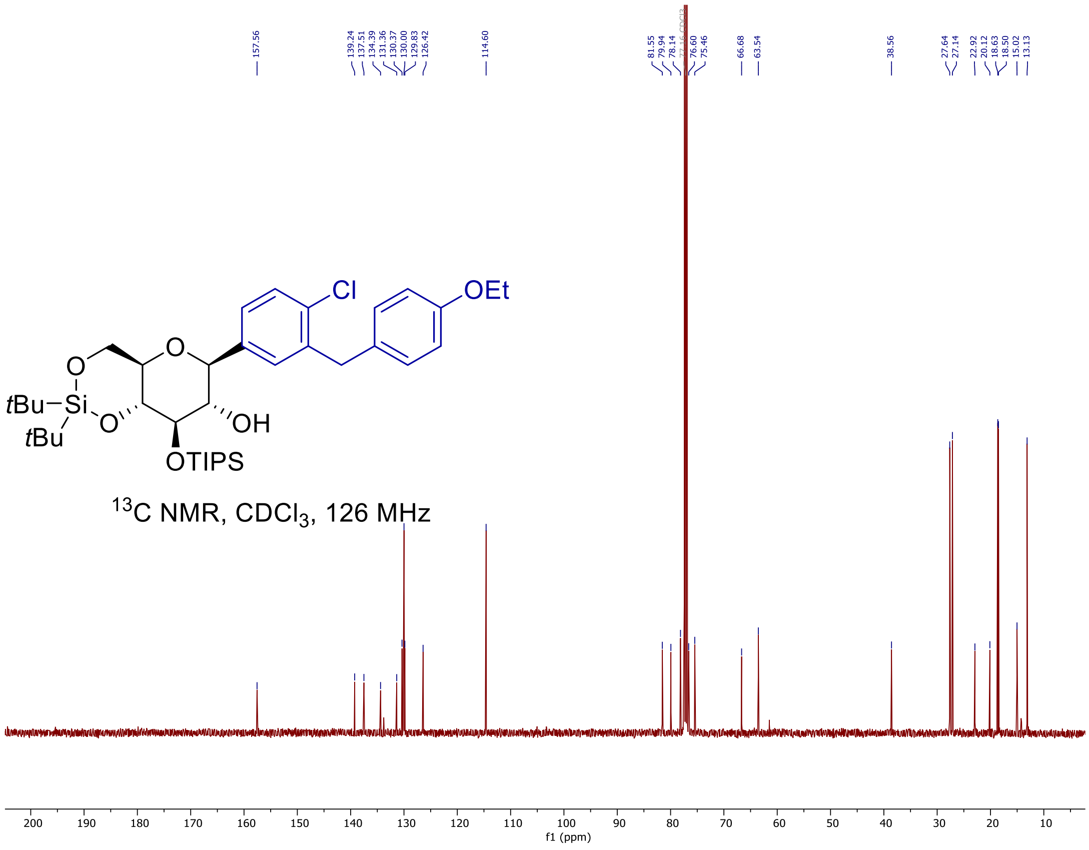

(4a*R*,6*S*,7*S*,8*R*,8a*R*)-2,2-Di-*tert*-butyl-8-((triisopropylsilyl)oxy)-6-(2,3,4-trimethoxyphenyl)hexahydropyrano[3,2-*d*][1,3,2]dioxasilin-7-ol (<u>19d</u>)

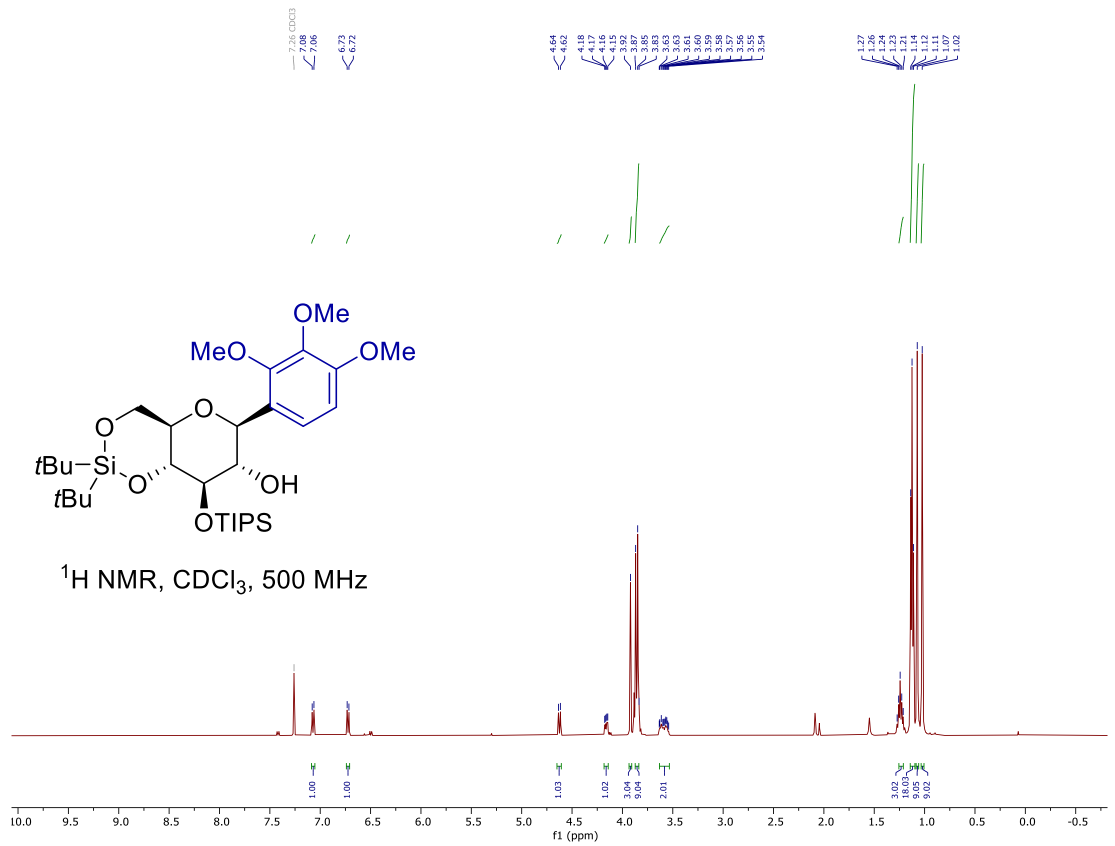

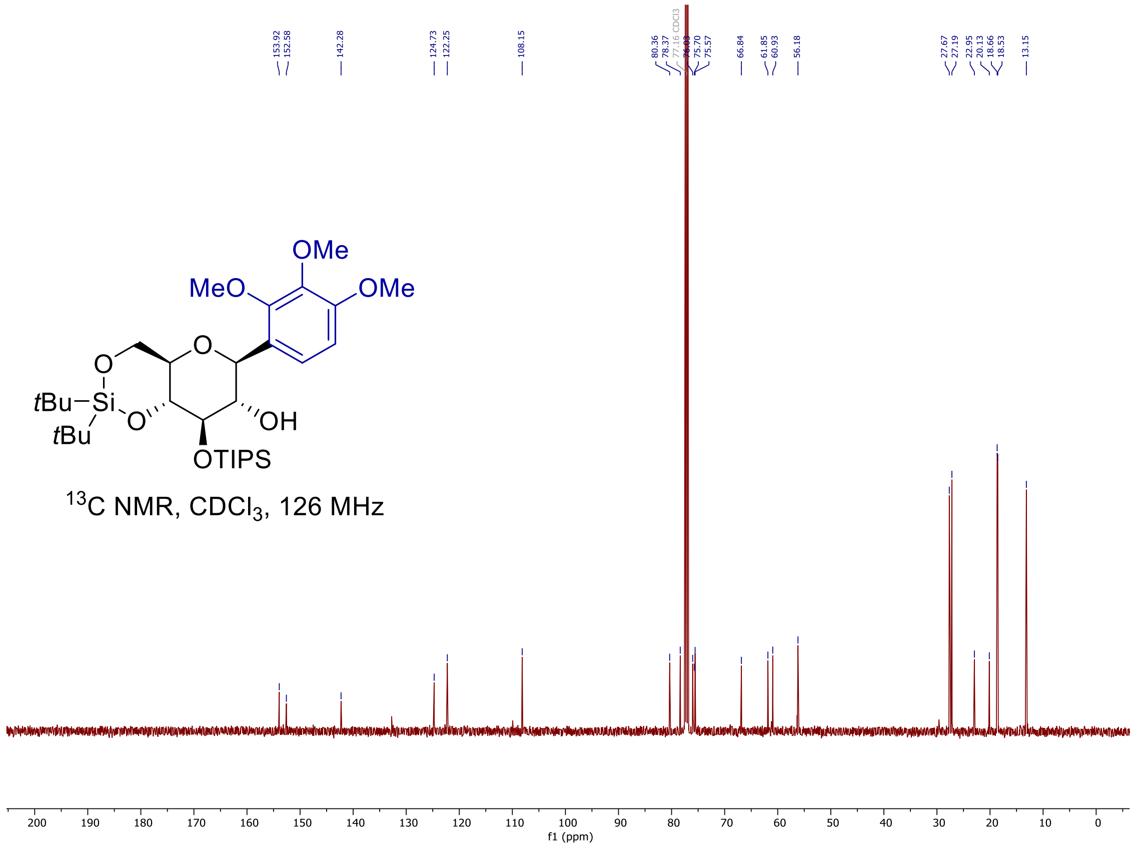

Methyl 5-((4a*R*,6*S*,7*S*,8*R*,8a*R*)-2,2-di-*tert*-butyl-7-hydroxy-8-((triisopropylsilyl)oxy)hexahydropyrano[3,2-*d*][1,3,2]dioxasilin-6-yl)-2,3,4-trimethoxybenzoate (19e)

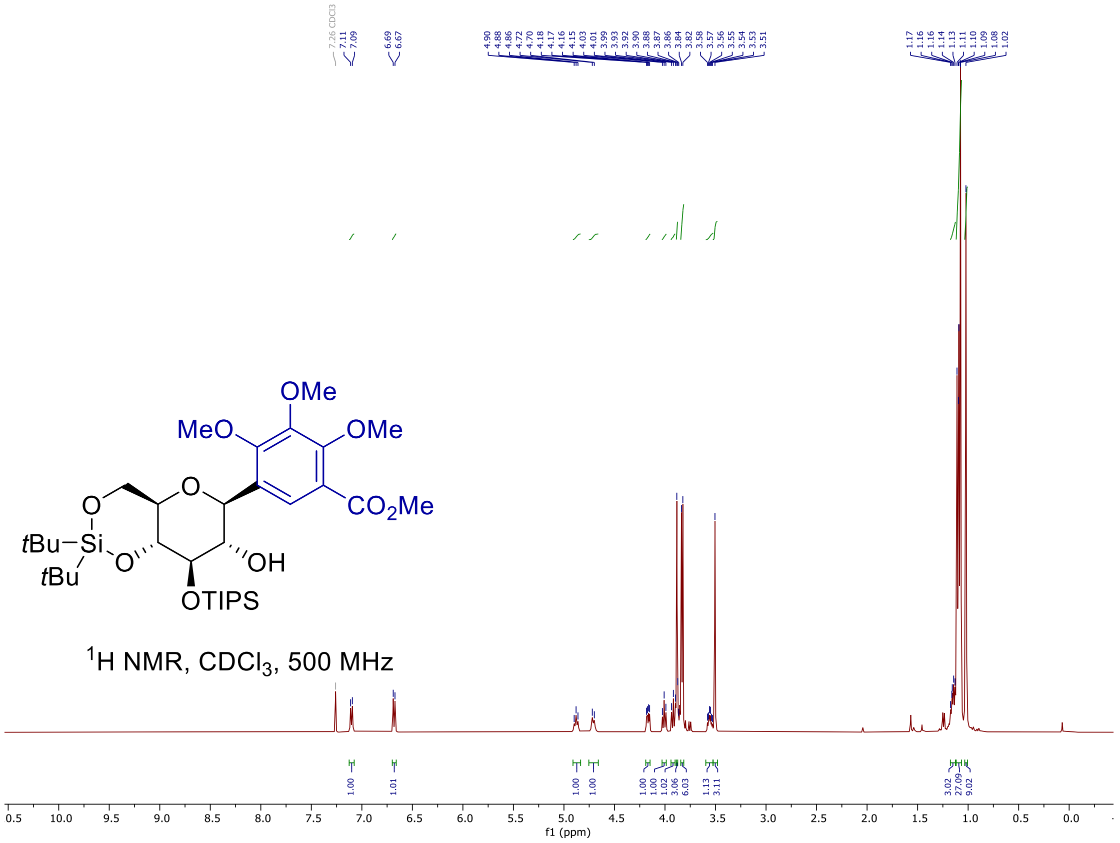

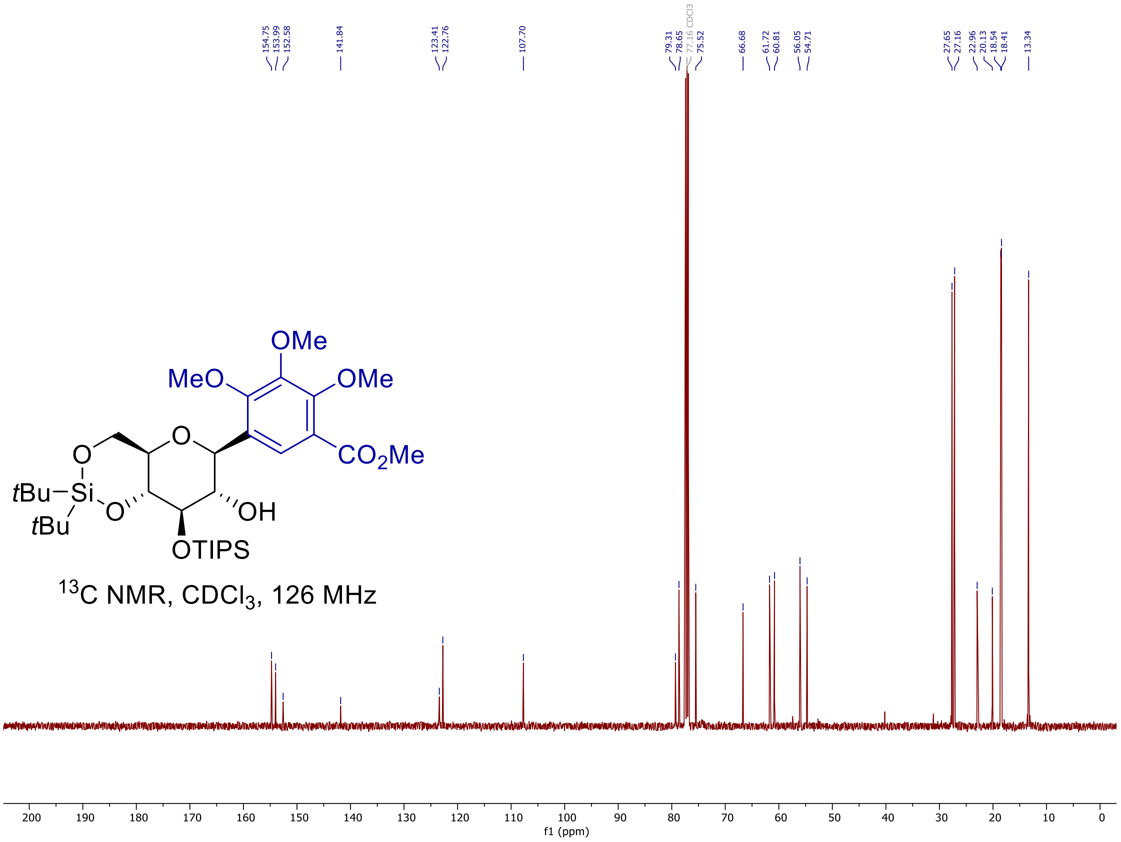

Supplement: Supplementary file 3 — Supplementary Data 1 [file 42004_2024_1339_MOESM3_ESM.pdf]
